# Inhibition of Insulin-Regulated Aminopeptidase by Imidazo [1,5-α]pyridines—Synthesis and Evaluation

**DOI:** 10.3390/ijms25052516

**Published:** 2024-02-21

**Authors:** Karin Engen, Thomas Lundbäck, Anubha Yadav, Sharathna Puthiyaparambath, Ulrika Rosenström, Johan Gising, Annika Jenmalm-Jensen, Mathias Hallberg, Mats Larhed

**Affiliations:** 1 Department of Medicinal Chemistry, Uppsala University, BMC, P.O. Box 574, SE-751 23 Uppsala, Sweden; karin.engen@gmail.com (K.E.); ulrika.rosenstrom@ilk.uu.se (U.R.); 2 Chemical Biology Consortium Sweden (CBCS), Science for Life Laboratory, Department of Medical Biochemistry and Biophysics, Division of Chemical Biology and Genome Engineering, Karolinska Institutet, Tomtebodavägen 23A, SE-171 65 Solna, Sweden; thomas.lundback@astrazeneca.com (T.L.); annika.jensen@scilifelab.se (A.J.-J.); 3 Mechanistic & Structural Biology, Discovery Sciences, R&D, AstraZeneca, SE-431 83 Mölndal, Sweden; 4 The Beijer Laboratory, Science for Life Laboratory, Department of Medicinal Chemistry, Uppsala University, BMC, P.O. Box 574, SE-751 23 Uppsala, Sweden; anubha.yadav@ilk.uu.se (A.Y.); sharathna.puthiyaparambath@ilk.uu.se (S.P.); johan.gising@ilk.uu.se (J.G.); 5 The Beijer Laboratory, Department of Pharmaceutical Biosciences, Neuropharmacology and Addiction Research, Uppsala University, BMC, P.O. Box 591, SE-751 24 Uppsala, Sweden; mathias.hallberg@uu.se

**Keywords:** IRAP, imidazo [1,5-α]pyridine-based inhibitors, enzyme inhibitor, insulin-regulated aminopeptidase

## Abstract

Inhibition of insulin-regulated aminopeptidase (IRAP) has been shown to improve cognitive functions in several animal models. Recently, we performed a screening campaign of approximately 10,000 compounds, identifying novel small-molecule-based compounds acting as inhibitors of the enzymatic activity of IRAP. Here we report on the chemical synthesis, structure-activity relationships (SAR) and initial characterization of physicochemical properties of a series of 48 imidazo [1,5-α]pyridine-based inhibitors, including delineation of their mode of action as non-competitive inhibitors with a small L-leucine-based IRAP substrate. The best compound displays an IC_50_ value of 1.0 µM. We elucidate the importance of two chiral sites in these molecules and find they have little impact on the compound’s metabolic stability or physicochemical properties. The carbonyl group of a central urea moiety was initially believed to mimic substrate binding to a catalytically important Zn^2+^ ion in the active site, although the plausibility of this binding hypothesis is challenged by observation of excellent selectivity versus the closely related aminopeptidase N (APN). Taken together with the non-competitive inhibition pattern, we also consider an alternative model of allosteric binding.

## 1. Introduction

Neurodegenerative disorders represent many currently incurable diseases that are rapidly rising in prevalence, in part due to an increasing elderly population. Consequently, effective pharmacotherapies and the development of new treatment approaches are desperately needed. For several years, our laboratory has been interested in investigating the biological properties of the hexapeptide angiotensin IV (Ang IV). Intracerebroventricular injections of Ang IV have consistently demonstrated the enhancement of memory and learning across various animal models [1,2,3,4,5,6,7]. Ang IV and structurally related analogs are proposed to exert their actions by inhibition of insulin-regulated aminopeptidase (IRAP), an enzyme belonging to the M1-family of aminopeptidases [8,9,10]. IRAP is abundantly expressed in areas of the brain associated with cognition, such as the amygdala, hippocampus and cerebral cortex [11,12,13]. One of the main hypotheses by which IRAP inhibition improves cognition originates from the prevention of IRAP-mediated processing of macrocyclic peptides, such as vasopressin and oxytocin, as these are known to improve parameters associated with cognition [14,15,16,17]. Inhibition of IRAP could potentially prolong the half-lives of these peptides to furnish the observed memory-enhancing effects. It has also been proposed that IRAP inhibition might facilitate glucose uptake in neurons by regulating GLUT4 translocation, and these two mechanisms constitute the major hypotheses by which IRAP inhibition improves memory and learning [18,19,20,21,22,23,24,25]. Additionally, IRAP, together with ERAP1 and ERAP2, two other M1 aminopeptidases, have been demonstrated to be involved in the regulation of adaptive immune response, such as trimming of antigenic peptides and trafficking of T-cell receptors [26,27,28,29,30].

**Figure 1:**
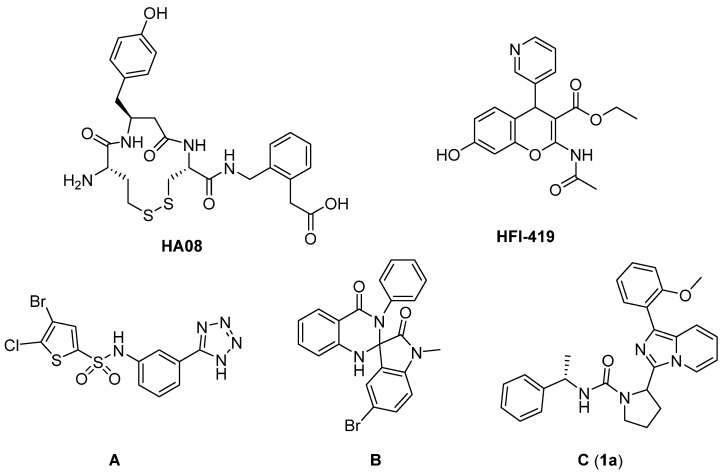
IRAP inhibitors HA08, HFI-419 and exemplified chemical cluster (**A**–**C**).

The potential of IRAP as a target for pharmaceutical agents aimed at treating cognitive disorders has attracted increasing interest over the last decade. Several efforts to develop potent inhibitors as research tools for further investigations into the memory-enhancing effects of IRAP inhibition have been undertaken. The majority of published inhibitors have been peptides or pseudopeptides. These include compounds incorporating *β*-homo amino acids and constrained amino acid analogs of Ang IV, or peptide-like transition state mimicking compounds. Notably, these inhibitors have consistently demonstrated excellent potency and a relatively good selectivity profile toward other aminopeptidases [26,27,28,29,30,31,32,33,34,35,36,37]. Our group has used the structures of Ang IV and the IRAP substrate oxytocin to design both linear and macrocyclic inhibitors with high potency and stability [38,39,40]. One of the most potent macrocycles, **HA08**, enhances dendritic spine density (DSD) in rat hippocampal primary cultures and alters the spine morphology, a process associated with memory facilitation (Figure 1) [41]. The close correlation between DSD and learning in vivo is well-established in a variety of behavioral studies [42,43,44,45,46]. Although displaying high potency and selectivity, these peptide-based inhibitors are foreseen to suffer from low bioavailability and are consequently of limited use as in vivo tools to study IRAP inhibition in models of cognition.

A virtual screening approach was used by Albiston and Chai to identify the first class of drug-like IRAP inhibitors. A series of benzopyran-based compounds were synthesized, e.g., **HFI-419** (Figure 1), and displayed in vivo efficacy by dose-dependent improvement of the visual recognition task in rats [47,48]. Lateral ventricle administration of the inhibitors also improved spatial working memory. These results inspired us to initiate efforts to identify new classes of inhibitors with alternative chemical structures. An assay based on IRAP naturally expressed in Chinese Hamster Ovary (CHO) cells was used in a screen of approximately 10,500 lead-like and drug-like compounds using the in-house Chemical Biology Consortium Sweden (CBCS) compound library. Through this effort, we identified three novel chemical clusters of IRAP inhibitors, exemplified by (Figure 1A–C), with representatives in the low µM range [49]. The structure activity relationship (SAR) of an aryl-sulfonamide-based class of inhibitors (A) has been published [50]. Additionally, we could demonstrate that these compounds, similar to **HA08** and **HFI-419**, can increase the number of mushroom-shaped spines, a morphology associated with memory enhancement [51].

Synthetic procedures to obtain analogs of a second hit compound, comprising a spiro-oxindole dihydroquinazolinone scaffold (Figure 1B), have also been disclosed [52]. This series of uncompetitive IRAP inhibitors includes representatives with sub-µM affinity [53]. Herein, we report the synthesis and initial SAR of the third cluster of IRAP-inhibitors (**C**) encompassing an imidazopyridine scaffold where the hit compound (**1a**) exhibited an IC_50_ of 2.9 µM (pIC_50_ of 5.5) [49].

## 2. Results and Discussions

Following the primary compound library screen, which identified **1a** as a novel IRAP inhibitor, we evaluated the parent CBCS library for structural analogs with inhibitory capacity (Table 1). Shortly, the analogs demonstrated tolerance for modifications on the phenyl ring directly attached to the bicyclic core (e.g., **1b**–**c**), while alterations of substituents, spacer lengths and aromatic groups connected to the urea were more restricted. Of note, care must be exercised when interpreting these data as several of the analogs display poor solubility (vide infra), which is also visible when running the enzymatic assay, such that the observed SAR is not simply governed by the activity towards IRAP but also on compound bioavailability in the test system. The emerging preliminary SAR has shown that removal of the methyl group decreased inhibitory potency/solubility (**1d**–**e**), while elongation of the spacer between the urea and the phenyl ring regained some activity (**1g**–**h**). In addition, the substitution pattern on the phenyl ring directly connected to the bicyclic core had a significant impact on the observed inhibition (**1h**–**l**). Both the exchange of the phenyl ring for thiophene (**1m**–**n**) and the shortening of the spacer between the urea and the phenyl ring (**1o**) decreased inhibitory capacity. Moreover, substitutions to cyclohexyl (**1p**), isopropyl (**1q)**, allyl (**1r**) or ester (**1s**) were detrimental to observed inhibition. Expansion to bicyclic moieties connected to the urea afforded inactive or poorly active inhibitors (**1t**–**u**), and finally, substitution on the phenyl ring rendered inactive or poorly active inhibitors (**1v**–**w**).

**Figure 2:**
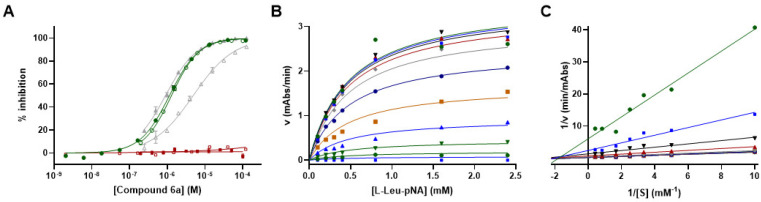
(**A**) Selectivity profiling of hit compound **6a** through concentration–response experiments on human IRAP and APN. Data are presented as the average and standard deviation of three technical replicates for each concentration in experiments based on membrane preparations with overexpressed IRAP and APN, respectively. The symbols represent two independent test occasions for compound **6a** on human IRAP (●,○) and aminopeptidase N (◼,◻). Also included are inhibition curves for human IRAP (▲) and APN (△) in the presence of a sulfonamide-based inhibitor that was used as a positive control (see compound **3** in ref [50]). Solid lines represent the best fits to a four-parameter inhibition curve within GraphPad Prism, with the average pIC_50_ for human IRAP at 5.9 ± 0.05. (**B**) Rates of enzymatic processing by IRAP as a function of L-Leu-*p*NA concentration in the presence of **6a** from 0 to 100 µM (2.5-fold dilutions between concentrations). Data are based on the average of four technical replicates in experiments conducted on IRAP in membrane preparations from CHO cells (for example, raw data at a low and a high substrate concentration are provided in Supplementary Materials Figure S1). The solid lines represent the best fit to a model of non-competitive inhibition, resulting in a K_m_ value of 0.48 ± 0.03 mM, a V_max_ of 3.6 ± 0.07 mAbs/min and a pK_i_ value of 5.64 ± 0.03. (**C**) Data from Figure 2A presented as a double-reciprocal Lineweaver Burk plot, demonstrating the characteristic behavior of non-competitive inhibitors with K_m_ unaffected and with V_max_ gradually reduced in the presence of increasing concentrations of inhibitor.

Given these initial observations, we set out to better define the underlying mechanism of inhibition and to explore the SAR further through directed synthetic efforts. To facilitate these efforts, we hypothesized the tentative binding pose of these compounds in the active site of IRAP, with the urea carbonyl interacting with the catalytically important zinc (see Supplementary Materials Figure S1). The preliminary pose broadly explained the observed activities and was thus used to guide early chemistry efforts, although it was later challenged by experimental evidence demonstrating full selectivity versus the closely related aminopeptidase N (APN). While **6a** (resynthesized hit compound, Table 2) confirmed similar inhibitory potency on human IRAP [49], it had no impact on the activity of human APN at concentrations up to 125 µM (Figure 2A). As the active sites of these enzymes are homologous, a similar binding pose can also be envisioned for APN (Supplementary Material Figure S1). Hence, such binding runs counter to the observed selectivity unless more complex models are evoked, e.g., that the equilibrium between the open and closed forms of these enzymes [10] differ significantly, such that open-form binding is significantly favored for IRAP over APN.

Given this anomaly, we also explored how the inhibitory activities varied with substrate concentration, aiming to understand whether a representative compound acts as a competitive binder in the active site. We recently demonstrated that one of the other hit compounds, a spiro-oxindole dihydroquinazolinone, displayed an uncompetitive inhibition pattern despite it occupying a portion of the active site about 4 Å away from the Zn^2+^ ion [53]. These studies were based on the same synthetic substrate that was employed for large-scale screening of IRAP, i.e., L-Leu-*para*-nitroanilide (L-Leu-*p*NA). Here, these experiments were reproduced for **6a** (see below for details). As shown in Figure 2B,C, inhibition was not associated with a progressive increase in apparent K_m_ value, as expected for a competitive inhibitor. Instead, V_max_ was shown to decrease with increasing compound concentration, clearly signifying non-competitive binding with the substrate, i.e., binding occurs with equal affinity to apo-enzyme and enzyme-substrate complex and with a measured IC_50_ value that is independent of substrate concentration (Supplementary Material Figure S2). It is, therefore, important to emphasize that we have previously demonstrated that **6a** binds reversibly to IRAP through jump-dilution experiments [49]. Although there are literature examples of non-competitive binding also for active site binders [54], these data, together with the observed selectivity, required us to consider an allosteric binding site alongside the active site model [55]. To enable structure-guided compound optimization and to firmly establish the inhibitor binding site, we have initiated co-crystallization efforts with the recombinant human protein [10]. Interestingly, the observation of non-competitive binding contrasts with the known peptide-based inhibitors and benzopyranes, which are known to act competitively with the substrate, and with the above-mentioned uncompetitive inhibitors.

**Scheme 1:**
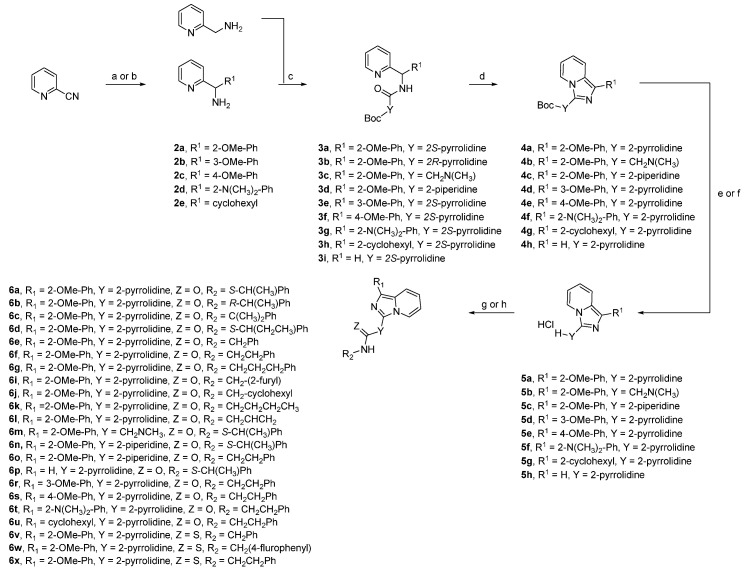
Reagents and conditions: (a) i. Grignard reagent, toluene, 1h, ii. NaBH_4_, 14 h, 67–95%; (b) i. aryl or alkyl bromide, Mg, I_2_, THF, MW, 100 °C, 1 h [56], ii. nitrile, rt., 2 h, iii. NaBH_4_, 14 h, 44–90%; (c) Boc-protected amino acid, EDC, HOBt, TEA, DCM, rt., 3 h, 70–95%; (d) POCl_3_, pyridine, DCM, 0 °C to rt., 14 h, 70–85%; (e) HCl in dioxane, rt., 14 h, 99%; (f) acetyl chloride, MeOH, DCM, 0 °C, 2 h, 94–99%; (g) isocyanate or isothiocyanate, TEA, MeCN, rt., 20 min, 48–99%; (h) i. amine, triphosgene, TEA, DCM, 0 °C, 1 h, ii. **5a**, TEA, DCM, rt., 30 min, 61–94%.

**Scheme 2:**
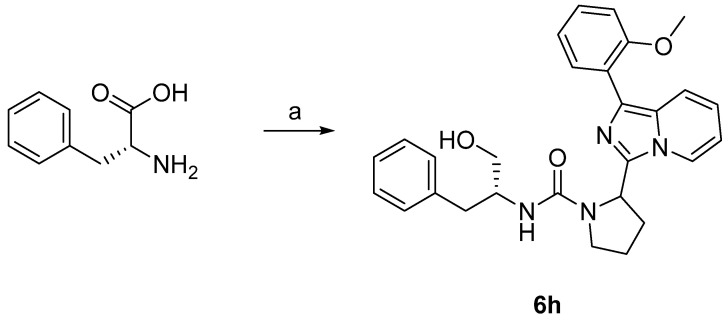
Reagents and conditions: (a) i. SOCl_2_, MeOH, rt., ii. Triphosgene, TEA, DCM, 0 °C, 1 h, iii. **5a**, TEA, rt., iv. LiAlH_4_, THF, 1 h, 69%.

In parallel to the mechanistic studies, we initiated structure-activity relationship investigations. Compounds **6a**–**x** were synthesized according to Scheme 1 and Scheme 2. Furthermore, our endeavors encompassed the resynthesis of several active library compounds (**1a**, **1h**–**j**) to validate the precision of the screening results. The synthetic route employed a Grignard reaction [56], followed by the reduction of the formed imine to obtain benzylamines **2a**–**e**. Pyridine-2-ylmethanamine or **2a**–**e** were subsequently coupled with the appropriate amino acid to obtain **3a**–**i**. POCl_3_-mediated ring closure formed the bicyclic core structure **4a**–**h**. Unfortunately, this reaction step caused racemization of the C2 position in the pyrrolidine ring. Thereafter, the amine was Boc-deprotected (**5a**–**h**) and reacted with the appropriate isocyanate or isothiocyanate to obtain the final products **6a**–**d, f**–**s, u**–**x**. Compound **6e** was synthesized according to Scheme 2, where the appropriate amino acid was esterified, followed by a reaction with triphosgene to form the isocyanate, which then reacted with **5a**. Compound **6q** was synthesized from pyrrolidine and (*S*)-(-)-alpha-methylbenzyl isocyanate according to step g in Scheme 1.

The results from the evaluation of compounds **6a**–**x** as IRAP inhibitors are presented in Table 2. As mentioned above, we attempted to synthesize all the analogs with the specific stereochemistry from the amino acids intact, but unfortunately, the ring closure reaction (Scheme 1, step d) caused racemization, and the analogs had to be tested as mixtures of diastereomers or enantiomers. Initially, we resynthesized the hit compound (**6a**) and the analog with the opposite stereochemistry of the methyl group (**6b**). Compound **6b**, with the *R*-configuration, showed a 20-fold drop in activity compared to **6a**. Thereafter, we chose to separate the two diastereomers of **6a** using supercritical fluid chromatography (SFC) and evaluate their inhibitory capacity separately. This resulted in the best diastereomer **6a_dia2** displaying an IC_50_ value of 1.0 µM, which is 15-fold more potent than the less active diastereomer **6a_dia1** (Table 2). Regarding lipophilicity and metabolic stability, no difference was observed between the two diastereomers. They both displayed some aqueous solubility but, unfortunately, poor metabolic stability in both human liver microsomes and rat hepatocytes. However, the compounds displayed good stability in plasma and relatively large unbound fractions (Table 3). A dimethylated analog (**6c**) resulted in a drop of activity compared to **6a**, while elongation to an ethyl group (**6d**) furnished a compound with similar activity as **6a**. Removal of the methyl group (**6e**) gave a much less potent compound than **6a** and, compared to the methylated analogs, much poorer solubility (Table 3). Evaluation of these compounds did indicate that the stereochemistry of the methyl group is important for inhibition, with the *S*-configuration as the preferred one. Either because the methyl group makes up an important interaction point itself or because it directs the phenyl group in a more favorable orientation for binding. Elongation of the linker between the urea and the phenyl group with one additional carbon (**6f**) resulted in a compound less active than **6a** but more potent than **6e**. However, further elongation of the linker (**6g**) rendered a further drop in activity. The introduction of a polar sidechain to **6f** as in **6h** also resulted in the loss of inhibitory activity. The introduction of other kinds of substituents to the urea tail, such as a furyl (**6i**), cyclohexyl (**6j**), pentyl (**6k**), or an allyl group (**6l**), did not render any improvement in potency.

Thereafter, we started to investigate the central part of the molecule and whether the removal of the pyrrolidine ring to an open chain linker would be beneficial for inhibition (**6m**). The results demonstrated that the five-membered ring is preferred to retain activity, as **6m** caused a dramatic loss of potency compared to **6a**. However, no difference was observed regarding solubility and metabolic stability (Table 3). The exchange of the five-membered ring to a six-membered ring (**6n**–**o**) also resulted in a drop in activity.

Next, we looked at the bicyclic core and the aromatic ring connected to this moiety. Removal of the anisole part, as in **6p**, resulted in a less active compound, and additional removal of the bicyclic core, as in **6q**, furnished an inactive compound. Hence, this part of the molecule seems to be critical for retaining inhibitory capacity. Thereafter, we shifted the methoxy substituent from the ortho to the meta (**6r**) and para positions (**6s**), but neither gave any improvement in potency. Exchange of the para-anisole to a para-dimethylaniline (**6t**) also resulted in loss of activity. Substitution of the anisole to a saturated cyclohexyl (**6u**) provided a less potent compound as well.

To investigate if the urea participates in a hydrogen bond interaction, we wanted to study how switching from urea to thiourea would affect the inhibitory activity. Thioureas are weaker hydrogen bond acceptors than ureas [57,58], and hence, a potential loss of hydrogen bond interaction would cause a drop in potency. All three thioureas (**6v**–**x**) were much less active than the corresponding ureas, and hence, one can hypothesize that the carbonyl oxygen participates in a particular hydrogen bond interaction. The solubility also decreased considerably (Table 3).

The screening campaign and the following SAR investigations were performed on CHO cells, known to be an abundant source of IRAP activity [59]. To further study if the identified compounds can be used as starting points for the development of new IRAP inhibitors, we tested **6a**–**b** on the human orthologue of IRAP (hIRAP) overexpressed in HEK293 suspension cells as well as the recombinant soluble enzyme (sIRAP). A comparison of the amino acid sequences using the Clustal Omega web tool at EMBL-EBI (default parameters) for hamster and human IRAP shows 88.4% sequence similarity [60]. As previously mentioned, we also evaluated them on human APN [61], a closely related enzyme also belonging to the M1 aminopeptidase family, to gain information on selectivity. As displayed in Figure 2A and Table 4, both **6a** and **6b** proved to inhibit human IRAP with similar potencies as obtained on CHO-cell IRAP. With regards to selectivity towards APN, neither compound showed any inhibitory activity on this enzyme.

**Figure 3:**
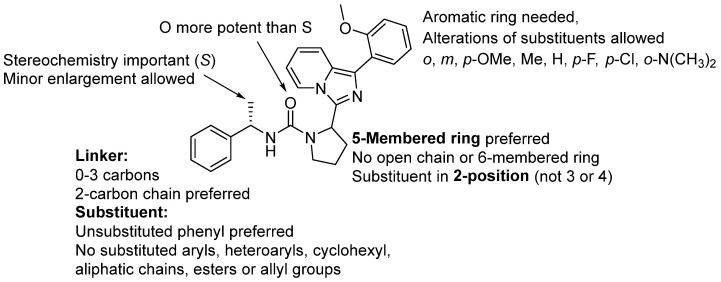
Summary of SAR exploration.

In summary, the stereochemistry of the methyl group on the urea tail seems important for inhibition, with the *S*-configuration being preferred (Figure 3). Two interpretations for this preference may be that the methyl group itself is an important interaction point or because it directs the phenyl group into a more favorable orientation for binding. Minor enlargement, for example, to an ethyl group, was allowed, but bigger groups were not tolerated. Regarding the spacer length between the urea and the phenyl ring, a two-carbon linker appears to be the most preferred length of the variants evaluated so far. Elongation to three carbons reduced potency, like observations when shortening to one carbon. Having the aryl group directly connected to the urea furnished inactive compounds or compounds with low potency. Furthermore, an unsubstituted phenyl ring was preferred to substituted rings of various kinds, heteroaryls, cyclohexyls, lipophilic chains, esters or allyl groups. The synthesized thioureas were less active than the corresponding ureas. Thioureas are weaker hydrogen bond acceptors than ureas; hence, the drop-in potency could be due to a potential loss of hydrogen bond interaction with the carbonyl oxygen [58]. A central five-membered ring is preferred in comparison to a one-carbon open chain or a six-membered ring with the nitrogen in 2, 3 or 4-position. Alterations of the substituents on the topmost anisole-ring (*o*, *m*, *p*-OMe, Me, H, *p*-F, *p*-Cl, *o*-N(CH_3_)_2_) are allowed without significantly affecting the activity. Neither did switching from an anisole to a cyclohexyl ring. However, the removal of the entire moiety resulted in a drop in activity, indicating that this part significantly contributes to the inhibitory capacity.

## 3. Materials and Methods

All chemicals were purchased from Sigma Aldrich/Acros (Burlington, MA, USA) and were used as received. Purification was performed on column chromatography using silica gel (60–120 mesh size) and for test compounds also reversed-phase high-performance liquid chromatography (RP-HPLC) (UV-triggered (254 nm) fraction collection with a Dionex (Sunnyvale, CA, USA) UltiMate 3000 HPLC system, using a Macherey–Nagel (Düren, Germany) Nucleodur C18 column (21 × 125 mm, 5 μm particle size), and a gradient of H_2_O/CH_3_CN/0.1% CF_3_COOH as eluent (10 mLmin^−1^ over 15–20 min). Analytical HPLC-mass spectrometry (HPLC-MS) was performed on a Dionex UltiMate 3000 HPLC system with a Bruker (Billerica, MA, USA) amazon SL ion trap mass spectrometer and detection by UV (diode array detector) and MS (electrospray ionization, ESI− or ESI+), using a Phenomenex (Torrance, CA, USA) Kinetex C18 column (50 × 3.0 mm, 2.6 μm particle size, 100 Å pore size) and a flow rate of 1.5 mLmin^−1^. A gradient of H_2_O/CH_3_CN/0.05% HCOOH was used. High-resolution mass spectra (HRMS) were recorded on a Micromass (Wilmslow, UK) Q-Tof2 mass spectrometer equipped with an electrospray ion source. ^1^H and ^13^C NMR spectra were recorded at room temperature on a Varian (Palo Alto, CA, USA) 400-MR spectrometer at 400 MHz and 100 MHz, respectively. Chemical shifts are reported in ppm with the solvent residual peak as the internal standard. For rotamers, NMR experiments were performed both at room temperature and at elevated temperature; see spectra in Supplementary Materials.

### 3.1. General Procedure Grignard Reaction

Method A: The Grignard reagent in THF (1.0 M, 1.2–1.5 equiv) was added to a solution of 2-cyanopyridine (1 equiv) in dry toluene or THF. The mixture was stirred for 1 h at ambient temperature. Isobutanol was added to a clear solution. Thereafter, NaBH_4_ (2–4 equiv) was added, and the mixture was stirred overnight. The reaction was quenched by the addition of a 1:1 mixture of MeOH and H_2_O, and the organic solvents were removed under reduced pressure. To the remaining aqueous mixture was added 1M NaOH (aq), which was subsequently extracted with DCM (×3). The combined organic phases were washed with brine, dried over Na_2_CO_3_ and concentrated. The crude products were purified by silica flash column chromatography using MeOH in DCM as eluent.

Method B: The aryl or alkyl bromide (2.25 mmol), Mg (219 mg, 9.0 mmol) and one crystal of I_2_ were added to a Smith vial (2–5 mL) and degassed and flushed with nitrogen. Dry THF (4 mL) was added, and the mixture was heated with MW irradiation for 1 h at 100 °C [62]. Thereafter, the solution was transferred via syringe to a round-bottomed flask containing 2-cyanopyridine (144 µL, 1.5 mmol) in dry THF (1 mL). The mixture was stirred at ambient temperature for 2 h. Isobutanol (2 mL) was added to a clear solution. NaBH_4_ (113 mg, 3.0 mmol) was added, and the mixture was stirred overnight. The reaction was quenched by the addition of a 1:1 mixture of MeOH and H_2_O, and the organic solvents were removed under reduced pressure. To the remaining aqueous mixture was added 20 mL 1M NaOH (aq), which was subsequently extracted with DCM (×3). The combined organic phases were washed with brine, dried over Na_2_CO_3_, filtered and concentrated. The crude product was purified by silica flash column chromatography using MeOH in DCM as eluent to afford the product.

#### 3.1.1. (2-Methoxyphenyl)(pyridine-2-yl)methanamine (**2a**)

Method A. 2-cyanopyridine (0.5 mL, 5.2 mmol), toluene (25 mL), 2-methoxymagnesiumbromide in THF (1M, 6.2 mL, 6.2 mmol) and NaBH_4_ (393 mg, 10.4 mmol). Yellow oil (1.06 g, 95%). ^1^H NMR (400 MHz, Methanol-d_4_) *δ* 8.48 (ddd, *J* = 5.1, 1.8, 1.0 Hz, 1H), 7.72 (ddd, *J* = 7.7, 6.8, 1.8 Hz, 1H), 7.35 (ddd, *J* = 7.7, 1.3, 1.0 Hz, 1H), 7.27–7.21 (m, 3H), 6.98–6.90 (m, 2H), 5.45 (s, 1H), 4.85 (s, 2H), 3.78 (s, 3H). ^13^C NMR (101 MHz, CDCl_3_) *δ* 163.4, 156.8, 149.1, 136.3, 133.4, 128.2, 127.8, 121.7, 121.7, 121.0, 110.7, 55.5, 55.0.

#### 3.1.2. (3-Methoxyphenyl)(pyridin-2-yl)methanamine (**2b**)

Method B. 3-bromoanisole (285 µL). Yellow oil (288 mg, 90%). ^1^H NMR (400 MHz, Methanol-d_4_) *δ* 8.49 (dd, *J* = 4.8, 0.8 Hz, 1H), 7.77–7.71 (m, 1H), 7.43 (dd, *J* = 7.8, 0.6 Hz, 1H), 7.25 (ddd, *J* = 7.6, 4.8, 0.6 Hz, 1H), 7.21 (ddd, *J* = 8.2, 7.8, 0.4 Hz, 1H), 6.98–6.96 (m, 1H), 6.93 (ddd, *J* = 7.8, 1.8, 1.0 Hz, 1H), 6.78 (ddd, *J* = 8.2, 2.6, 1.0 Hz, 1H), 5.15 (s, 1H), 4.86 (s, 3H), 3.75 (s, 3H). ^13^C NMR (101 MHz, MeOD) *δ* 164.3, 161.4, 149.6, 146.8, 138.6, 130.6, 123.6, 123.1, 120.3, 113.8, 113.7, 61.7, 55.6.

#### 3.1.3. (4-Methoxyphenyl)(pyridin-2-yl)methanamine (**2c**)

Method B. 4-bromoanisole (282 µL). Yellow oil (269 mg, 84%). ^1^H NMR (400 MHz, Methanol-d_4_) *δ* 8.48 (ddd, *J* = 5.0, 1.9, 0.9 Hz, 1H), 7.74 (ddd, *J* = 7.7, 6.0, 1.9 Hz, 1H), 7.43–7.38 (m, 1H), 7.27 (dd, *J* = 8.8, 2.2 Hz, 2H), 7.24 (ddd, *J* = 7.7, 5.0, 1.1 Hz, 1H), 6.86 (dd, *J* = 8.8, 2.2 Hz, 2H), 5.13 (s, 1H), 4.85 (s, 3H), 3.74 (s, 3H). ^13^C NMR (101 MHz, CD_3_OD) *δ* 164.7, 160.4, 149.6, 138.6, 137.3, 129.2, 123.5, 123.0, 115.0, 61.1, 55.7.

#### 3.1.4. 2-(Amino(pyridin-2-yl)methyl)-*N*,*N*-dimethylaniline (**2d**)

Method B. 2-bromo-*N*,*N*-dimethylaniline (326 µL). Yellow oil (149 mg, 44%). ^1^H NMR (400 MHz, Methanol-d_4_) *δ* 8.51 (ddd, *J* = 4.9, 1.9, 1.0 Hz, 1H), 7.71 (ddd, *J* = 7.9, 7.7, 1.9 Hz, 1H), 7.32 (ddd, *J* = 7.9, 1.0, 0.3 Hz, 1H), 7.29–7.24 (m, 3H), 7.24–7.21 (m, 1H), 7.09 (ddd, *J* = 8.5, 6.8, 1.9 Hz, 1H), 5.73 (s, 1H), 4.87 (s, 2H), 2.61 (s, 6H). ^13^C NMR (101 MHz, MeOD) *δ* 164.4, 153.9, 149.5, 140.7, 138.2, 129.4, 129.1, 125.9, 123.4, 123.3, 122.5, 56.2, 46.2.

#### 3.1.5. Cyclohexyl(pyridin-2-yl)methanamine (**2e**)

Method A. 2-cyanopyridine (144 µL, 1.5 mmol), THF (5 mL), cyclohexylmagnesiumchloride in THF (1M, 2.25 mL, 2.25 mmol) and NaBH_4_ (227 mg, 6.0 mmol). Yellow oil (190 mg, 67%). ^1^H NMR (400 MHz, Methanol-d_4_) *δ* 8.49 (ddd, *J* = 5.0, 1.6, 0.9 Hz, 1H), 7.78 (dd, *J* = 7.6, 1.6 Hz, 1H), 7.38 (dd, *J* = 7.9, 1.1, 0.9 Hz, 1H), 7.28 (ddd, *J* = 7.6, 5.0, 1.1 Hz, 1H), 4.85 (s, 3H), 3.66 (d, *J* = 7.3 Hz, 1H), 1.96–1.87 (m, 1H), 1.83–1.74 (m, 1H), 1.71–1.60 (m, 3H), 1.36–1.11 (m, 4H), 1.10–0.91 (m, 2H). ^13^C NMR (101 MHz, MeOD) *δ* 164.4, 149.5, 138.3, 123.7, 123.6, 63.2, 46.0, 31.1, 30.2, 27.5, 27.3.

### 3.2. General Procedure for Peptide Coupling

To a solution of the appropriate amine (1 equiv), the appropriate amino acid (1.2 equiv), HOBt (1.2 equiv) and TEA (2.0 equiv) in dry DCM under an inert atmosphere was added EDC×Cl (1.2 equiv) and the reaction mixture was stirred at room temperature for 3 h. The reaction mixture was diluted with more DCM, washed with water and brine, dried over MgSO_4_, filtered, and concentrated under reduced pressure. The crude products were purified by silica flash column chromatography using MeOH in DCM.

#### 3.2.1. (2*S*)-*Tert*-butyl-2-(((2-methoxyphenyl)(pyridine-2-yl)methyl)carbamoyl)pyrrolidine-1-carboxylate (**3a**)

**2a** (300 mg, 1.4 mmol), *N*-Boc-L-Proline (362 mg, 1.7 mmol), HOBt (257 mg, 1.7 mmol), TEA (0.4 mL, 2.9 mmol), EDC*HCl (322 mg, 1.7 mmol) and DCM (14 mL). White semi-solid mixture of diastereomers (1:1) and rotamers (542 mg, 94%). ^1^H NMR (400 MHz, DMSO-d6) *δ* 8.59 (d, *J* = 8.3 Hz, 1H, 1 isomer) 8.52 (d, *J* = 8.3 Hz, 1H, 1 isomer), 8.49–8.44 (m, 1H + 1H, 2 isomers), 7.77–7.68 (m, 1H + 1H, 2 isomers), 7.47–7.18 (m, 4H + 4H, 2 isomers), 6.98 (dd, *J* = 2.2, 1.1 Hz, 1H, 1 isomers), 6.95 (dd, *J* = 2.2, 1.1 Hz, 1H, 1 isomers), 6.94–6.86 (m, 1H + 1H, 2 isomers), 6.45 (t, *J* = 8.9 Hz, 1H, 1 isomer), 6.37 (t, *J* = 7.2 Hz, 1H, 1 isomer), 4.35–4.21 (m, 1H + 1H, 2 isomers), 3.75 (d, *J* = 2.3 Hz, 3H + 3H, 2 isomers), 3.34 (m, 3H + 3H, 2 isomers), 2.19–1.96 (m, 1H + 1H, 2 isomers), 1.90–1.68 (m, 3H + 3H, 2 isomers), 1.47–1.16 (m, 9H + 9H, 2 isomers). ^13^C NMR (101 MHz, DMSO-d6). Isomer 1: 171.51 (major rotamer), 171.1 (minor rotamer), 160.6, 156.3, 153.8, 148.8, 136.64, 129.7, 128.4, 128.2, 122.13, 121.8 (major rotamer), 121.6 (minor rotamer), 120.2, 111.0, 78.8 (minor rotamer), 78.42 (major rotamer), 59.6 (major rotamer), 59.4 (minor rotamer), 55.43, 52.1 (minor rotamer), 51.85 (major rotamer), 46.7, 31.0, 28.1, 23.9. Isomer 2: 171.46 (major rotamer), 171.0 (minor rotamer), 160.2, 156.3, 153.4, 148.8, 136.55, 129.5, 128.3, 127.5, 122.08, 121.42 (minor rotamer), 121.25 (major rotamer), 120.2, 111.0, 78.7 (minor rotamer), 78.36 (major rotamer), 59.5 (major rotamer), 59.3 (minor rotamer), 55.4, 51.90 (minor rotamer), 51.7 (major rotamer), 46.5, 29.5, 27.8, 23.1.

#### 3.2.2. *Tert*-butyl-(2-(((2-methoxyphenyl)(pyridin-2-yl)methyl)amino)-2-oxoethyl)(methyl)carbamate (**3c**)

**2a** (300 mg, 1.4 mmol), *N*-Boc-Sarcosine (318 mg, 1.7 mmol), HOBt (257 mg, 1.7 mmol), TEA (0.4 mL, 2.9 mmol), EDC*HCl (322 mg, 1.7 mmol) and DCM (14 mL). White amorphous solid (481 mg, 89%). ^1^H NMR (400 MHz, CDCl_3,_ 50 °C) *δ* 8.46 (ddd, *J* = 4.9, 1.8, 0.9 Hz, 1H), 8.00 (d, *J* = 7.8 Hz, 1H), 7.52 (td, *J* = 7.7, 1.8 Hz, 1H), 7.33–7.26 (m, 2H), 7.18 (ddd, *J* = 8.2, 7.4, 1.7 Hz, 1H), 7.06 (ddd, *J* = 7.5, 4.9, 1.2 Hz, 1H), 6.88 (td, *J* = 7.5, 1.1 Hz, 1H), 6.83 (dd, *J* = 8.2, 1.1 Hz, 1H), 6.50 (d, *J* = 7.7 Hz, 1H), 3.98–3.87 (m, 2H), 3.79 (s, 3H), 2.96 (s, 3H), 1.45 (s, 9H). ^13^C NMR (101 MHz, CDCl_3,_ 50 °C) *δ* 168.3, 159.6, 156.8, 148.7, 136.5, 130.1, 128.7, 128.2, 122.0, 121.8, 121.0, 111.5, 80.5, 55.7, 52.7, 35.7, 28.4.

#### 3.2.3. *Tert*-butyl-2-(((2-methoxyphenyl)(pyridin-2-yl)methyl)carbamoyl)piperidine-1-carboxylate (**3d**)

**2a** (321 mg, 1.5 mmol), *N*-Boc-piperidine-2-carboxylic acid (412 mg, 1.8 mmol), HOBt (275 mg, 1.8 mmol), TEA (1.04 mL, 7.5 mmol) and EDC×HCl (345 mg, 1.8 mmol). Yellow oil (449 mg, 70%). ^1^H NMR (400 MHz, DMSO-d_6_, 90 °C) *δ* 8.47 (ddq, *J* = 4.0, 2.1, 1.0 Hz, 1H), 8.09 (d, *J* = 8.2 Hz, 1H), 7.70 (td, *J* = 7.6, 1.7 Hz, 1H), 7.32 (d, *J* = 7.6 Hz, 1H), 7.23 (ddd, *J* = 8.9, 6.9, 3.5 Hz, 3H), 6.99 (dd, *J* = 8.6, 1.2 Hz, 1H), 6.90 (td, *J* = 7.4, 1.1 Hz, 1H), 6.40 (d, *J* = 8.0 Hz, 1H), 4.66 (dd, *J* = 6.3, 2.5 Hz, 1H), 3.87 (dt, *J* = 13.3, 3.4 Hz, 1H), 3.77 (d, *J* = 0.8 Hz, 3H), 3.07 (td, *J* = 12.6, 3.4 Hz, 1H), 2.09 (dt, *J* = 13.4, 2.8 Hz, 1H), 1.64–1.50 (m, 3H), 1.39 (s, 9H), 1.37–1.29 (m, 2H). ^13^C NMR (101 MHz, DMSO-d_6_, 90 °C) *δ* 169.7, 159.6, 156.3, 154.5, 148.1, 136.0, 129.6, 127.9, 127.4, 121.5, 121.0, 120.0, 111.3, 78.7, 55.3, 53.9, 52.1, 40.9, 27.6, 26.0, 23.8, 19.2.

#### 3.2.4. *Tert*-butyl-2-(((3-methoxyphenyl)(pyridin-2-yl)methyl)carbamoyl)pyrrolidine-1-carboxylate (**3e**)

**2b** (280 mg, 1.3 mmol), *N*-Boc-L-Prolin (338 mg, 1.6 mmol), HOBt (240 mg, 1.6 mmol), TEA (364 µL, 2.6 mmol), EDC*HCl (301 mg, 1.6 mmol) and DCM (13 mL). Clear oil (401 mg, 75%). ^1^H NMR (400 MHz, DMSO-d_6_, 65 °C) δ 8.52 (ddd, *J* = 4.8, 1.8, 1.0 Hz, 1H + 1H, 2 isomers), 8.36 (t, *J* = 7.3 Hz, 1H + 1H, 2 isomers), 7.75 (tdd, *J* = 7.7, 2.9, 1.8 Hz, 1H + 1H, 2 isomers), 7.48 (dt, *J* = 7.8, 1.1 Hz, 1H, 1 isomer), 7.44 (dt, *J* = 7.9, 1.2 Hz, 1H, 1 isomer), 7.26 (ddd, *J* = 7.5, 4.8, 1.2 Hz, 1H + 1H, 2 isomers), 7.19 (td, *J* = 7.9, 3.1 Hz, 1H + 1H, 2 isomers), 6.93–6.85 (m, 2H + 2H, 2 isomers), 6.79 (dt, *J* = 8.2, 1.8 Hz, 1H + 1H, 2 isomers), 6.11 (dd, *J* = 8.1, 3.5 Hz, 1H + 1H, 2 isomers), 4.33–4.26 (m, 1H + 1H, 2 isomers), 3.73 (s, 3H + 3H. 2 isomers), 3.37 (dtt, *J* = 10.3, 7.8, 3.1 Hz, 2H + 2H, 2 isomers), 2.12 (dt, *J* = 15.1, 7.5 Hz, 1H + 1H. 2 isomers), 1.92–1.74 (m, 3H + 3H, 2 isomers), 1.32 (s, 9H + 9H, 2 isomers). ^13^C NMR (101 MHz, DMSO-d6, 65 °C) δ Isomer 1: 170.9, 159.5, 159.0, 148.28, 142.9, 136.25, 128.66, 121.78, 121.3, 118.9, 112.8, 111.9, 78.3, 59.4, 56.87, 54.7, 46.2, 27.6. Isomer 2: 170.9, 159.6, 159.0, 148.31, 143.1, 136.30, 128.70, 121.79, 121.5, 119.1, 112.9, 112.0, 78.3, 59.4, 56.91, 54.7, 46.2, 27.6.

#### 3.2.5. *Tert*-butyl-2-((cyclohexyl(pyridin-2-yl)methyl)carbamoyl)pyrrolidine-1-carboxylate (**3h**)

**2e** (190 mg, 1.0 mmol), *N*-Boc-L-Prolin (258 mg, 1.2 mmol), HOBt (183 mg, 1.2 mmol), TEA (278 µL, 2.0 mmol), EDC×HCl (230 mg, 1.2 mmol) and DCM (10 mL). Clear oil (375 mg, 97%). ^1^H NMR (400 MHz, DMSO-d_6_, 90 °C) *δ* 8.51 (ddd, *J* = 4.8, 2.0, 1.0 Hz, 1H + 1H, 2 isomers), 7.70 (tdd, *J* = 7.7, 4.0, 1.8 Hz, 1H + 1H, 2 isomers), 7.63 (t, *J* = 7.9 Hz, 1H + 1H, 2 isomers), 7.32 (dt, *J* = 7.8, 1.1 Hz, 1H), 7.29 (dd, *J* = 7.8, 1.1 Hz, 1H), 7.22 (ddt, *J* = 7.7, 4.8, 1.5 Hz, 1H + 1H, 2 isomers), 4.77 (ddd, *J* = 8.8, 7.5, 4.3 Hz, 1H + 1H, 2 isomers), 4.22 (d, *J* = 2.8 Hz, 1H), 4.21 (d, *J* = 3.4 Hz, 1H), 3.42–3.29 (m, 2H + 2H, 2 isomers), 2.16–2.01 (m, 1H + 1H, 2 isomers), 1.90–1.54 (m, 17H, 2 isomers), 1.35 (s, 9H), 1.27 (s, 9H), 1.20–0.90 (m, 11H, 2 isomers). ^13^C NMR (101 MHz, DMSO-d_6_, 90 °C, mixture of 2 diastereomers) δ 171.0, 170.9, 159.98, 159.95, 148.20, 148.17, 135.63, 135.58, 121.7, 121.5, 121.4, 78.2, 78.1, 59.49, 59.46, 57.9, 57.8, 46.1, 41.7, 41.6, 29.10, 29.08, 28.0, 27.9, 27.6, 27.5, 25.4, 25.1, 25.0, 22.9.

#### 3.2.6. (*S*)-*Tert*-butyl-2-((pyridin-2-ylmethyl)carbamoyl)pyrrolidine-1-carboxylate (**3i**)

2-(aminomethyl)pyridine (0.5 mL, 4.85 mmol), *N*-Boc-L-proline (1.57 g, 7.3 mmol), HOBt (1.11 g, 7.3 mmol), TEA (1.7 mL, 12 mmol) and EDC×HCl (1.39 g, 7.3 mmol). White powder (1.4 g, 95%). [α]^D^_24_ −80° (c = 1.63, CHCl3). ^1^H NMR (400 MHz, DMSO-d_6_) δ 8.53–8.40 (m, 2H), 7.78–7.67 (m, 1H), 7.36–7.21 (m, 2H), 4.41 (dd, *J* = 15.9, 6.2 Hz, 1H), 4.30 (dd, *J* = 15.9, 5.8 Hz, 1H), 4.19–4.09 (m, 1H), 3.46–3.35 (m, 1H), 3.32–3.25 (m, 1H), 2.49–2.04 (m, 1H), 1.91–1.71 (m, 3H), 1.35 (d, 9H, signal from two rotamers). ^13^C NMR (101 MHz, DMSO-d_6_) δ 172.7 (major rotamer), 172.5 (minor rotamer), 158.8 (minor rotamer), 158.7 (major rotamer), 153.8 (minor rotamer), 153.4 (major rotamer), 148.8 (major rotamer), 148.7 (minor rotamer), 136.52, 122.1 (major rotamer), 121.9 (minor rotamer), 121.0 (major rotamer), 120.5 (minor rotamer), 78.7 (minor rotamer), 78.5 (major rotamer), 59.9 (major rotamer), 59.8 (minor rotamer), 46.7 (minor rotamer), 46.5 (major rotamer), 44.2 (major rotamer), 44.0 (minor rotamer), 31.0 (major rotamer), 30.0 (minor rotamer), 28.1 (minor rotamer), 28.0 (major rotamer), 24.0 (minor rotamer), 23.2 (major rotamer).

### 3.3. General Procedure Ring Closure Reaction

A solution of the appropriate amide (1 equiv) and pyridine (6 equiv) in dry DCM under an inert atmosphere was cooled to 0 °C in an ice bath, and POCl_3_ (1.8 equiv) was added dropwise. The reaction mixture was stirred at room temperature for 14 h. The mixture was washed with NaOH (1%, aq) and brine, dried over MgSO_4_ and concentrated under reduced pressure. Purification by flash column chromatography using 40% EtOAc in pentane as eluent gave the desired compounds.

#### 3.3.1. *Tert*-butyl-2-(1-(2-methoxyphenyl)imidazo [1,5-a]pyridin-3-yl)pyrrolidine-1-carboxylate (**4a**)

**3a** (300 mg, 0.73 mmol), pyridine (0.35 mL, 4.37 mmol), POCl_3_ (0.12 mL, 1.31 mmol) and DCM (5 mL). Green semi-solid (240 mg, 84%). ^1^H NMR (400 MHz, DMSO-d_6_, 100 °C) *δ* 8.26 (dd, *J* = 7.3, 1.0 Hz, 1H), 7.60 (dd, *J* = 7.6, 1.8 Hz, 1H), 7.47 (dd, *J* = 9.2, 1.3 Hz, 1H), 7.29 (ddd, *J* = 8.3, 7.4, 1.8 Hz, 1H), 7.10 (dd, *J* = 8.3, 1.1 Hz, 1H), 7.02 (ddd, *J* = 7.6, 7.4, 1.1 Hz, 1H), 6.73 (ddd, *J* = 9.2, 6.3, 1.0 Hz, 1H), 6.64 (ddd, *J* = 7.4, 6.3, 1.3 Hz, 1H), 5.35 (dd, *J* = 7.4, 4.6 Hz, 1H), 3.80 (s, 3H), 3.62–3.49 (m, 2H), 2.38–2.21 (m, 2H), 2.19–2.11 (m, 1H), 2.01–1.90 (m, 1H), 1.18 (s, 9H). ^13^C NMR (101 MHz, DMSO-d_6_, 100 °C) *δ* 156.7, 154.1, 140.0, 131.4, 131.2, 127.4, 127.2, 125.0, 122.4, 120.4, 120.1, 118.2, 112.3, 78.9, 56.1, 55.9, 53.1, 46.9, 28.5, 28.3, 24.1.

#### 3.3.2. *Tert*-butyl-((1-(2-methoxyphenyl)imidazo [1,5-a]pyridin-3-yl)methyl)(methyl)carbamate (**4b**)

**3c** (298 mg, 0.69 mmol), pyridine (130 µL, 1.6 mmol), POCl_3_ (97 µL, 1.1 mmol) and DCM (7 mL). Green semi-solid (287 mg, 83%). ^1^H NMR (400 MHz, DMSO-d_6_) *δ* 8.31–8.12 (m, 1H), 7.60 (dd, *J* = 7.6, 1.8 Hz, 1H), 7.50 (dt, *J* = 9.3, 1.2 Hz, 1H), 7.31 (ddd, *J* = 8.3, 7.3, 1.8 Hz, 1H), 7.12 (dd, *J* = 8.3, 1.1 Hz, 1H), 7.03 (td, *J* = 7.4, 1.1 Hz, 1H), 6.81 (ddd, *J* = 9.3, 6.3, 1.1 Hz, 1H), 6.77–6.72 (m, 1H), 4.83 (s, 2H), 3.81 (s, 3H), 2.80 (s, 3H), 1.42 (s, 9H). ^13^C NMR (101 MHz, DMSO-d_6_) δ 155.7, 134.7, 130.7, 128.0, 127.5, 126.6, 123.7, 122.0, 120.5, 120.0, 118.6, 112.4, 111.5, 79.4, 55.2, 43.2, 33.5, 28.0.

#### 3.3.3. *Tert*-butyl-2-(1-(2-methoxyphenyl)imidazo [1,5-a]pyridin-3-yl)piperidine-1-carboxylate (**4c**)

**3d** (410 mg, 0.96 mmol), pyridine (779 µL, 9.6 mmol), POCl_3_ (353 µL, 3.9 mmol) and DCM (10 mL). Green semi-solid (314 mg, 80%). ^1^H NMR (400 MHz, DMSO-d_6_) *δ* 8.15 (dt, *J* = 7.3, 1.2 Hz, 1H), 7.62 (dd, *J* = 7.6, 1.8 Hz, 1H), 7.51 (dt, *J* = 9.2, 1.2 Hz, 1H), 7.31 (ddd, *J* = 8.3, 7.3, 1.8 Hz, 1H), 7.12 (dd, *J* = 8.4, 1.1 Hz, 1H), 7.04 (td, *J* = 7.4, 1.1 Hz, 1H), 6.79 (ddd, *J* = 9.2, 6.3, 1.0 Hz, 1H), 6.71 (ddd, *J* = 7.3, 6.3, 1.3 Hz, 1H), 5.79–5.73 (m, 1H), 3.81 (s, 3H), 3.80–3.78 (m, 1H), 3.01–2.89 (m, 1H), 2.34–2.22 (m, 1H), 2.21–2.13 (m, 1H), 1.91–1.81 (m, 1H), 1.71–1.61 (m, 2H), 1.50–1.41 (m, 1H), 1.39 (s, 9H). ^13^C NMR (101 MHz, DMSO-d_6_) *δ* 155.7, 154.3, 137.5, 130.7, 127.9, 127.0, 126.1, 123.9, 121.8, 120.5, 120.1, 118.5, 112.3, 111.6, 79.3, 55.2, 40.9, 28.0, 27.5, 25.2, 19.8.

#### 3.3.4. *Tert*-butyl-2-(1-(3-methoxyphenyl)imidazo [1,5-a]pyridin-3-yl)pyrrolidine-1-carboxylate (**4d**)

**3e** (385 mg, 0.94 mmol), pyridine (605 µL, 7.5 mmol), POCl_3_ (214 µL, 2.3 mmol) and DCM (9 mL). Green semi-solid (257 mg, 70%). ^1^H NMR (400 MHz, DMSO-d_6_, 90 °C) *δ* 8.30 (dd, *J* = 7.2, 1.0 Hz, 1H), 7.83 (dt, *J* = 9.4, 1.2 Hz, 1H), 7.44 (dt, *J* = 7.7, 1.3 Hz, 1H), 7.39 (dd, *J* = 2.7, 1.5 Hz, 1H), 7.34 (t, *J* = 7.9 Hz, 1H), 6.88–6.80 (m, 2H), 6.69 (ddd, *J* = 7.2, 6.4, 1.2 Hz, 1H), 5.35 (dd, *J* = 7.4, 4.6 Hz, 1H), 3.84 (s, 3H), 3.66–3.50 (m, 2H), 2.39–2.22 (m, 2H), 2.17–2.10 (m, 1H), 2.01–1.92 (m, 1H), 1.16 (s, 9H). ^13^C NMR (101 MHz, DMSO-d_6_, 90 °C) *δ* 159.3, 153.1, 139.3, 136.1, 129.1, 128.3, 125.6, 122.0, 119.2, 118.0, 118.0, 111.7, 111.2, 78.0, 54.7, 52.2, 46.0, 31.2, 27.5, 23.1.

#### 3.3.5. *Tert*-butyl-2-(1-(4-methoxyphenyl)imidazo [1,5-a]pyridin-3-yl)pyrrolidine-1-carboxylate (4e)

**3f** (460 mg, 1.1 mmol), pyridine (542 µL, 6.7 mmol), POCl_3_ (184 µL, 2.0 mmol) and DCM (11 mL). Green semi-solid (361 mg, 82%). ^1^H NMR (400 MHz, DMSO-d_6_, 80 °C) *δ* 8.27 (dd, *J* = 7.2, 1.0 Hz, 1H), 7.78 (dd, *J* = 9.2, 1.2 Hz, 1H), 7.77 (dd, *J* = 8.9, 2.2 Hz, 2H), 7.01 (dd, *J* = 8.9, 2.1 Hz, 2H), 6.79 (ddd, *J* = 9.2, 6.3, 1.0 Hz, 1H), 6.65 (ddd, *J* = 7.2, 6.3, 1.2 Hz, 1H), 5.33 (dd, *J* = 7.3, 4.7 Hz, 1H), 3.80 (s, 3H), 3.63–3.49 (m, 2H), 2.37–2.21 (m, 2H), 2.16–2.08 (m, 1H), 2.00–1.90 (m, 1H), 1.14 (s, 9H). ^13^C NMR (101 MHz, DMSO-d_6_, 80 °C) *δ* 157.5, 153.2, 139.0, 128.6, 127.6, 126.7, 124.7, 121.8, 118.5, 117.8, 113.9, 111.6, 78.0, 54.8, 52.1, 46.0, 31.3, 27.5, 23.2.

#### 3.3.6. *Tert*-butyl-2-(1-(2-(dimethylamino)phenyl)imidazo [1,5-a]pyridin-3-yl)pyrrolidine-1-carboxylate (**4f**)

**3g** (186 mg, 0.45 mmol), pyridine (283 µL, 3.5 mmol), POCl_3_ (100 µL, 1.1 mmol) and DCM (5 mL). Green oil (361 mg, 83%). ^1^H NMR (400 MHz, Methanol-d_4_, 55 °C) *δ* 8.12 (dd, *J* = 7.8, 1.2, 1H), 7.45 (dd, *J* = 9.1, 1.5 Hz, 1H), 7.41 (dd, *J* = 7.6, 1.7 Hz, 1H), 7.25 (ddd, *J* = 8.1, 7.3, 1.7 Hz, 1H), 7.09 (dd, *J* = 8.2, 1.2 Hz, 1H), 6.98 (td, *J* = 7.4, 1.2 Hz, 1H), 6.70 (ddd, *J* = 9.1, 6.4, 1.2 Hz, 1H), 6.66 (ddd, *J* = 7.8, 6.4, 1.5 Hz, 1H), 5.35 (dd, *J* = 7.5, 5.9 Hz, 1H), 3.79–3.71 (m, 1H), 3.66–3.58 (m, 1H), 2.51 (s, 6H), 2.46–2.37 (m, 1H), 2.26–2.13 (m, 2H), 2.06–1.95 (m, 1H), 1.12 (s, 9H). ^13^C NMR (101 MHz, Methanol-d_4_, 55 °C) *δ* 156.0, 153.1, 140.8, 133.4, 131.2, 129.3, 128.2, 127.9, 122.4, 122.1, 121.2, 119.1, 118.7, 114.0, 81.0, 54.5, 47.9, 43.4, 34.2, 28.6, 25.0.

#### 3.3.7. *Tert*-butyl-2-(1-cyclohexylimidazo [1,5-a]pyridin-3-yl)pyrrolidine-1-carboxylate (**4g**)

**3h** (363 mg, 0.94 mmol), pyridine (758 µL, 9.4 mmol), POCl_3_ (343 µL, 3.8 mmol) and DCM (9 mL). Light green semi-solid (286 mg, 83%). ^1^H NMR (400 MHz, DMSO-d_6_, 90 °C) *δ* 8.10 (dd, *J* = 7.0, 1.2 Hz, 1H), 7.46 (dt, *J* = 9.1, 1.4 Hz, 1H), 6.55 (ddd, *J* = 9.1, 6.3, 1.2 Hz, 2H), 5.23 (dd, *J* = 7.3, 4.9 Hz, 1H), 3.58–3.45 (m, 2H), 2.90 (tt, *J* = 11.5, 3.5 Hz, 1H), 2.31–2.14 (m, 2H), 2.09–2.01 (m, 1H), 1.96–1.87 (m, 1H), 1.87–0.77 (m, 4H), 1.74–1.59 (m, 3H), 1.48–1.24 (m, 4H), 1.12 (s, 9H). ^13^C NMR (101 MHz, DMSO-d_6_, 90 °C) *δ* 153.1, 137.2, 135.3, 124.2, 121.0, 117.3, 115.4, 110.9, 77.8, 52.2, 46.0, 36.1, 32.5, 32.4, 31.3, 27.4, 25.7, 25.3, 23.1.

#### 3.3.8. *Tert*-butyl-2-(imidazo [1,5-a]pyridin-3-yl)pyrrolidine-1-carboxylate (**4h**)

**3i** (324 mg, 1.1 mmol), pyridine (515 µL, 6.4 mmol), POCl_3_ (194 µL, 2.1 mmol) and DCM (11 mL). Brown oil (252 mg, 83%). ^1^H NMR (400 MHz, DMSO-d_6_, 100 °C) *δ* 8.24 (dd, *J* = 7.3, 0.9 Hz, 1H), 7.47 (dt, *J* = 9.2, 1.3 Hz, 1H), 7.29 (s, 1H), 6.70 (ddd, *J* = 9.2, 6.3, 0.9 Hz, 1H), 6.60 (ddd, *J* = 7.3, 6.3, 1.3 Hz, 1H), 5.30 (dd, *J* = 7.5, 4.5 Hz, 1H), 3.56–3.48 (m, 2H), 2.32–2.16 (m, 3H), 2.10–2.02 (m, 1H), 1.97–1.87 (m, 2H), 1.16 (s, 9H). ^13^C NMR (101 MHz, DMSO-d_6_, 100 °C) *δ* 153.1, 139.3, 129.4, 121.3, 118.0, 117.6, 117.3, 111.2, 77.9, 52.1, 45.9, 31.3, 27.6, 23.1.

### 3.4. General Procedure for Boc-Deprotection

**A:** HCl in dioxane (4M, 10 equiv) or HCl in Et_2_O (2M, 10 equiv) was added to a solution of the Boc-protected amine (1 equiv) in dioxane or Et_2_O and the mixture was stirred overnight. The obtained hydrochloride salt was filtered and used in the next step without further purification.

**B:** The Boc-protected amine (1 equiv) was dissolved in dry DCM and MeOH (10 equiv) under a nitrogen atmosphere and cooled to 0 °C in an ice bath. Acetyl chloride (10 equiv) was added dropwise, and the mixture was stirred for 2 h. The obtained hydrochloric salt was filtered and used in the next step without further purification.

#### 3.4.1. 1-(2-Methoxyphenyl)-3-(pyrrolidin-2-yl)imidazo [1,5-a]pyridine hydrochloride (**5a**)

Method A. **4a** (235 mg, 0.59 mmol), HCl in dioxane (1.5 mL, 5.9 mmol). Beige salt (170 mg, 99%). ^1^H NMR (400 MHz, Methanol-d_4_) *δ* 8.19 (dt, *J* = 7.2, 1.1 Hz, 1H), 7.53 (ddd, *J* = 7.5, 1.8, 0.4 Hz, 1H), 7.42 (dt, *J* = 9.3, 1.2 Hz, 1H), 7.34 (ddd, *J* = 8.3, 7.4, 1.8 Hz, 1H), 7.10 (dd, *J* = 8.3, 1.1 Hz, 1H), 7.04 (ddd, *J* = 7.5, 7.4, 1.1 Hz, 1H), 6.77 (ddd, *J* = 9.3, 6.4, 1.1 Hz, 1H), 6.69 (ddd, *J* = 7.2, 6.4, 1.3 Hz, 1H), 4.64 (t, *J* = 7.6 Hz, 1H), 3.81 (s, 3H), 3.18 (ddd, *J* = 10.9, 7.4, 6.2 Hz, 1H), 2.98 (ddd, *J* = 10.9, 7.8, 6.2 Hz, 1H), 2.36–2.20 (m, 2H), 2.11–2.00 (m, 1H), 2.00–1.88 (m, 1H). ^13^C NMR (101 MHz, Methanol-d_4_) *δ* 158.0, 139.3, 132.3, 129.7, 129.6, 128.2, 124.6, 122.6, 121.7, 121.1, 119.8, 114.0, 112.4, 55.8, 55.3, 47.6, 31.2, 26.7.

#### 3.4.2. 1-(2-Methoxyphenyl)-3-(piperidin-2-yl)imidazo [1,5-a]pyridine hydrochloride (**5c**)

Method B. **4c** (300 mg, 0.74 mmol), acetyl chloride (525 µL, 7.4 mmol)), methanol (298 µL, 7.4 mmol) and DCM (7 mL). Beige salt (255 mg, 99%). ^1^H NMR (400 MHz, CD_3_OD) *δ* 8.80 (dd, *J* = 6.4, 1.8 Hz, 1H), 7.80 (dt, *J* = 9.2, 1.2 Hz, 1H), 7.66 (dd, *J* = 7.6, 1.7 Hz, 1H), 7.57 (ddd, *J* = 8.5, 7.5, 1.7 Hz, 1H), 7.36–7.28 (m, 2H), 7.26 (dd, *J* = 8.5, 1.0 Hz, 1H), 7.18 (td, *J* = 7.5, 1.0 Hz, 1H), 5.50 (dd, *J* = 12.3, 3.1 Hz, 1H), 3.92 (s, 3H), 3.67–3.60 (m, 1H), 3.53–3.44 (m, 1H), 2.68–2.55 (m, 1H), 2.40–2.32 (m, 1H), 2.20–2.12 (m, 1H), 2.09–2.00 (m, 2H), 1.99–1.85 (m, 1H). ^13^C NMR (101 MHz, CD_3_OD) *δ* 158.4, 133.1, 132.3, 130.5, 129.6, 125.4, 124.7, 124.1, 122.2, 120.7, 119.3, 116.9, 112.9, 56.3, 50.8, 46.9, 28.2, 23.4, 22.8.

#### 3.4.3. 1-(3-Methoxyphenyl)-3-(pyrrolidin-2-yl)imidazo [1,5-a]pyridine hydrochloride (**5d**)

Method B. **4d** (235 mg, 0.60 mmol), acetyl chloride (426 µL, 6.0 mmol)), methanol (242 µL, 6.0 mmol) and DCM (6 mL). Beige salt (180 mg, 94%). ^1^H NMR (400 MHz, CD_3_OD) *δ* 8.66 (dt, *J* = 7.2, 1.1 Hz, 1H), 8.03 (dt, *J* = 9.3, 1.2 Hz, 1H), 7.51 (ddd, *J* = 8.2, 7.2, 0.9 Hz, 1H), 7.46–7.41 (m, 2H), 7.33 (ddd, *J* = 9.3, 6.8, 0.9 Hz, 1H), 7.26 (td, *J* = 6.8, 1.2 Hz, 1H), 7.09 (ddd, *J* = 8.2, 2.4, 1.2 Hz, 1H), 5.69 (dd, *J* = 9.6, 7.6 Hz, 1H), 3.91 (s, 3H), 3.70–3.59 (m, 2H), 2.86–2.69 (m, 2H), 2.51–2.41 (m, 1H), 2.37–2.23 (m, 1H). ^13^C NMR (101 MHz, CD_3_OD) *δ* 161.8, 131.6, 130.8, 130.3, 129.2, 128.4, 125.6, 123.7, 121.4, 120.0, 119.1, 116.1, 114.7, 56.1, 53.6, 47.4, 30.2, 25.3.

#### 3.4.4. 1-(4-Methoxyphenyl)-3-(pyrrolidin-2-yl)imidazo [1,5-a]pyridine hydrochloride (**5e**)

Method B. **4e** (340 mg, 0.86 mmol), acetyl chloride (614 µL, 8.6 mmol), methanol (350 µL, 8.6 mmol) and DCM (8 mL). Beige salt (285mg, 99%). ^1^H NMR (400 MHz, CD_3_OD) *δ* 8.66 (dd, *J* = 7.0, 1.4 Hz, 1H), 8.00 (dt, *J* = 9.2, 1.3 Hz, 1H), 7.80 (dt, *J* = 8.8, 1.9 Hz, 2H), 7.36–7.25 (m, 2H), 7.17 (dt, *J* = 8.8, 1.9 Hz, 2H), 5.70 (dd, *J* = 9.8, 7.6 Hz, 1H), 3.90 (s, 3H), 3.68–3.60 (m, 2H), 2.87–2.69 (m, 2H), 2.52–2.41 (m, 1H), 2.37–2.23 (m, 1H). ^13^C NMR (101 MHz, CD_3_OD) *δ* 162.4, 130.9, 129.4, 128.6, 128.4, 125.4, 123.6, 121.2, 120.2, 119.5, 115.9, 56.0, 53.4, 47.4, 30.0, 25.4.

#### 3.4.5. *N*,*N*-Dimethyl-2-(3-(pyrrolidin-2-yl)imidazo [1,5-a]pyridin-1-yl)aniline hydrochloride (**5f**)

Method B. **4f** (147 mg, 0.36 mmol), acetyl chloride (258 µL, 3.6 mmol), methanol (146 µL, 3.6 mmol) and DCM (4 mL). Beige salt (123 mg, 99%). ^1^H NMR (400 MHz, CD_3_OD) *δ* 8.52 (dt, *J* = 7.2, 1.1 Hz, 1H), 8.07–7.97 (m, 3H), 7.69 (td, *J* = 7.6, 1.3 Hz, 1H), 7.62 (ddd, *J* = 8.2, 7.4, 1.6 Hz, 1H), 7.24 (ddd, *J* = 9.3, 6.5, 0.9 Hz, 1H), 7.07 (ddd, *J* = 7.5, 6.6, 1.1 Hz, 1H), 5.45 (dd, *J* = 9.1, 7.3 Hz, 1H), 3.76–3.67 (m, 1H), 3.59–3.53 (m, 1H), 3.50 (s, 6H), 2.76–2.62 (m, 2H), 2.48–2.36 (m, 1H), 2.35–2.26 (m, 1H). ^13^C NMR (101 MHz, CD_3_OD) *δ* 141.0, 133.9, 131.8, 131.5, 130.2, 129.9, 126.9, 126.3, 124.8, 123.7, 122.7, 119.3, 116.5, 55.4, 48.0, 46.7, 30.4, 24.6.

#### 3.4.6. 1-Cyclohexyl-3-(pyrrolidin-2-yl)imidazo [1,5-a]pyridine hydrochloride (**5g**)

Method B. **4g** (265 mg, 0.72 mmol), acetyl chloride (512 µL, 7.2 mmol), methanol (291 µL, 7.2 mmol) and DCM (7 mL). Beige salt (219 mg, 99%). ^1^H NMR (400 MHz, CD_3_OD) *δ* 8.65–8.61 (m, 1H), 8.04–7.98 (m, 1H), 7.31–7.25 (m, 2H), 5.67 (dd, *J* = 10.5, 7.4 Hz, 1H), 3.66–3.60 (m, 2H), 2.90–2.78 (m, 1H), 2.75–2.66 (m, 1H), 2.51–2.41 (m, 1H), 2.34–2.23 (m, 1H), 2.05–1.79 (m, 9H), 1.57–1.41 (m, 4H). ^13^C NMR (101 MHz, CD_3_OD) *δ* 133.3, 128.6, 127.8, 124.5, 123.5, 120.1, 120.1, 52.9, 47.5, 36.7, 33.4, 29.9, 27.4, 26.6, 25.4.

#### 3.4.7. 3-(Pyrrolidin-2-yl)imidazo [1,5-a]pyridine hydrochloride (**5h**)

Method A. **4h** (219 mg, 0.76 mmol), HCl in dioxane (1.9 mL, 7.6 mmol) and dioxane (7 mL). Beige salt (168 mg, 99%). ^1^H NMR (400 MHz, CD_3_OD) *δ* 8.18 (dt, *J* = 7.5, 1.4 Hz, 1H), 7.47 (dq, *J* = 9.5, 1.4 Hz, 1H), 7.32 (t, *J* = 1.3 Hz, 1H), 6.76 (ddt, *J* = 9.1, 6.5, 1.3 Hz, 1H), 6.65 (ddt, *J* = 7.8, 6.5, 1.4 Hz, 1H), 4.61 (td, *J* = 7.4, 1.7 Hz, 1H) 3.09 (dddd, *J* = 11.3, 8.3, 6.5, 1.5 Hz, 1H), 2.99 (dddd, *J* = 11.1, 7.4, 5.8, 1.4 Hz, 1H), 2.32–2.19 (m, 2H), 2.02 (dddd, *J* = 15.9, 11.9, 7.6, 5.8 Hz, 1H), 1.90 (dqdd, *J* = 12.3, 7.9, 6.5, 1.4 Hz, 1H). ^13^C NMR (101 MHz, CD_3_OD) *δ* 140.2, 132.6, 122.7, 120.3, 119.3, 118.7, 114.0, 55.0, 47.6, 31.1, 26.8.

### 3.5. General Procedure for Acylation

Method A: The appropriate isocyanate or isothiocyanate (1.2 equiv) was added to a solution of **5a**–**h** (1 equiv) and TEA (3 equiv) in MeCN. The mixture was stirred until completion, a maximum of 1 h, and thereafter concentrated and purified by silica flash column chromatography using MeOH in DCM as eluent to afford the pure product. A small fraction of all test compounds were also purified by reversed-phase HPC before biological testing.

Method B: A round-bottomed flask containing triphosgene (108 mg, 0.36 mmol) was degassed and flushed with nitrogen three times. It was cooled to 0 °C, and dry DCM (3 mL) was added. TEA (169 µL, 1.2 mmol) was added to the cooled solution, and the appropriate amine (0.6 mmol) and the reaction mixture was stirred for 1 h. Thereafter, the cooling bath was removed, and the mixture was allowed to reach room temperature. At that point, the mixture was stirred without septum for 10 min, followed by the addition of **5a** (100 mg, 0.30 mmol) and TEA (42 µL, 0.3 mmol) and the mixture was stirred for 30 min. The mixture was concentrated and purified by silica flash column chromatography using MeOH in DCM as eluent. Yields were calculated, and NMR experiments were conducted. Thereafter, a small fraction of all test compounds were also purified by reversed-phase HPLC before biological testing.

#### 2-(1-(2-Methoxyphenyl)imidazo [1,5-a]pyridin-3-yl)-*N*-((*S*)-1-phenylethyl)pyrrolidine-1-carboxamide (**6a**) [49]

Method A. **5a** (100 mg, 0.30 mmol), (*S*)-(-)-α-methylbenzyl isocyanate (52 μL, 0.37 mmol), TEA (127 μL, 0.91 mmol) and MeCN (3 mL). Yellow semi-solid (117 mg, 88%). ^1^H NMR (400 MHz, DMSO-d6, mixture of 2 diastereomers) *δ* 8.59–8.54 (m, 1H + 1H, 2 isomers), 7.61 (dt, *J* = 9.3, 1.2 Hz, 1H), 7.58 (dt, *J* = 3.4, 1.2 Hz, 1H), 7.56–7.54 (m, 1H), 7.53–7.48 (m, 1H + 1H, 2 isomers), 7.40 (dd, *J* = 7.6, 1.8 Hz, 1H), 7.33–7.22 (m, 5H + 5H, 2 isomers), 7.22–7.02 (m, 4H + 4H, 2 isomers), 6.87–6.80 (m, 1H + 1H, 2 isomers), 5.61 (dd, *J* = 7.8, 4.2 Hz, 1H), 5.59 (dd, *J* = 7.7, 4.1 Hz, 1H), 4.76 (dd, *J* = 15.2, 7.6 Hz, 1H + 1H, 2 isomers), 3.85 (s, 3H), 3.77 (s, 3H), 3.76–3.69 (m, 1H + 1H, 2 isomers), 3.55–3.47 (m, 1H + 1H, 2 isomers), 2.42–2.31 (m, 1H + 1H, 2 isomers), 2.21–2.10 (m, 2H + 2H, 2 isomers), 2.09–2.00 (m, 1H + 1H, 2 isomers), 1.37 (d, *J* = 7.2 Hz, 3H), 1.34 (d, *J* = 6.1 Hz, 3H). ^13^C NMR (101 MHz, DMSO-d6, mixture of diastereomers) *δ* 158.4, 158.0, 156.4, 156.3, 155.8, 155.7, 146.0, 145.5, 138.91, 138.89, 131.3, 131.2, 128.1, 128.0, 126.43, 126.36, 126.34, 126.28, 125.92, 125.91, 122.9, 122.8, 120.72, 120.68, 119.5, 119.4, 111.9, 55.6, 55.5, 52.2, 52.0, 49.3, 49.1, 46.2, 30.83, 30.77, 24.60, 24.56, 22.9, 22.8. HRMS (ESI+): *m/z* [M + H]^+^ calculated for C_27_H_29_N_4_O_2_: 441.2291, found: 441.2289.

### 3.6. Chiral Separation of Compound **6a**

**Analytical run**. Supercritical fluid chromatography (SFC) analysis was performed on an SFC system connected to a back pressure regulator and a PDA detector. The sample was diluted to a concentration of around 1 mg/mL, and 10 µL was injected into a 5 µm CHIRAL ART Cellulose-SB, 4.6 × 150 mm (diameter × length) column held at 45 °C. An isocratic condition of 35% MeOH in CO_2_ was applied at a flow rate of 5 mL/min. The back pressure was set to 120 bar. The PDA scanned from 220 to 400.

**Preparative run**. The diastereomers of 6a were separated by preparative SFC. The sample for chiral separation was prepared by dissolving 20 mg in 600 µL MeOH, and the preparative run was performed by stacked injections on an SFC system connected to a PDA detector. The column used was a 5 µM CHIRAL ART Cellulose-SB, 10 × 250 mm (diameter × length) column held at 45 °C. An isocratic condition of 35% MeOH in CO_2_ was applied at a flow rate of 15 mL/min. The backpressure was set to 120 bars. The PDA was scanned from 22+ to 400, and the diastereomers were collected in separate fractions (with the aid of 2 mL/min MeOH as the make-up solvent for the collection) and pooled from each injection.

**6a_dia1**. ^1^H NMR (400 MHz, CD_3_OD) *δ* 8.15 (dt, *J* = 7.3, 1.2 Hz, 1H), 7.45 (dd, *J* = 7.5, 1.8 Hz, 1H), 7.40 (dt, *J* = 9.3, 1.2 Hz, 1H), 7.34 (ddd, *J* = 8.3, 7.4, 1.8 Hz, 1H), 7.29–7.25 (m, 2H), 7.23–7.18 (m, 2H), 7.17–7.13 (m, 1H), 7.09 (dd, *J* = 8.4, 1.1 Hz, 1H), 7.03 (td, *J* = 7.5, 1.1 Hz, 1H), 6.77 (ddd, *J* = 9.3, 6.4, 1.0 Hz, 1H), 6.67 (ddd, *J* = 7.5, 6.3, 1.3 Hz, 1H), 5.48 (dd, *J* = 7.6, 5.0 Hz, 1H), 4.81 (q, *J* = 7.0 Hz, 1H), 3.84–3.79 (m, 1H),3.77 (s, 3H), 3.59 (ddd, *J* = 9.5, 7.4, 5.9 Hz, 1H), 2.44–2.35 (m, 1H), 2.31–2.22 (m, 1H), 2.22–2.13 (m, 1H), 2.12–2.03 (m, 1H), 1.35 (d, *J* = 6.9 Hz, 3H). ^13^C NMR (101 MHz, CD_3_OD) *δ* 158.4, 158.1, 146.0, 140.1, 132.5, 129.9, 129.3, 129.2, 127.7, 127.1, 126.8, 124.3, 122.7, 121.7, 121.2, 119.7, 114.3, 112.4, 55.8, 55.1, 51.1, 47.6, 32.3, 25.7, 23.0.

**6a_dia2**. ^1^H NMR (400 MHz, CD_3_OD) *δ* 8.12 (dt, *J* = 7.3, 1.2 Hz, 1H), 7.51 (dd, *J* = 7.5, 1.8 Hz, 1H), 7.43 (dt, *J* = 9.3, 1.3 Hz, 1H), 7.35 (ddd, *J* = 8.3, 7.3, 1.8 Hz, 1H), 7.21–7.07 (m, 6H), 7.04 (td, *J* = 7.5, 1.1 Hz, 1H), 6.78 (ddd, *J* = 9.3, 6.4, 1.0 Hz, 1H), 6.66 (ddd, *J* = 7.5, 6.3, 1.3 Hz, 1H), 5.43 (dd, *J* = 7.6, 5.3 Hz, 1H), 4.77 (q, *J* = 7.0 Hz, 1H), 3.84–3.77 (m, 1H), 3.80 (s, 3H), 3.66–3.58 (m, 1H), 2.43–2.34 (m, 1H), 2.31–2.14 (m, 2H), 2.11–2.02 (m, 1H), 1.36 (d, *J*= 0.0 Hz, 3H). ^13^C NMR (101 MHz, CD_3_OD) *δ* 158.4, 158.1, 146.5, 139.9, 132.5, 129.8, 129.4, 129.3, 128.3, 127.6, 126.8, 124.5, 122.8, 121.7, 121.2, 119.7, 114.1, 112.3, 55.8, 55.0, 51.3, 47.8, 32.5, 25.6, 23.2.

#### 3.6.1. 2-(1-(2-Methoxyphenyl)imidazo [1,5-a]pyridin-3-yl)-*N*-((*R*)-1-phenylethyl)pyrrolidine-1-carboxamide (**6b**)

Method A. **5a** (60 mg, 0.18 mmol), (*R*)-(-)-α-methylbenzyl isocyanate (29 μL, 0.21 mmol), TEA (51 μL, 0.37 mmol) and MeCN (2 mL). Green semi-solid (58 mg, 72%). 1H NMR (400 MHz, DMSO-d_6_, mixture of 2 diastereomers) *δ* 8.53 (d, *J* = 7.2 Hz, 1H + 1H, 2 isomers), 7.60– 7.56 (m, 1H), 7.53 (dd, *J* = 7.6, 1.7 Hz, 1H), 7.51–7.45 (m, 1H + 1H, 2 isomers), 7.40 (dd, *J* = 7.6, 1.7 Hz, 1H), 7.31–7.19 (m, 5H + 5H, 2 isomers), 7.18–7.09 (m, 2H + 2H, 2 isomers), 7.09–6.96 (m, 2H + 2H, 2 isomers), 6.79 (d, *J* = 8.1 Hz, 1H + 1H, 2 isomers), 5.59 (dd, *J* = 7.9, 4.0 Hz, 1H), 5.56 (dd, *J* = 7.9, 4.1 Hz, 1H), 4.74 (dq, *J* = 14.7, 7.4 Hz, 1H + 1H, 2 isomers), 3.83 (s, 3H), 3.76 (s, 3H), 3.73–3.66 (m, 1H + 1H, 2 isomers), 3.53–3.45 (m, 1H + 1H, 2 isomers), 2.38–2.28 (m, 1H + 1H, 2 isomers), 2.21–2.09 (m, 2H + 2H, 2 isomers), 2.08–1.98 (m, 1H + 1H, 2 isomers), 1.36 (d, *J* = 7.1 Hz, 3H), 1.32 (d, *J* = 7.1 Hz, 3H). ^13^C NMR (101 MHz, DMSO-d_6_, mixture of 2 diastereomers) *δ* 158.3, 158.0, 156.32, 156.27, 155.8, 155.7, 146.0, 145.5, 139.01, 138.87, 131.2, 131.1, 128.1, 128.0, 126.5, 126.4, 126.32, 126.27, 125.91, 125.90, 122.9, 122.7, 120.70, 120.65, 119.5, 119.4, 111.9, 55.54, 55.48, 52.2, 52.0, 49.3, 49.1, 46.2, 30.9, 30.8, 24.59, 24.56, 22.9, 22.8. HRMS (ESI+): *m/z* [M + H]^+^ calculated for C_27_H_29_N_4_O_2_: 441.2291, found: 441.2294.

#### 3.6.2. 2-(1-(2-Methoxyphenyl)imidazo [1,5-a]pyridin-3-yl)-*N*-(2-phenylpropan-2-yl)pyrrolidine-1-carboxamide (**6c**)

Method B. 2-phenylpropan-2-amine (87 µL). Green semi-solid (130 mg, 94%). ^1^H NMR (400 MHz, CD_3_OD) *δ* 8.36 (dt, *J* = 7.1, 1.2 Hz, 1H), 7.62 (dt, *J* = 9.3, 1.3 Hz, 1H), 7.56 (ddd, *J* = 8.4, 7.4, 1.7 Hz, 1H), 7.47 (dd, *J* = 7.6, 1.7 Hz, 1H), 7.34–7.30 (m, 2H), 7.24 (dd, *J* = 8.5, 1.0 Hz, 1H), 7.20–7.14 (m, 4H), 7.14–7.07 (m, 2H), 5.57–5.52 (m, 1H), 3.91–3.87 (m, 1H), 3.86 (s, 3H), 3.72–3.65 (m, 1H), 2.57–2.49 (m, 1H), 2.27–2.15 (m, 3H), 1.62 (s, 3H), 1.59 (s, 3H). ^13^C NMR (101 MHz, CD_3_OD) *δ* 158.5, 157.8, 149.3, 138.7, 133.0, 132.2, 129.1, 128.6, 127.0, 125.8, 124.5, 123.2, 122.1, 121.3, 120.6, 118.8, 116.5, 112.9, 56.8, 56.3, 54.3, 47.9, 31.9, 30.5, 30.4, 26.3. HRMS (ESI+): *m/z* [M + H]^+^ calculated for C_28_H_31_N_4_O_2_: 455.2447, found: 455.2462.

#### 3.6.3. 2-(1-(2-Methoxyphenyl)imidazo [1,5-a]pyridin-3-yl)-*N*-((*S*)-1-phenylpropyl)pyrrolidine-1-carboxamide (**6d**)

Method A. **5a** (51 mg, 0.16 mmol), (*S*)-(-)-1-phenylpropyl isocyanate (50 mg, 0.31 mmol), TEA (100 μL, 0.72 mmol) and MeCN (2 mL). White solid (70 mg, 81%). ^1^H NMR (400 MHz, DMSO-d_6_, mixture of two diastereomers) δ 8.41 (d, *J* = 7.2 Hz, 1H), 8.35 (d, *J* = 7.2 Hz, 0.2 H), 7.57 (dd, *J* = 7.6, 1.8 Hz, 1H), 7.49 (dd, *J* = 7.6, 1.8 Hz, 0.18H), 7.45 (d, *J* = 9.3 Hz, 1H), 7.40 (dt, *J* = 9.3, 1.2 Hz, 0.19 H), 7.31 (ddd, *J* = 8.4, 7.3, 1.8 Hz, 1H), 7.26–7.19 (m, 4H), 7.18–7.14 (m, 0.6 H), 7.12 (dd, *J* = 8.4, 1.1 Hz, 1H), 7.03 (td, *J* = 7.4, 1.1 Hz, 1H), 6.80–6.73 (m, 1H), 6.73–6.69 (m, 0.09 H), 6.66–6.62 (m, 1H), 6.59 (s, 0.08 H), 6.38 (d, *J* = 8.1 Hz, 1H), 5.49 (dd, *J* = 7.4, 3.3 Hz, 0.18 H), 5.43 (dd, *J* = 6.9, 4.2 Hz, 1H), 4.49 (q, *J* = 7.8 Hz, 1H), 3.82 (s, 3H), 3.78 (s, 0.5 H), 3.67–3.57 (m, 1H), 3.56–3.43 (m, 1H), 2.45–2.28 (m, 1H), 2.18 (d, *J* = 8.3 Hz, 2H), 2.02 (td, *J* = 7.2, 3.7 Hz, 1H), 1.63 (dq, *J* = 13.9, 7.2 Hz, 2H), 0.77 (t, *J* = 7.3 Hz, 3H). ^13^C NMR (126 MHz, DMSO-d_6_) δ 156.6, 156.2, 145.6, 145.4, 140.4, 131.2, 128.4, 128.3, 128.3, 127.3, 126.9, 126.8, 126.7, 126.5, 124.4, 123.2, 120.9, 120.2, 118.6, 112.1, 111.9, 56.0, 55.6, 52.5, 46.5, 31.5, 29.9, 25.0, 11.8. HRMS (ESI+): *m/z* [M + H]^+^ calculated for C_28_H_31_N_4_O_2_: 455.2447, found: 455.2440.

#### 3.6.4. (*S*)-*N*-Benzyl-2-(1-(2-methoxyphenyl)imidazo [1,5-a]pyridin-3-yl)pyrrolidine-1-carboxamide (**6e**)

Method A. **5a** (80 mg, 0.24 mmol), benzyl isocyanate (33 μL, 0.27 mmol), TEA (70 μL, 0.50 mmol) and MeCN (2 mL). White solid (49 mg, 48%). ^1^H NMR (400 MHz, DMSO-d_6_) *δ* 8.56 (d, *J* = 7.0 Hz, 1H), 7.60 (dt, *J* = 9.3, 1.3 Hz, 1H), 7.54–7.46 (m, 2H), 7.28–7.20 (m, 6H), 7.18–7.11 (m, 2H), 7.07 (dd, *J* = 12.1, 7.8 Hz, 1H), 5.62 (dd, *J* = 7.9, 3.7 Hz, 1H), 4.20 (qd, *J* = 15.6, 5.9 Hz, 2H), 3.82 (s, 3H), 3.46 (dt, *J* = 9.2, 7.0 Hz, 1H), 2.37 (dtt, *J* = 8.8, 6.6, 3.6 Hz, 1H), 2.20–2.01 (m, 3H). ^13^C NMR (101 MHz, DMSO-d_6_) *δ* 158.4, 158.0, 156.5, 156.4, 140.8, 138.9, 131.3, 128.1, 126.9, 126.5, 122.9, 122.1, 120.7, 119.5, 117.7, 115.8, 114.8, 111.9, 55.6, 52.2, 46.2, 43.2, 30.9, 24.5. HRMS (ESI+): *m*/*z* [M + H]^+^ calculated for C_26_H_27_N_4_O_2_: 427.2134, found: 427.2149

#### 3.6.5. 2-(1-(2-Methoxyphenyl)imidazo [1,5-a]pyridin-3-yl)-*N*-phenethylpyrrolidine-1-carboxamide (**6f**)

Method A. **5a** (50 mg, 0.15 mmol), phenethyl isocyanate (32 μL, 0.23 mmol), TEA (64 μL, 0.46 mmol) and MeCN (1.5 mL). White solid (47 mg, 69%). ^1^H NMR (400 MHz, CD_3_OD) *δ* 8.19 (dt, *J* = 7.2, 1.1 Hz, 1H), 7.51 (dd, *J* = 7.5, 1.8 Hz, 1H), 7.41 (dt, *J* = 9.2, 1.2 Hz, 1H), 7.33 (ddd, *J* = 8.3, 7.4, 1.8 Hz, 1H), 7.22–7.16 (m, 2H), 7.15–7.10 (m, 3H), 7.09 (dd, *J* = 8.4, 1.1 Hz, 1H), 7.03 (td, *J* = 7.5, 1.1 Hz, 1H), 6.77 (ddd, *J* = 9.2, 6.4, 1.0 Hz, 1H), 6.69 (ddd, *J* = 7.5, 6.4, 1.3 Hz, 1H), 5.47 (dd, *J* = 7.6, 4.6 Hz, 1H), 3.80 (s, 3H), 3.71 (ddd, *J* = 9.2, 7.4, 5.3 Hz, 1H), 3.46 (dt, *J* = 9.2, 6.9 Hz, 1H), 3.39–3.33 (m, 1H), 3.24 (dt, *J* = 13.3, 7.4 Hz, 1H), 2.70 (t, *J* = 7.4 Hz, 2H), 2.41–2.32 (m, 1H), 2.30–2.20 (m, 1H), 2.18–2.10 (m, 1H), 2.09–2.01 (m, 1H). ^13^C NMR (101 MHz, CD_3_OD) *δ* 159.3, 158.1, 140.8, 140.4, 132.5, 129.8, 129.8, 129.4, 129.1, 128.1, 127.1, 124.5, 122.7, 121.7, 121.1, 119.6, 114.0, 112.4, 55.8, 54.7, 47.5, 43.3, 37.6, 32.5, 25.6. HRMS (ESI+): *m/z* [M + H]^+^ calculated for C_27_H_29_N_4_O_2_: 441.2291, found: 441.2288.

#### 3.6.6. 2-(1-(2-Methoxyphenyl)imidazo [1,5-a]pyridin-3-yl)-*N*-(3-phenylpropyl)pyrrolidine-1-carboxamide (**6g**)

Method A. **5a** (50 mg, 0.15 mmol), 3-phenylpropyl isocyanate (35 μL, 0.23 mmol), TEA (43 μL, 0.31 mmol) and MeCN (2 mL). White solid (47 mg, 68%). ^1^H NMR (400 MHz, CD_3_OD) *δ* 8.18 (dt, *J* = 7.2, 1.2 Hz, 1H), 7.50 (dd, *J* = 7.5, 1.8 Hz, 1H), 7.40 (dt, *J* = 9.2, 1.3 Hz, 1H), 7.32 (ddd, *J* = 8.3, 7.4, 1.8 Hz, 1H), 7.21–7.16 (m, 2H), 7.13–7.05 (m, 4H), 6.98 (td, *J* = 7.5, 1.1 Hz, 1H), 6.76 (ddd, *J* = 9.3, 6.4, 1.0 Hz, 1H), 6.69 (ddd, *J* = 7.6, 6.3, 1.3 Hz, 1H), 5.46 (dd, *J* = 7.6, 4.7 Hz, 1H), 3.76 (s, 3H), 3.74–3.70 (m, 1H), 3.48 (ddd, *J* = 9.3, 7.3, 6.2 Hz, 1H), 3.16 (dt, *J* = 13.7, 6.9 Hz, 1H), 3.06 (dt, *J* = 13.6, 7.0 Hz, 1H), 2.50 (t, *J* = 7.8 Hz, 2H), 2.42–2.33 (m, 1H), 2.30–2.20 (m, 1H), 2.20–2.11 (m, 1H), 2.10–2.01 (m, 1H), 1.76–1.67 (m, 2H). ^13^C NMR (101 MHz, CD_3_OD) *δ* 159.3, 158.1, 143.3, 140.3, 132.5, 129.7, 129.4, 129.3, 129.2, 128.2, 126.7, 124.5, 122.7, 121.7, 121.2, 119.6, 114.1, 112.4, 55.8, 54.8, 47.5, 41.2, 34.1, 33.2, 32.5, 25.6. HRMS (ESI+): *m/z* [M + H]^+^ calculated for C_28_H_31_N_4_O_2_: 455.2447, found: 455.2450.

#### 3.6.7. *N*-(Furan-2-ylmethyl)-2-(1-(2-methoxyphenyl)imidazo [1,5-a]pyridin-3-yl)pyrrolidine-1-carboxamide (**6i**)

Method B. 2-furfurylmethanamine (54 µL). Green oil (126 mg, 40%). ^1^H NMR (400 MHz, DMSO-*d*_6_) δ 8.43 (d, *J* = 7.2 Hz, 1H), 7.54 (dd, *J* = 7.6, 1.8 Hz, 1H), 7.50–7.45 (m, 1H), 7.41 (d, *J* = 9.3 Hz, 1H), 7.28 (ddd, *J* = 8.9, 7.3, 1.8 Hz, 1H), 7.09 (dd, *J* = 8.4, 1.1 Hz, 1H), 7.01 (td, *J* = 7.5, 1.1 Hz, 1H), 6.76–6.72 (m, 1H), 6.64 (td, *J* = 6.7, 6.2, 1.3 Hz, 1H), 6.29 (dd, *J* = 3.2, 1.8 Hz, 1H), 6.11 (d, *J* = 3.2 Hz, 1H), 5.47 (dd, *J* = 7.5, 3.2 Hz, 1H), 4.20 (dd, *J* = 15.8, 5.9 Hz, 1H), 4.08 (dd, *J* = 15.8, 5.6 Hz, 1H), 3.79 (s, 3H), 3.63–3.47 (m, 1H), 3.47–3.37 (m, 1H), 2.38–2.26 (m, 1H), 2.25–2.06 (m, 2H), 2.07–1.89 (m, 1H). ^13^C NMR (126 MHz, DMSO) δ 156.9, 156.2, 154.4, 142.0, 140.4, 131.2, 128.3, 126.5, 124.5, 123.2, 120.9, 120.2, 118.6, 112.0, 111.9, 110.7, 106.6, 55.6, 52.5, 46.3, 37.3, 31.5, 24.9. HRMS (ESI+): *m/z* [M + H]^+^ calculated for C_24_H_25_N_4_O_3_: 417.1927, found: 417.1927.

#### 3.6.8. *N*-(Cyclohexylmethyl)-2-(1-(2-methoxyphenyl)imidazo [1,5-a]pyridin-3-yl)pyrrolidine-1-carboxamide (**6j**)

Method B. Cyclohexanemethylamine (79 µL). Green oil (105 mg, 80%). ^1^H NMR (400 MHz, Chloroform-d) δ 8.13–8.06 (m, 1H), 7.63 (dd, *J* = 7.6, 1.8 Hz, 1H), 7.51 (dt, *J* = 9.3, 1.2 Hz, 1H), 7.32 (ddd, *J* = 8.2, 7.4, 1.8 Hz, 1H), 7.06 (td, *J* = 7.5, 1.1 Hz, 1H), 7.00 (dd, *J* = 8.2, 1.1 Hz, 1H), 6.71 (ddd, *J* = 9.3, 6.4, 1.0 Hz, 1H), 6.59 (ddd, *J* = 7.5, 6.3, 1.3 Hz, 1H), 5.41 (t, *J* = 6.8 Hz, 1H), 4.57 (t, *J* = 5.9 Hz, 1H), 3.85 (s, 3H), 3.83–3.75 (m, 1H), 3.70–3.60 (m, 1H), 3.11 (dt, *J* = 13.4, 6.7 Hz, 1H), 2.75 (ddd, *J* = 13.3, 6.6, 4.7 Hz, 1H), 2.45–2.33 (m, 3H), 2.13–1.99 (m, 1H), 1.57–1.46 (m, 3H), 1.37 (t, *J* = 13.6 Hz, 2H), 1.22–1.11 (m, 1H), 1.08–0.91 (m, 3H), 0.62 (qd, *J* = 11.9, 3.0 Hz, 2H). ^13^C NMR (101 MHz, CDCl_3_) δ 163.5, 157.5, 156.4, 137.7, 131.4, 128.8, 128.5, 122.1, 121.1, 120.7, 118.7, 113.1, 111.3, 55.5, 54.9, 47.2, 46.8, 38.3, 31.7, 30.6, 30.6, 26.5, 25.9, 25.0. HRMS (ESI+): *m/z* [M + H]^+^ calculated for C_26_H_33_N_4_O_2_: 433.2604, found: 433.2605.

#### 3.6.9. *N*-Butyl-2-(1-(2-methoxyphenyl)imidazo [1,5-a]pyridin-3-yl)pyrrolidine-1-carboxamide (**6k**)

Method B. n-Butylamine (60 µL). Green oil (95 mg, 80%). ^1^H NMR (400 MHz, CD_3_OD) *δ* 8.18 (dt, *J* = 7.2, 1.2 Hz, 1H), 7.51 (dd, *J* = 7.6, 1.8 Hz, 1H), 7.40 (dt, *J* = 9.2, 1.3 Hz, 1H), 7.33 (ddd, *J* = 8.3, 7.4, 1.8 Hz, 1H), 7.09 (dd, *J* = 8.3, 1.0 Hz, 1H), 7.03 (td, *J* = 7.4, 1.1 Hz, 1H), 6.76 (ddd, *J* = 9.3, 6.4, 1.0 Hz, 1H), 6.68 (ddd, *J* = 7.5, 6.3, 1.3 Hz, 1H), 5.47 (dd, *J* = 7.6, 4.7 Hz, 1H), 3.80 (s, 3H), 3.74 (ddd, *J* = 9.3, 7.4, 5.4 Hz, 1H), 3.50 (dt, *J* = 9.2, 6.8 Hz, 1H), 3.12 (dt, *J* = 14.0, 7.1 Hz, 1H), 3.01 (dt, *J* = 13.6, 7.0 Hz, 1H), 2.43–2.33 (m, 1H), 2.26 (tdd, *J* = 13.9, 9.4, 6.5 Hz, 1H), 2.15 (dtd, *J* = 11.7, 5.9, 4.7 Hz, 1H), 2.11–2.01 (m, 1H), 1.39 (dtd, *J* = 8.8, 7.6, 7.1, 6.0 Hz, 2H), 1.25 (dq, *J* = 9.8, 7.3 Hz, 2H), 0.85 (t, *J* = 7.3 Hz, 3H). ^13^C NMR (101 MHz, CD_3_OD) *δ* 159.4, 158.1, 140.4, 132.5, 129.7, 129.1, 128.2, 124.6, 122.7, 121.7, 121.1, 119.5, 114.0, 112.4, 55.8, 54.8, 47.5, 41.3, 33.5, 32.5, 25.7, 20.9, 14.2. HRMS (ESI+): *m/z* [M + H]^+^ calculated for C_23_H_29_N_4_O_2_: 393.2291, found: 393.2307.

#### 3.6.10. *N*-Allyl-2-(1-(2-methoxyphenyl)imidazo [1,5-a]pyridin-3-yl)pyrrolidine-1-carboxamide (**6l**)

Method B. Allylamine (46 µL). Green oil (70 mg, 61%). ^1^H NMR (400 MHz, Chloroform-d) δ 8.23 (d, *J* = 7.2 Hz, 1H), 7.65 (dd, *J* = 7.6, 1.8 Hz, 1H), 7.50 (dt, *J* = 9.3, 1.3 Hz, 1H), 7.30 (ddd, *J* = 8.2, 7.4, 1.8 Hz, 1H), 7.05 (td, *J* = 7.5, 1.1 Hz, 1H), 7.00 (dd, *J* = 8.3, 1.1 Hz, 1H), 6.70 (ddd, *J* = 9.3, 6.3, 1.0 Hz, 1H), 6.58 (ddd, *J* = 7.3, 6.4, 1.3 Hz, 1H), 5.78–5.66 (m, 1H), 5.47 (dd, *J* = 7.7, 5.2 Hz, 1H), 5.00–4.91 (m, 2H), 4.54 (t, *J* = 5.7 Hz, 1H), 3.85 (s, 3H), 3.83–3.77 (m, 1H), 3.75–3.57 (m, 3H), 2.59–2.43 (m, 2H), 2.39–2.29 (m, 1H), 2.15–2.04 (m, 1H). ^13^C NMR (101 MHz, CDCl_3_) δ 157.3, 156.5, 138.2, 135.4, 131.5, 128.6, 128.3, 127.5, 124.1, 122.2, 121.1, 120.6, 118.4, 115.4, 112.8, 111.4, 55.6, 54.2, 46.9, 43.1, 31.5, 25.3. HRMS (ESI+): *m/z* [M + H]^+^ calculated for C_22_H_25_N_4_O_2_: 377.1978, found: 377.1982.

#### 3.6.11. (*S*)-1-((1-(2-Methoxyphenyl)imidazo [1,5-a]pyridin-3-yl)methyl)-1-methyl-3-(1-phenylethyl)urea (**6m**)

Method A. **5b** (100 mg, 0.26 mmol), (*S*)-(-)-α-methylbenzyl isocyanate (40 μL, 0.28 mmol), TEA (92 μL, 0.6 mmol) and MeCN (3 mL). Light green solid (109 mg, 52%).^1^H NMR (400 MHz, DMSO-d_6_) δ 8.26 (d, *J* = 7.2 Hz, 1H), 7.67–7.58 (m, 1H), 7.49 (d, *J* = 9.4 Hz, 1H), 7.33 (d, *J* = 7.5 Hz, 3H), 7.28 (d, *J* = 7.2 Hz, 2H), 7.23–7.16 (m, 1H), 7.13 (dd, *J* = 8.3, 1.1 Hz, 1H), 7.04 (dd, *J* = 7.5, 1.1 Hz, 1H), 6.83–6.78 (m, 1H), 6.65–6.60 (m, 1H), 4.90 (dd, *J* = 8.5, 6.4 Hz, 1H), 4.86 (d, *J* = 4.0 Hz, 2H), 3.82 (s, 4H), 1.39 (d, *J* = 7.1 Hz, 4H). ^13^C NMR (126 MHz, DMSO-d_6_) δ 157.7, 156.2, 146.4, 135.9, 131.1, 128.5, 128.0, 126.7, 126.4, 122.9, 120.9, 120.4, 119.2, 112.8, 112.0, 55.6, 50.1, 43.8, 34.0, 23.2. HRMS (ESI+): *m/z* [M + H]^+^ calculated for C_25_H_27_N_4_O_2_: 415.2134, found: 415.2127.

#### 3.6.12. 2-(1-(2-Methoxyphenyl)imidazo [1,5-a]pyridin-3-yl)-*N*-((*S*)-1-phenylethyl)piperidine-1-carboxamide (**6n**)

Method A. **5c** (69 mg, 0.2 mmol), (*S*)-(-)-α-methylbenzyl isocyanate (34 μL, 0.24 mmol), TEA (84 μL, 0.6 mmol) and MeCN (2 mL). Light yellow solid (91 mg, 99%).^1^H NMR (400 MHz, DMSO-d_6_) δ 8.17 (d, *J* = 7.3 Hz, 1H), 7.64 (dd, *J* = 7.6, 1.8 Hz, 2H), 7.51 (d, *J* = 9.3 Hz, 1H), 7.37–7.26 (m, 7H), 7.25–7.17 (m, 1H), 7.13 (dd, *J* = 8.4, 1.1 Hz, 2H), 7.05 (td, *J* = 7.4, 1.1 Hz, 2H), 6.91 (d, *J* = 7.8 Hz, 2H), 6.79 (ddd, *J* = 9.3, 6.4, 1.0 Hz, 2H), 6.66 (ddd, *J* = 7.4, 6.3, 1.2 Hz, 2H), 5.92 (d, *J* = 5.7 Hz, 2H), 4.90 (t, *J* = 7.3 Hz, 2H), 3.83 (s, 6H), 2.92 (t, *J* = 12.7 Hz, 2H), 2.38 (d, *J* = 13.3 Hz, 1H), 2.17 (s, 1H), 1.97–1.54 (m, 4H), 1.59–1.40 (m, 1H). ^13^C NMR (101 MHz, DMSO-d_6_) δ 157.1, 156.2, 146.7, 138.4, 131.3, 128.6, 128.3, 127.4, 126.7, 126.4, 126.3, 124.5, 122.7, 121.0, 120.4, 118.9, 112.6, 112.0, 55.7, 50.1, 46.3, 27.8, 25.9, 23.3, 20.5. HRMS (ESI+): *m/z* [M + H]^+^ calculated for C_28_H_31_N_4_O_2_: 455.2447, found: 455.2438.

#### 3.6.13. 2-(1-(2-Methoxyphenyl)imidazo [1,5-a]pyridin-3-yl)-*N*-phenethylpiperidine-1-carboxamide (**6o**)

Method A. **5c** (69 mg, 0.2 mmol),phenethyl isocyanate (34 μL, 0.24 mmol), TEA (84 μL, 0.6 mmol) and MeCN (2 mL). Light yellow solid (91 mg, 99%).^1^H NMR (400 MHz, Methanol-d_4_) δ 8.04 (dt, *J* = 7.3, 1.1 Hz, 1H), 7.62 (dd, *J* = 7.5, 1.8 Hz, 1H), 7.46 (d, *J* = 9.3 Hz, 1H), 7.34 (ddd, *J* = 8.3, 7.4, 1.8 Hz, 1H), 7.29–7.22 (m, 4H), 7.22–7.16 (m, 1H), 7.10 (dd, *J* = 8.4, 1.1 Hz, 1H), 7.05 (td, *J* = 7.4, 1.1 Hz, 1H), 6.76 (ddd, *J* = 9.3, 6.3, 1.0 Hz, 1H), 6.61 (ddd, *J* = 7.4, 6.4, 1.2 Hz, 1H), 5.98–5.91 (m, 1H), 3.82 (s, 3H), 3.57–3.50 (m, 1H), 3.47 (td, *J* = 7.1, 3.1 Hz, 1H), 2.85 (t, *J* = 7.3 Hz, 2H), 2.80–2.73 (m, 2H), 2.54–2.36 (m, 2H), 1.96–1.70 (m, 2H), 1.66–1.46 (m, 2H). ^13^C NMR (101 MHz, MeOD) δ 158.3, 156.7, 139.5, 136.7, 131.2, 128.6, 128.2, 128.1, 128.0, 128.0, 126.9, 125.8, 123.7, 121.8, 120.4, 119.7, 118.3, 112.4, 111.1, 54.5, 42.2, 41.0, 36.1, 26.6, 25.0, 20.4. HRMS (ESI+): *m/z* [M + H]^+^ calculated for C_28_H_31_N_4_O_2_: 455.2447, found: 455.2441.

#### 3.6.14. 2-(Imidazo [1,5-a]pyridin-3-yl)-*N*-((*S*)-1-phenylethyl)pyrrolidine-1-carboxamide (**6p**)

Method A. **5h** (60 mg, 0.27 mmol), (*S*)-(-)-α-methylbenzyl isocyanate (51 μL, 0.36 mmol), TEA (120 μL, 0.86 mmol) and MeCN (3 mL). White solid (90 mg, 53%). ^1^H NMR 1H NMR (400 MHz, DMSO-_d6_) δ 8.40 (dd, *J* = 7.3, 1.1 Hz, 1H), 7.59–7.42 (m, 1H), 7.27–7.21 (m, 5H), 7.18–7.13 (m, 1H), 6.71 (ddd, *J* = 9.1, 6.3, 0.9 Hz, 1H), 6.59 (ddd, *J* = 7.4, 6.3, 1.3 Hz, 1H), 6.41 (d, *J* = 7.8 Hz, 1H), 5.40 (dd, *J* = 7.7, 3.3 Hz, 1H), 4.87–4.48 (m, 1H), 3.57 (ddd, *J* = 9.4, 8.1, 4.2 Hz, 1H), 3.47 (dt, *J* = 9.5, 7.4 Hz, 1H), 3.32 (s, 4H), 2.41–2.25 (m, 1H), 2.21–1.95 (m, 3H), 1.28 (d, *J* = 7.1 Hz, 4H). ^13^C NMR (126 MHz, DMSO-d_6_) δ 156.3, 146.6, 140.6, 130.2, 128.5, 126.6, 126.3, 123.1, 118.6, 118.5, 118.2, 112.0, 52.2, 49.5, 46.3, 31.5, 25.0, 23.5. HRMS (ESI+): *m/z* [M + H]^+^ calculated for C_20_H_23_N_4_O: 335.1875, found: 335.1872.

#### 3.6.15. (*S*)-*N*-(1-Phenylethyl)pyrrolidine-1-carboxamide (**6q**)

Method A. Pyrrolidine (20 µL, 0.24 mmol), (*S*)-(-)-α-methylbenzyl isocyanate (34 µL, 0.24 mmol), TEA (33 µL, 0.24 mmol) and MeCN (2.5 mL). Light yellow oil (10 mg, 19%). ^1^H NMR (400 MHz, CD_3_CN) *δ* 7.38–7.27 (m, 4H), 7.25–7.17 (m, 1H), 5.25 (s, 1H), 4.96–4.83 (m, 1H), 3.33–3.19 (m, 4H), 1.92–1.79 (m, 4H), 1.41 (d, *J* = 7.1 Hz, 3H). ^13^C NMR (101 MHz, CD_3_CN) *δ* 157.1, 147.2, 129.2, 127.5, 126.9, 50.8, 46.4, 26.2, 23.4. HRMS (ESI+): *m/z* [M + H]^+^ calculated for C_13_H_19_N_2_O: 219.1497, found: 219.1498.

#### 3.6.16. 2-(1-(3-Methoxyphenyl)imidazo [1,5-a]pyridin-3-yl)-*N*-phenethylpyrrolidine-1-carboxamide (**6r**)

Method A. **5d** (66 mg, 0.2 mmol), phenethyl isocyanate (33 µL, 0.24 mmol), TEA (84 µL, 0.6 mmol) and MeCN (2 mL). Green semi-solid (81 mg, 92%). ^1^H NMR (400 MHz, Chloroform-d) δ 8.23 (d, *J* = 7.2 Hz, 1H), 7.67 (dt, *J* = 9.3, 1.2 Hz, 1H), 7.36–7.30 (m, 2H), 7.26 (dd, *J* = 8.9, 7.4 Hz, 1H), 7.13–7.08 (m, 2H), 7.07–7.02 (m, 1H), 6.96 (dt, *J* = 6.7, 1.6 Hz, 2H), 6.74 (ddd, *J* = 8.1, 2.5, 1.2 Hz, 1H), 6.67 (ddd, *J* = 9.3, 6.4, 1.0 Hz, 1H), 6.50 (ddd, *J* = 7.4, 6.4, 1.2 Hz, 1H), 5.34 (dd, *J* = 7.7, 4.5 Hz, 1H), 4.34 (t, *J* = 5.7 Hz, 1H), 3.79 (s, 3H), 3.46–3.34 (m, 3H), 3.23 (dtd, *J* = 12.3, 7.0, 5.4 Hz, 1H), 2.59 (t, *J* = 7.2 Hz, 2H), 2.56–2.46 (m, 1H), 2.33 (dddd, *J* = 12.5, 6.8, 5.7, 4.4 Hz, 1H), 2.25–2.15 (m, 1H), 1.98 (dddd, *J* = 14.4, 12.6, 7.2, 5.7 Hz, 1H). ^13^C NMR (101 MHz, CDCl_3_) δ 160.0, 157.3, 139.2, 138.6, 136.7, 130.1, 129.7, 128.7, 128.5, 127.3, 126.3, 122.7, 119.7, 119.2, 118.7, 112.7, 112.3, 111.9, 55.4, 53.6, 46.5, 41.8, 36.3, 31.6, 25.3. HRMS (ESI+): *m/z* [M + H]^+^ calculated for C_27_H_29_N_4_O_2_: 441.2291, found: 441.2299.

#### 3.6.17. 2-(1-(4-Methoxyphenyl)imidazo [1,5-a]pyridin-3-yl)-*N*-phenethylpyrrolidine-1-carboxamide (**6s**)

Method A. **5e** (66 mg, 0.2 mmol), phenethyl isocyanate (33 µL, 0.24 mmol), TEA (84 µL, 0.6 mmol) and MeCN (2 mL). Green-yellow solid (87 mg, 99%). 1H NMR (400 MHz, Chloroform-d) δ 8.21 (d, *J* = 7.2 Hz, 1H), 7.80 (dt, *J* = 8.7, 2.0 Hz, 2H), 7.71 (dd, *J* = 9.3, 1.2 Hz, 1H), 7.19 (dd, *J* = 7.7, 1.7 Hz, 2H), 7.14 (dt, *J* = 7.3, 1.5 Hz, 1H), 7.08 (dt, *J* = 7.0, 1.6 Hz, 2H), 7.01 (dt, *J* = 8.8, 2.1 Hz, 2H), 6.84 (dd, *J* = 9.3, 6.4 Hz, 1H), 6.73 (t, *J* = 6.9 Hz, 1H), 5.49 (t, *J* = 6.8 Hz, 1H), 4.64 (s, 1H), 3.85 (s, 3H), 3.55–3.38 (m, 2H), 3.36–3.25 (m, 1H), 2.70 (t, *J* = 7.0 Hz, 2H), 2.60–2.44 (m, 2H), 2.43–2.33 (m, 1H), 2.06 (dt, *J* = 12.0, 7.2 Hz, 1H). ^13^C NMR (101 MHz, CDCl_3_) δ 159.4, 157.5, 139.2, 137.7, 128.9, 128.8, 128.8, 128.6, 126.4, 122.2, 120.6, 119.2, 114.9, 114.6, 55.5, 53.1, 46.9, 41.9, 36.4, 31.8, 25.5. HRMS (ESI+): *m/z* [M + H]^+^ calculated for C_27_H_29_N_4_O_2_: 441.2291, found: 441.2313.

#### 3.6.18. 2-(1-(2-(Dimethylamino)phenyl)imidazo [1,5-a]pyridin-3-yl)-*N*-phenethylpyrrolidine-1-carboxamide (**6t**)

Method A. **5f** (69 mg, 0.2 mmol), phenethyl isocyanate (33 µL, 0.24 mmol), TEA (84 µL, 0.6 mmol) and MeCN (2 mL). Green oil (56 mg, %). ^1^H NMR (400 MHz, CDCl_3_) *δ* ^1^H NMR (400 MHz, Chloroform-d) δ 8.20 (d, *J* = 7.2 Hz, 1H), 7.62–7.53 (m, 1H), 7.50 (dd, *J* = 7.5, 1.8 Hz, 1H), 7.26 (s, 1H), 7.26–7.20 (m, 1H), 7.20–7.17 (m, 1H), 7.17–7.11 (m, 1H), 7.06 (td, *J* = 6.1, 1.4 Hz, 3H), 7.00 (dd, *J* = 7.4, 1.2 Hz, 1H), 6.67 (ddd, *J* = 9.3, 6.3, 1.0 Hz, 1H), 6.60–6.53 (m, 1H), 5.45 (dd, *J* = 7.6, 5.2 Hz, 1H), 4.53 (s, 0H), 3.75–3.48 (m, 2H), 3.45–3.18 (m, 2H), 2.65 (td, *J* = 7.0, 1.7 Hz, 2H), 2.54 (s, 5H), 2.43 (dq, *J* = 12.4, 6.0 Hz, 1H), 2.38–2.27 (m, 1H), 2.07 (ddd, *J* = 13.9, 9.7, 6.1 Hz, 1H). ^13^C NMR (101 MHz, CDCl_3_) *δ* 157.4, 151.4, 139.3, 138.0, 132.4, 130.2, 128.8, 128.6, 127.9, 127.5, 127.4, 126.4, 122.0, 121.4, 120.5, 118.1, 117.7, 112.8, 54.2, 46.8, 43.1, 42.1, 36.6, 31.7, 29.8, 25.2. HRMS (ESI+): *m/z* [M + H]^+^ calculated for C_28_H_32_N_5_O: 454.2607, found: 454.2629.

#### 3.6.19. 2-(1-Cyclohexylimidazo [1,5-a]pyridin-3-yl)-*N*-phenethylpyrrolidine-1-carboxamide (**6u**)

Method A. **5g** (61 mg, 0.2 mmol), phenethyl isocyanate (33 µL, 0.24 mmol), TEA (84 µL, 0.6 mmol) and MeCN (2 mL). Green oil (56 mg, 67%). ^1^H NMR (400 MHz, Chloroform-d) δ 7.86 (dd, *J* = 7.3, 1.3 Hz, 1H), 7.34 (dt, *J* = 9.2, 1.3 Hz, 1H), 7.18–7.16 (m, 1H), 7.08 (d, *J* = 1.3 Hz, 1H), 7.06 (d, *J* = 2.1 Hz, 1H), 6.90 (dd, *J* = 7.8, 1.7 Hz, 2H), 6.49 (ddd, *J* = 9.2, 6.3, 1.0 Hz, 1H), 6.38 (ddd, *J* = 7.4, 6.3, 1.3 Hz, 1H), 5.16 (t, *J* = 6.9 Hz, 1H), 4.65 (t, *J* = 6.0 Hz, 1H), 4.44 (t, *J* = 5.8 Hz, 1H), 3.70–3.58 (m, 1H), 3.48–3.39 (m, 1H), 3.36–3.28 (m, 3H), 3.18–3.08 (m, 1H), 2.83–2.71 (m, 1H), 2.68 (d, *J* = 7.0 Hz, 1H), 2.48 (t, *J* = 7.0 Hz, 2H), 2.25 (d, *J* = 4.9 Hz, 1H), 1.96–1.84 (m, 1H), 1.83–1.54 (m, 6H), 1.38–1.25 (m, 2H). ^13^C NMR (101 MHz, CDCl_3_) δ 158.25, 157.39, 139.36, 139.18, 136.96, 135.80, 128.84, 128.62, 128.40, 126.30, 126.15, 121.67, 118.48, 116.64, 112.40, 55.06, 46.95, 41.86, 41.59, 37.37, 36.52, 36.31, 33.46, 33.23, 31.22, 26.86, 26.14, 24.81. HRMS (ESI+): *m/z* [M + H]^+^ calculated for C_26_H_33_N_4_O: 417.2654, found: 417.2664.

#### 3.6.20. *N*-Benzyl-2-(1-(2-methoxyphenyl)imidazo [1,5-a]pyridin-3-yl)pyrrolidine-1-carbothioamide (**6v**)

Method A. **5a** (80 mg, 0.24 mmol), benzylisothiocyanate (26 µL, 0.27 mmol), TEA (71 µL, 0.51 mmol) and MeCN (2 mL). Green oil (55 mg, 52%). ^1^H NMR (400 MHz, DMSO-_d6_) *δ* 8.57 (d, *J* = 4.2 Hz, 1H), 8.31 (s, 1H), 7.66–7.58 (m, 1H), 7.50 (m, 2H), 7.35–7.03 (m, 9H), 6.10 (d, *J* = 5.1 Hz, 1H), 4.80 (dd, *J* = 15.4, 5.9 Hz, 1H), 4.67 (dd, *J* = 15.4, 5.5 Hz, 1H), 3.99–3.91 (m, 1H), 3.81 (s, 3H), 3.65–3.55 (m, 1H), 2.47–2.38 (m, 1H), 2.25–2.04 (m, 3H). ^13^C NMR (101 MHz, DMSO--d_6_) *δ* 179.4, 158.3, 158.0, 156.3, 139.4, 131.2, 128.0, 127.0, 126.6, 126.1, 122.5, 120.7, 119.5, 117.5, 111.9, 109.5, 55.5, 47.9, 30.6, 24.2. HRMS (ESI+): *m/z* [M + H]^+^ calculated for C_26_H_27_N_4_OS: 443.1906, found: 443.1905

#### 3.6.21. *N*-(4-Fluorobenzyl)-2-(1-(2-methoxyphenyl)imidazo [1,5-a]pyridin-3-yl)pyrrolidine-1-carbothioamide (**6w**)

Method A. **5a** (80 mg, 0.24 mmol), 4-fluorobenzyl isothiocyanate (40 µL, 0.27 mmol), TEA (71 µL, 0.51 mmol) and MeCN (2 mL). Green oil (83 mg, 74%). ^1^H NMR (400 MHz, DMSO-d_6_) *δ* 8.56 (s, 1H), 8.30 (s, 1H), 7.61 (d, *J* = 8.7 Hz, 1H), 7.53–7.46 (m, 2H), 7.32 (t, *J* = 7.1 Hz, 2H), 7.23 (d, *J* = 8.3 Hz, 1H), 7.13 (t, *J* = 7.6 Hz, 1H), 7.08 (d, *J* = 8.7 Hz, 3H), 6.09 (d, *J* = 5.5 Hz, 1H), 4.77 (dd, *J* = 15.3, 5.8 Hz, 1H), 4.63 (dd, *J* = 15.0, 5.4 Hz, 1H), 3.98–3.89 (m, 2H), 3.81 (s, 3H), 3.63–3.54 (m, 1H), 2.47–2.37 (m, 1H), 2.28–2.02 (m, 3H). ^13^C NMR (101 MHz, DMSO-d_6_) *δ* 179.4, 161.0 (*J_C-F_* = 242.4 Hz), 158.3, 157.9, 156.3, 135.6 (*J_C-F_* = 2.8 Hz), 131.1, 129.1 (*J_C-F_* = 8.1 Hz), 126.2, 122.5, 120.7, 119.5, 114.67 (*J_C-F_* = 21.3 Hz), 114.65, 111.9, 57.0, 55.5, 48.6, 47.2, 30.6, 24.1. HRMS (ESI+): *m/z* [M + H]^+^ calculated for C_26_H_26_FN_4_OS: 461.1811, found: 461.1792.

#### 3.6.22. 2-(1-(2-Methoxyphenyl)imidazo [1,5-a]pyridin-3-yl)-*N*-phenethylpyrrolidine-1-carbothioamide (**6x**)

Method A. **5a** (80 mg, 0.24 mmol), phenethyl isothiocyanate (40 µL, 0.27 mmol), TEA (71 µL, 0.51 mmol) and MeCN (2 mL). Green oil (77 mg, 70%). ^1^H NMR (400 MHz, DMSO-d_6_) *δ* 8.59 (d, *J* = 5.7 Hz, 1H), 7.89 (s, 1H), 7.65–7.59 (m, 1H), 7.52 (dd, *J* = 7.6, 1.7 Hz, 1H), 7.47 (d, *J* = 8.0 Hz, 1H), 7.31–7.25 (m, 2H), 7.22 (d, *J* = 8.4 Hz, 1H), 7.21–7.16 (m, 3H), 7.13 (td, *J* = 7.0, 4.0, 1.0 Hz, 2H), 7.10–7.05 (m, 1H), 6.13–6.07 (m, 1H), 3.89–3.84 (m, 1H), 3.82 (s, 3H), 3.74–3.63 (m, 1H), 3.53 (ddt, *J* = 20.3, 17.2, 7.3 Hz, 2H), 2.90–2.76 (m, 2H), 2.46–2.35 (m, 1H), 2.26–2.18 (m, 1H), 2.18–2.10 (m, 1H), 2.09–2.01 (m, 1H). ^13^C NMR (101 MHz, DMSO-d_6_) *δ* 178.8, 158.3, 157.9, 156.3, 139.3, 138.4, 131.2, 128.6, 128.4, 126.1, 122.5, 120.7, 119.4, 111.9, 56.8, 55.5, 48.4, 46.6, 34.8, 30.6, 24.1. HRMS (ESI+): *m/z* [M + H]^+^ calculated for C_27_H_29_N_4_OS: 457.2062, found: 457.2067.

#### 3.6.23. *N*-((*R*)-1-Hydroxy-3-phenylpropan-2-yl)-2-(1-(2-methoxyphenyl)imidazo [1,5-a]pyridin-3-yl)pyrrolidine-1-carboxamide (**6h**)

Step 1: D-Phenylalanine (380 mg, 2.3 mmol) was dissolved in dry MeOH (20 mL) and cooled to 0 °C under a nitrogen atmosphere. SOCl_2_ (671 µL, 9.2 mmol) was added dropwise, and thereafter, the reaction mixture was allowed to reach room temperature and stirred for 12 h. The D-phenylalanine methyl ester hydrochloride salt was concentrated and used in the next step without further purification. White solid (472 mg, 95%). Spectral data were in agreement with previously published data [62].

Step 2: D-phenylalanine methyl ester hydrochloride (128 mg, 0.59 mmol) and triphosgene (96 mg, 0.32 mmol) were added to a round-bottomed flask which was degassed and flushed with nitrogen. The flask was cooled to 0 °C, and DCM (6 mL) and TEA (230 µL, 1.65 mmol) were added, and the mixture was stirred for 45 min. Thereafter, it was allowed to reach room temperature and stirred for 45 min without septum. Thereafter, TEA (150 µL) and **5a** (158 mg, 0.54 mmol) were added, and the mixture was stirred for an additional 30 min. After completion of the reaction (LCMS), water was added, and the phases were separated. The aqueous phase was extracted once with DCM. The combined organic phases were dried over MgSO4, filtered and concentrated, and used in the next step without further purification.

Step 3: The crude product from step 2 was dissolved in dry THF, LiAlH4 was added, and the reaction was stirred for 4 h. A saturated solution of Rochelle salt (aq.) was added to quench the reaction. EtOAc was added, the phases were separated, and the aqueous phase was extracted once with EtOAc. The combined organic phases were dried over MgSO4, filtered and concentrated. The crude product was purified by silica flash column chromatography using MeOH in DCM as eluent to afford the product as a green-yellow semi-solid (175 mg, 69%). ^1^H NMR (400 MHz, DMSO-d_6_, mixture of 2 diastereomers) *δ* 8.51 (t, *J* = 7.1 Hz, 2H), 7.56 (d, *J* = 9.3 Hz, 2H), 7.51 (ddd, *J* = 9.3, 7.6, 1.7 Hz, 2H), 7.48–7.42 (m, 2H), 7.23–7.18 (m, 4H), 7.18–7.08 (m, 11H), 7.06–6.91 (m, 4H), 6.12–6.01 (m, 2H), 5.57–5.49 (m, 2H), 3.82 (s, 3H), 3.80 (s, 3H), 3.70 (dtt, *J* = 11.6, 7.9, 3.9 Hz, 4H), 3.63–3.54 (m, 3H), 3.42 (dt, *J* = 9.2, 7.0 Hz, 1H), 3.37–3.28 (m, 4H), 3.28–3.20 (m, 2H), 2.79 (ddd, *J* = 15.8, 13.5, 5.9 Hz, 2H), 2.65 (ddd, *J* = 13.6, 11.6, 8.1 Hz, 2H), 2.34–2.24 (m, 2H), 2.21–2.08 (m, 4H), 2.05–1.94 (m, 2H). ^13^C NMR (101 MHz, DMSO-d_6_, mixture of 2 diastereomers) *δ* 156.3, 156.2, 139.6, 139.5, 131.1, 129.1, 129.0, 128.0, 128.0, 126.5, 126.5, 125.8, 125.7, 122.8, 122.8, 120.6, 119.5, 119.4, 111.8, 63.1, 62.8, 55.5, 54.0, 53.8, 52.1, 51.9, 46.1, 46.1, 36.8, 36.8, 30.8, 24.5. HRMS (ESI+): *m/z* [M + H]^+^ calculated for C_28_H_31_N_4_O_3_: 471.2396, found: 471.2395.

### 3.7. Biology

#### 3.7.1. Overexpression of Human IRAP and Aminopeptidase N

Human IRAP and APN were overexpressed, as previously described [63].

#### 3.7.2. Membrane Preparations

Membrane preparations from frozen CHO cells and HEK293T cells with overexpressed IRAP and APN were prepared and handled as previously described [63].

#### 3.7.3. Concentration-Response Experiments in 96-Well Format

Details for the assay used to evaluate the inhibitory activity of the compounds have already been published [9].

### 3.8. Mechanism of Action Studies for Compound **6a**

The design of the enzyme kinetics experiments followed that of the inhibitory experiments, except for the substrate concentrations that were variable at 0.1, 0.2, 0.3, 0.4, 0.8, 1.6 and 2.4 mM. The serial dilution of the compound stock solution was reproduced across all eight rows of a set of four 96-well plates. Four technical replicates were obtained at each substrate concentration by adding the same substrate solution to the top or bottom four rows of each 96-well plate, i.e., a total of four plates were used for this experiment, including a no-substrate control. The absorbance of these plates was then measured every 10 min to follow the enzymatic reaction over time. Data obtained in the presence of 100 µM inhibitor were used to define maximal IRAP inhibition under these assay conditions, i.e., this slope was subtracted from those obtained at lower inhibitor concentrations. Data were fitted to a model for non-competitive inhibition within GraphPad and compared with models for competitive and uncompetitive binding. The non-competitive inhibition model explains the experimental data with a probability of 99.99%, compared to 0.01% and <0.01% for competitive and uncompetitive inhibition, respectively. For illustration purposes, data are also shown as a double-reciprocal plot, resulting in the expected decrease in V_max_ value with increasing inhibitor concentration while the K_m_ remains constant.

### 3.9. Compound Profiling

Pre-clinical profiling of the compounds was performed at AstraZeneca R&D in Gothenburg, Sweden, applying an integrated panel of assays referred to as the DMPK Wave 1 panel [63].

## 4. Conclusions

We have synthesized and evaluated a new class of small-molecule-based IRAP inhibitors with affinities down to the single digit µM range (IC_50_ = 1.0 µM for **6a_dia2**) and with selectivity towards the closely related APN. The obtained SAR concluded limited possibilities for structural modifications in the urea tail as well as in the central ring system. Alterations of the anisole part are more tolerated; however, the presence of this motif is important for inhibition. The compounds display similar affinity to IRAP regardless of source; hence, both the hamster and the human enzyme are inhibited. Unfortunately, this series suffers from poor metabolic stability and, in general, limited solubility. The compounds display a noncompetitive behavior with the substrate L-Leu-*p*NA, so in parallel with improving solubility and metabolic stability, we are also working on specifying the binding site. We have previously shown that another hit compound from the screen, which is uncompetitive with the same substrate, binds in the active site but not in the absolute vicinity of the catalytic Zn^2+^ [53]. We are currently working on investigations to see if this could also be the case for this inhibitor class. Work is also ongoing to elucidate the absolute stereochemistry of the pyrrolidine ring in the separated diastereomers **6a_dia1** and **6a_dia2**.

## Figures and Tables

**Table 1:** IRAP inhibitory capacity of selected analogs from the CBCS compound library.

Compound	Structure ^[a]^	IC_50_ ^[b]^ (µM)	pIC_50_^[c]^
**1a**	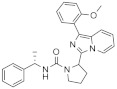	2.9	5.5 (1)
**1b**	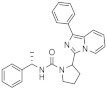	2.9	5.5 ± 0.2 (2)
**1c**	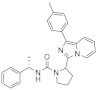	7.9	5.1 ± 0.05 (2)
**1d**	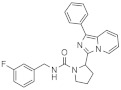	>125	<3.9 (2)
**1e**	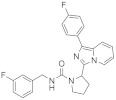	100	4.0 ± 0.3 (2)
**1f**	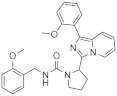	>125	<3.9 (2)
**1g**	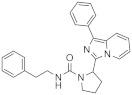	100	4.0 ± 0.2 (2)
**1h**	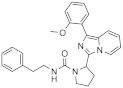	40	4.4 ± 0.1 (2)
**1i**	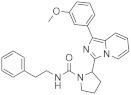	79	4.1 ± 0.1 (2)
**1j**	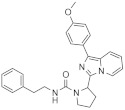	>125	<3.9 (1)
**1k**	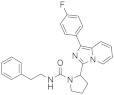	63	4.2 ± 0.2 (2)
**1l**	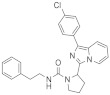	50	4.3 (1)
**1m**	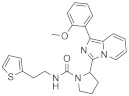	>125	<3.9 (2)
**1n**	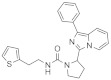	63	4.2 ± 0.06 (2)
**1o**	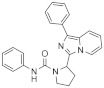	>125	<3.9 (1)
**1p**	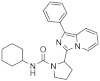	>125	<3.9 (1)
**1q**	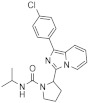	>125	<3.9 (1)
**1r**	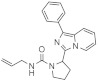	>125	<3.9 (1)
**1s**	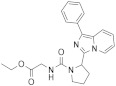	>125	<3.9 (1)
**1t**	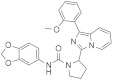	Inactive	inactive (1)
**1u**	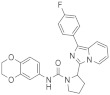	>125	<3.9 (1)
**1v**	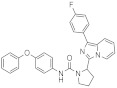	Inactive	inactive (1)
**1w**	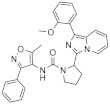	>125	<3.9 (1)

^[a]^ The purity of the library compounds was ensured by LC/MS analysis, ≥95% purity. ^[b]^ IC_50_ is defined as the molar compound concentration required to inhibit CHO cells’ IRAP activity by 50%. ^[c]^ pIC_50_ is defined as the negative log of IC_50_. Values represent the mean ± standard deviation of best-fit values from individual test occasions (the number of independent test occasions is provided within parentheses). No values over 125 µM are reported, as this was the highest compound concentration tested in the concentration–response experiment.

**Table 2:** Evaluation of IRAP inhibitory capacity of synthesized and resynthesized analogs of the hit compound.

Compounds	Structure	IC_50_ ^[a]^ (µM)	pIC_50_^[b]^
**6a**	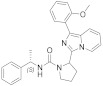	2.0	5.7 ± 0.1 (28)
**6a_dia1**	Data	16	4.8 ± 0.2 (4)
**6a_dia2**	Data	1.0	6.0 ± 0.1 (4)
**6b**	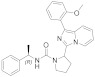	40	4.4 ± 0.1 (8)
**6c**	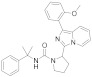	32	4.5 ± 0.07 (2)
**6d**	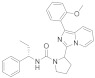	4.0	5.4 ± 0.04 (3)
**6e**	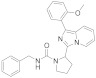	50	4.3 ± 0.2 (3)
**6f**	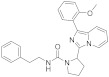	32	4.5 ± 0.1 (3)
**6g**	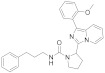	63	4.2 ± 0.3 (3)
**6h**	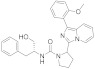	63	4.2 ± 0.2 (3)
**6i**	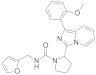	79	4.1 ± 0.03 (2)
**6j**	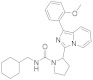	16	4.8 ± 0.2 (4)
**6k**	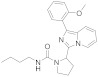	63	4.2 ± 0.04 (2)
**6l**	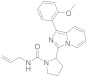	40	4.4 ± 0.4 (4)
**6m**	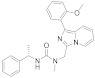	>125	<3.9 (3)
**6n**	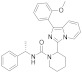	>125 ^[c]^	<3.9 (3) ^[c]^
**6o**	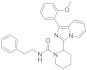	>125 ^[c]^	<3.9 (3) ^[c]^
**6p**	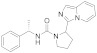	>125	<3.9 (3)
**6q**	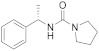	Inactive	Inactive (3)
**6r**	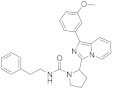	8.7	5.06 ± 0.07 (3)
**6s**	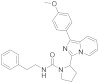	40	4.4 ± 0.08 (2)
**6t**	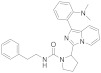	63	4.2 ± 0.09 (2)
**6u**	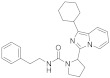	40	4.4 ± 0.5 (3)
**6v**	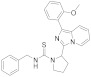	>125 ^[c]^	<3.9 (3) ^[c]^
**6w**	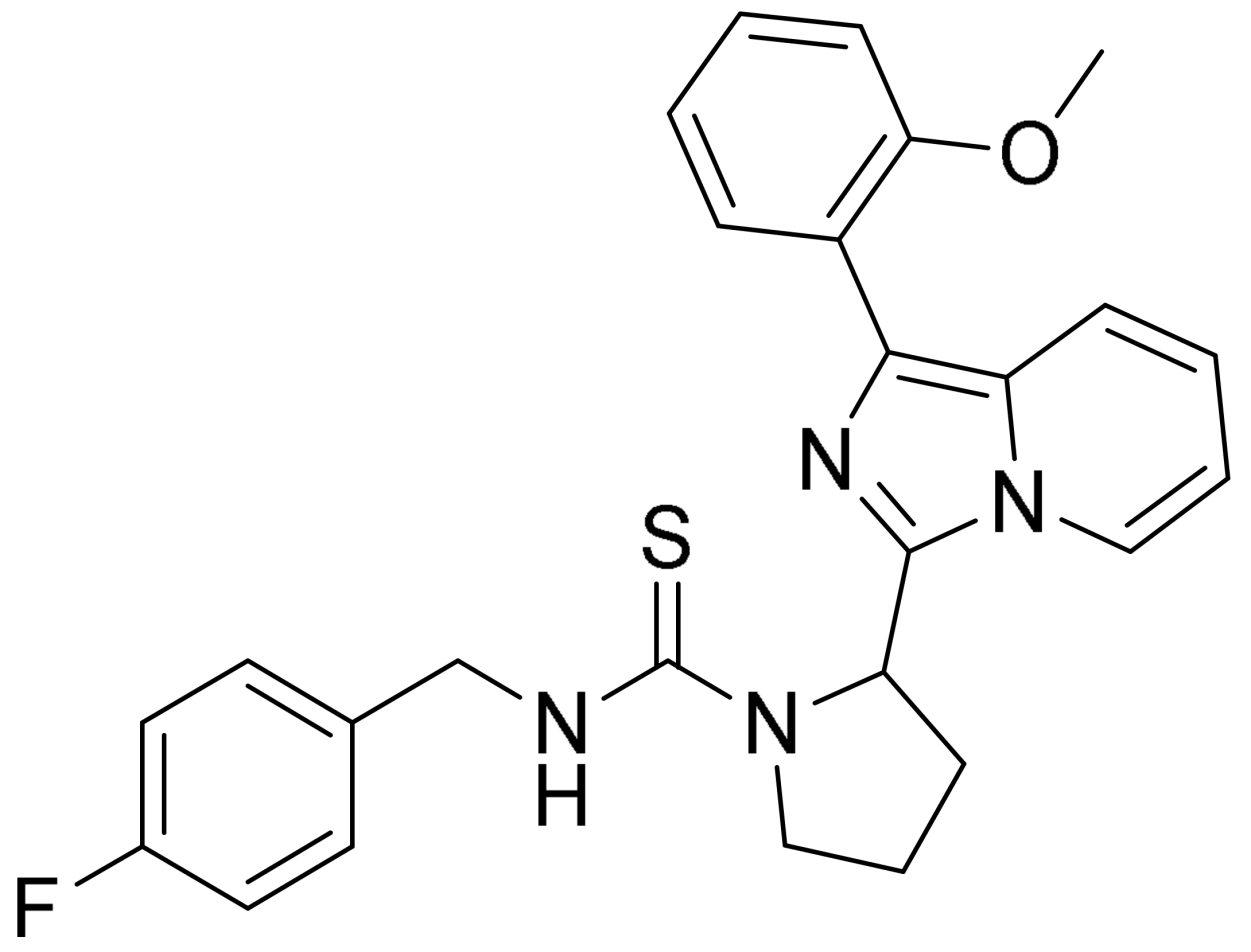	>125 ^[c]^	<3.9 (3) ^[c]^
**6x**	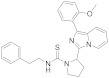	>125 ^[c]^	<3.9 (3) ^[c]^

^[a]^ IC_50_ is defined as the molar compound concentration required to inhibit CHO cells’ IRAP activity by 50%. ^[b]^ pIC_50_ is defined as the negative log of IC_50_. Values represent the mean ± standard deviation of best-fit values from individual test occasions (the number of independent test occasions is provided within brackets). No values over 125 µM are reported, as this was the highest compound concentration tested in the concentration–response experiment. ^[c]^ Inhibition was observed; however, specific potency was not possible to establish due to solubility issues and visual precipitation in the assay.

**Table 3:** Data for selected IRAP inhibitors.

Compounds	LogD_7.4_	Sol. ^[a]^	HLM Cl_int_^[b]^	HLM t ½	Rat hep. Cl_int_^[c]^	Rat hep. t ½	PPB ^[d]^	Plasma Stab ^[e]^
**6a_dia1**	2.8 ± 0.7	40–124 ^[f]^	>300 ^[h]^	<2.3 ^[h]^	>300 ^[h]^	<2.3 ^[h]^	1.7 ± 0.3	102 ± 5
**6a_dia2**	2.6 ± 0.7	76 ± 14 ^[g]^	>300 ^[h]^	<2.3 ^[h]^	>300 ^[h]^	<2.3 ^[h]^	1.8 ± 0.4	104 ± 3
**6e**	2.7 ± 0.4	2.2–11 ^[f]^	>300 ^[h]^	<2.3 ^[h]^	>300 ^[h]^	<2.3 ^[h]^	1.8 ± 0.4	97 ± 3
**6m**	2.9 ± 0.6	55 ± 12	>300 ^[h]^	<2.3 ^[h]^	>300 ^[h]^	<2.3 ^[h]^	0.6 ± 0.1	99 ± 5
**6v**	3.3 ± 0.4	0.6–2 ^[f]^	>300 ^[h]^	<2.3 ^[h]^	>300 ^[h]^	<2.3 ^[h]^	0.7 ± 0.1	76 ± 6

All the data are reported as the average and standard deviation from three independent test occasions. ^[a]^ Dried DMSO solubility in aqueous phosphate buffer (PBS) at pH 7.4; µM. ^[b]^ Human liver microsome intrinsic clearance; µL/min/mg. ^[c]^ Rat hepatocytes intrinsic clearance; µL/min/10^6^ cells. ^[d]^ Human protein plasma binding; % free fraction. ^[e]^ Human plasma stability; % compound remaining after 18 h incubation. ^[f]^ High-throughput solubility assays are associated with large variability, especially below the 10 µM range. When the standard deviation is higher than half the mean value, the entire range is given. ^[g]^ An outlier value was observed with higher solubility amongst the three replicates. The higher value was removed. ^[h]^ A higher uncertainty is associated with these values as compound dilutions result in non-linear responses.

**Table 4:** Evaluation of selected compounds on IRAP from different species as well as APN.

Compounds	CHO IRAP ^[a]^ IC_50_ (µM)/pIC_50_	hIRAP ^[b]^ IC_50_ (µM)/pIC_50_	sIRAP ^[c]^ IC_50_ (µM)/pIC_50_	APN ^[d]^ IC_50_ (µM)/pIC_50_
**6a**	2.0/5.7 ± 0.1 (28)	1.3/5.9 ± 0.05 (2)	1.0/6.0 (1)	inactive (2)
**6a_dia1**	16/4.8 ± 0.2 (4)	6.3/5.2 (1)	-	-
**6a_dia2**	1.0/6.0 ± 0.1 (4)	1.0/6.0 (1)	-	inactive (1)
**6b**	40/4.4 ± 0.1 (8)	50/4.3 ± 0.05 (2)	-	inactive (2)

IC_50_ is defined as the molar compound concentration required to inhibit CHO cells’ IRAP activity by 50%. pIC_50_ is defined as the negative log of IC_50._ Values represent the mean ± standard deviation of best-fit values from individual test occasions (the number of independent test occasions is provided within brackets). ^[a]^ Evaluated on IRAP from Chinese Hamster Ovary Cells. ^[b]^ Evaluated on human membrane-bound IRAP overexpressed in HEK293 cells. ^[c]^ Evaluated on human recombinant soluble enzyme. ^[d]^ Evaluated on human aminopeptidase N overexpressed in HEK293 cells.

## Data Availability

Data are contained within the article and Supplementary Material.

## Supplementary Materials

The following supporting information can be downloaded at https://www.mdpi.com/article/10.3390/ijms25052516/s1. Figure S1: The proposed binding mode of compound **6a** in the APN catalytic site; Figure S2: Substrate competition experiments for a representative compound; NMR spectra.
