# Supplementary material for: Inhibition of Insulin-Regulated Aminopeptidase by Imidazo [1,5-α]pyridines—Synthesis and Evaluation
Source: Int J Mol Sci. 2024 Feb 21;25(5):2516. doi: 10.3390/ijms25052516 (PMC10931632; doi:10.3390/ijms25052516)
Supplement: Supplementary file 1 [file ijms-25-02516-s001.zip › ijms-2862975-supplementary.pdf]

Supplementary Material

Inhibition of Insulin-regulated Aminopeptidase by Imidazo[1,5-α]pyridines; Synthesis and Evaluation

Karin Engen [1], Thomas Lundbäck [2, 3], Anubha Yadav [4], Sharathna Puthiyaparambath [4], Ulrika Rosenström [1], Johan Gising [1], Annika Jenmalm-Jensen [2], Mathias Hallberg [5] and Mats Larhed [4,*]

Table of Contents

Supplementary Figure S1 2
Supplementary Figure S2 3
NMR spectra 4

Supplementary Figure S1

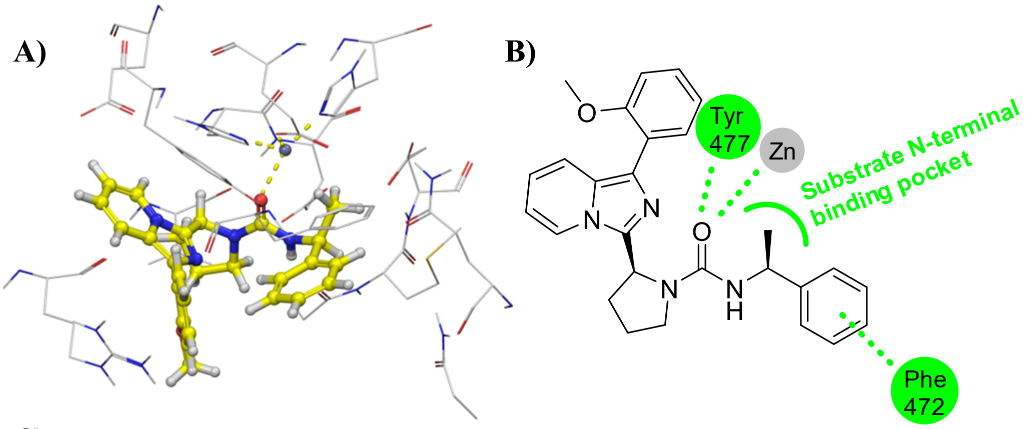

C)

```
Q9UIQ6 LCAP_HUMAN      278  SYTDESNEKKYFAATQFEPLAARSAFPCFDEPAFKATFIIKIIRDEQYTALSNMPKKSS-     336
P15144 AMPN_HUMAN      197  EYMEG-NVRKVVATTQMQAADARKSFPCFDEPAMKAEFNITLIHPKDLTALSNMLPKGPS     255
                            .* :  * :* .*:**::   **.:*********:** * *.:*: :: ******  *.

Q9UIQ6 LCAP_HUMAN      337  --VVLDDGLVQDEFSESVKMSTYLVAFIVGEMKNLS-QDVNGTLVSIYAVPEKI--GQVH     391
P15144 AMPN_HUMAN      256  TPLPEDPNWNVTEFHTTPKMSTYLLAFIVSEFDYVEKQASNGVLIRIWARPSAIAAGHGD     315
                              :  * .    **  : ******:****.*:. :. *  **.*: *:* *. *  *: .

Q9UIQ6 LCAP_HUMAN      392  YALETTVKLLEFFQNYFEIQYPLKKLDLVAIPDFEAGAMENWGLLTFREETLLYDSNTSS     451
P15144 AMPN_HUMAN      316  YALNVTGPILNFFAGHYDTPYPLPKSDQIGLPDFNAGAMENWGLVTYRENSLLFDPLSSS     375
                            ***:.*  :*:** .::  *** * * :.:***:*******:*:**::**:*  :**

Q9UIQ6 LCAP_HUMAN      452  MADRKLVTKIIAHELAHQWFGNLVTMKWWNDLWLNEGFATFMEYFSLEKIFKELSSY-ED     510
P15144 AMPN_HUMAN      376  SSNKERVVTVIAHELAHQWFGNLVTIEWWNDLWLNEGFASYVEYLGADYAEPTWNLKDLM     435
                             :::: *..:*****************:****************::**:. :      .

Q9UIQ6 LCAP_HUMAN      511  FLDARFKTMKKDSLNSSHPISSS---VQSSEQIEEMFDSLSYTKGSSLLLMLKTYLSEDV     567
P15144 AMPN_HUMAN      436  VLNDVYRVMAVDALASSHPLSTPASEINTPAQISELFDAISYSKGASVLRMLSSFLSEDV     495
                            .*:  ::.*  *:* ****:*:     :::  **.*:**::**:**:*:* **.::*****

Q9UIQ6 LCAP_HUMAN      568  FQHAVVLYLHNHSYASIQSDDLWDSFNEVTNQ-----TLDVKRMMKTWTLQKGFPLVTVQ     622
P15144 AMPN_HUMAN      496  FKQGLASYLHTFAYQNTIYLNLWDHLQEAVNNRSIQLPTTVRDIMNRWTLQMGFPVITVD     555
                            *::.:. ***..:* .    :*** ::*..*:         *: :*: **** ***::**:

Q9UIQ6 LCAP_HUMAN      623  KKGKELFIQQERFFLNMK-PEIQPSDTSYLWHIPLSYVTEGRNYSKYQSVSLLDKKSGVI     681
P15144 AMPN_HUMAN      556  TSTGTL--SQEHFLLDPDSNVTRPSEFNYVWIVPITSIRDGRQQQDYWLIDVRAQN--DL     611
                            ..   *  .**:*:*: .     :**: .*:* :*:: : :**: ..*  :.:  ::   :
```

Phe544, Tyr549 (IRAP); Phe472, Tyr477 (APN)

Figure S1. A) The proposed binding mode of compound **6a** in the APN catalytic site (APN PDB ID: 4FYT). B) Schematic key interactions of compound **6a** (with defined *S*-stereochemistry at the C2 position of the pyrrolidine) and the catalytic site. The molecular docking suggested that **6a** could obtain a substrate-like binding mode with key interactions such as zinc coordination and hydrogen bond interaction with Tyr477 as well as pi-stacking with Phe472. C) Standard amino acid sequence analysis of IRAP (top) and APN (bottom) using Clustal Omega web server at EBI (default parameters) shows 28.7% sequence similarity, with high similarity in the catalytic site. There are no differences located in the proposed binding site (**blue**). Further, the zinc binding motif and GAMEN loop residues are also conserved as shown in **red**. We thereby postulated that **6a** could display an almost identical binding pose in IRAP. However, this hypothesis was later challenged due to high selectivity for IRAP over APN, see manuscript.

Supplementary Figure S2

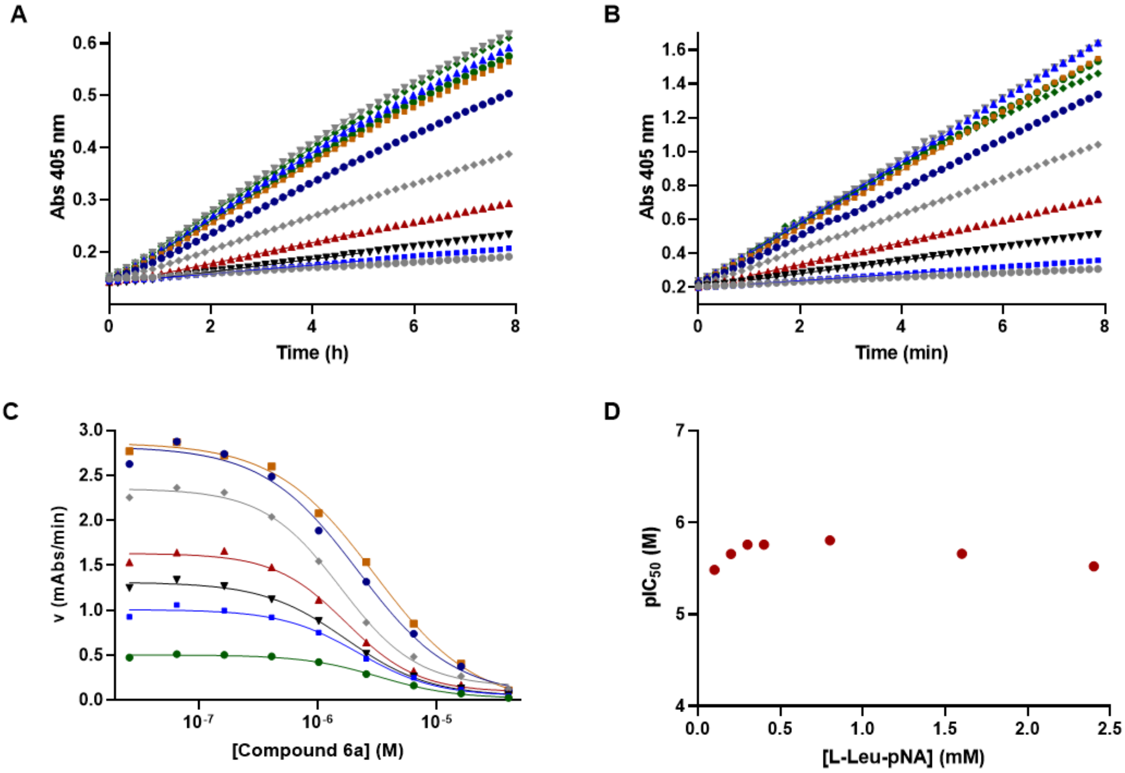

Figure S2. Substrate competition experiments for a representative compound (see also Fig. 2 in the main manuscript). A) The absorbance at 405 nm measured as a function of time at a substrate concentration below K_m (0.2 mM; average and standard deviation of four technical replicates). Data were obtained in the presence of increasing concentrations of compound **6a**; 0 nM (◆), 27 nM (●), 66 nM (▼), 0.16 µM (▲), 0.41 µM (■), 1.0 µM (●), 2.6 µM (◆), 6.4 µM (▲), 16 µM (▼), 40 µM (■) and 100 µM (●). Solid lines represent best fits to a straight line in the 1-5 h interval with regression coefficient r^2 values ranging from 0.977-1.00. B) The corresponding data at 2.4 mM substrate, *i.e.* well above K_m, with r^2 values ranging from 0.995-1.00. The slope obtained in the presence of 100 µM of compound was used to define maximal IRAP inhibition, *i.e.* this slope was subtracted to obtain IRAP reaction rates at lower compound concentrations. C) Concentration response curves for **6a** at the seven different substrate concentrations from 0.1 (●) to 2.4 mM (■). Solid lines represent best-fits to the four-parameter model in GraphPad with variable Hill slope. D) pIC_{50} values (negative logarithm of best-fit IC_{50} values) at the different substrate concentrations.

NMR spectra

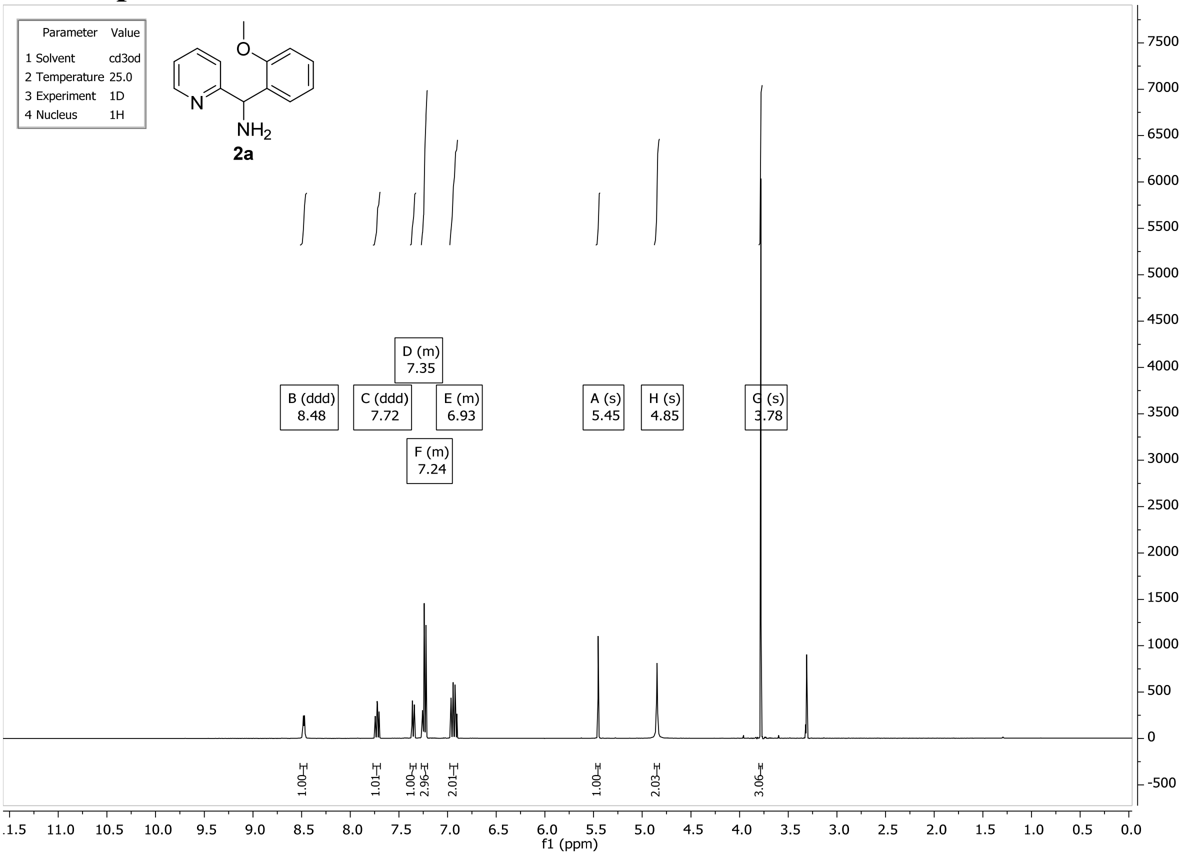

Parameter Value
1 Solvent cd3od
2 Temperature 25.0
3 Experiment 1D
4 Nucleus 1H
NH2
2a
D (m) 7.35
B (ddd) 8.48
C (ddd) 7.72
E (m) 6.93
A (s) 5.45
H (s) 4.85
G (s) 3.78
F (m) 7.24
f1 (ppm)

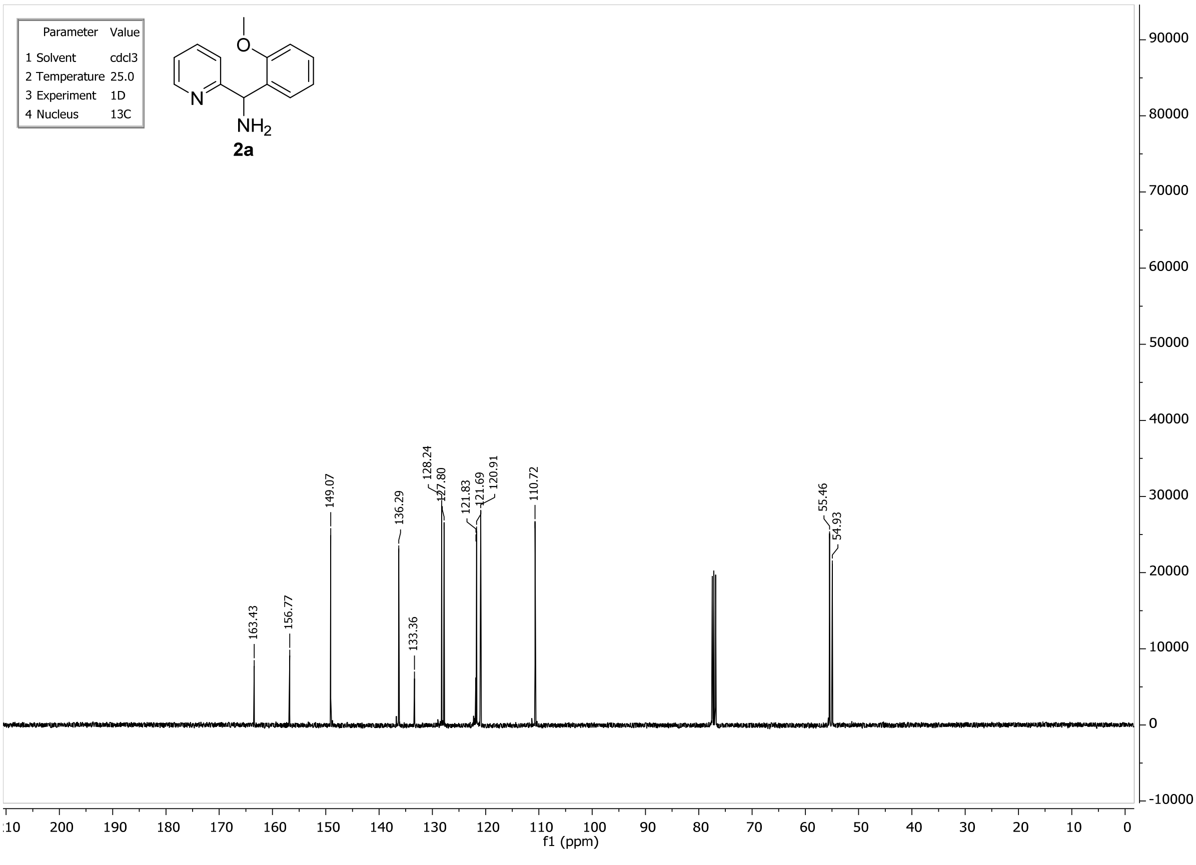

Parameter Value
1 Solvent cdcl3
2 Temperature 25.0
3 Experiment 1D
4 Nucleus 13C
NH2
2a
163.43
156.77
149.07
136.29
133.36
128.24
127.80
121.83
121.69
120.91
110.72
55.46
54.93
f1 (ppm)

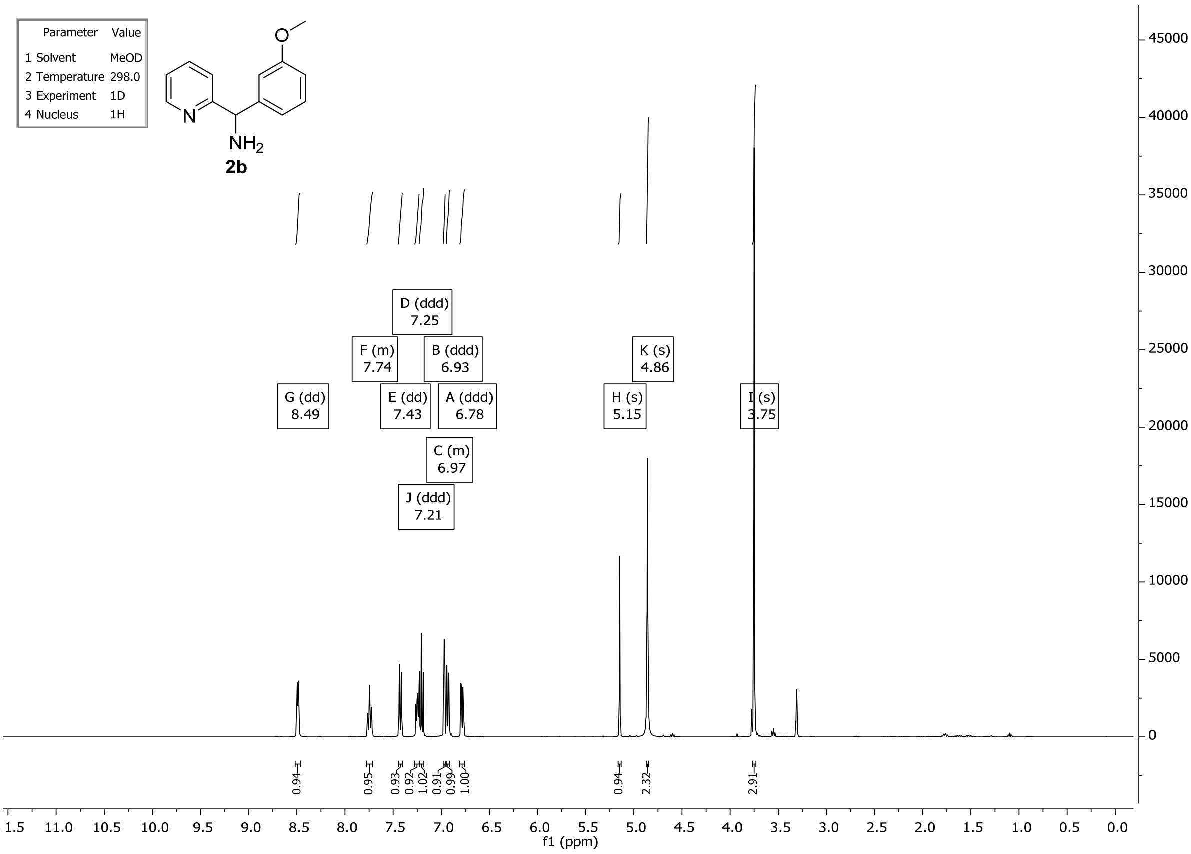
Parameter Value
1 Solvent MeOD
2 Temperature 298.0
3 Experiment 1D
4 Nucleus 1H
NH2
2b
G (dd) 8.49
F (m) 7.74
E (dd) 7.43
D (ddd) 7.25
J (ddd) 7.21
C (m) 6.97
B (ddd) 6.93
A (ddd) 6.78
H (s) 5.15
K (s) 4.86
I (s) 3.75
f1 (ppm)

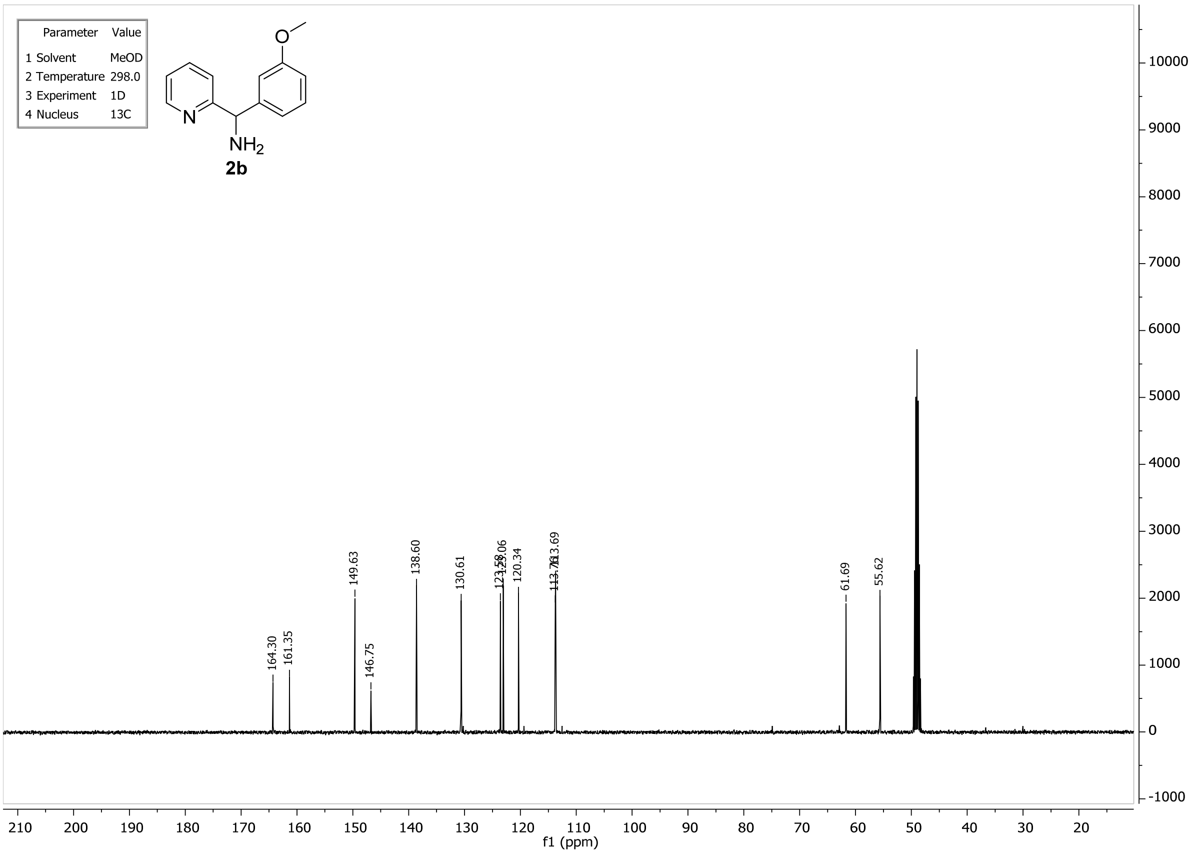
Parameter Value
1 Solvent MeOD
2 Temperature 298.0
3 Experiment 1D
4 Nucleus 13C
NH2
2b
164.30
161.35
149.63
146.75
138.60
130.61
120.34
61.69
55.62
f1 (ppm)

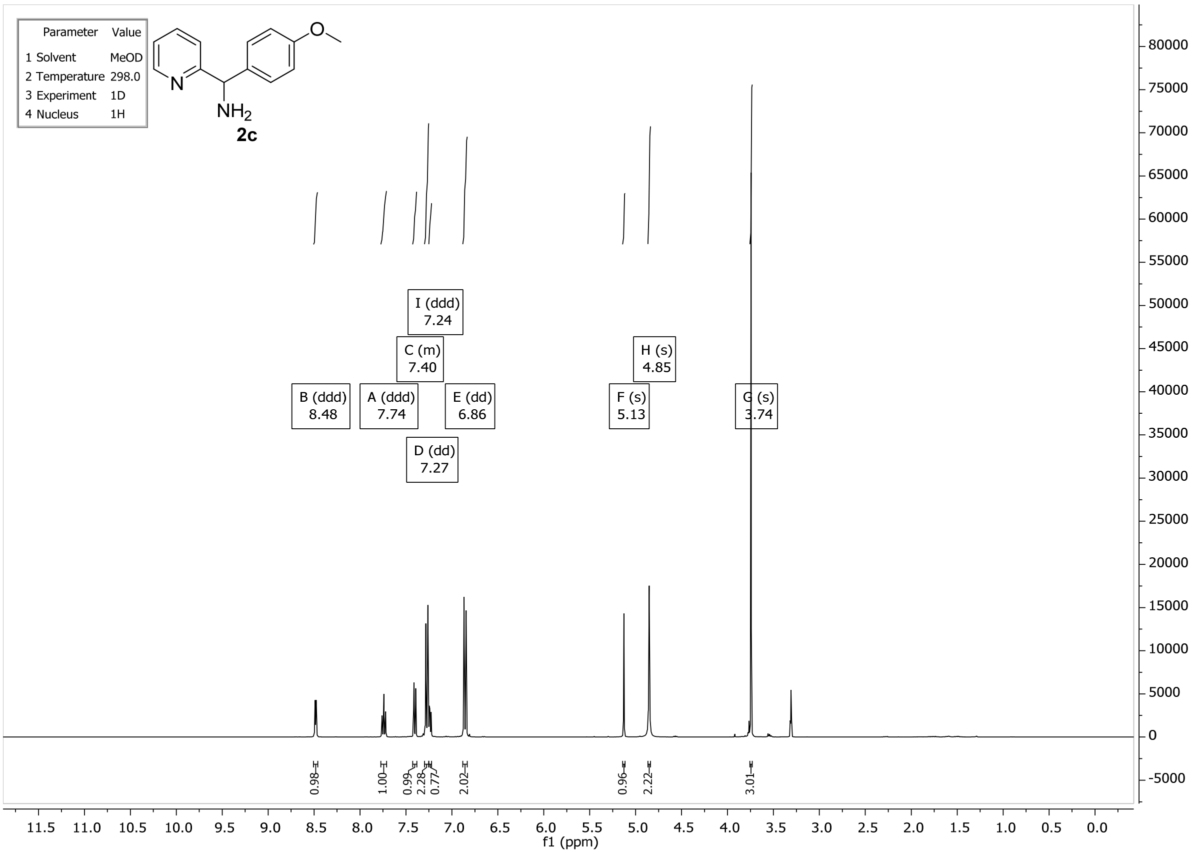

Parameter Value
1 Solvent MeOD
2 Temperature 298.0
3 Experiment 1D
4 Nucleus 1H
NH2
2c
I (ddd) 7.24
C (m) 7.40
H (s) 4.85
B (ddd) 8.48
A (ddd) 7.74
E (dd) 6.86
F (s) 5.13
G (s) 3.74
D (dd) 7.27
0.98
1.00
0.99
2.28
0.77
2.02
0.96
2.22
3.01
f1 (ppm)

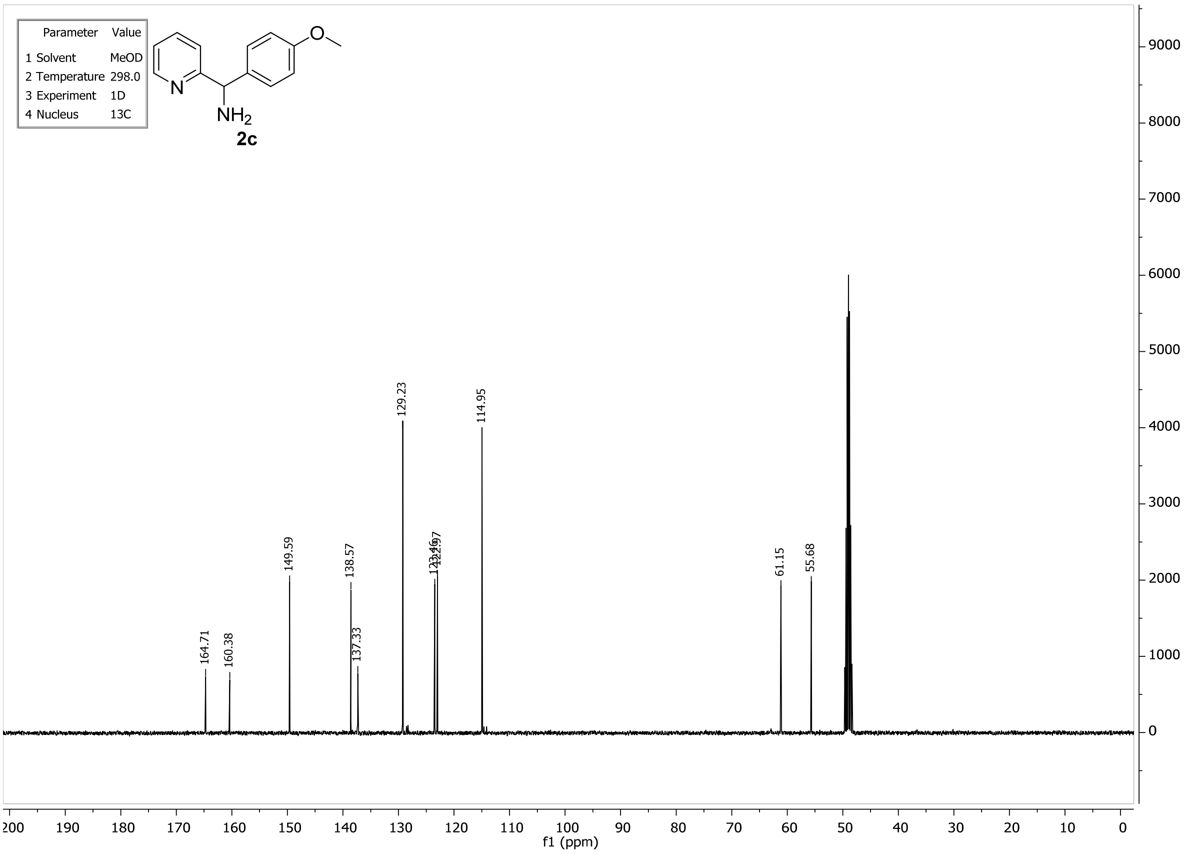

Parameter Value
1 Solvent MeOD
2 Temperature 298.0
3 Experiment 1D
4 Nucleus 13C
NH2
2c
164.71
160.38
149.59
138.57
137.33
129.23
114.95
61.15
55.68
f1 (ppm)

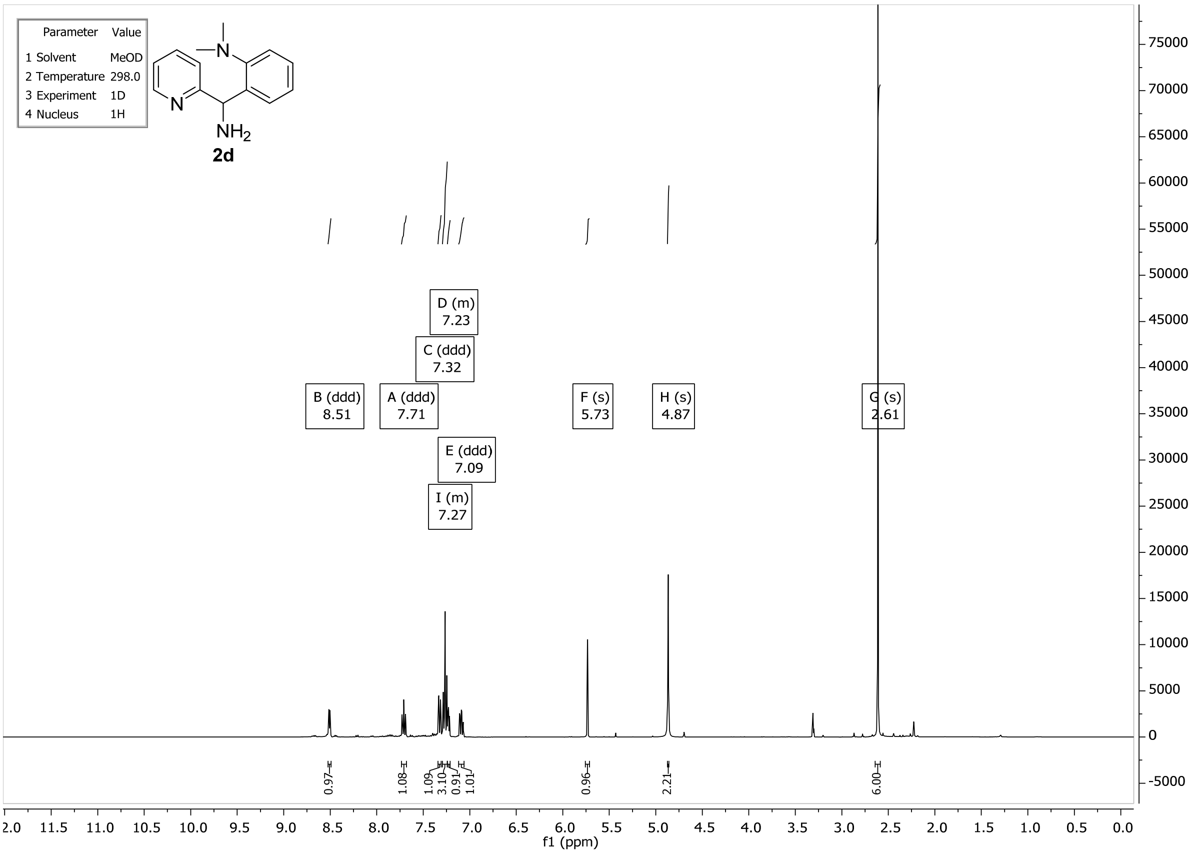

Parameter Value
1 Solvent MeOD
2 Temperature 298.0
3 Experiment 1D
4 Nucleus 1H
NH2
2d
B (ddd) 8.51
A (ddd) 7.71
C (ddd) 7.32
D (m) 7.23
E (ddd) 7.09
I (m) 7.27
F (s) 5.73
H (s) 4.87
G (s) 2.61
0.97
1.08
1.09
3.10
0.91
1.01
0.96
2.21
6.00
f1 (ppm)

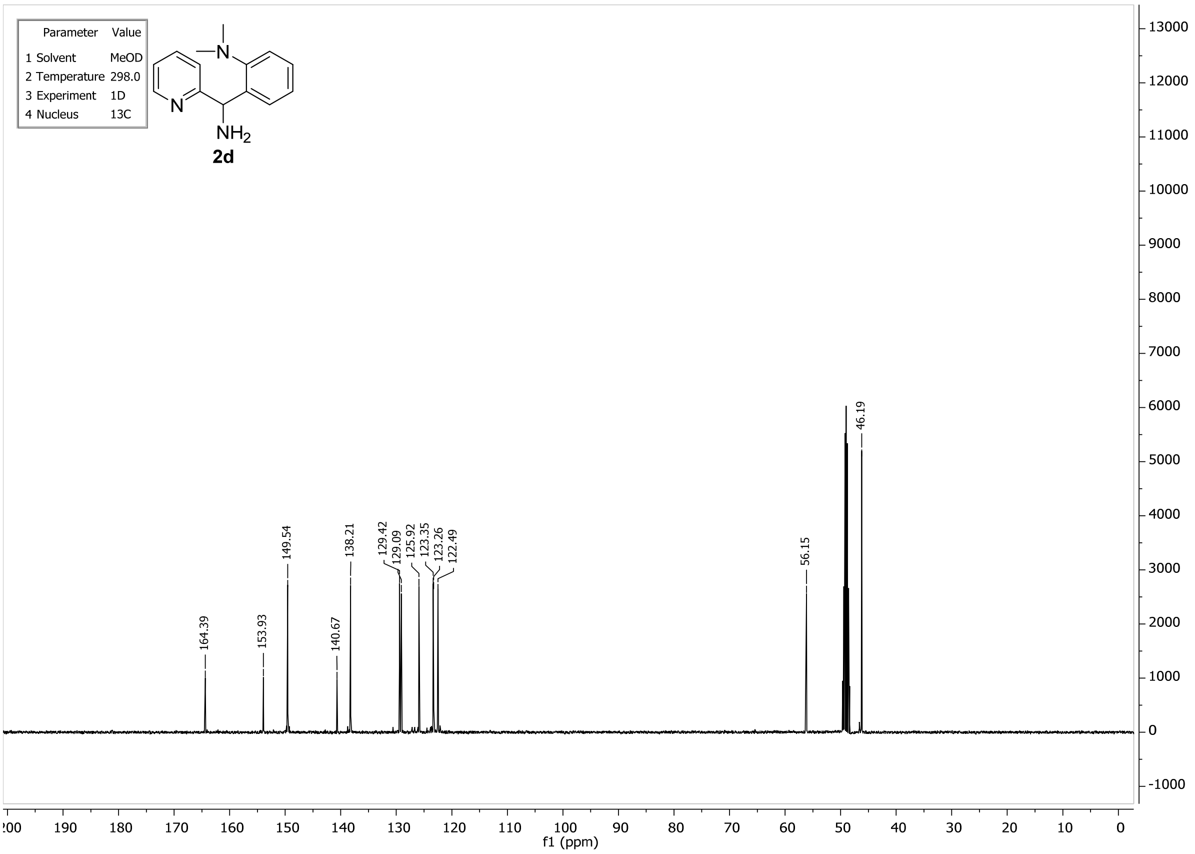

Parameter Value
1 Solvent MeOD
2 Temperature 298.0
3 Experiment 1D
4 Nucleus 13C
NH2
2d
164.39
153.93
149.54
140.67
138.21
129.42
129.09
125.92
123.35
123.26
122.49
56.15
46.19
f1 (ppm)

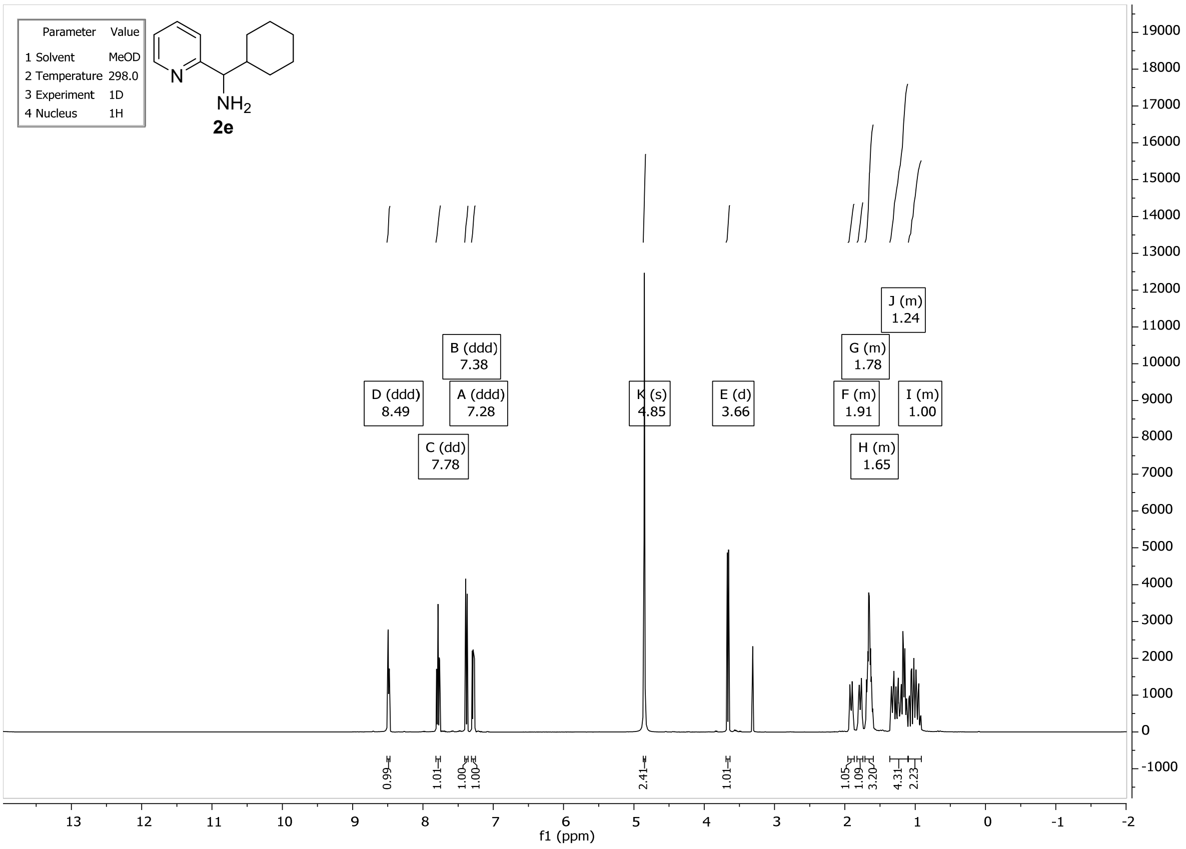

Parameter Value
1 Solvent MeOD
2 Temperature 298.0
3 Experiment 1D
4 Nucleus 1H
NH2
2e
D (ddd) 8.49
B (ddd) 7.38
A (ddd) 7.28
C (dd) 7.78
K (s) 4.85
E (d) 3.66
J (m) 1.24
G (m) 1.78
F (m) 1.91
I (m) 1.00
H (m) 1.65
0.99
1.01
1.00
1.00
2.41
1.01
1.05
1.09
3.20
4.31
2.23
f1 (ppm)

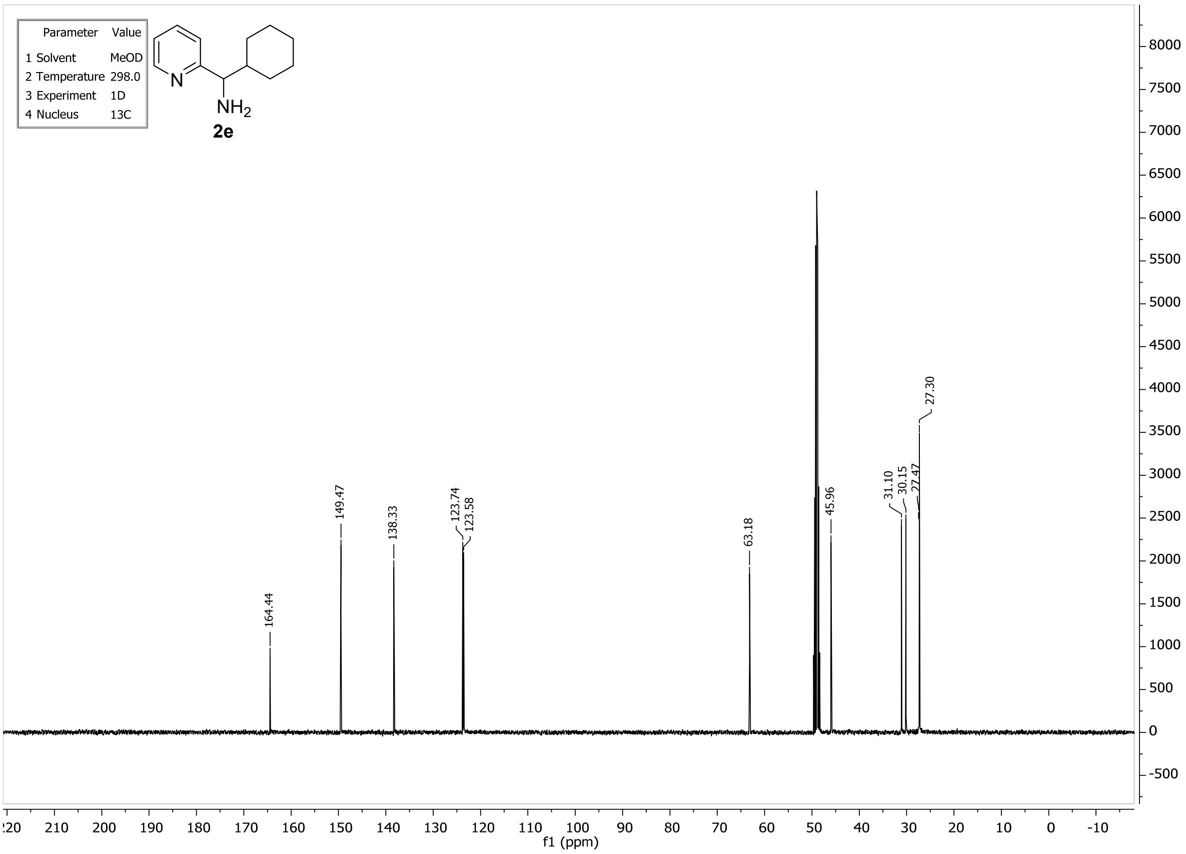

Parameter Value
1 Solvent MeOD
2 Temperature 298.0
3 Experiment 1D
4 Nucleus 13C
NH2
2e
164.44
149.47
138.33
123.74
123.58
63.18
45.96
31.10
30.15
27.47
27.30
f1 (ppm)

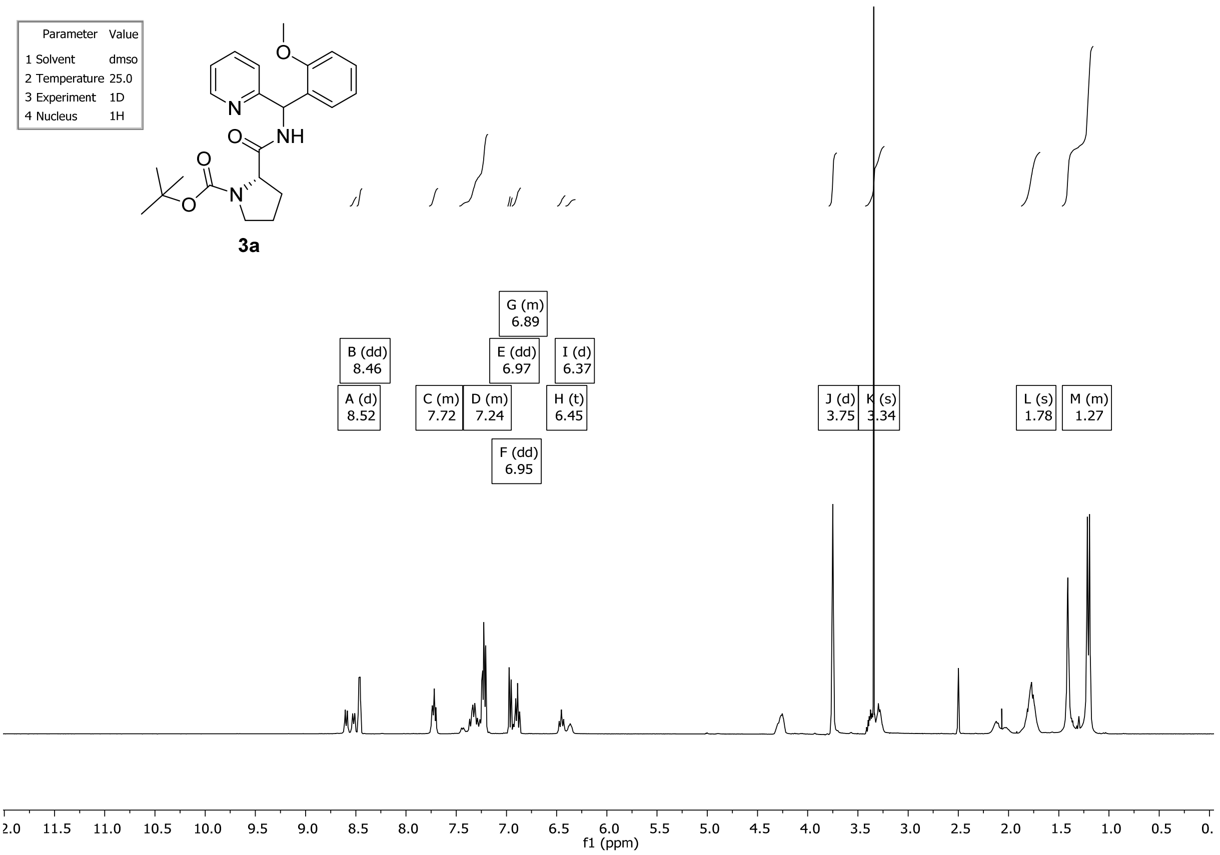
Parameter Value
1 Solvent dmso
2 Temperature 25.0
3 Experiment 1D
4 Nucleus 1H
A (d) 8.52
B (dd) 8.46
C (m) 7.72
D (m) 7.24
E (dd) 6.97
F (dd) 6.95
G (m) 6.89
H (t) 6.45
I (d) 6.37
J (d) 3.75
K (s) 3.34
L (s) 1.78
M (m) 1.27
f1 (ppm)

3a

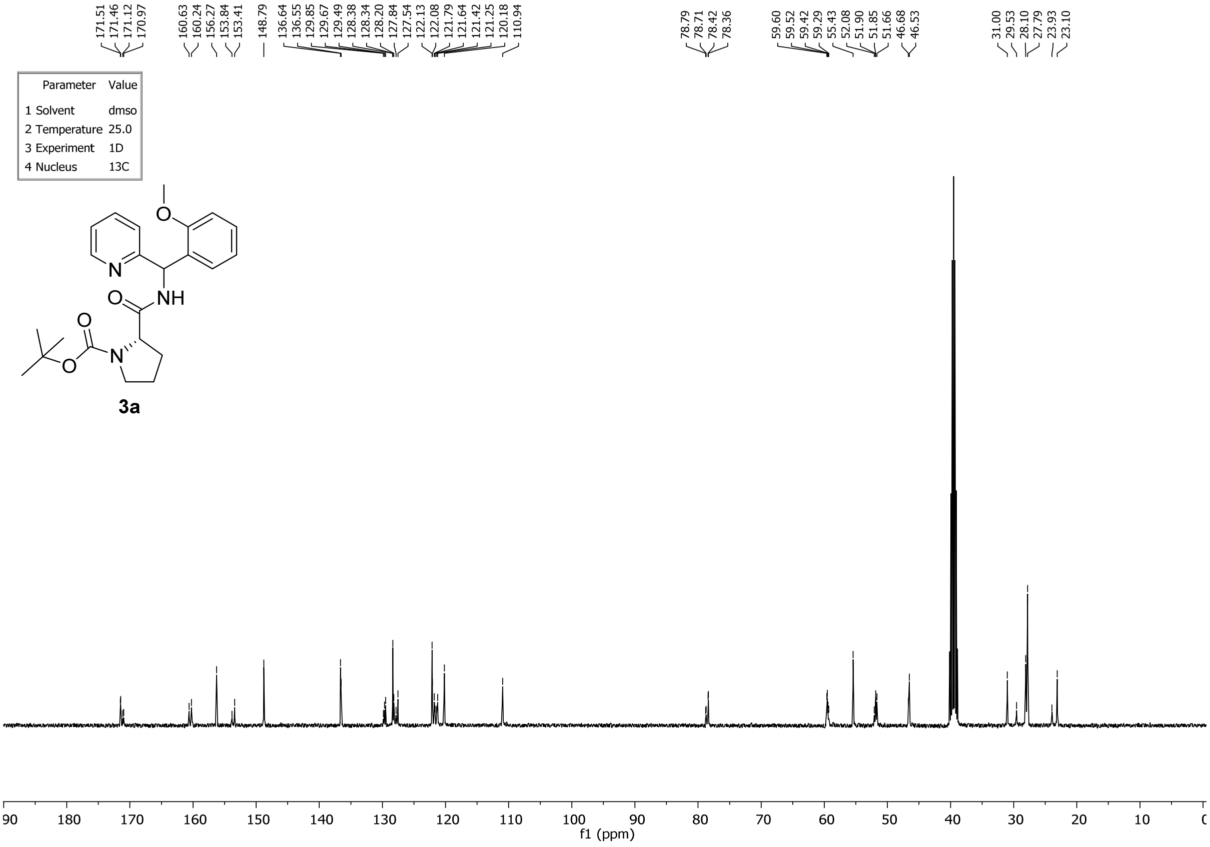
171.51 171.46 171.12 170.97 160.63 160.24 156.27 153.84 153.41 148.79 136.64 136.55 129.85 129.67 129.49 128.38 128.34 128.20 127.84 127.54 122.13 122.08 121.79 121.64 121.42 121.25 120.18 110.94 78.79 78.71 78.42 78.36 59.60 59.52 59.42 59.29 55.43 52.08 51.90 51.85 51.66 46.68 46.53 31.00 29.53 28.10 27.79 23.93 23.10
Parameter Value
1 Solvent dmso
2 Temperature 25.0
3 Experiment 1D
4 Nucleus 13C
f1 (ppm)

3a

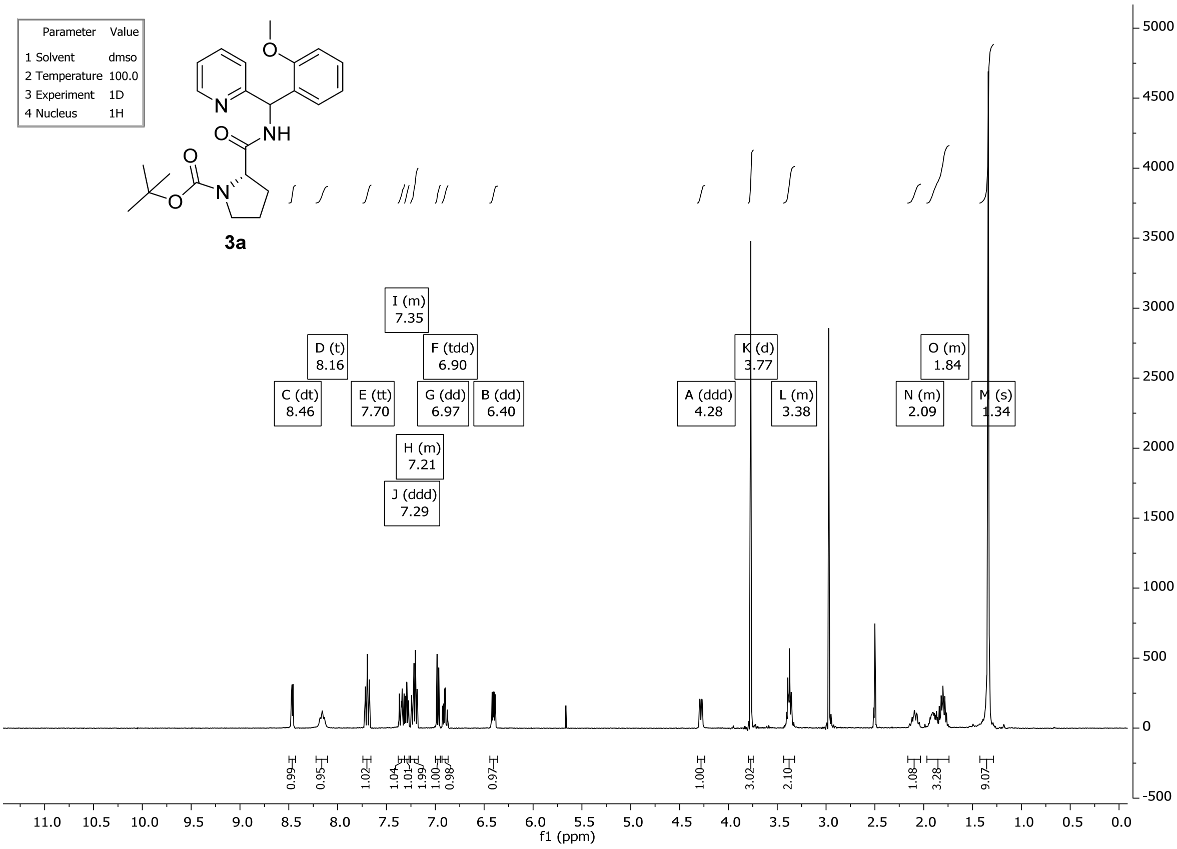
Parameter Value
1 Solvent dmso
2 Temperature 100.0
3 Experiment 1D
4 Nucleus 1H
3a
C (dt) 8.46
D (t) 8.16
E (tt) 7.70
I (m) 7.35
J (ddd) 7.29
H (m) 7.21
G (dd) 6.97
F (tdd) 6.90
B (dd) 6.40
A (ddd) 4.28
K (d) 3.77
L (m) 3.38
N (m) 2.09
O (m) 1.84
M (s) 1.34
f1 (ppm)

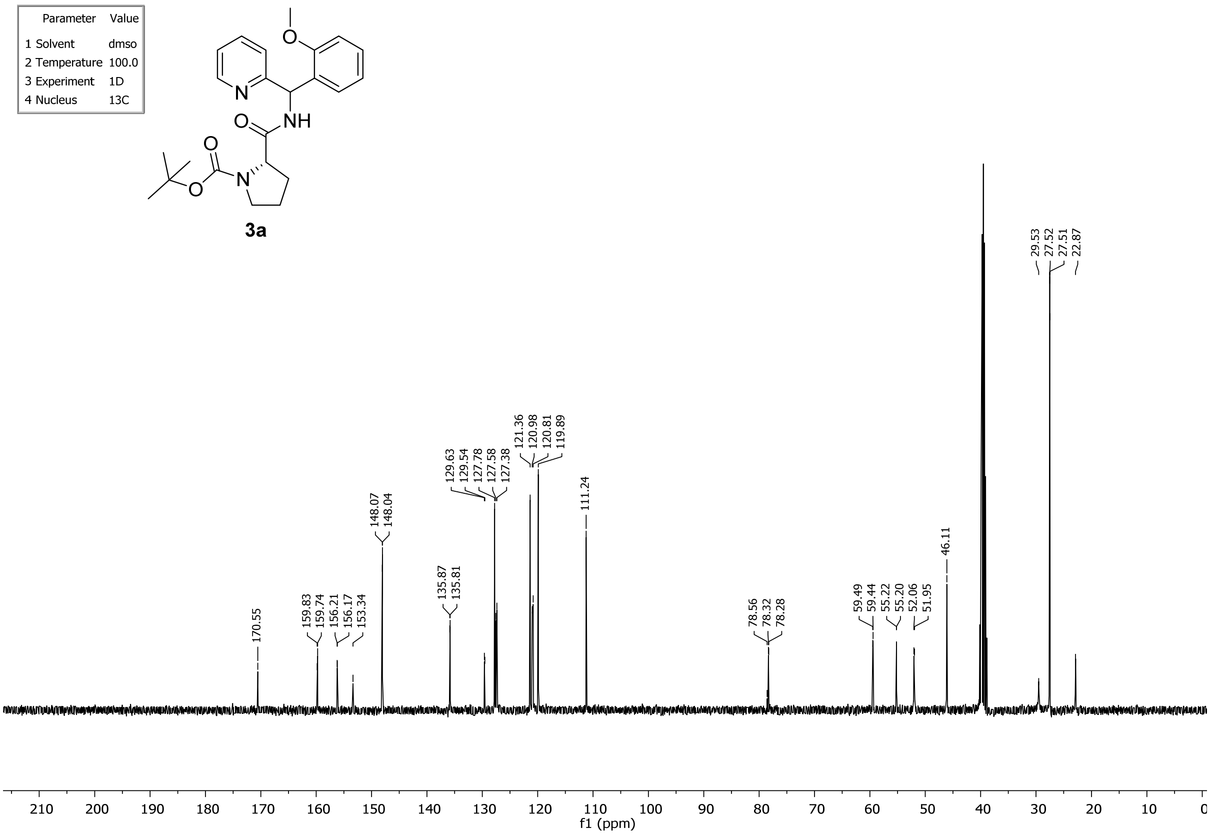
Parameter Value
1 Solvent dmso
2 Temperature 100.0
3 Experiment 1D
4 Nucleus 13C
3a
f1 (ppm)

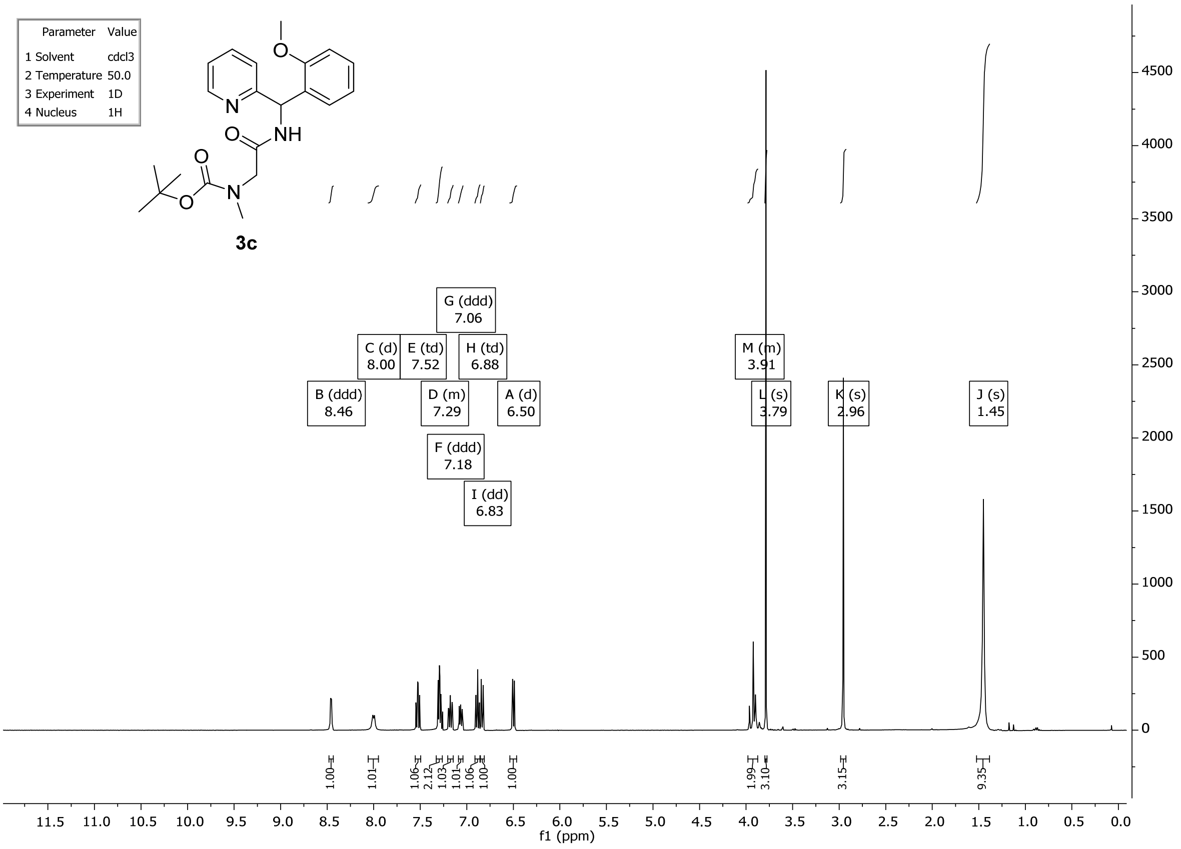

Parameter Value
1 Solvent cdcl3
2 Temperature 50.0
3 Experiment 1D
4 Nucleus 1H
3c
G (ddd) 7.06
C (d) 8.00
E (td) 7.52
H (td) 6.88
M (m) 3.91
B (ddd) 8.46
D (m) 7.29
A (d) 6.50
L (s) 3.79
K (s) 2.96
J (s) 1.45
F (ddd) 7.18
I (dd) 6.83
1.00
1.01
1.06
2.12
1.03
1.01
1.06
1.00
1.00
1.99
3.10
3.15
9.35
f1 (ppm)

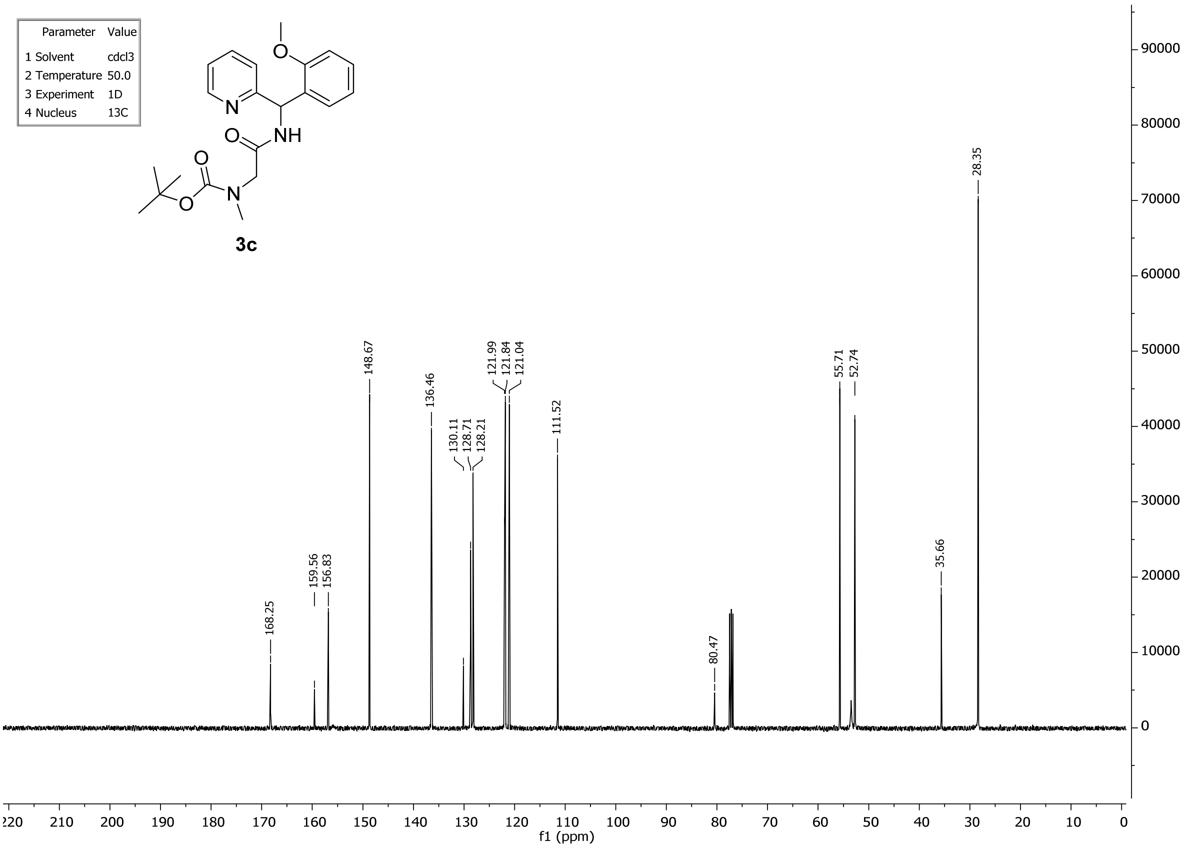

Parameter Value
1 Solvent cdcl3
2 Temperature 50.0
3 Experiment 1D
4 Nucleus 13C
3c
168.25
159.56
156.83
148.67
136.46
130.11
128.71
128.21
121.99
121.84
121.04
111.52
80.47
55.71
52.74
35.66
28.35
f1 (ppm)

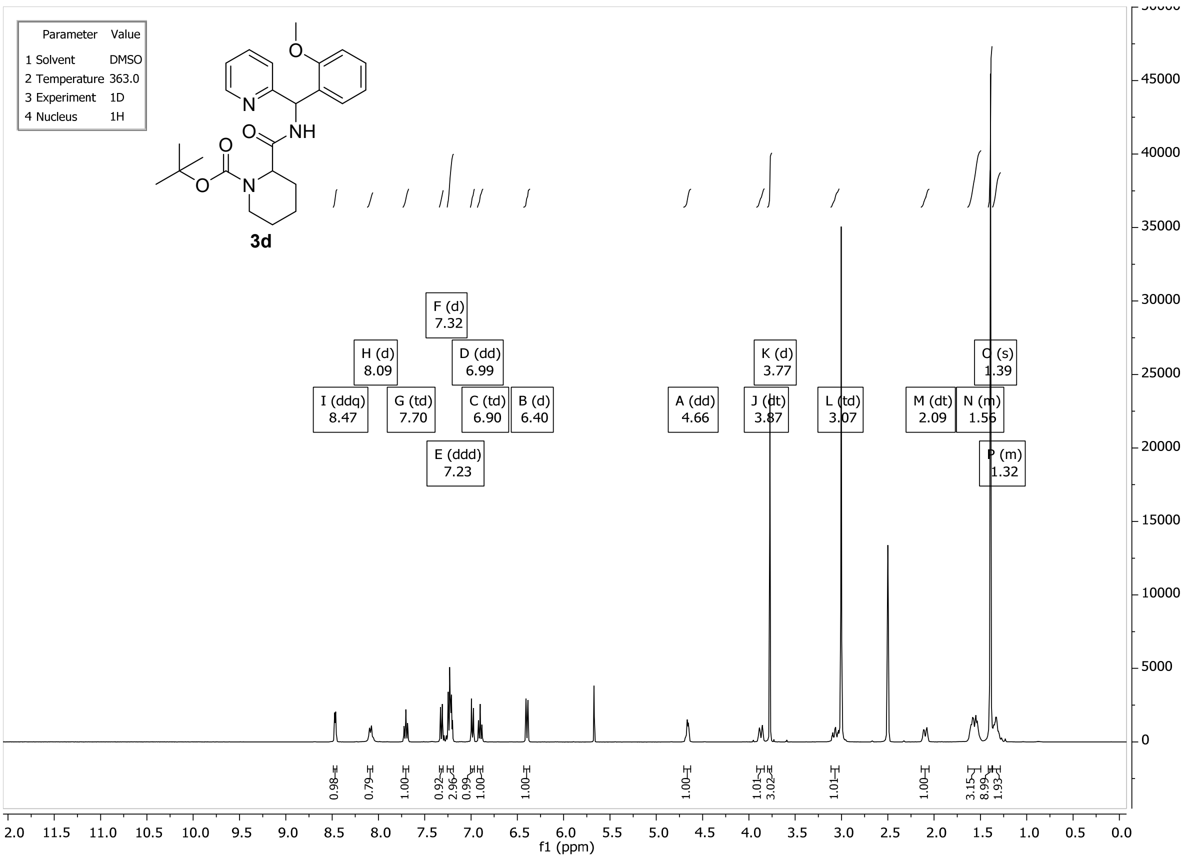
Parameter Value
1 Solvent DMSO
2 Temperature 363.0
3 Experiment 1D
4 Nucleus 1H
3d
I (ddq) 8.47
H (d) 8.09
G (td) 7.70
F (d) 7.32
E (ddd) 7.23
D (dd) 6.99
C (td) 6.90
B (d) 6.40
A (dd) 4.66
J (dt) 3.87
K (d) 3.77
L (td) 3.07
M (dt) 2.09
N (m) 1.56
O (s) 1.39
P (m) 1.32
0.98
0.79
1.00
0.92
2.96
0.99
1.00
1.00
1.00
1.01
3.02
1.01
1.00
3.15
8.99
1.93
f1 (ppm)

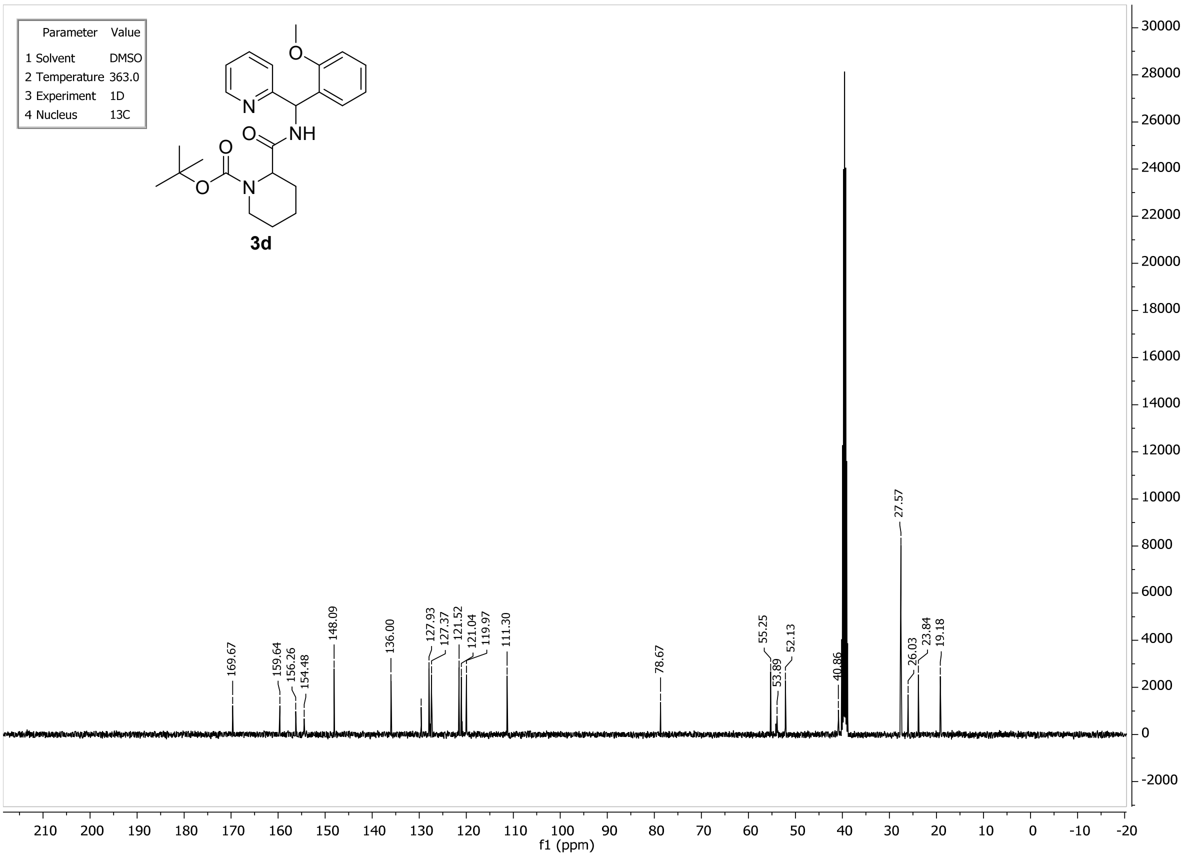
Parameter Value
1 Solvent DMSO
2 Temperature 363.0
3 Experiment 1D
4 Nucleus 13C
3d
169.67
159.64
156.26
154.48
148.09
136.00
127.93
127.37
121.52
121.04
119.97
111.30
78.67
55.25
53.89
52.13
40.86
27.57
26.03
23.84
19.18
f1 (ppm)

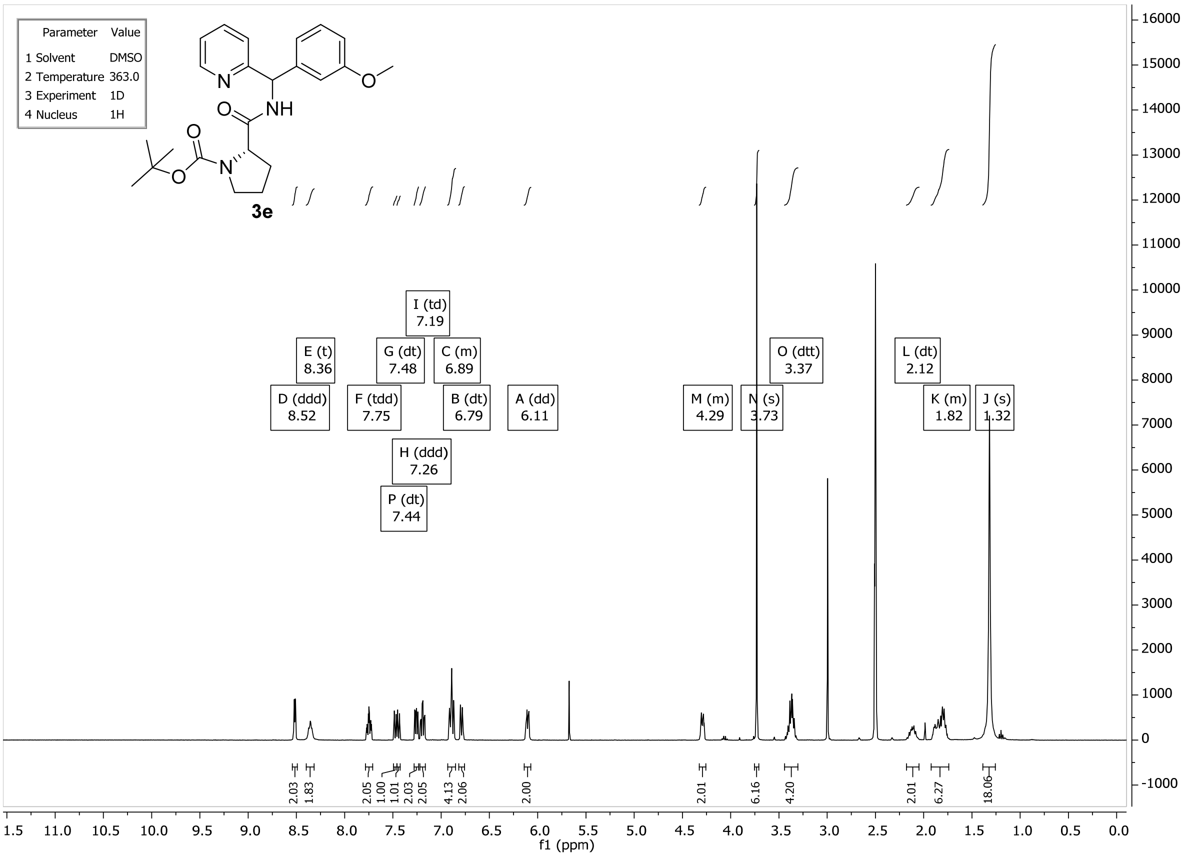
Parameter Value
1 Solvent DMSO
2 Temperature 363.0
3 Experiment 1D
4 Nucleus 1H
3e
I (td) 7.19
E (t) 8.36
G (dt) 7.48
C (m) 6.89
O (dtt) 3.37
L (dt) 2.12
D (ddd) 8.52
F (tdd) 7.75
B (dt) 6.79
A (dd) 6.11
M (m) 4.29
N (s) 3.73
K (m) 1.82
J (s) 1.32
H (ddd) 7.26
P (dt) 7.44
2.03
1.83
2.05
1.00
1.01
2.03
2.05
4.13
2.06
2.00
2.01
6.16
4.20
2.01
6.27
18.06
f1 (ppm)

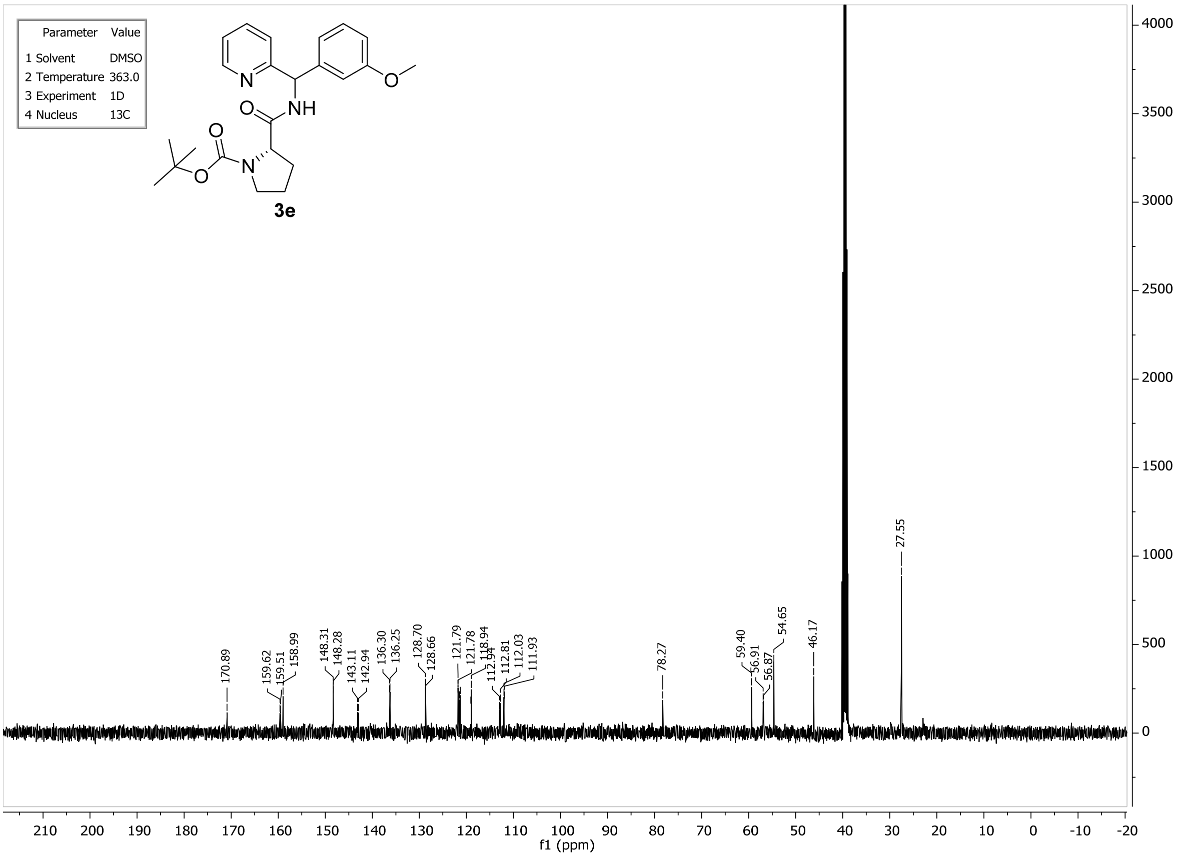
Parameter Value
1 Solvent DMSO
2 Temperature 363.0
3 Experiment 1D
4 Nucleus 13C
3e
170.89
159.62
159.51
158.99
148.31
148.28
143.11
142.94
136.30
136.25
128.70
128.66
121.79
121.78
118.94
112.94
112.81
112.03
111.93
78.27
59.40
56.91
56.87
54.65
46.17
27.55
f1 (ppm)

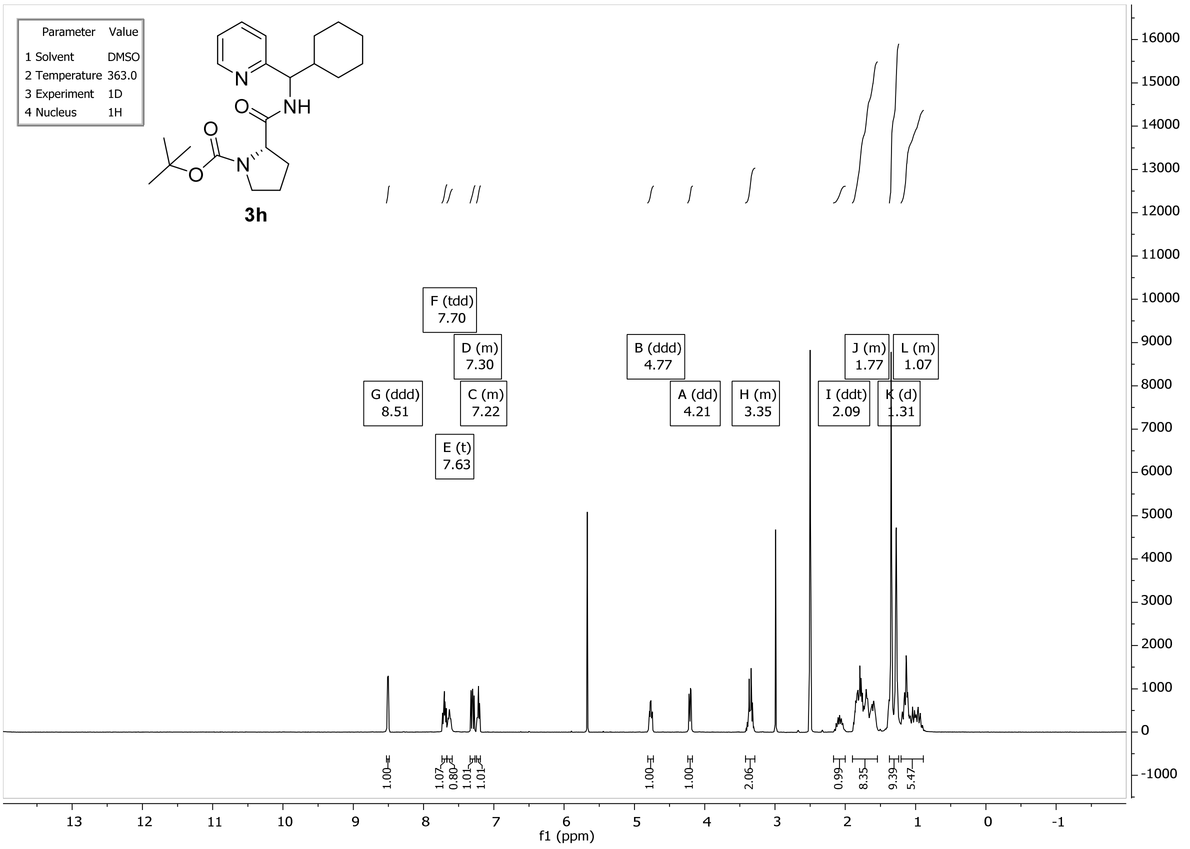
Parameter Value
1 Solvent DMSO
2 Temperature 363.0
3 Experiment 1D
4 Nucleus 1H
3h
G (ddd) 8.51
F (tdd) 7.70
E (t) 7.63
D (m) 7.30
C (m) 7.22
B (ddd) 4.77
A (dd) 4.21
H (m) 3.35
I (ddt) 2.09
J (m) 1.77
K (d) 1.31
L (m) 1.07
1.00
1.07
0.80
1.01
1.01
1.00
1.00
2.06
0.99
8.35
9.39
5.47
f1 (ppm)

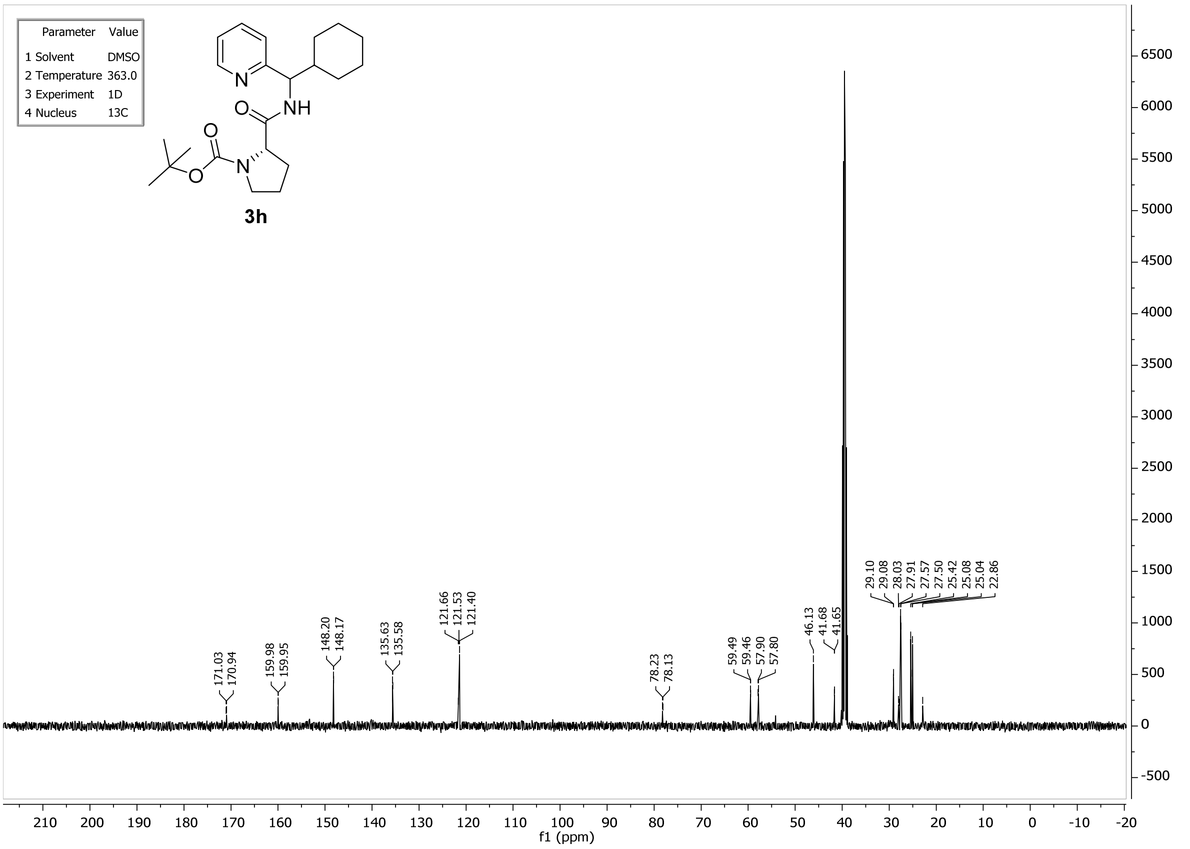
Parameter Value
1 Solvent DMSO
2 Temperature 363.0
3 Experiment 1D
4 Nucleus 13C
3h
171.03
170.94
159.98
159.95
148.20
148.17
135.63
135.58
121.66
121.53
121.40
78.23
78.13
59.49
59.46
57.90
57.80
46.13
41.68
41.65
29.10
29.08
28.03
27.91
27.57
27.50
25.42
25.08
25.04
22.86
f1 (ppm)

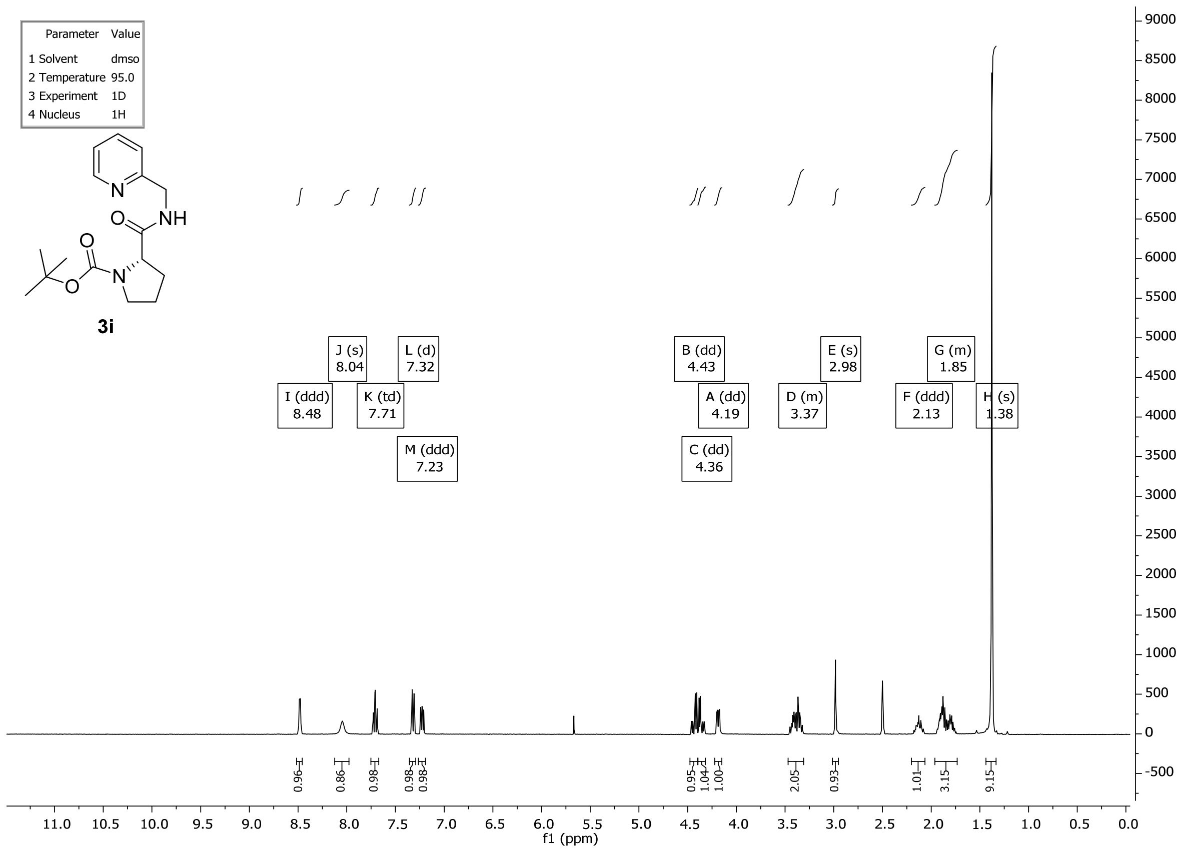
Parameter Value
1 Solvent dmso
2 Temperature 95.0
3 Experiment 1D
4 Nucleus 1H
3i
J (s) 8.04
L (d) 7.32
I (ddd) 8.48
K (td) 7.71
M (ddd) 7.23
B (dd) 4.43
A (dd) 4.19
C (dd) 4.36
D (m) 3.37
E (s) 2.98
F (ddd) 2.13
G (m) 1.85
H (s) 1.38
f1 (ppm)

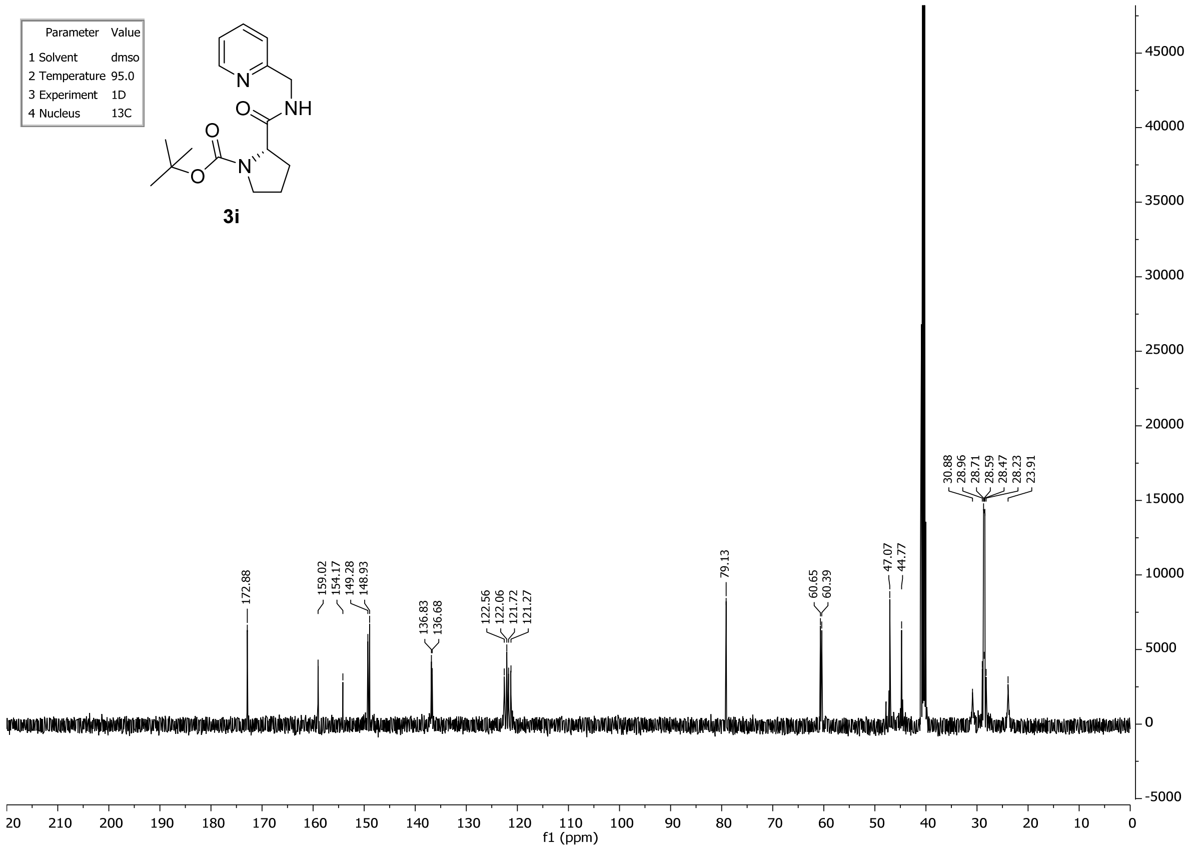
Parameter Value
1 Solvent dmso
2 Temperature 95.0
3 Experiment 1D
4 Nucleus 13C
3i
172.88
159.02
154.17
149.28
148.93
136.83
136.68
122.56
122.06
121.72
121.27
79.13
60.65
60.39
47.07
44.77
30.88
28.96
28.71
28.59
28.47
28.23
23.91
f1 (ppm)

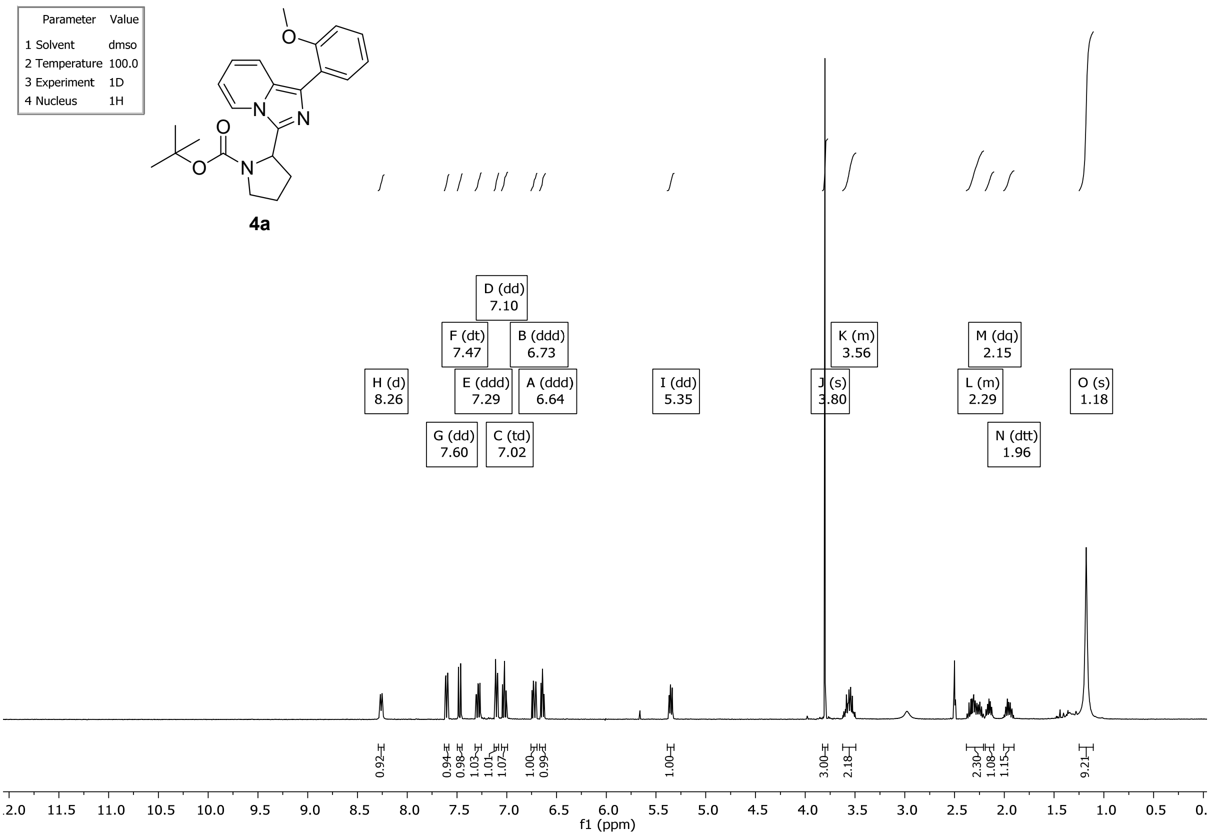
Parameter Value
1 Solvent dmso
2 Temperature 100.0
3 Experiment 1D
4 Nucleus 1H
4a
D (dd) 7.10
F (dt) 7.47
B (ddd) 6.73
K (m) 3.56
M (dq) 2.15
H (d) 8.26
E (ddd) 7.29
A (ddd) 6.64
I (dd) 5.35
J (s) 3.80
L (m) 2.29
O (s) 1.18
G (dd) 7.60
C (td) 7.02
N (dtt) 1.96
0.92 0.94 0.98 1.03 1.01 1.07 1.00 0.99 1.00 3.00 2.18 2.30 1.08 1.15 9.21
f1 (ppm)

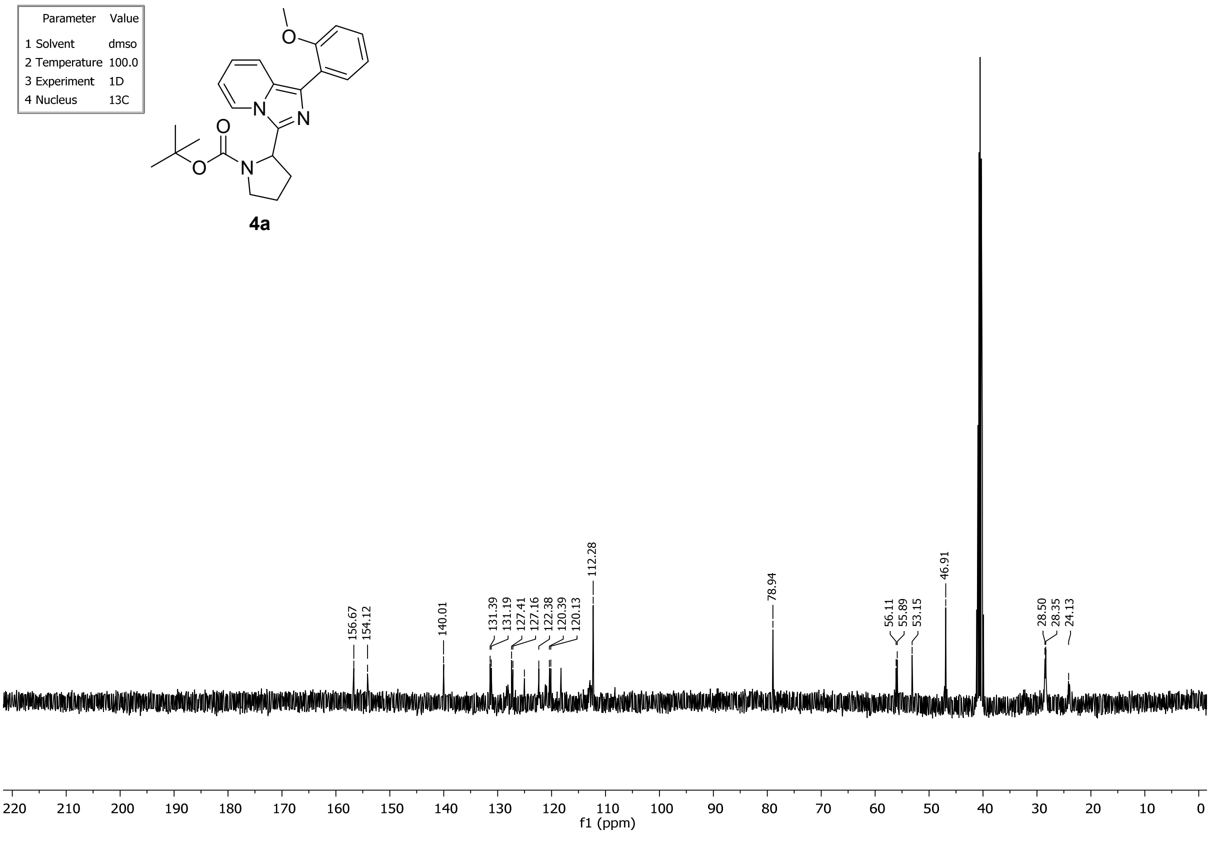
Parameter Value
1 Solvent dmso
2 Temperature 100.0
3 Experiment 1D
4 Nucleus 13C
4a
156.67 154.12 140.01 131.39 131.19 127.41 127.16 122.38 120.39 120.13 112.28 78.94 56.11 55.89 53.15 46.91 28.50 28.35 24.13
f1 (ppm)

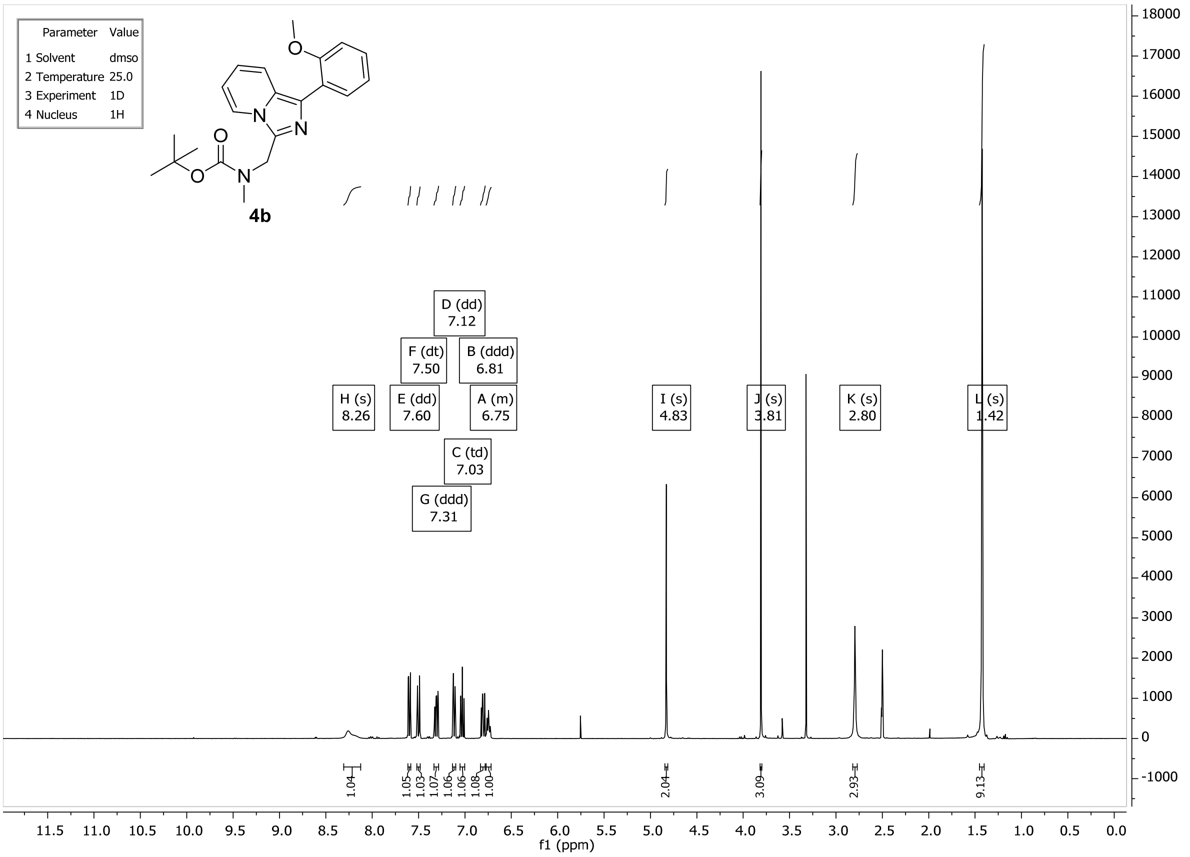
Parameter Value
1 Solvent dmso
2 Temperature 25.0
3 Experiment 1D
4 Nucleus 1H
4b
H (s) 8.26
E (dd) 7.60
F (dt) 7.50
G (ddd) 7.31
D (dd) 7.12
C (td) 7.03
B (ddd) 6.81
A (m) 6.75
I (s) 4.83
J (s) 3.81
K (s) 2.80
L (s) 1.42
1.04
1.05
1.03
1.07
1.06
1.06
1.08
1.00
2.04
3.09
2.93
9.13
f1 (ppm)

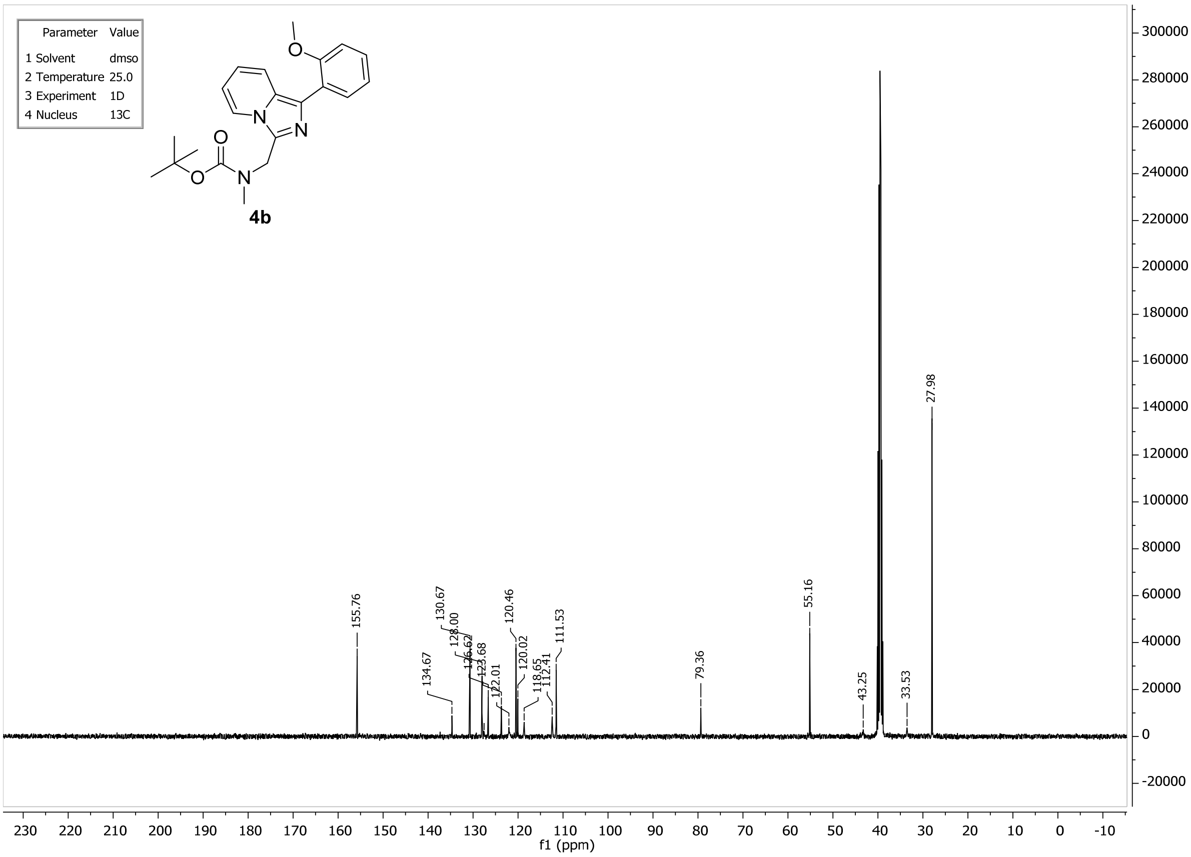
Parameter Value
1 Solvent dmso
2 Temperature 25.0
3 Experiment 1D
4 Nucleus 13C
4b
155.76
134.67
130.67
128.00
126.62
123.68
122.01
120.46
120.02
118.65
112.41
111.53
79.36
55.16
43.25
33.53
27.98
f1 (ppm)

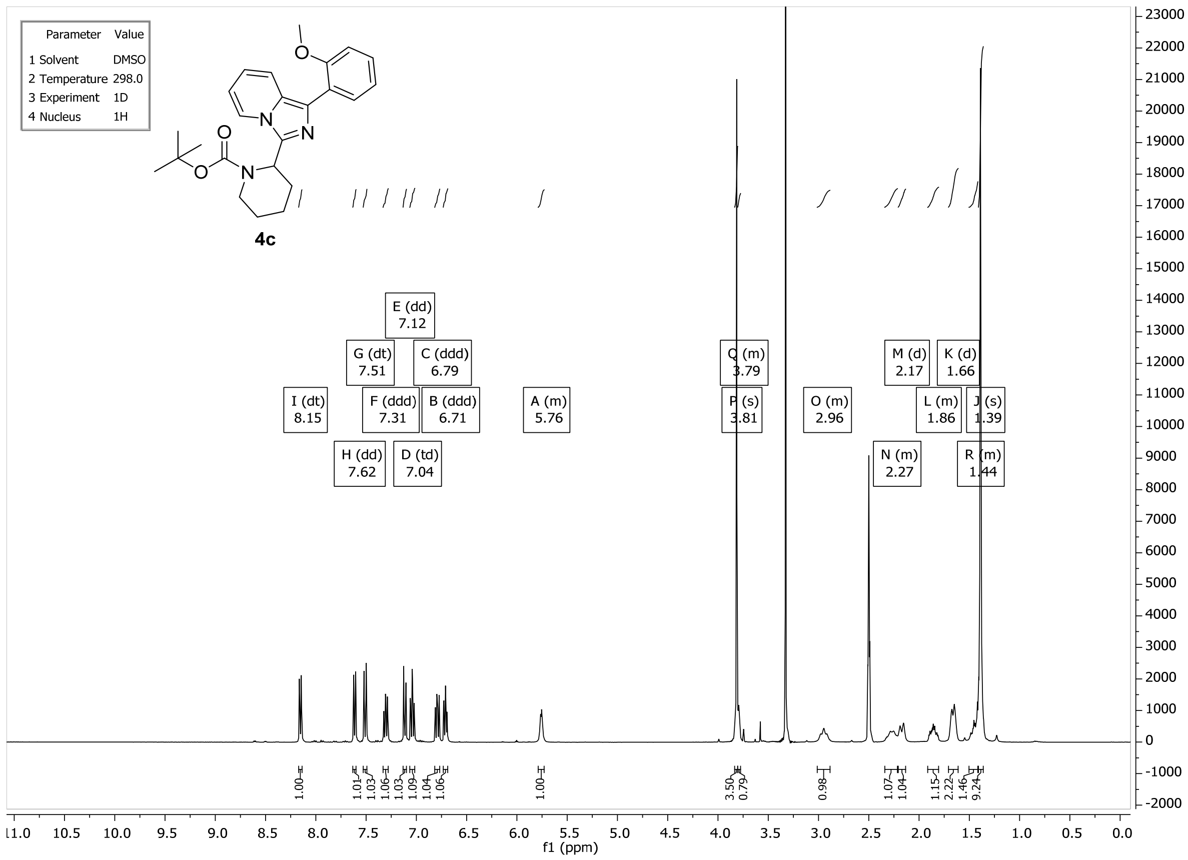
Parameter Value
1 Solvent DMSO
2 Temperature 298.0
3 Experiment 1D
4 Nucleus 1H
4c
I (dt) 8.15
H (dd) 7.62
G (dt) 7.51
F (ddd) 7.31
E (dd) 7.12
D (td) 7.04
C (ddd) 6.79
B (ddd) 6.71
A (m) 5.76
Q (m) 3.79
P (s) 3.81
O (m) 2.96
N (m) 2.27
M (d) 2.17
L (m) 1.86
K (d) 1.66
R (m) 1.44
J (s) 1.39
f1 (ppm)

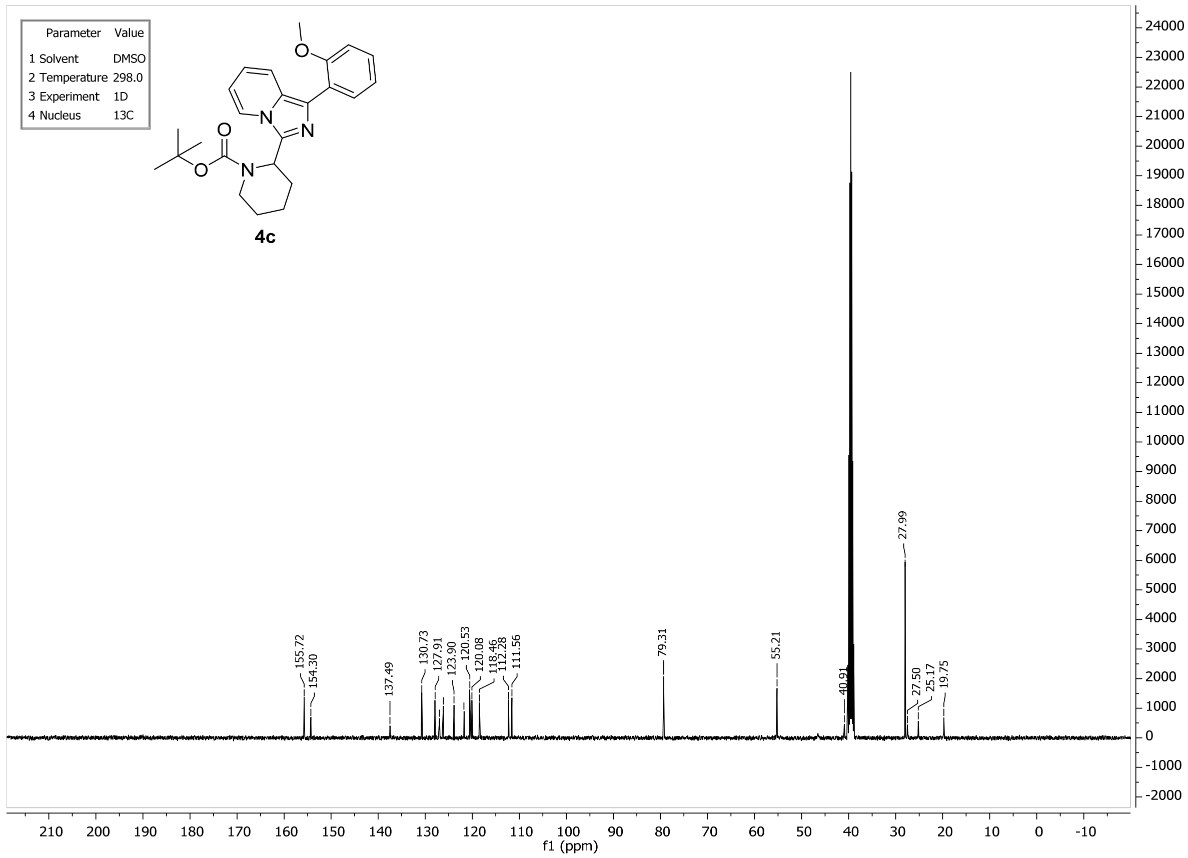
Parameter Value
1 Solvent DMSO
2 Temperature 298.0
3 Experiment 1D
4 Nucleus 13C
4c
155.72
154.30
137.49
130.73
127.91
123.90
120.53
120.08
118.46
112.28
111.56
79.31
55.21
40.91
27.99
27.50
25.17
19.75
f1 (ppm)

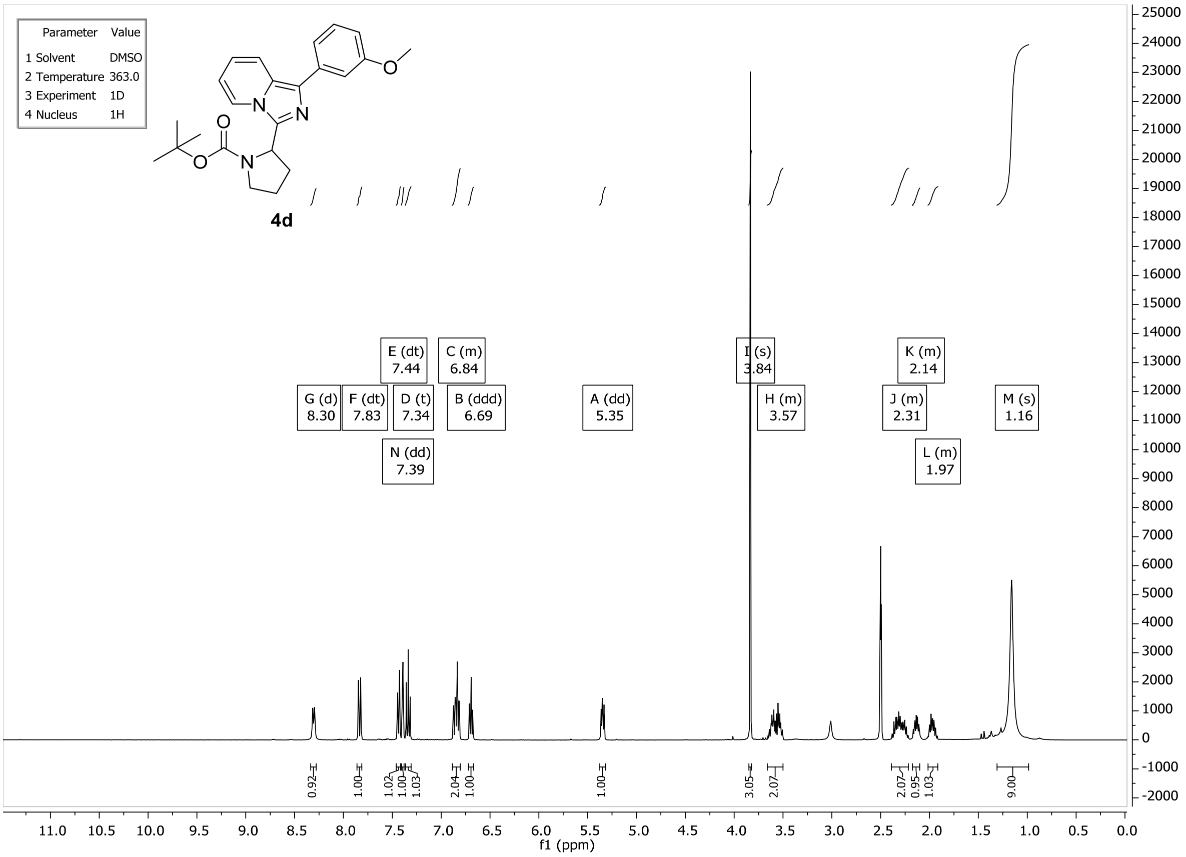
Parameter Value
1 Solvent DMSO
2 Temperature 363.0
3 Experiment 1D
4 Nucleus 1H
4d
G (d) 8.30
F (dt) 7.83
E (dt) 7.44
D (t) 7.34
N (dd) 7.39
C (m) 6.84
B (ddd) 6.69
A (dd) 5.35
I (s) 3.84
H (m) 3.57
K (m) 2.14
J (m) 2.31
L (m) 1.97
M (s) 1.16
f1 (ppm)

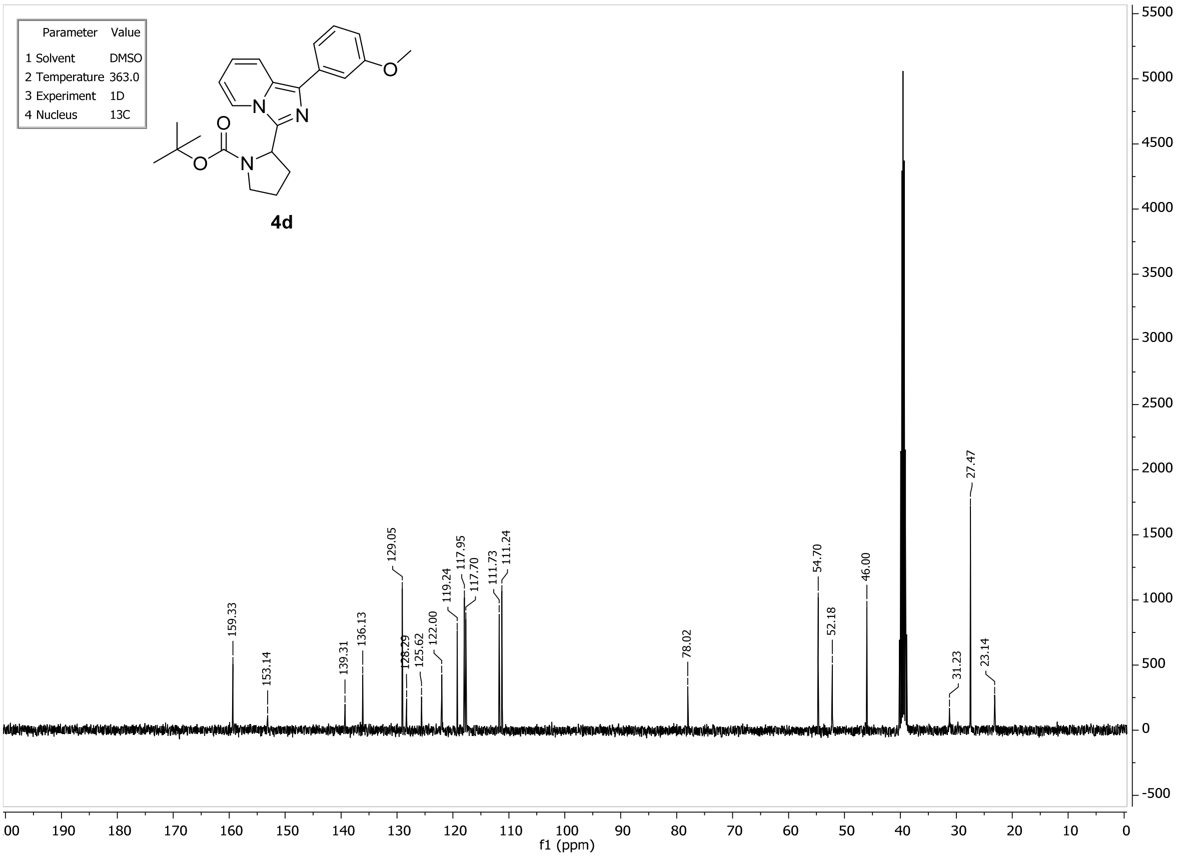
Parameter Value
1 Solvent DMSO
2 Temperature 363.0
3 Experiment 1D
4 Nucleus 13C
4d
159.33
153.14
139.31
136.13
129.05
128.29
125.62
122.00
119.24
117.95
117.70
111.73
111.24
78.02
54.70
52.18
46.00
31.23
27.47
23.14
f1 (ppm)

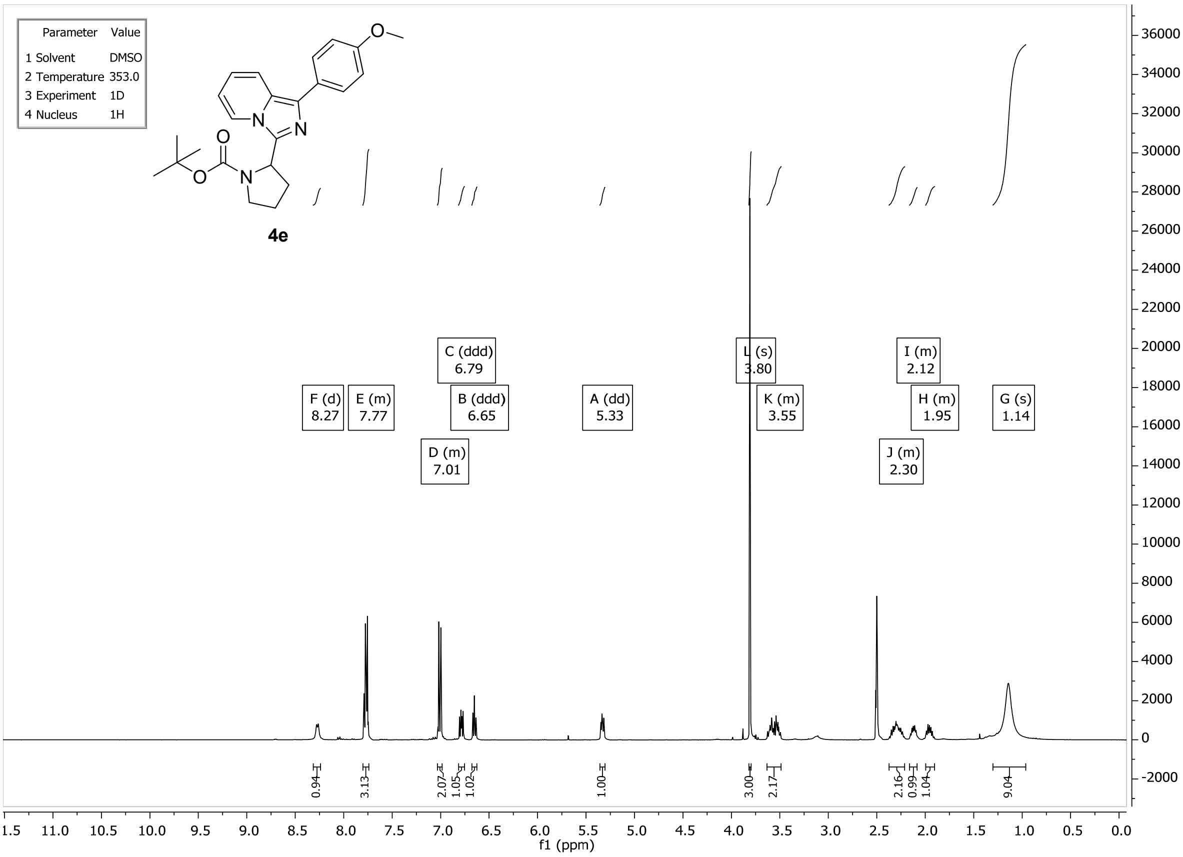
Parameter Value
1 Solvent DMSO
2 Temperature 353.0
3 Experiment 1D
4 Nucleus 1H
4e
F (d) 8.27
E (m) 7.77
C (ddd) 6.79
D (m) 7.01
B (ddd) 6.65
A (dd) 5.33
L (s) 3.80
K (m) 3.55
J (m) 2.30
I (m) 2.12
H (m) 1.95
G (s) 1.14
0.94
3.13
2.07
1.05
1.02
1.00
3.00
2.17
2.16
0.99
1.04
9.04
f1 (ppm)

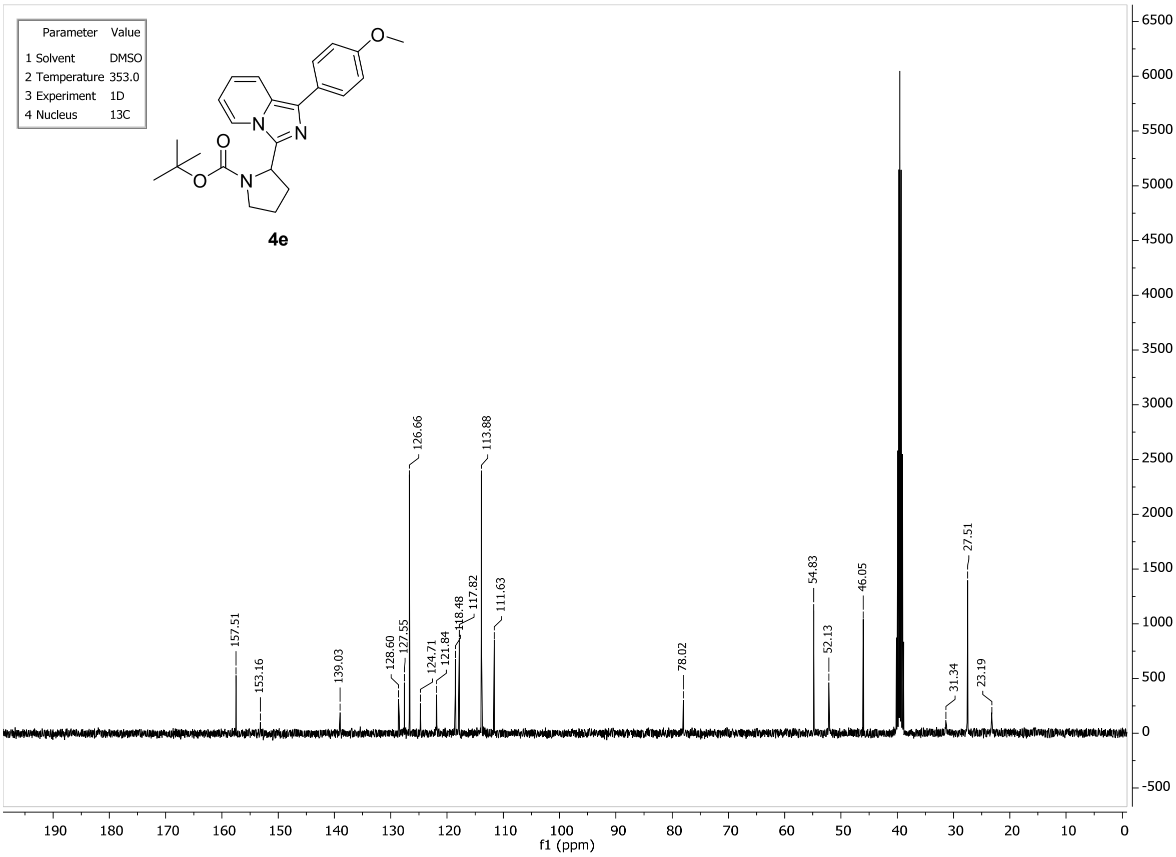
Parameter Value
1 Solvent DMSO
2 Temperature 353.0
3 Experiment 1D
4 Nucleus 13C
4e
157.51
153.16
139.03
128.60
127.55
126.66
124.71
121.84
118.48
117.82
113.88
111.63
78.02
54.83
52.13
46.05
31.34
27.51
23.19
f1 (ppm)

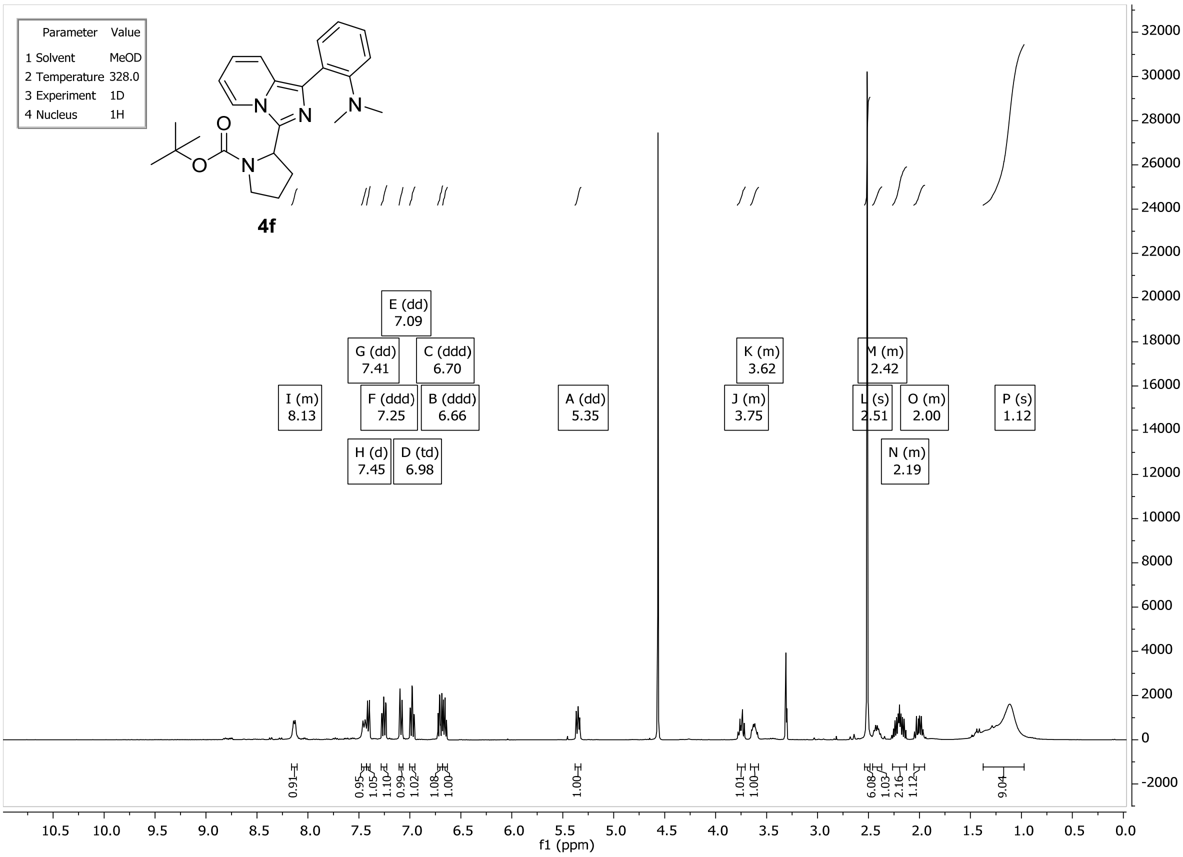
Parameter Value
1 Solvent MeOD
2 Temperature 328.0
3 Experiment 1D
4 Nucleus 1H
4f
E (dd) 7.09
G (dd) 7.41
C (ddd) 6.70
I (m) 8.13
F (ddd) 7.25
B (ddd) 6.66
H (d) 7.45
D (td) 6.98
A (dd) 5.35
K (m) 3.62
J (m) 3.75
M (m) 2.42
L (s) 2.51
O (m) 2.00
N (m) 2.19
P (s) 1.12
0.91
0.95
1.05
1.10
0.99
1.02
1.08
1.00
1.00
1.01
1.00
6.08
1.03
2.16
1.12
9.04
f1 (ppm)

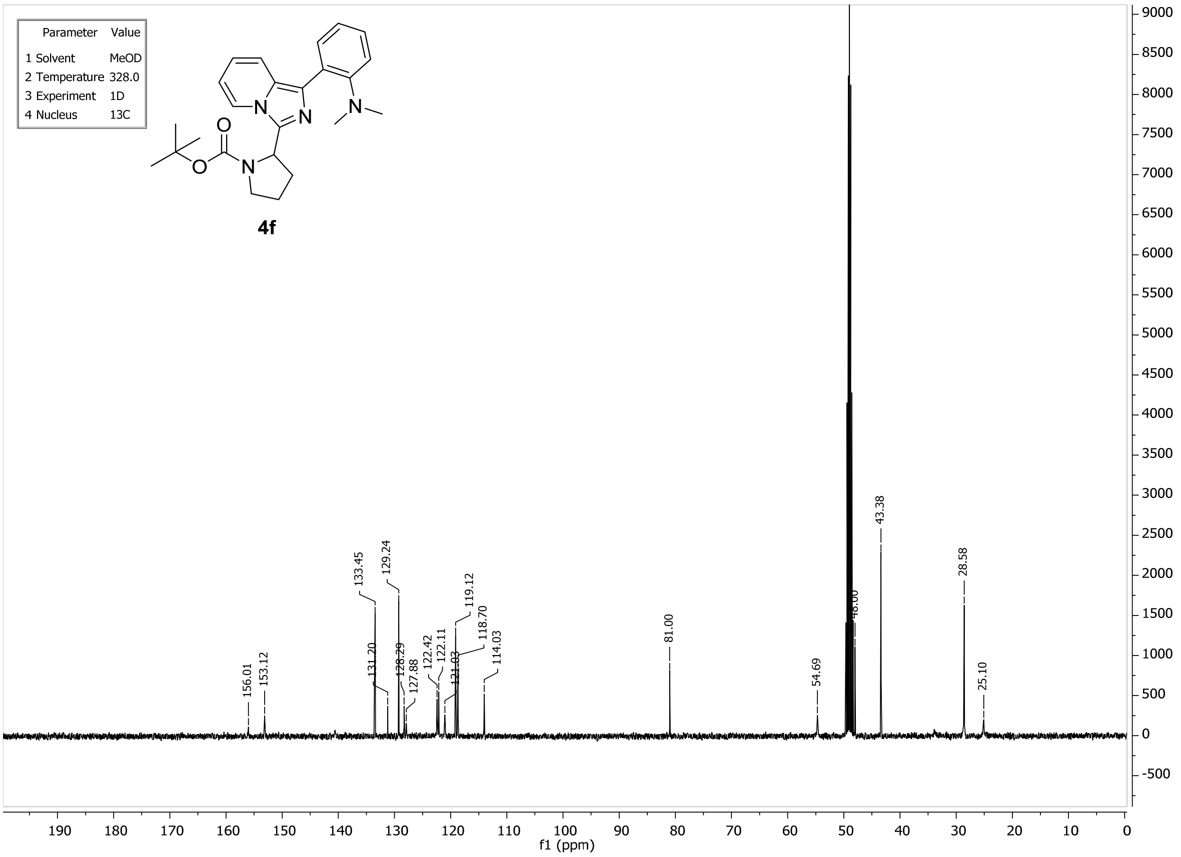
Parameter Value
1 Solvent MeOD
2 Temperature 328.0
3 Experiment 1D
4 Nucleus 13C
4f
156.01
153.12
133.45
131.20
129.24
128.29
127.88
122.42
122.11
121.03
119.12
118.70
114.03
81.00
54.69
48.00
43.38
28.58
25.10
f1 (ppm)

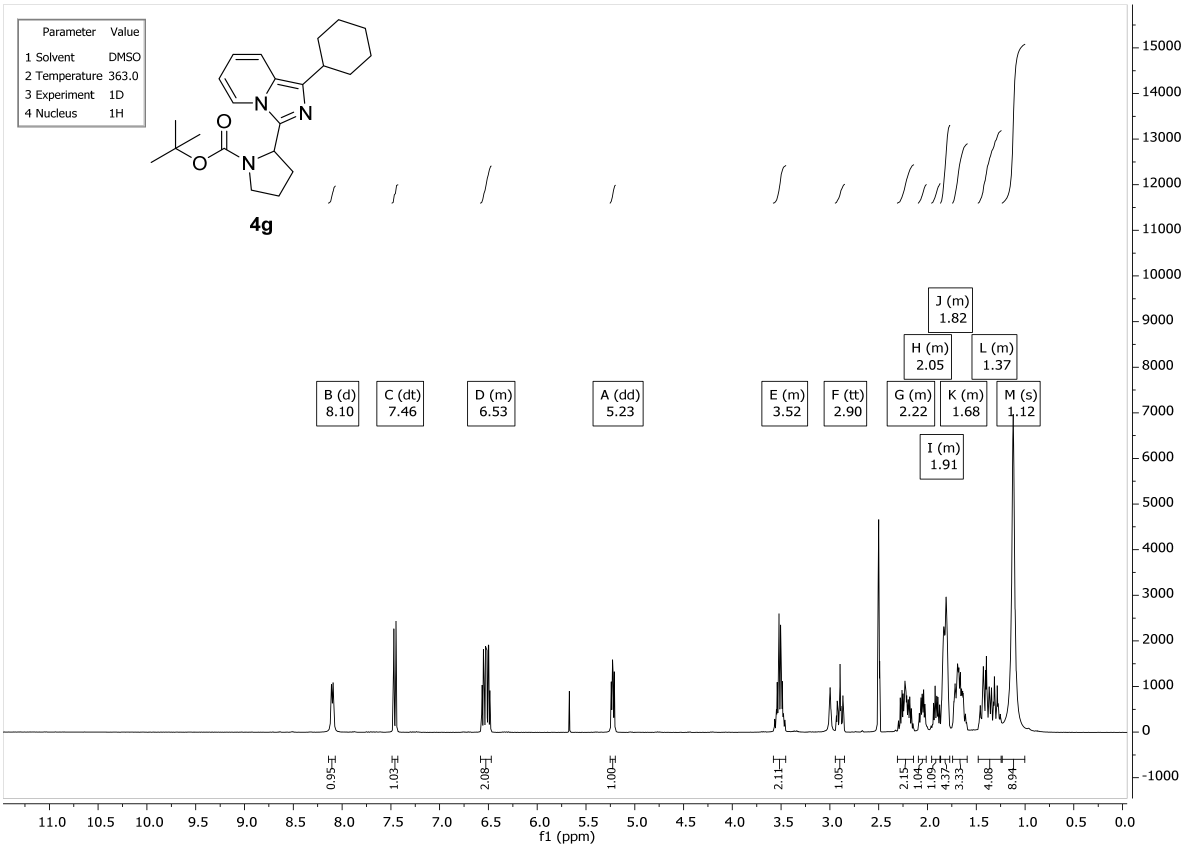

Parameter Value
1 Solvent DMSO
2 Temperature 363.0
3 Experiment 1D
4 Nucleus 1H
4g
B (d) 8.10
C (dt) 7.46
D (m) 6.53
A (dd) 5.23
E (m) 3.52
F (tt) 2.90
G (m) 2.22
H (m) 2.05
I (m) 1.91
J (m) 1.82
K (m) 1.68
L (m) 1.37
M (s) 1.12
f1 (ppm)

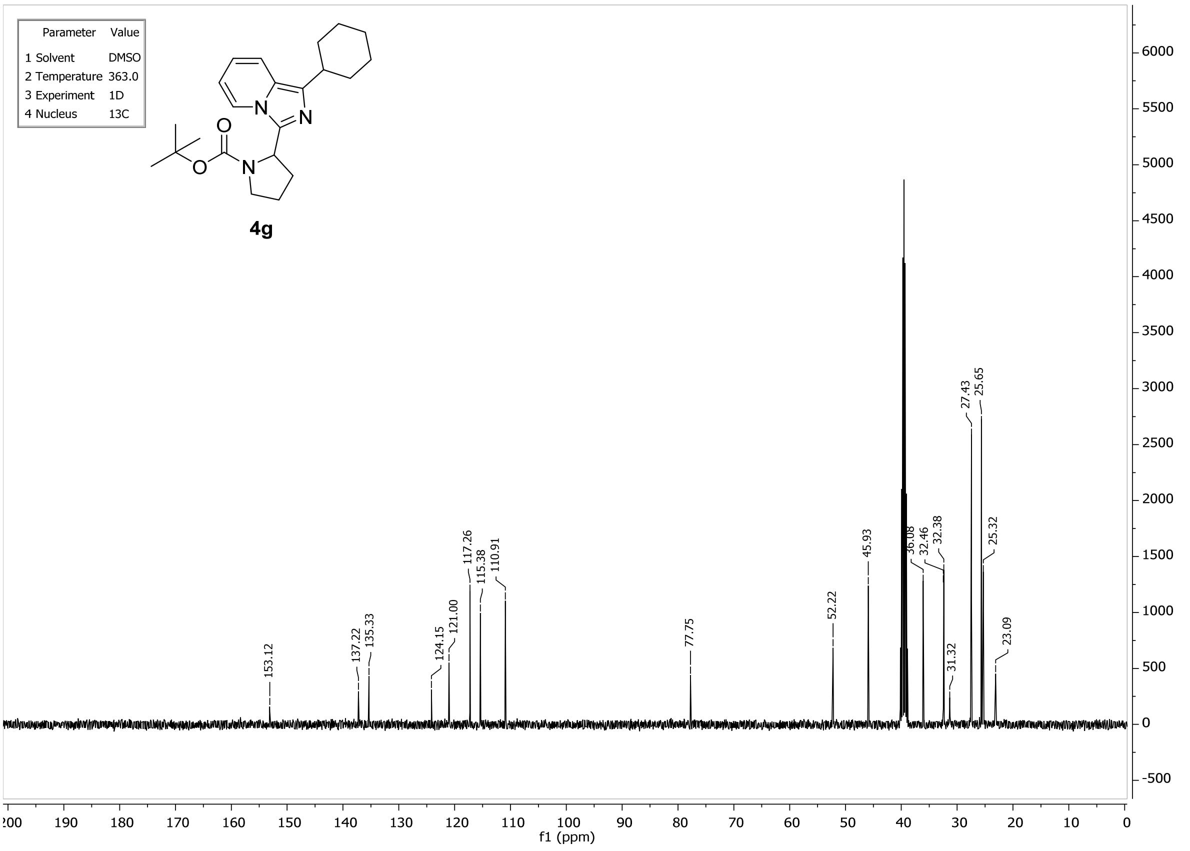

Parameter Value
1 Solvent DMSO
2 Temperature 363.0
3 Experiment 1D
4 Nucleus 13C
4g
153.12
137.22
135.33
124.15
121.00
117.26
115.38
110.91
77.75
52.22
45.93
36.08
32.46
32.38
31.32
27.43
25.65
25.32
23.09
f1 (ppm)

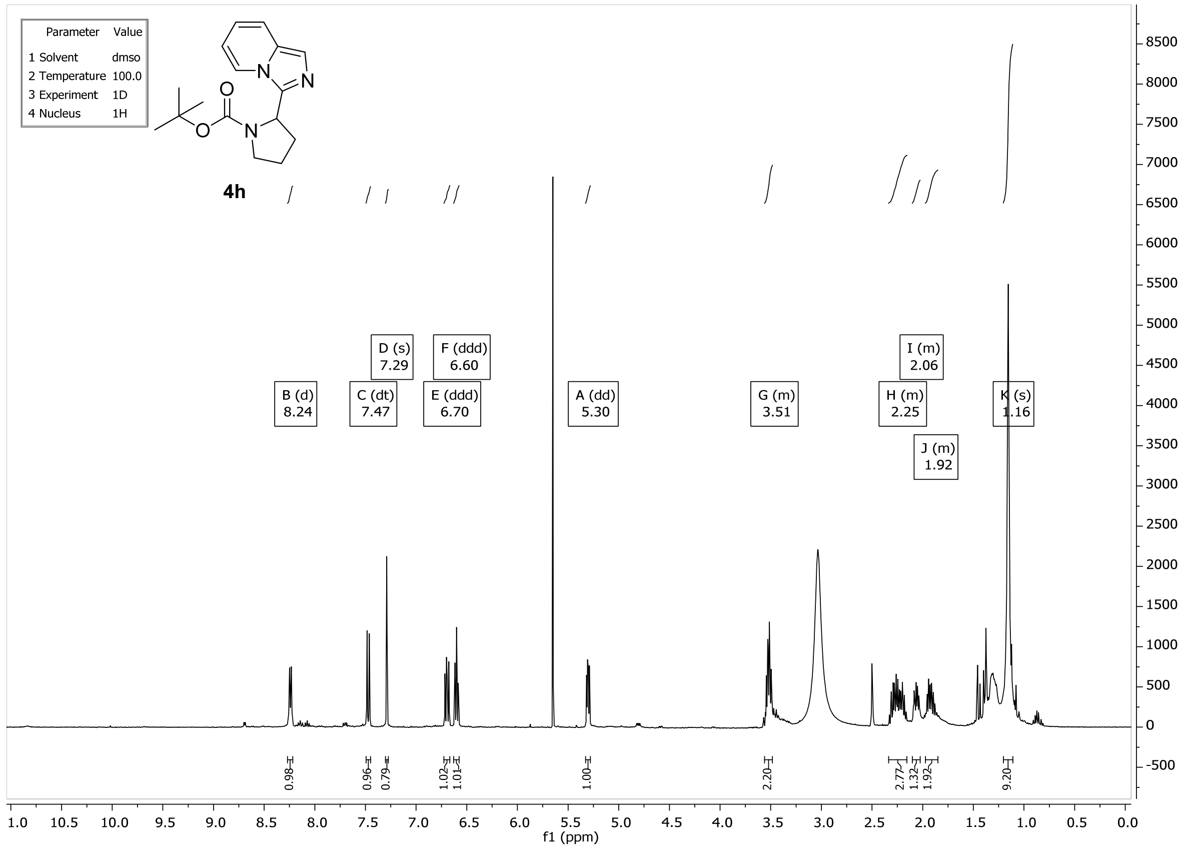
Parameter Value
1 Solvent dmso
2 Temperature 100.0
3 Experiment 1D
4 Nucleus 1H
4h
B (d) 8.24
C (dt) 7.47
D (s) 7.29
E (ddd) 6.70
F (ddd) 6.60
A (dd) 5.30
G (m) 3.51
H (m) 2.25
I (m) 2.06
J (m) 1.92
K (s) 1.16
0.98
0.96
0.79
1.02
1.01
1.00
2.20
2.77
1.32
1.92
9.20
f1 (ppm)

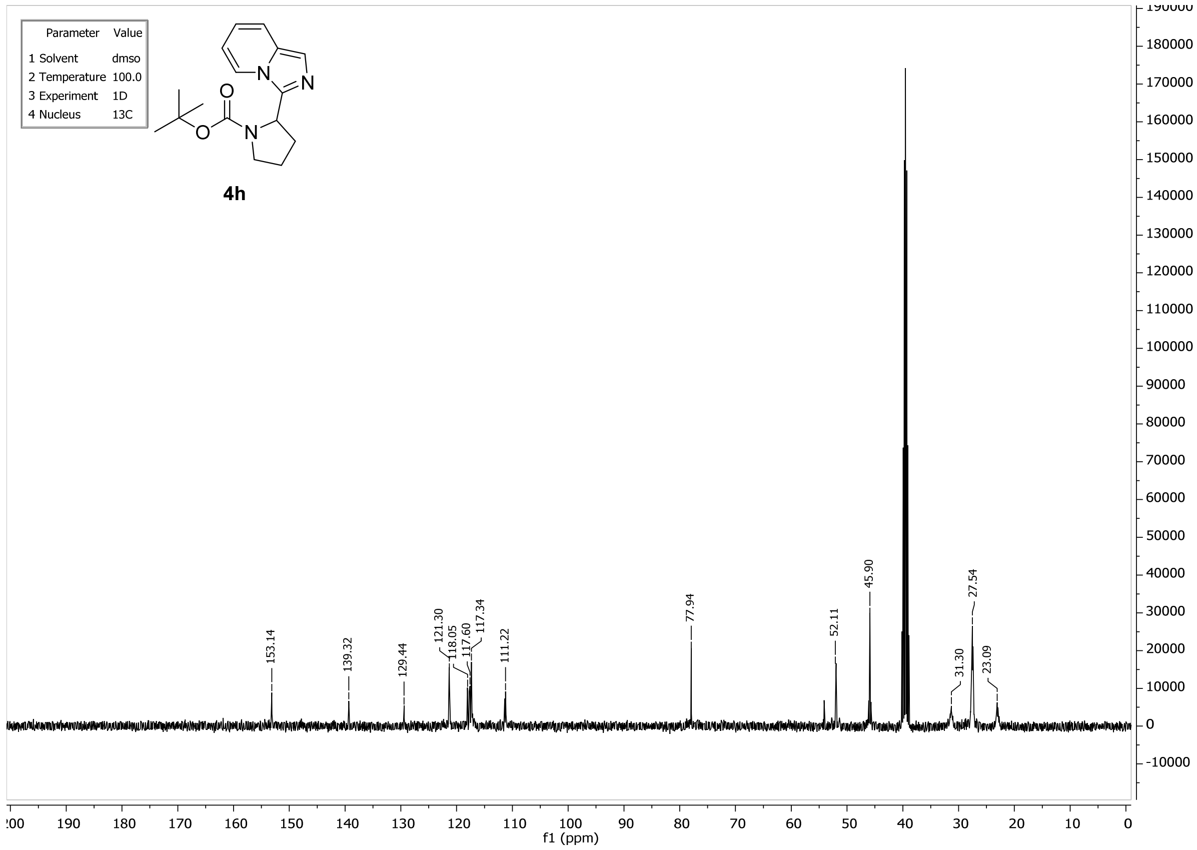
Parameter Value
1 Solvent dmso
2 Temperature 100.0
3 Experiment 1D
4 Nucleus 13C
4h
153.14
139.32
129.44
121.30
118.05
117.60
117.34
111.22
77.94
52.11
45.90
31.30
27.54
23.09
f1 (ppm)

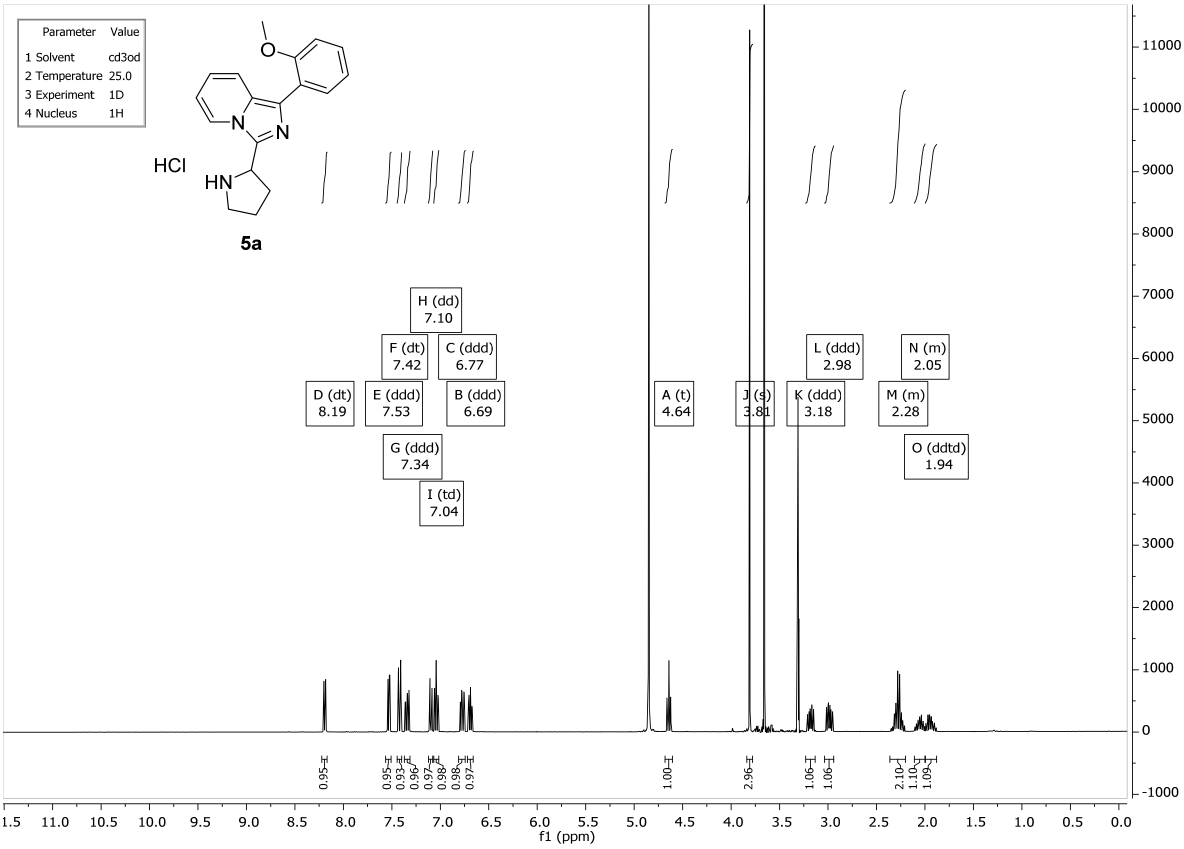
Parameter Value
1 Solvent cd3od
2 Temperature 25.0
3 Experiment 1D
4 Nucleus 1H
HCl
HN
N
N
O
5a
D (dt) 8.19
E (ddd) 7.53
F (dt) 7.42
G (ddd) 7.34
H (dd) 7.10
I (td) 7.04
C (ddd) 6.77
B (ddd) 6.69
A (t) 4.64
J (s) 3.81
K (ddd) 3.18
L (ddd) 2.98
M (m) 2.28
N (m) 2.05
O (ddtd) 1.94
0.95
0.95
0.93
0.96
0.97
0.98
0.98
0.97
1.00
2.96
1.06
1.06
2.10
1.10
1.09
f1 (ppm)

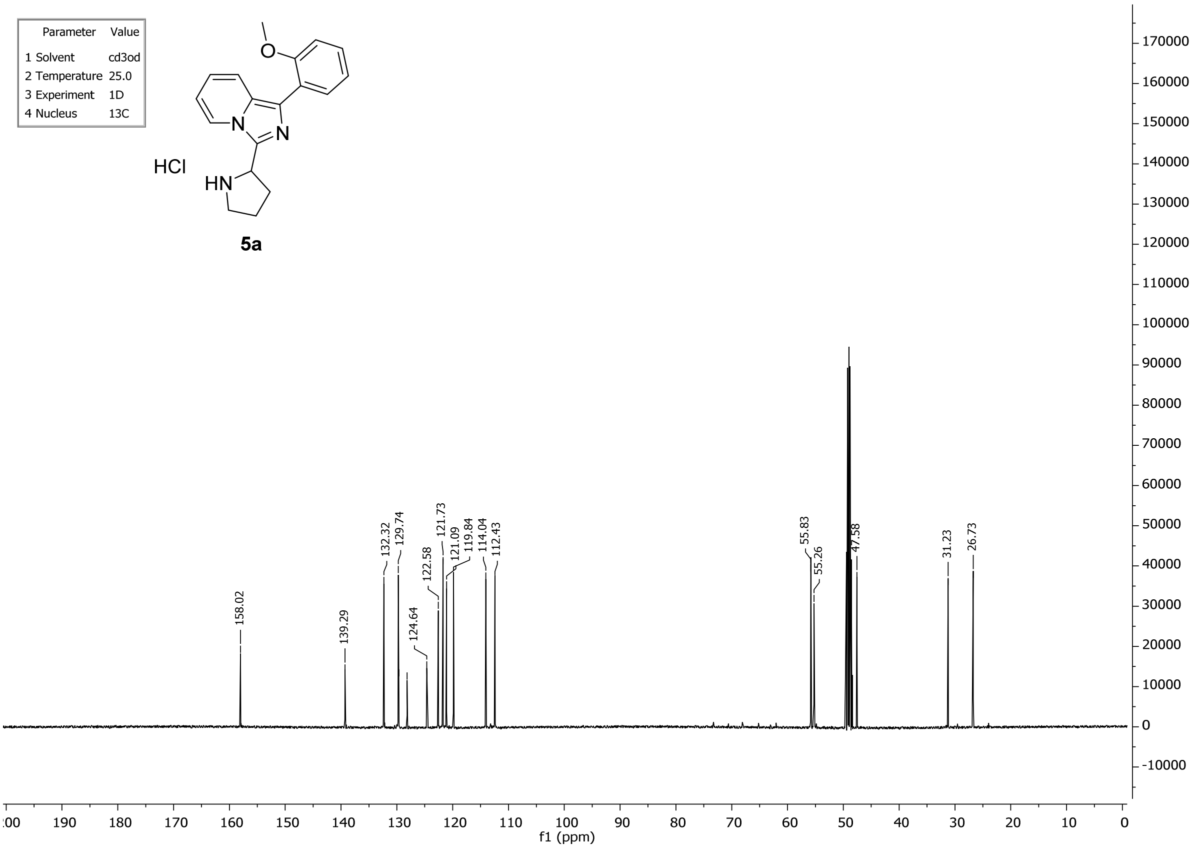
Parameter Value
1 Solvent cd3od
2 Temperature 25.0
3 Experiment 1D
4 Nucleus 13C
HCl
HN
N
N
O
5a
158.02
139.29
132.32
129.74
124.64
122.58
121.73
121.09
119.84
114.04
112.43
55.83
55.26
47.58
31.23
26.73
f1 (ppm)

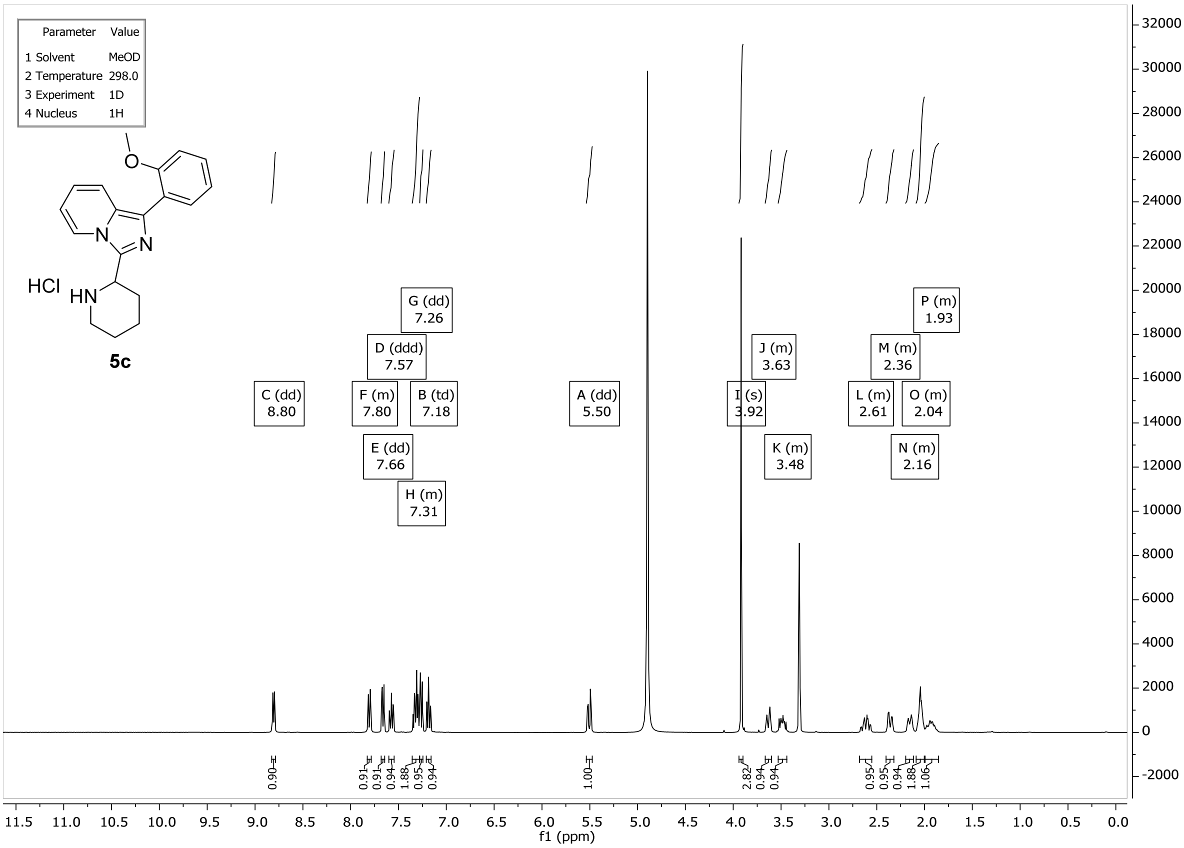

Parameter Value
1 Solvent MeOD
2 Temperature 298.0
3 Experiment 1D
4 Nucleus 1H
HCl
5c
C (dd) 8.80
F (m) 7.80
E (dd) 7.66
D (ddd) 7.57
H (m) 7.31
G (dd) 7.26
B (td) 7.18
A (dd) 5.50
I (s) 3.92
J (m) 3.63
K (m) 3.48
L (m) 2.61
M (m) 2.36
N (m) 2.16
O (m) 2.04
P (m) 1.93
f1 (ppm)

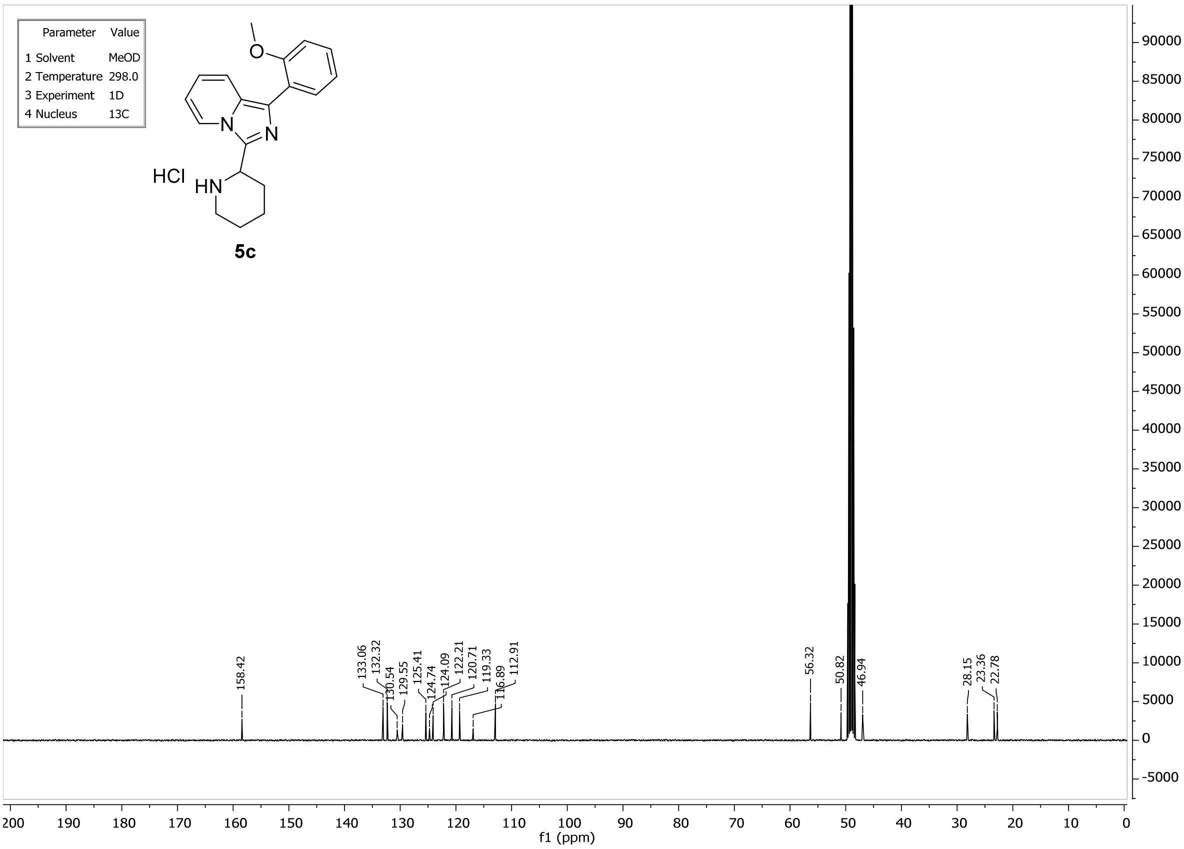

Parameter Value
1 Solvent MeOD
2 Temperature 298.0
3 Experiment 1D
4 Nucleus 13C
HCl
5c
158.42
133.06
132.32
130.54
129.55
125.41
124.74
124.09
122.21
120.71
119.33
116.89
112.91
56.32
50.82
46.94
28.15
23.36
22.78
f1 (ppm)

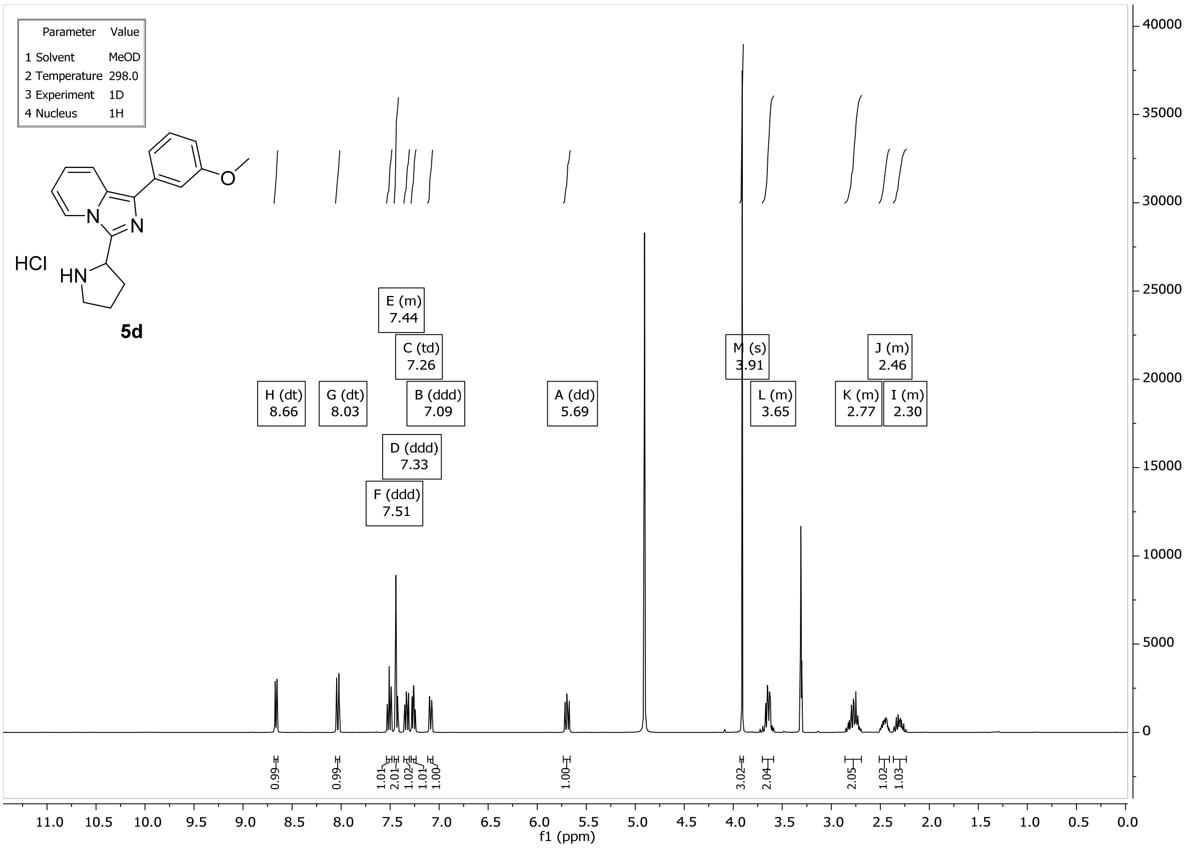
Parameter Value
1 Solvent MeOD
2 Temperature 298.0
3 Experiment 1D
4 Nucleus 1H
HCl
5d
H (dt) 8.66
G (dt) 8.03
E (m) 7.44
C (td) 7.26
B (ddd) 7.09
D (ddd) 7.33
F (ddd) 7.51
A (dd) 5.69
M (s) 3.91
L (m) 3.65
K (m) 2.77
J (m) 2.46
I (m) 2.30
0.99 0.99 1.01 2.01 1.02 1.01 1.00 1.00 3.02 2.04 2.05 1.02 1.03
f1 (ppm)

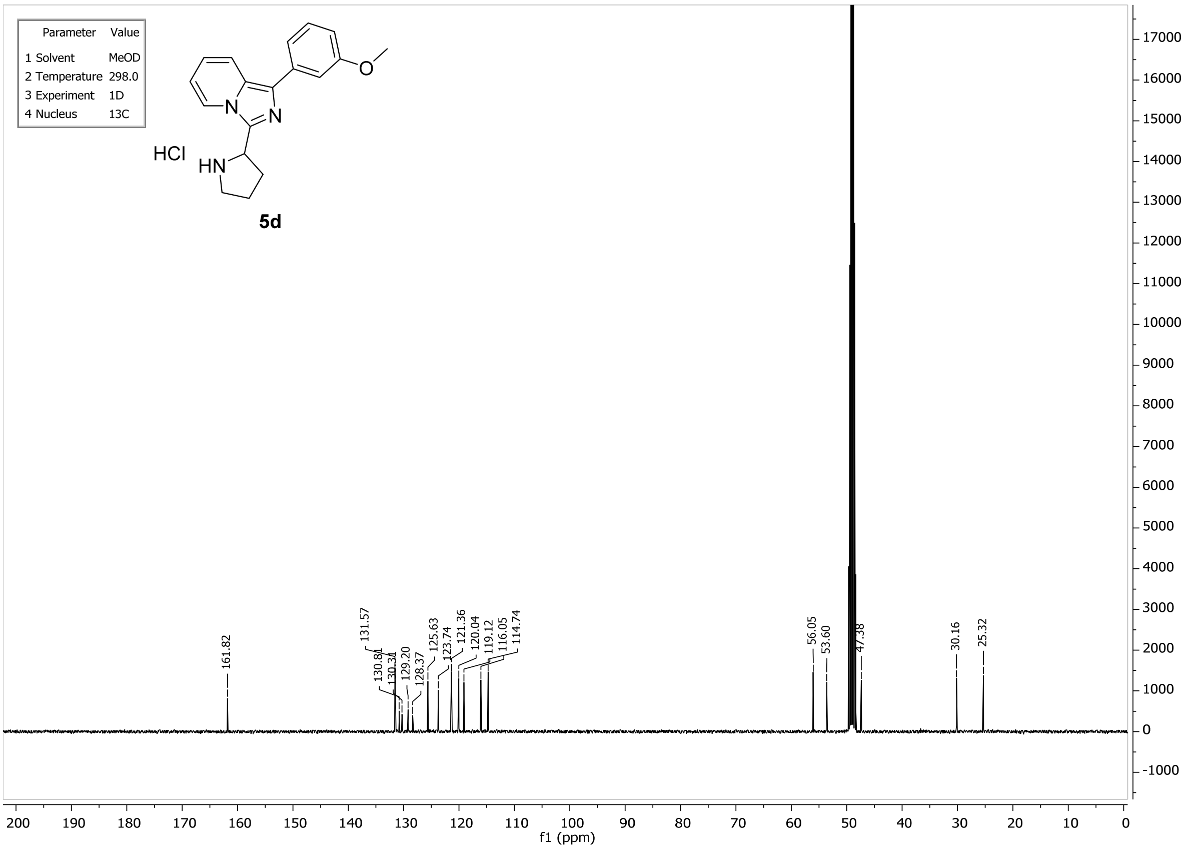
Parameter Value
1 Solvent MeOD
2 Temperature 298.0
3 Experiment 1D
4 Nucleus 13C
HCl
5d
161.82
131.57 130.84 130.31 129.20 128.37 125.63 123.74 121.36 120.04 119.12 116.05 114.74
56.05 53.60 47.38
30.16 25.32
f1 (ppm)

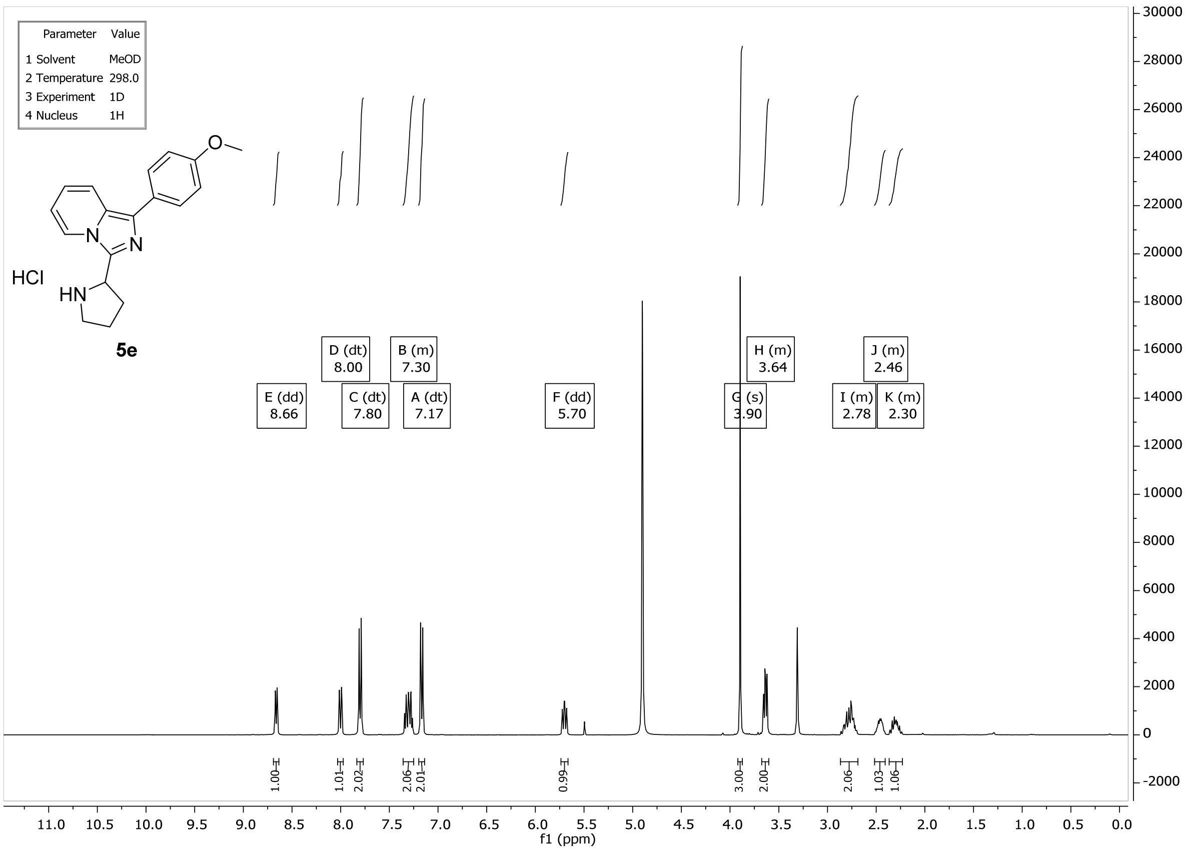

Parameter Value
1 Solvent MeOD
2 Temperature 298.0
3 Experiment 1D
4 Nucleus 1H
HCl
HN
N
N
O
5e
E (dd) 8.66
D (dt) 8.00
C (dt) 7.80
B (m) 7.30
A (dt) 7.17
F (dd) 5.70
G (s) 3.90
H (m) 3.64
I (m) 2.78
J (m) 2.46
K (m) 2.30
1.00
1.01
2.02
2.06
2.01
0.99
3.00
2.00
2.06
1.03
1.06
f1 (ppm)

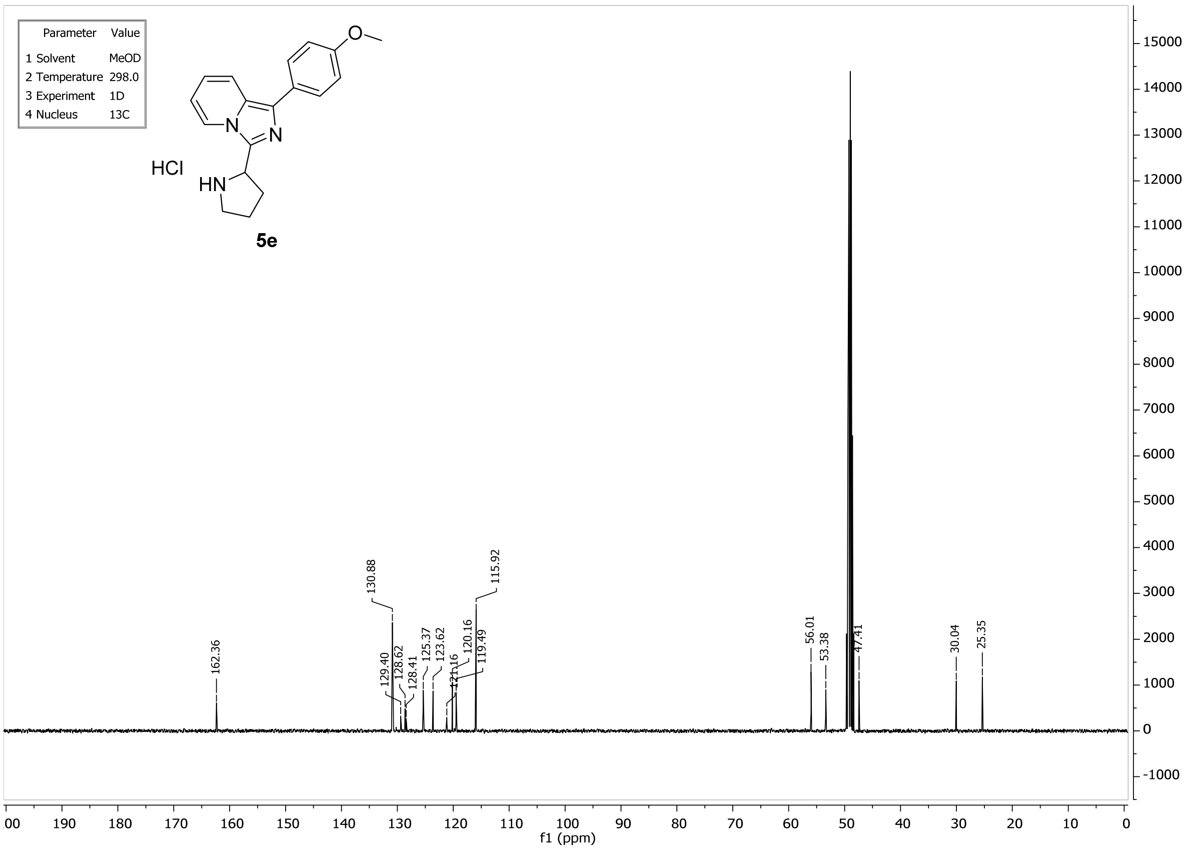

Parameter Value
1 Solvent MeOD
2 Temperature 298.0
3 Experiment 1D
4 Nucleus 13C
HCl
HN
N
N
O
5e
162.36
130.88
129.40
128.62
128.41
125.37
123.62
121.16
120.16
119.49
115.92
56.01
53.38
47.41
30.04
25.35
f1 (ppm)

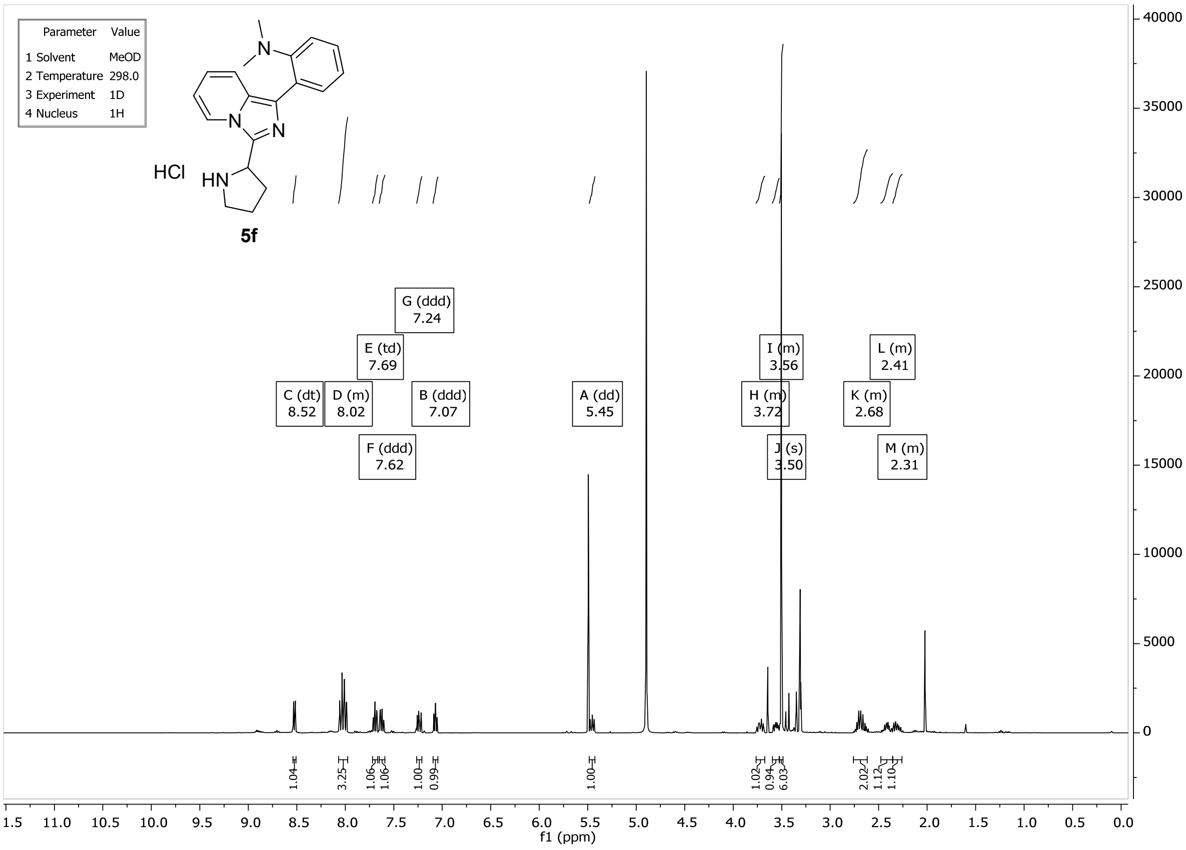
Parameter Value
1 Solvent MeOD
2 Temperature 298.0
3 Experiment 1D
4 Nucleus 1H
HCl
HN
N
N
N
5f
G (ddd) 7.24
E (td) 7.69
C (dt) 8.52
D (m) 8.02
B (ddd) 7.07
F (ddd) 7.62
A (dd) 5.45
I (m) 3.56
H (m) 3.72
J (s) 3.50
L (m) 2.41
K (m) 2.68
M (m) 2.31
1.04
3.25
1.06
1.06
1.00
0.99
1.00
1.02
0.94
6.03
2.02
1.12
1.10
f1 (ppm)

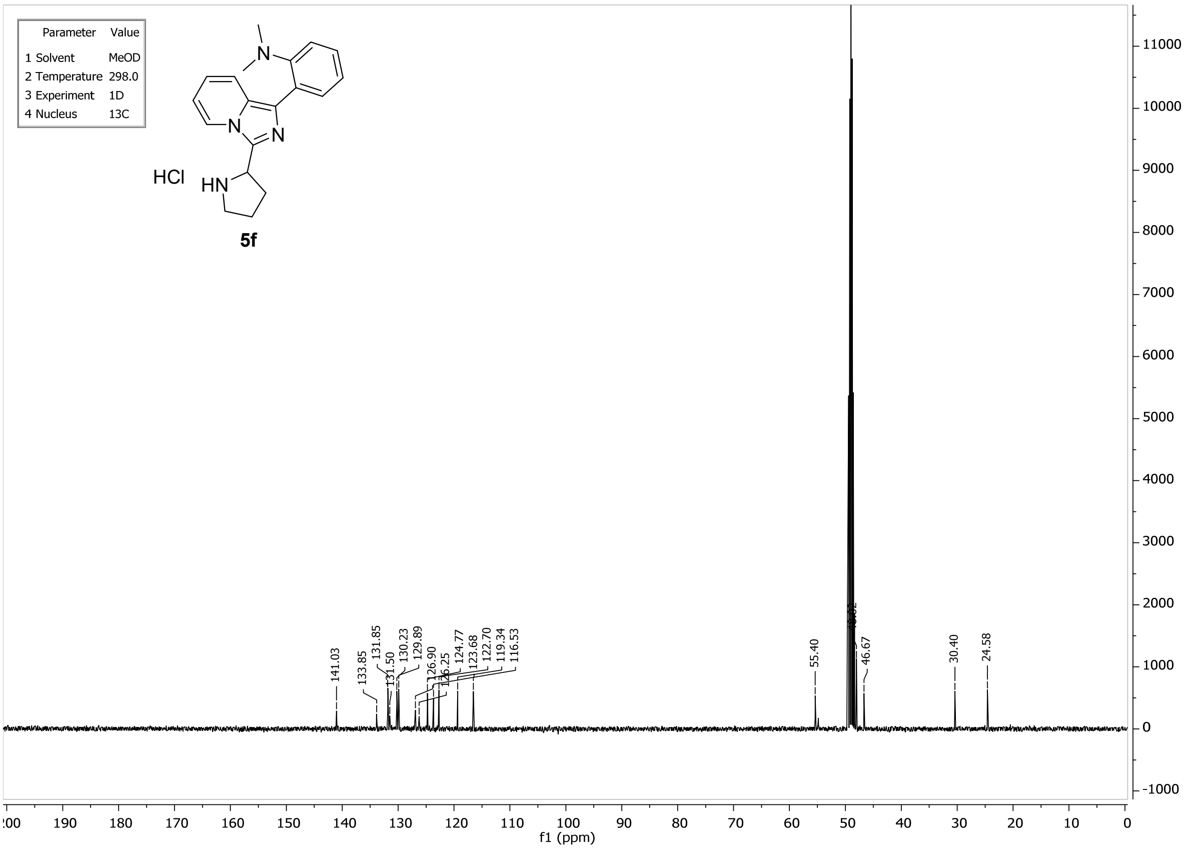
Parameter Value
1 Solvent MeOD
2 Temperature 298.0
3 Experiment 1D
4 Nucleus 13C
HCl
HN
N
N
N
5f
141.03
133.85
131.85
131.50
130.23
129.89
126.90
126.25
124.77
123.68
122.70
119.34
116.53
55.40
46.67
30.40
24.58
f1 (ppm)

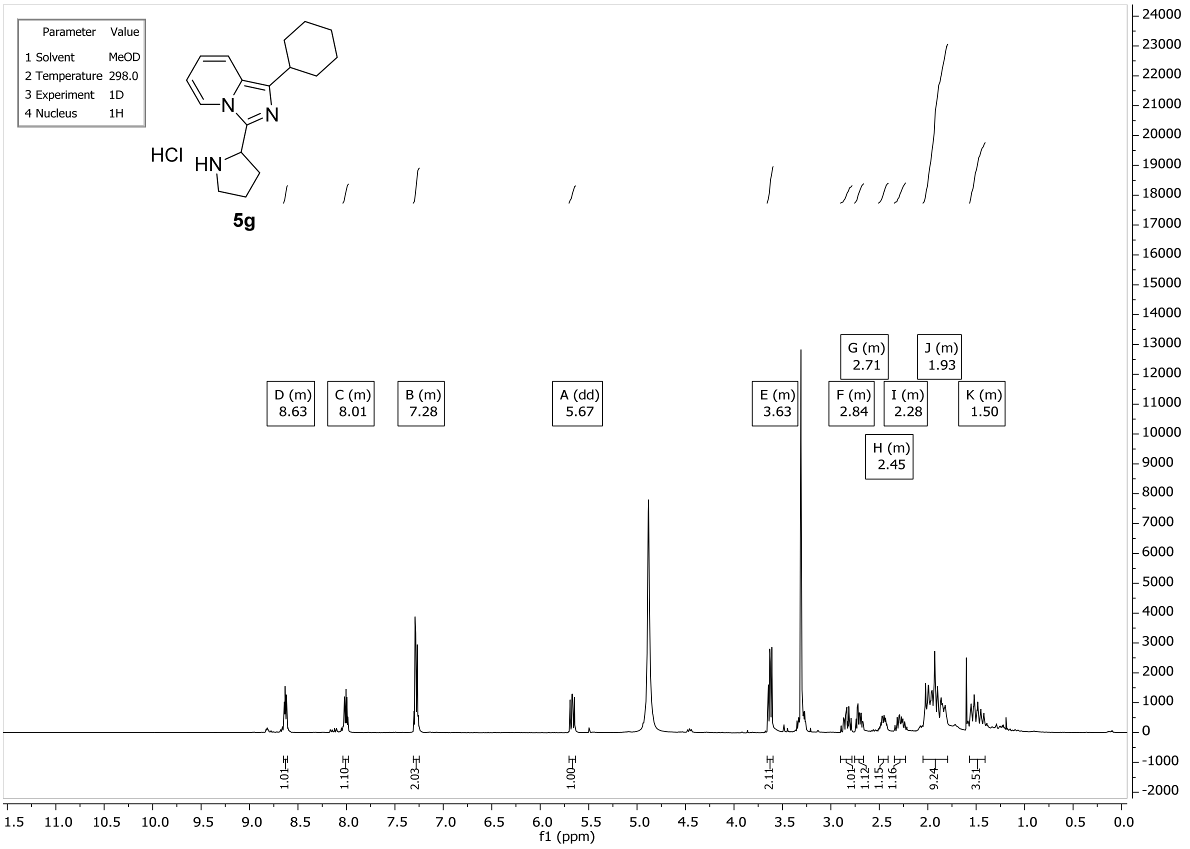
Parameter Value
1 Solvent MeOD
2 Temperature 298.0
3 Experiment 1D
4 Nucleus 1H
HCl
HN
N
N
5g
D (m) 8.63
C (m) 8.01
B (m) 7.28
A (dd) 5.67
E (m) 3.63
F (m) 2.84
G (m) 2.71
H (m) 2.45
I (m) 2.28
J (m) 1.93
K (m) 1.50
1.01
1.10
2.03
1.00
2.11
1.01
1.12
1.15
1.16
9.24
3.51
f1 (ppm)

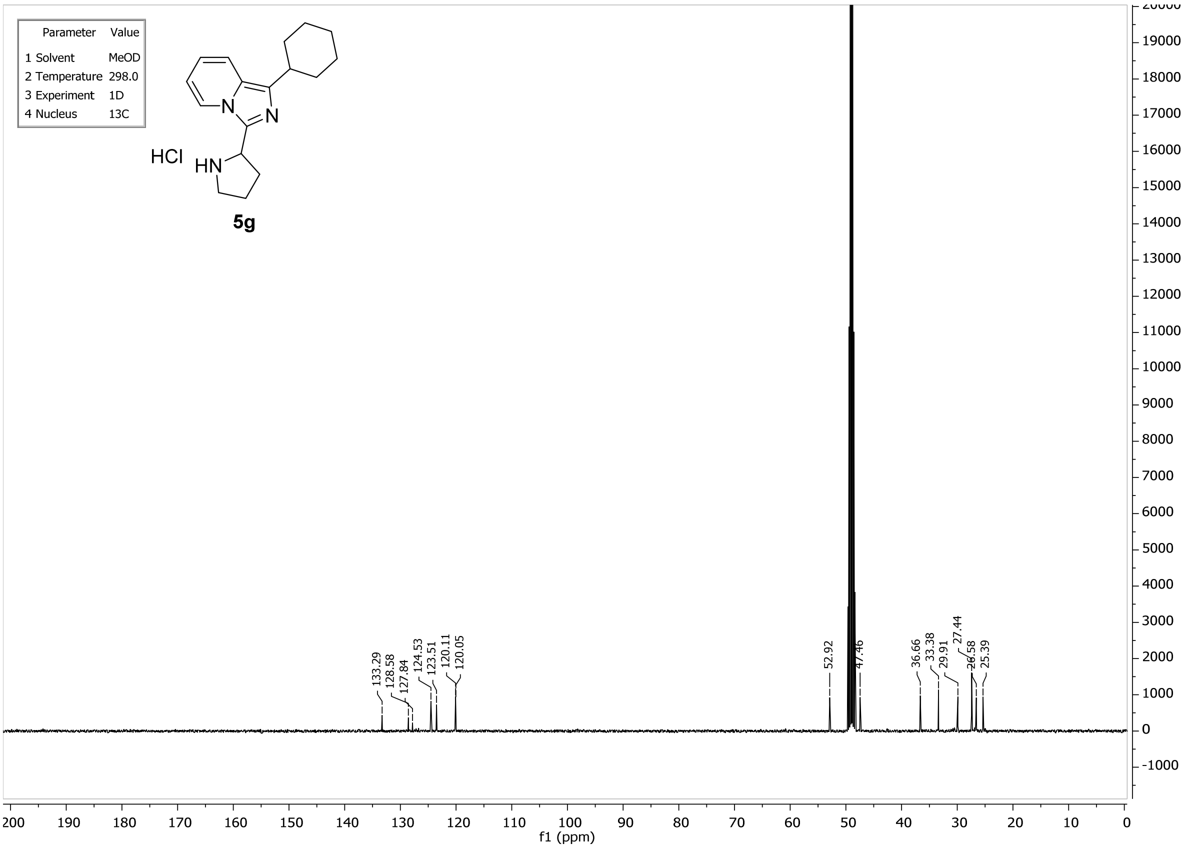
Parameter Value
1 Solvent MeOD
2 Temperature 298.0
3 Experiment 1D
4 Nucleus 13C
HCl
HN
N
N
5g
133.29
128.58
127.84
124.53
123.51
120.11
120.05
52.92
47.46
36.66
33.38
29.91
27.44
26.58
25.39
f1 (ppm)

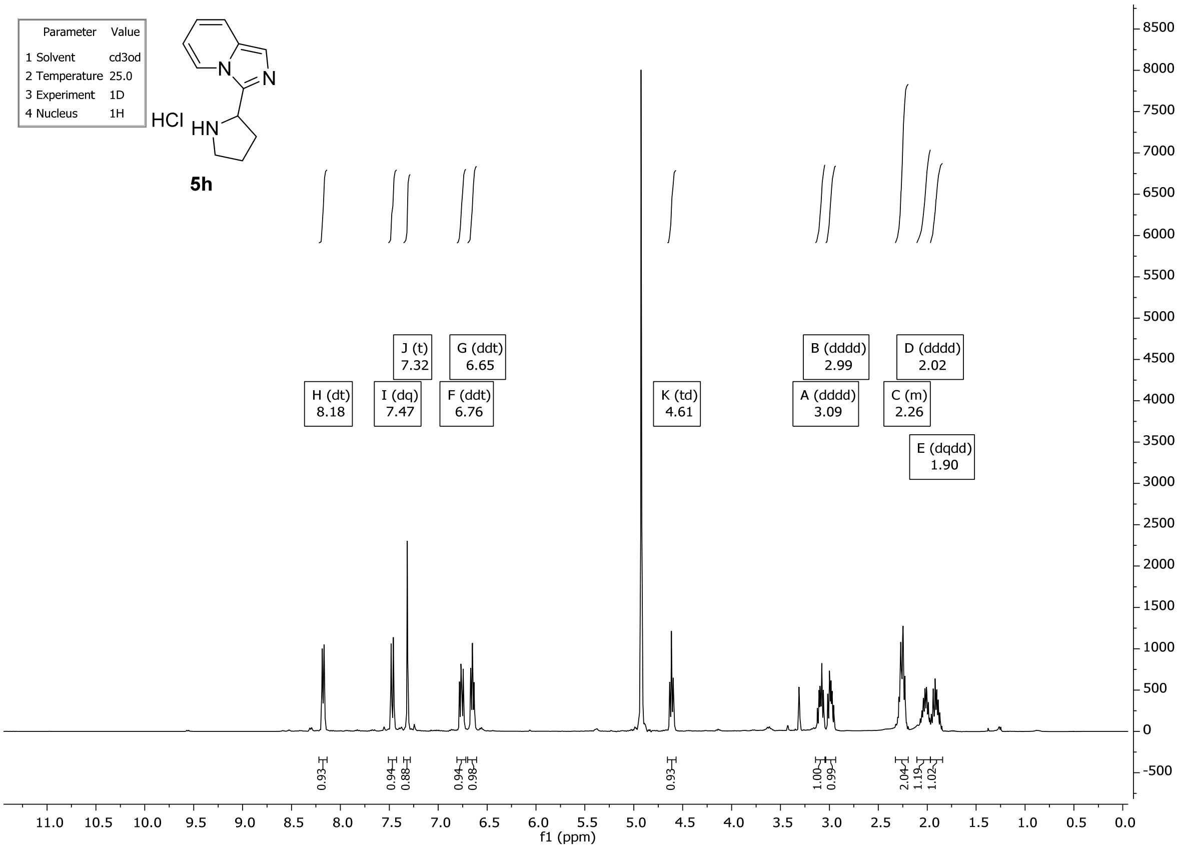

Parameter Value
1 Solvent cd3od
2 Temperature 25.0
3 Experiment 1D
4 Nucleus 1H
HCl
5h
J (t) 7.32
G (ddt) 6.65
B (dddd) 2.99
D (dddd) 2.02
H (dt) 8.18
I (dq) 7.47
F (ddt) 6.76
K (td) 4.61
A (dddd) 3.09
C (m) 2.26
E (dqdd) 1.90
f1 (ppm)

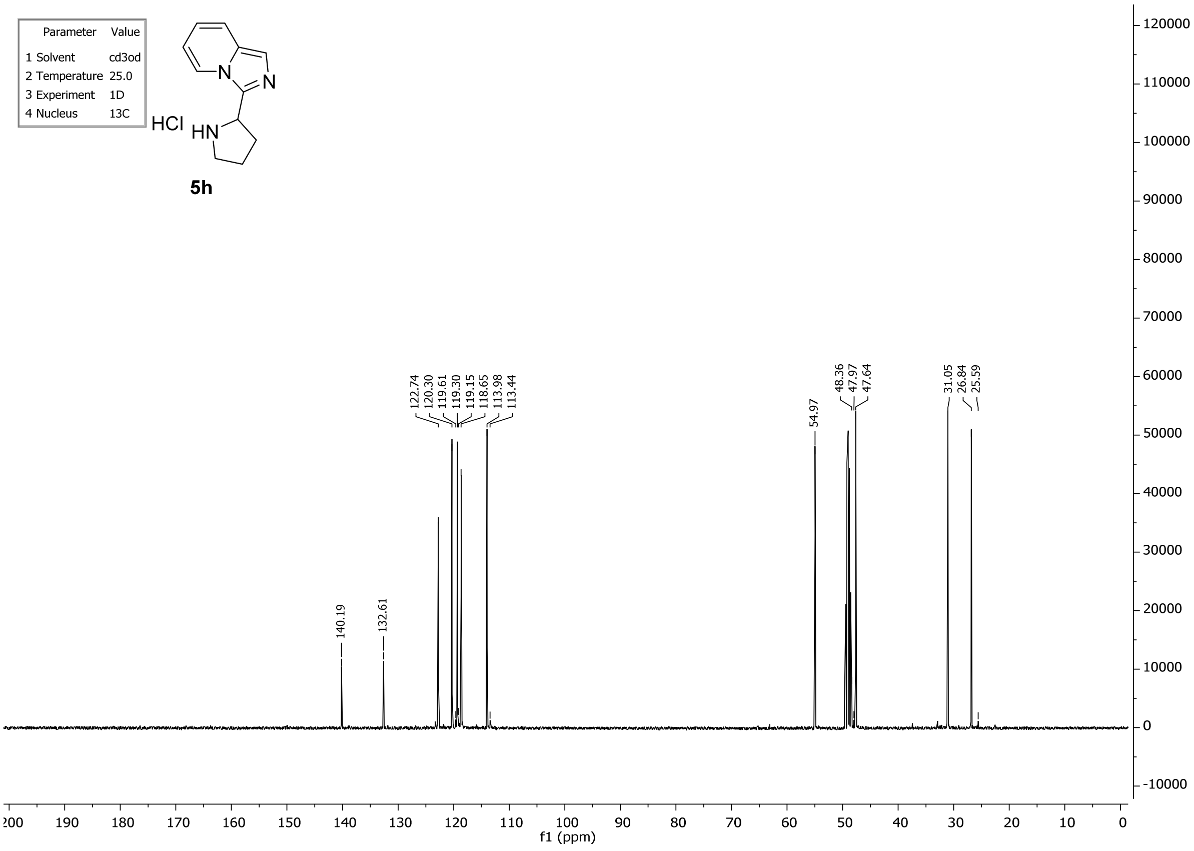

Parameter Value
1 Solvent cd3od
2 Temperature 25.0
3 Experiment 1D
4 Nucleus 13C
HCl
5h
140.19
132.61
122.74
120.30
119.61
119.30
119.15
118.65
113.98
113.44
54.97
48.36
47.97
47.64
31.05
26.84
25.59
f1 (ppm)

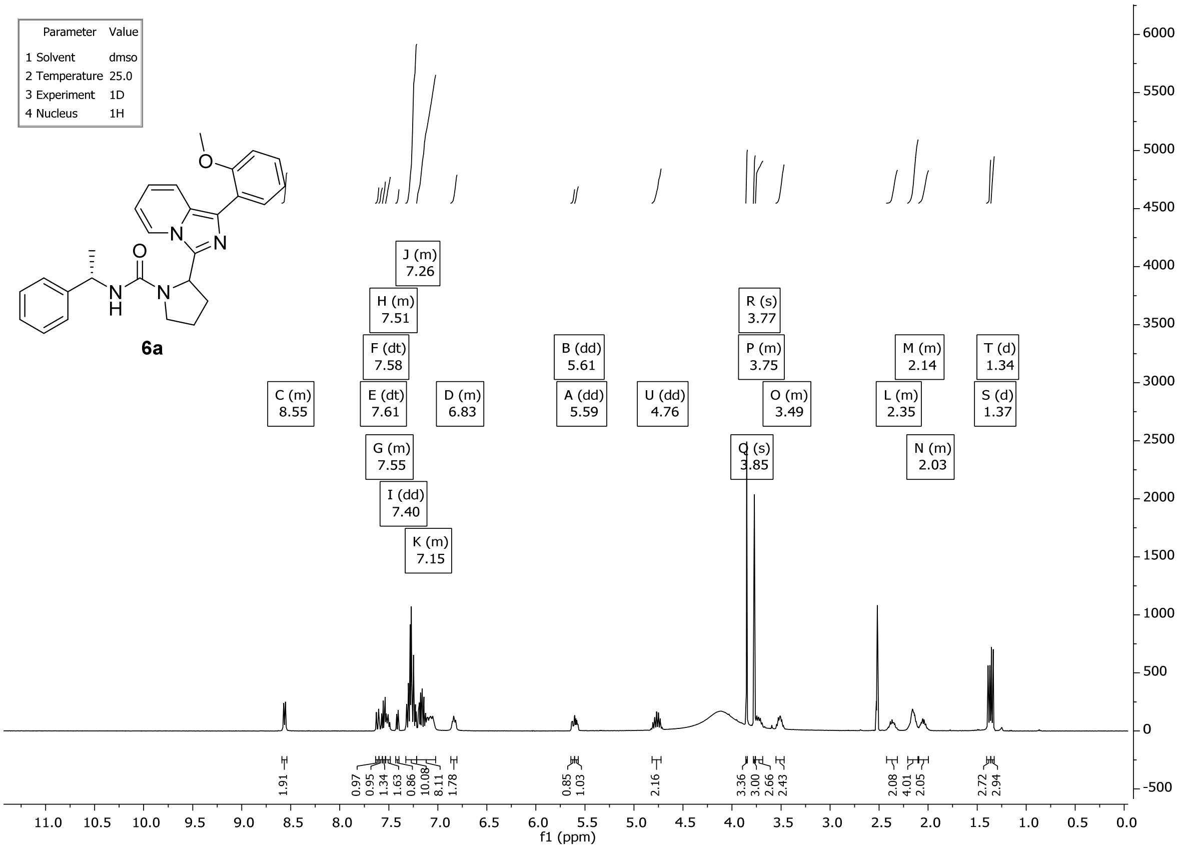
Parameter Value
1 Solvent dmso
2 Temperature 25.0
3 Experiment 1D
4 Nucleus 1H
6a
C (m) 8.55
E (dt) 7.61
F (dt) 7.58
G (m) 7.55
H (m) 7.51
I (dd) 7.40
J (m) 7.26
K (m) 7.15
D (m) 6.83
B (dd) 5.61
A (dd) 5.59
U (dd) 4.76
Q (s) 3.85
R (s) 3.77
P (m) 3.75
O (m) 3.49
L (m) 2.35
M (m) 2.14
N (m) 2.03
S (d) 1.37
T (d) 1.34
f1 (ppm)

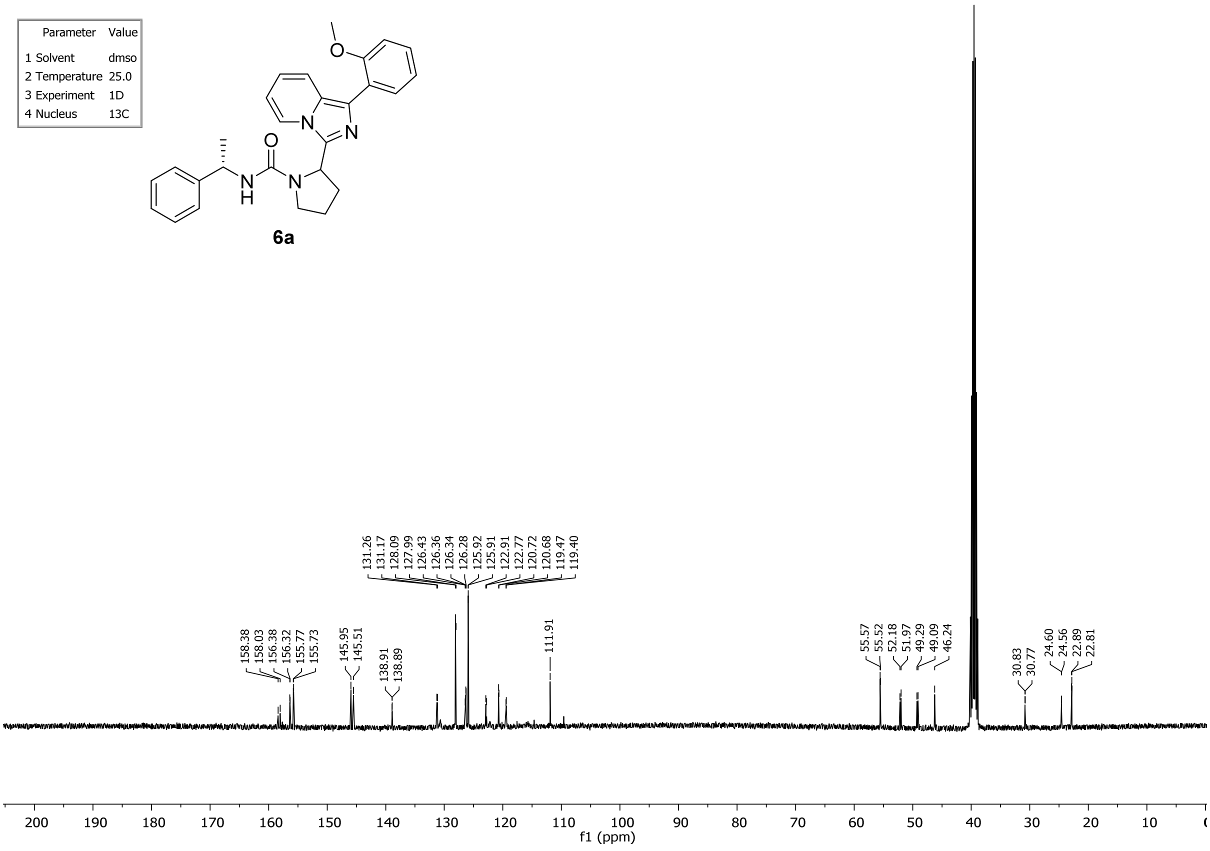
Parameter Value
1 Solvent dmso
2 Temperature 25.0
3 Experiment 1D
4 Nucleus 13C
6a
f1 (ppm)

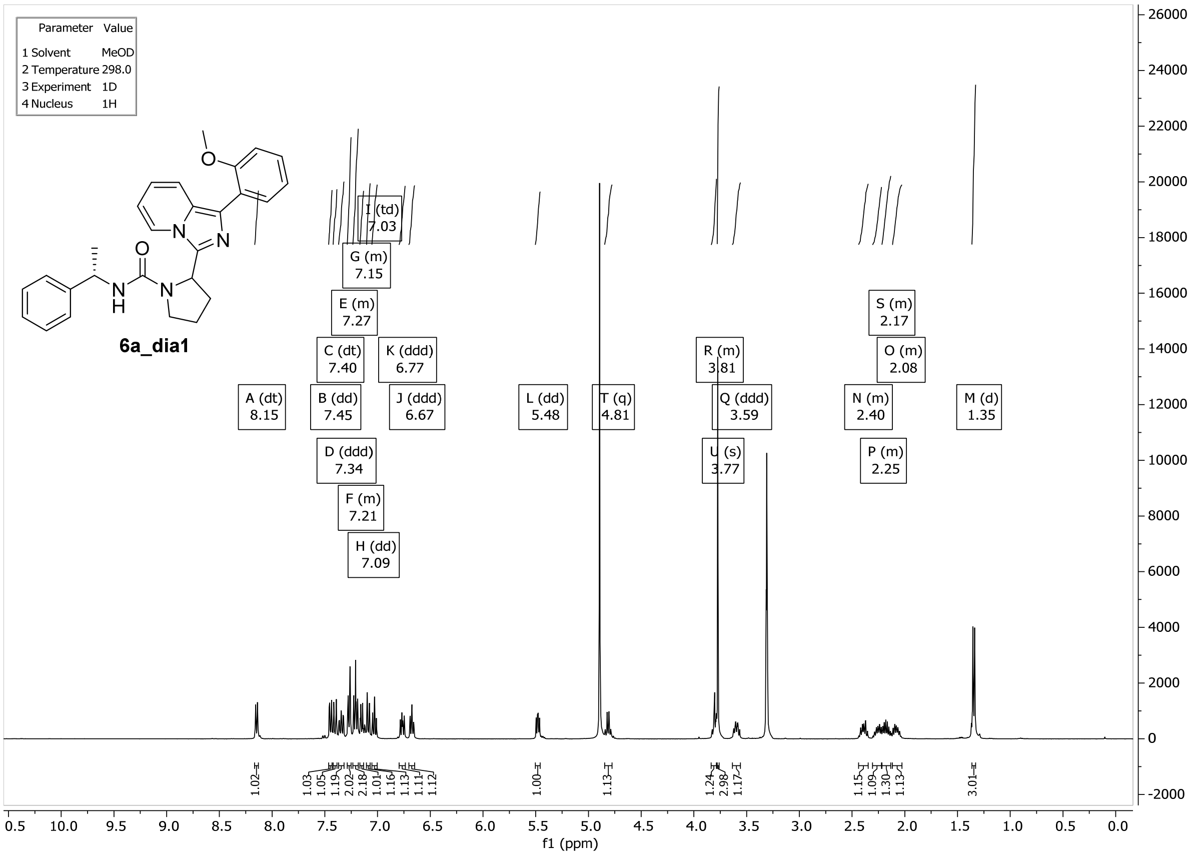
Parameter Value
1 Solvent MeOD
2 Temperature 298.0
3 Experiment 1D
4 Nucleus 1H
6a_dia1
A (dt) 8.15
B (dd) 7.45
C (dt) 7.40
D (ddd) 7.34
E (m) 7.27
F (m) 7.21
G (m) 7.15
H (dd) 7.09
I (td) 7.03
J (ddd) 6.67
K (ddd) 6.77
L (dd) 5.48
T (q) 4.81
R (m) 3.81
U (s) 3.77
Q (ddd) 3.59
N (m) 2.40
P (m) 2.25
S (m) 2.17
O (m) 2.08
M (d) 1.35
f1 (ppm)

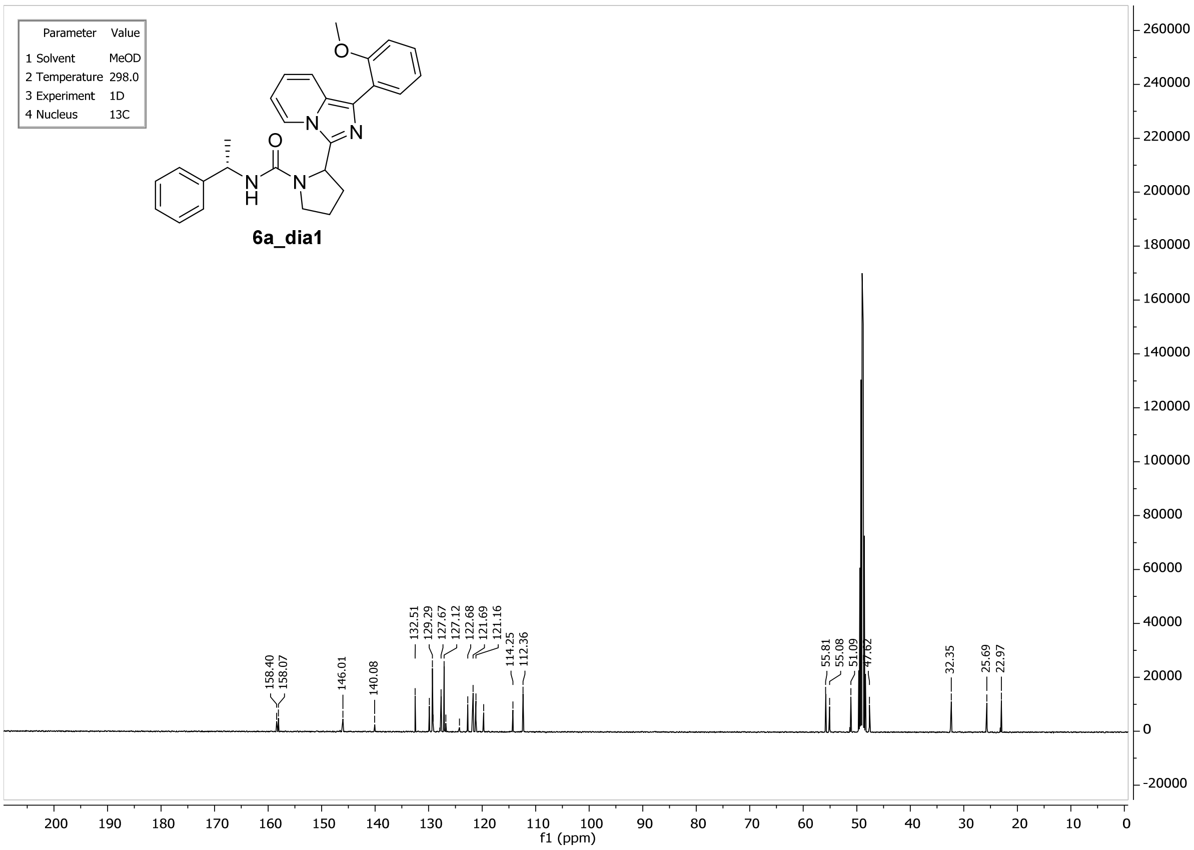
Parameter Value
1 Solvent MeOD
2 Temperature 298.0
3 Experiment 1D
4 Nucleus 13C
6a_dia1
158.40
158.07
146.01
140.08
132.51
129.29
127.67
127.12
122.68
121.69
121.16
114.25
112.36
55.81
55.08
51.09
47.62
32.35
25.69
22.97
f1 (ppm)

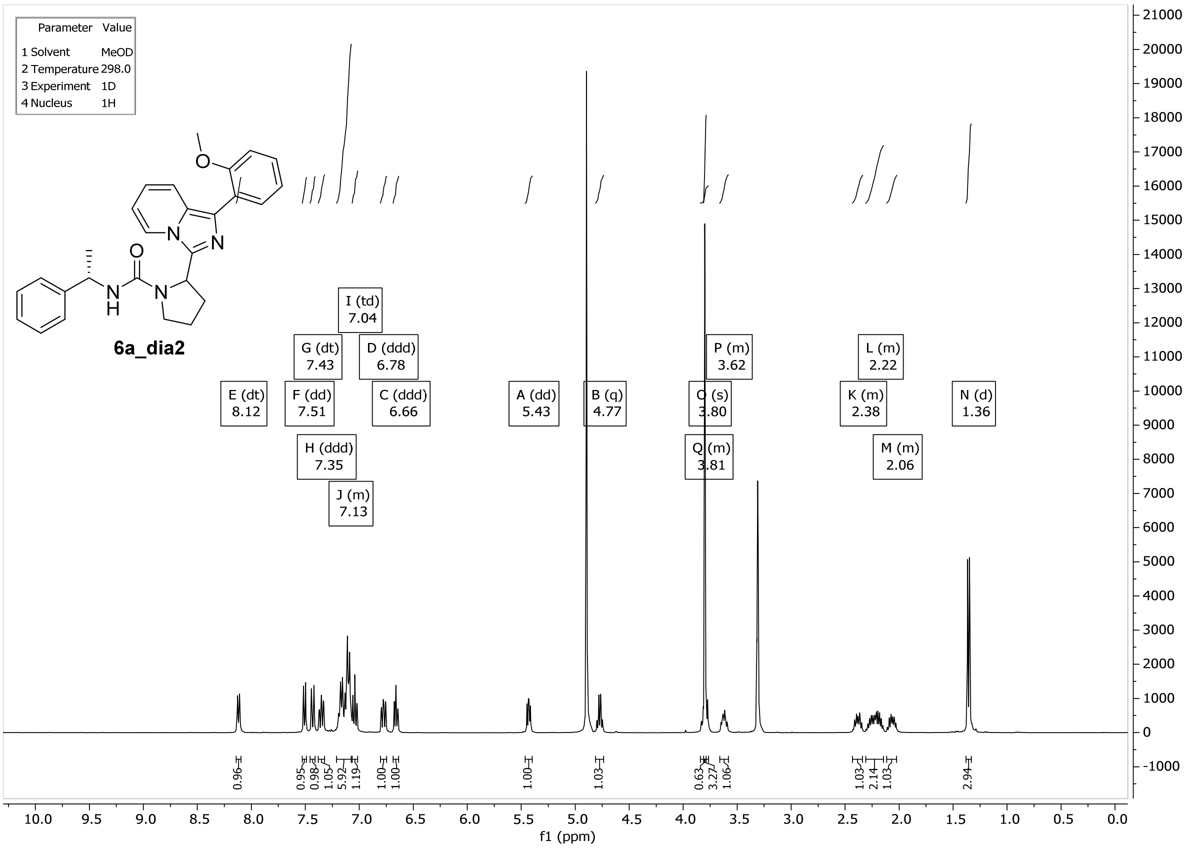
Parameter Value
1 Solvent MeOD
2 Temperature 298.0
3 Experiment 1D
4 Nucleus 1H
6a_dia2
I (td) 7.04
G (dt) 7.43
D (ddd) 6.78
P (m) 3.62
L (m) 2.22
E (dt) 8.12
F (dd) 7.51
C (ddd) 6.66
A (dd) 5.43
B (q) 4.77
O (s) 3.80
K (m) 2.38
N (d) 1.36
H (ddd) 7.35
Q (m) 3.81
M (m) 2.06
J (m) 7.13
f1 (ppm)

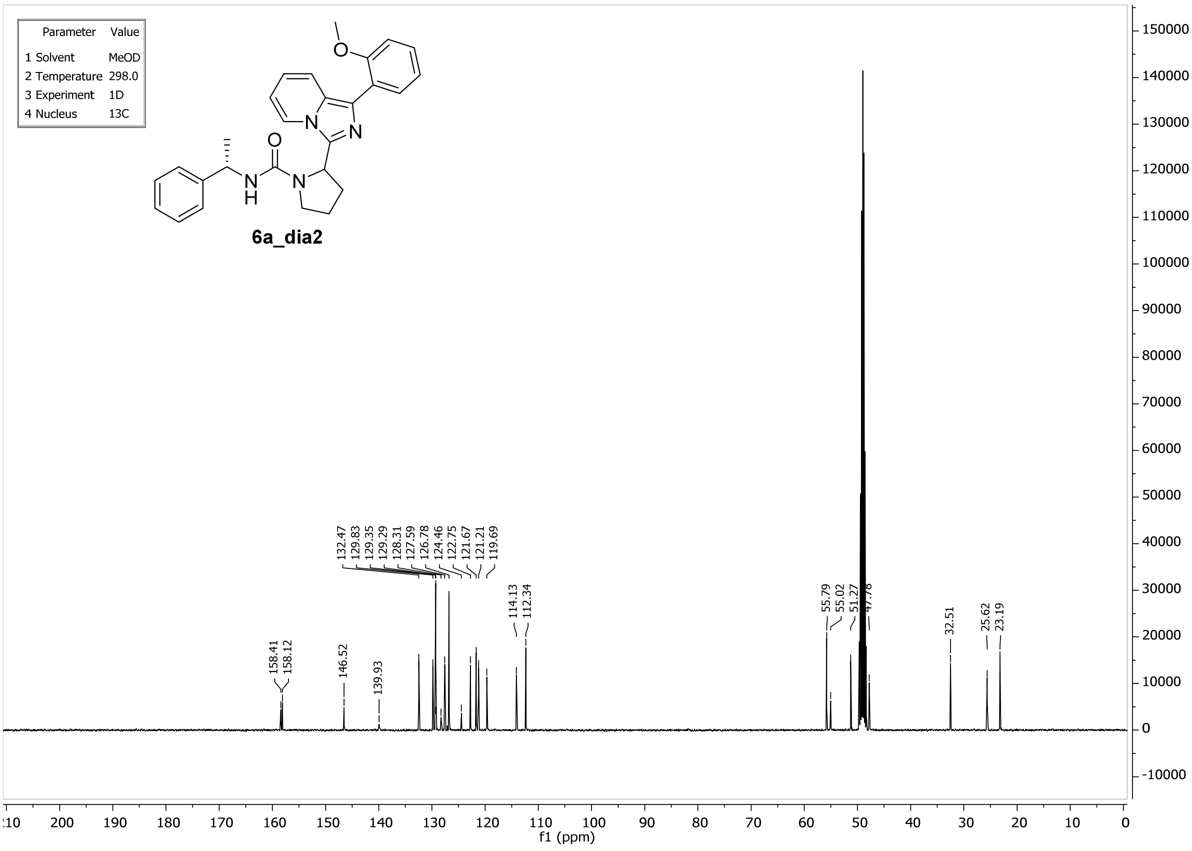
Parameter Value
1 Solvent MeOD
2 Temperature 298.0
3 Experiment 1D
4 Nucleus 13C
6a_dia2
158.41
158.12
146.52
139.93
132.47
129.83
129.35
129.29
128.31
127.59
126.78
124.46
122.75
121.67
121.21
119.69
114.13
112.34
55.79
55.02
51.27
47.78
32.51
25.62
23.19
f1 (ppm)

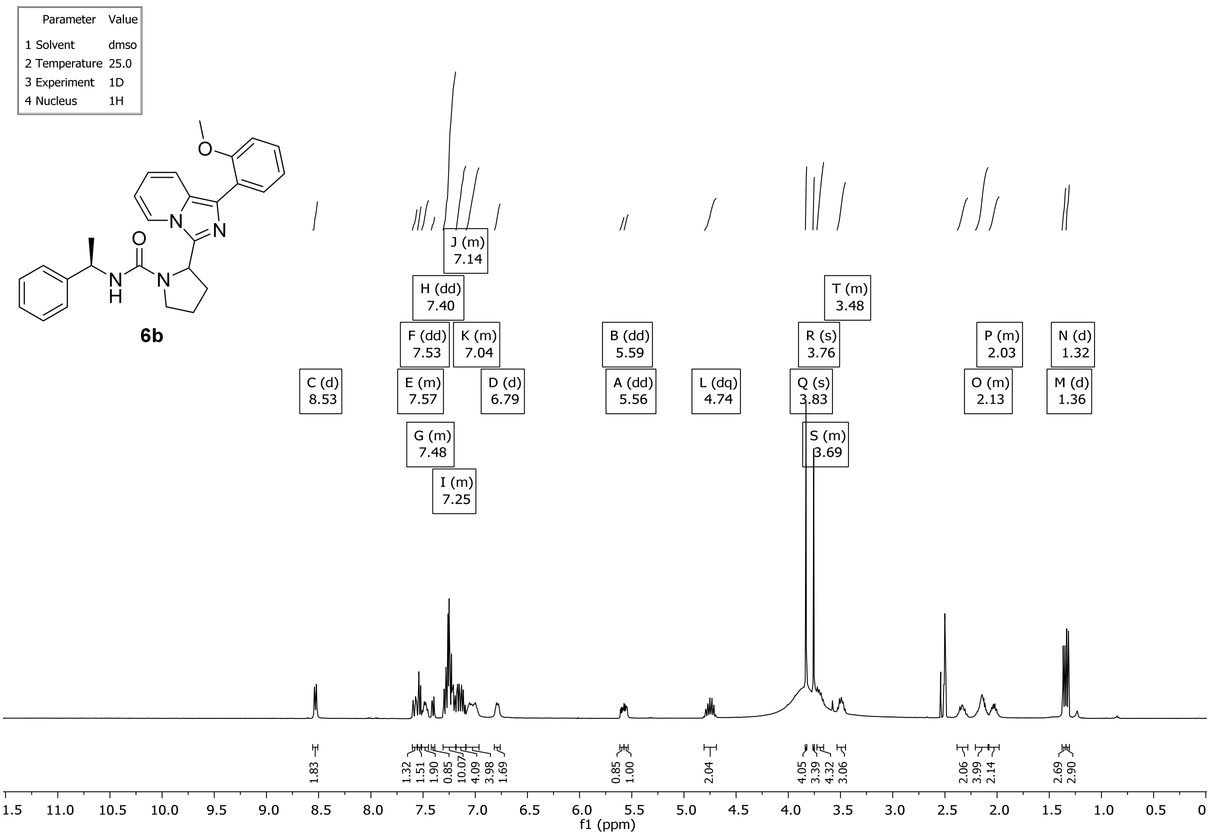
Parameter Value
1 Solvent dmso
2 Temperature 25.0
3 Experiment 1D
4 Nucleus 1H
6b
C (d) 8.53
E (m) 7.57
F (dd) 7.53
G (m) 7.48
H (dd) 7.40
I (m) 7.25
J (m) 7.14
K (m) 7.04
D (d) 6.79
B (dd) 5.59
A (dd) 5.56
L (dq) 4.74
Q (s) 3.83
R (s) 3.76
S (m) 3.69
T (m) 3.48
O (m) 2.13
P (m) 2.03
M (d) 1.36
N (d) 1.32
1.83
1.32 1.51 1.90 0.85 10.07 4.09 3.98 1.69
0.85 1.00
2.04
4.05 3.39 4.32 3.06
2.06 3.99 2.14
2.69 2.90
f1 (ppm)

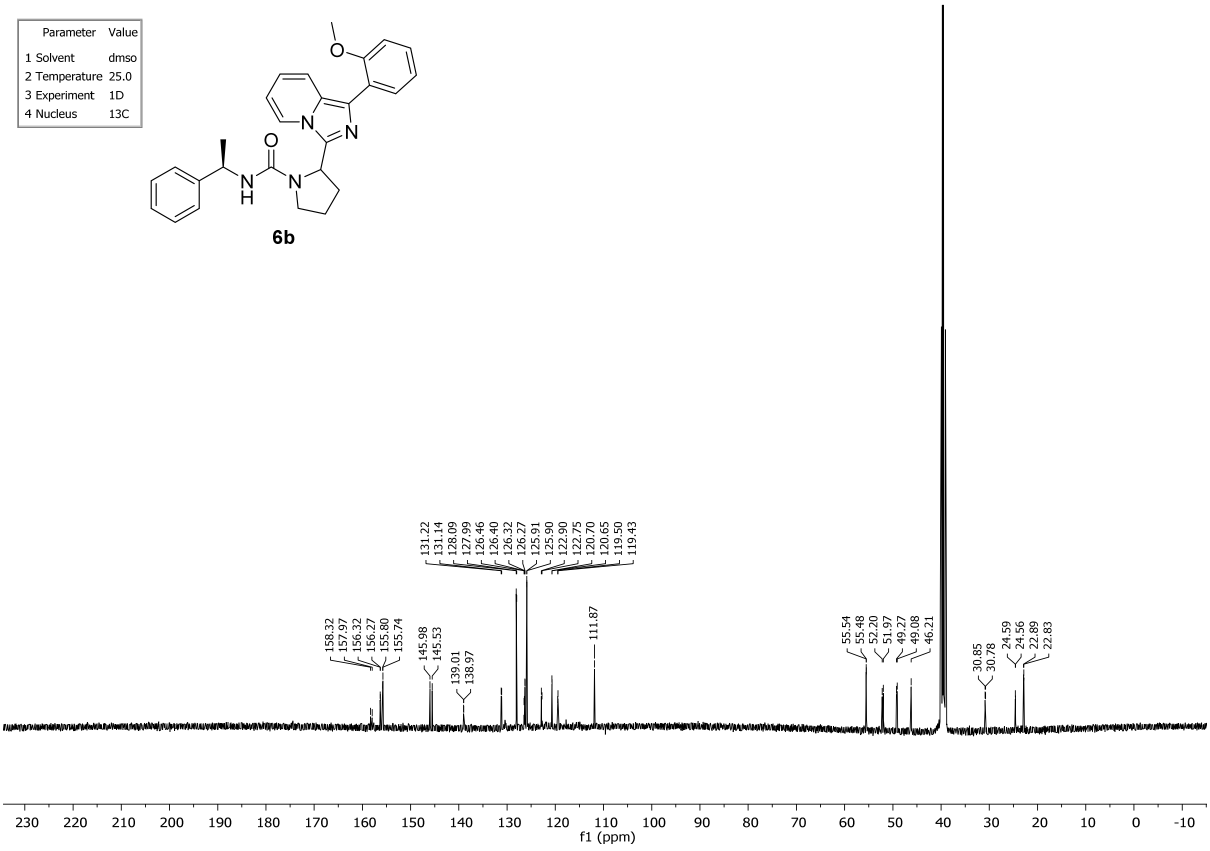
Parameter Value
1 Solvent dmso
2 Temperature 25.0
3 Experiment 1D
4 Nucleus 13C
6b
158.32 157.97 156.32 156.27 155.80 155.74
145.98 145.53
139.01 138.97
131.22 131.14 128.09 127.99 126.46 126.40 126.32 126.27 125.91 125.90 122.90 122.75 120.70 120.65 119.50 119.43
111.87
55.54 55.48 52.20 51.97 49.27 49.08 46.21
30.85 30.78
24.59 24.56 22.89 22.83
f1 (ppm)

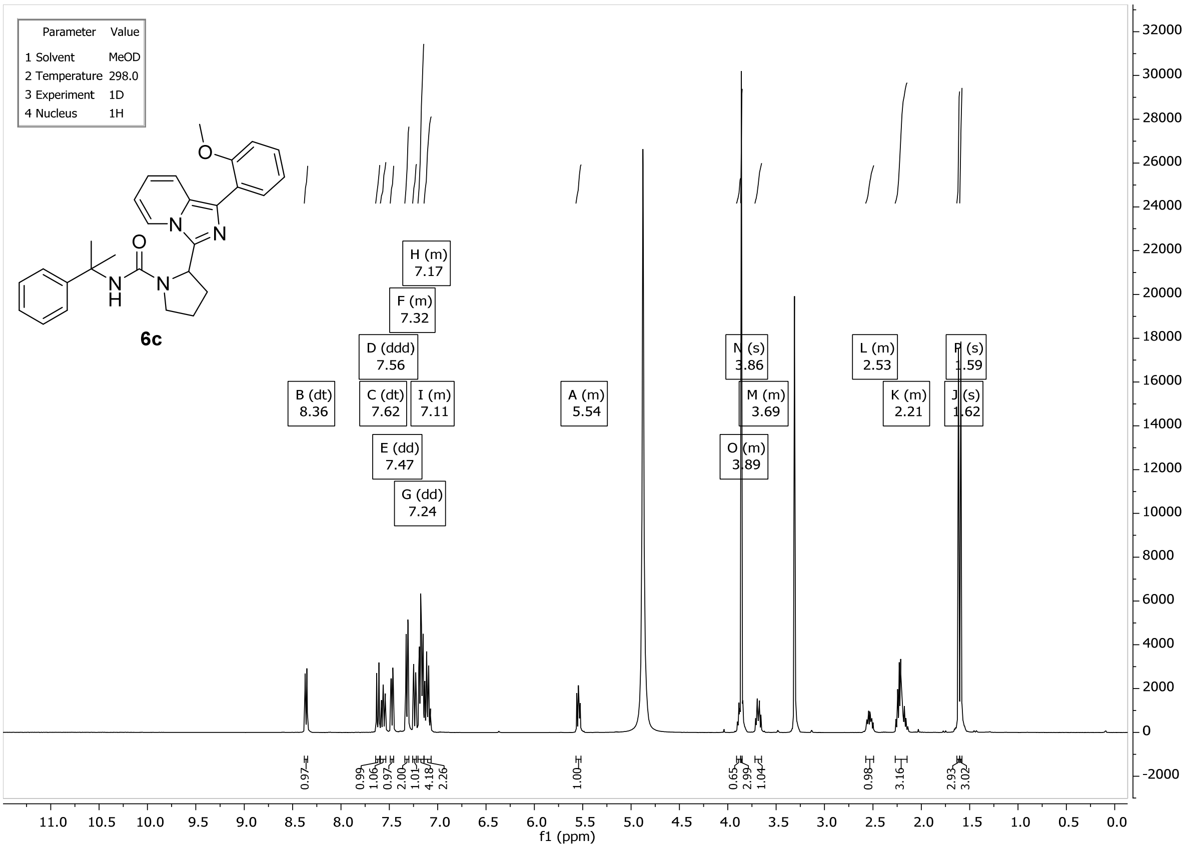
Parameter Value
1 Solvent MeOD
2 Temperature 298.0
3 Experiment 1D
4 Nucleus 1H
6c
B (dt) 8.36
C (dt) 7.62
D (ddd) 7.56
E (dd) 7.47
F (m) 7.32
G (dd) 7.24
H (m) 7.17
I (m) 7.11
A (m) 5.54
O (m) 3.89
N (s) 3.86
M (m) 3.69
L (m) 2.53
K (m) 2.21
J (s) 1.62
P (s) 1.59
f1 (ppm)

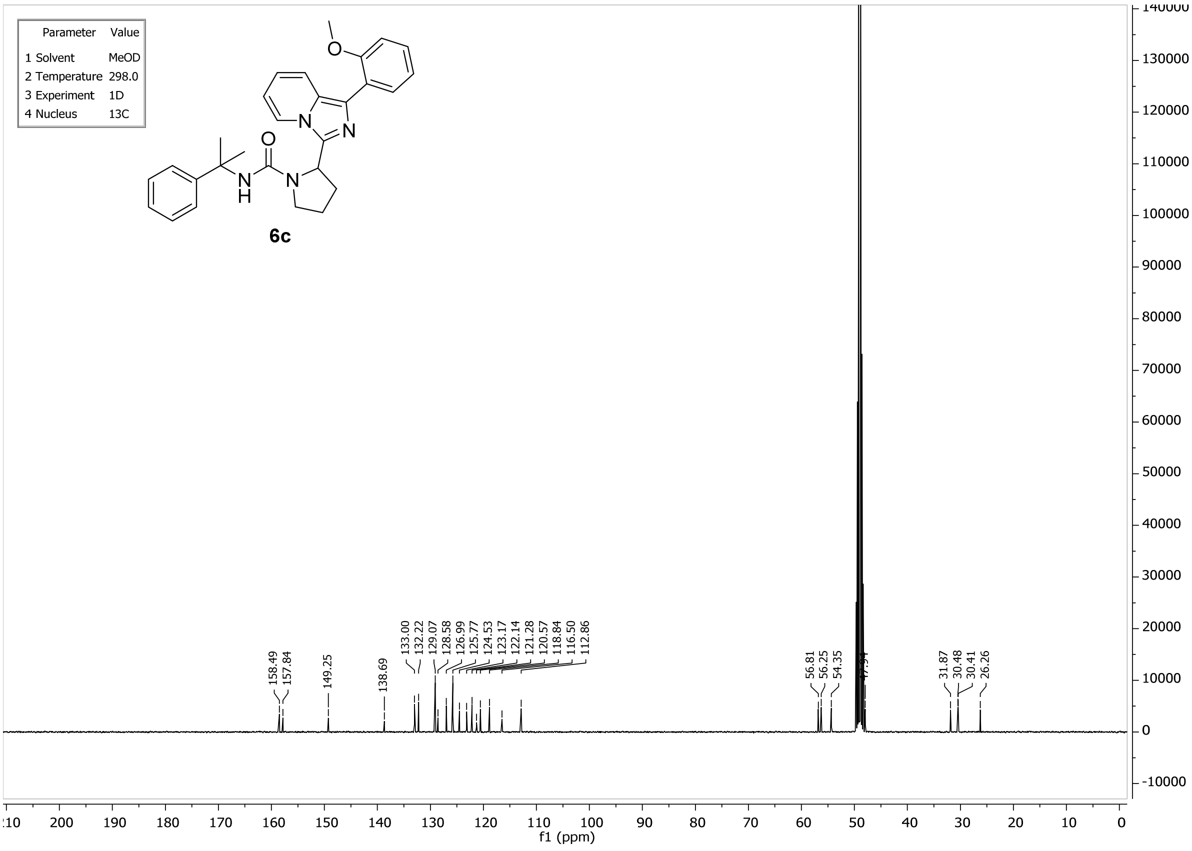
Parameter Value
1 Solvent MeOD
2 Temperature 298.0
3 Experiment 1D
4 Nucleus 13C
6c
158.49
157.84
149.25
138.69
133.00
132.22
129.07
128.58
126.99
125.77
124.53
123.17
122.14
121.28
120.57
118.84
116.50
112.86
56.81
56.25
54.35
31.87
30.48
30.41
26.26
f1 (ppm)

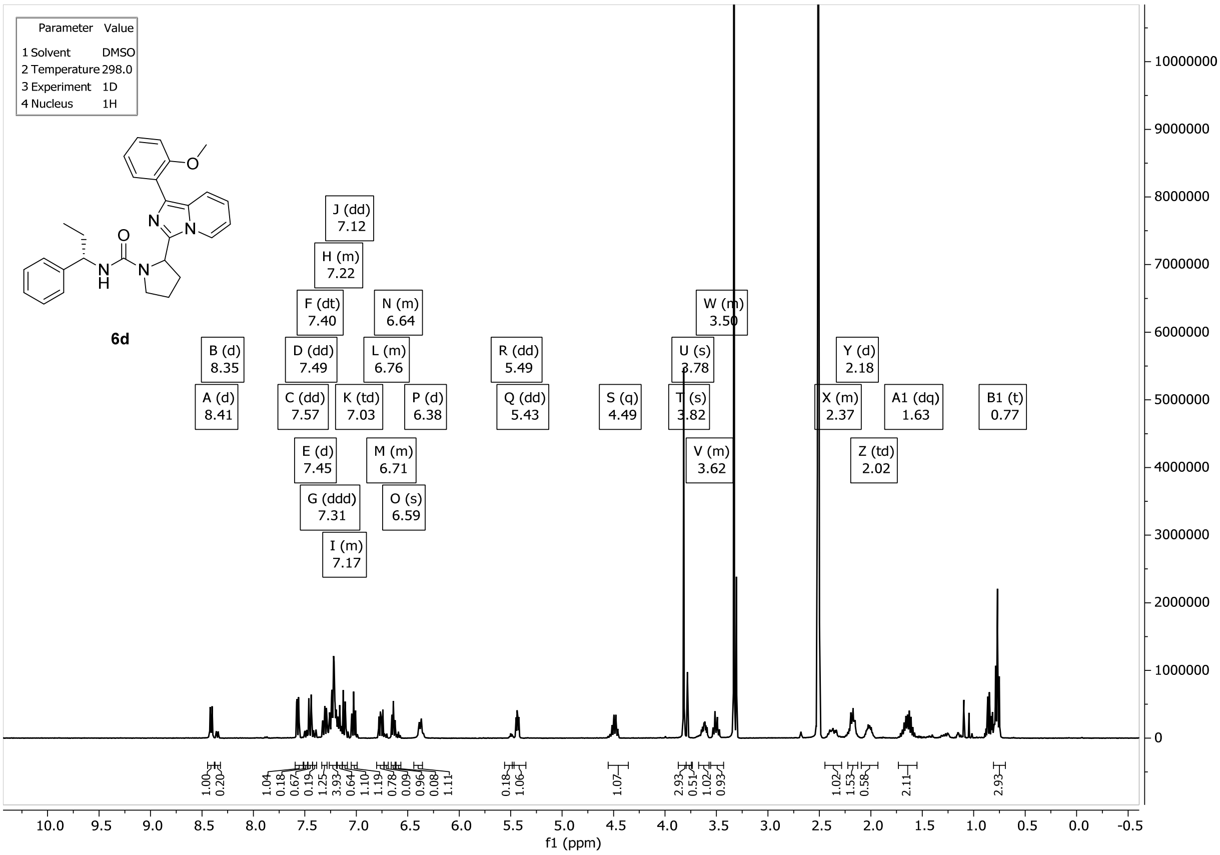

Parameter Value
1 Solvent DMSO
2 Temperature 298.0
3 Experiment 1D
4 Nucleus 1H
6d
A (d) 8.41
B (d) 8.35
C (dd) 7.57
D (dd) 7.49
E (d) 7.45
F (dt) 7.40
G (ddd) 7.31
H (m) 7.22
I (m) 7.17
J (dd) 7.12
K (td) 7.03
L (m) 6.76
M (m) 6.71
N (m) 6.64
O (s) 6.59
P (d) 6.38
Q (dd) 5.43
R (dd) 5.49
S (q) 4.49
T (s) 3.82
U (s) 3.78
V (m) 3.62
W (m) 3.50
X (m) 2.37
Y (d) 2.18
Z (td) 2.02
A1 (dq) 1.63
B1 (t) 0.77
f1 (ppm)

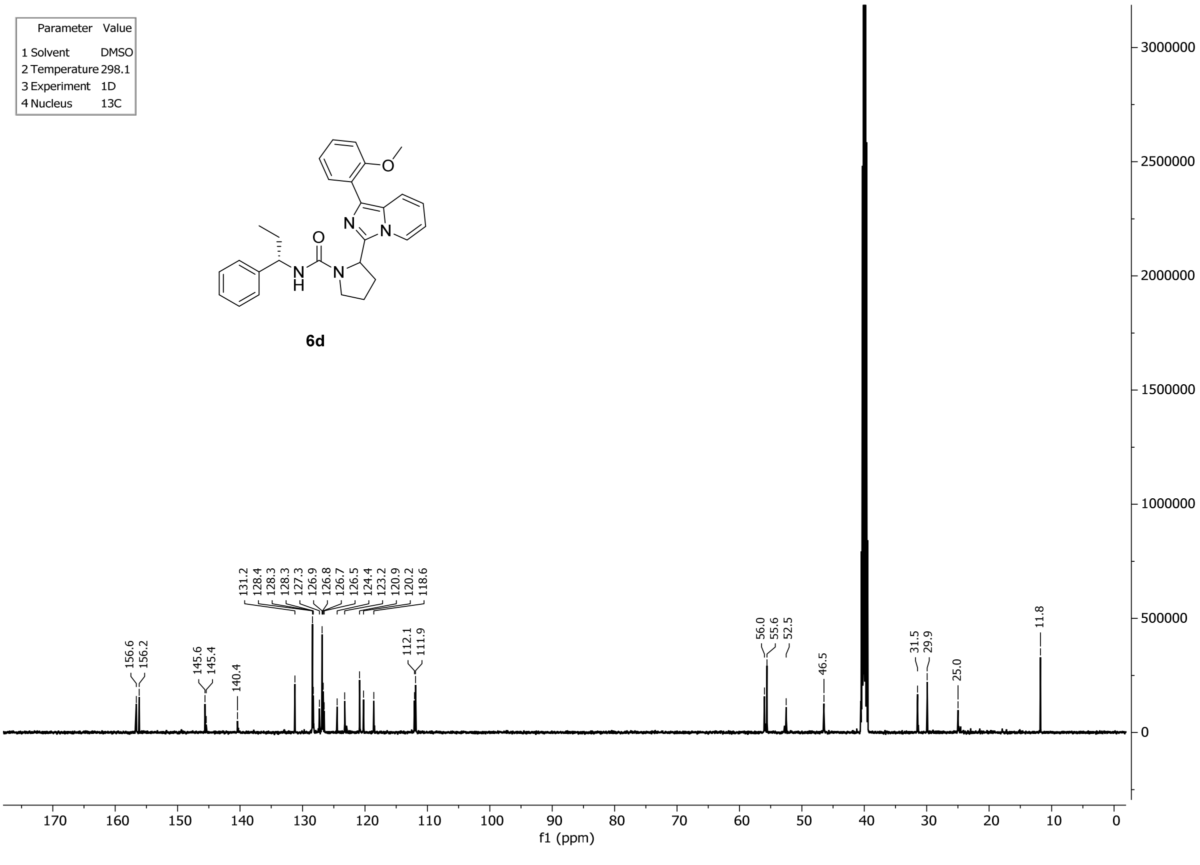

Parameter Value
1 Solvent DMSO
2 Temperature 298.1
3 Experiment 1D
4 Nucleus 13C
6d
156.6
156.2
145.6
145.4
140.4
131.2
128.4
128.3
128.3
127.3
126.9
126.8
126.7
126.5
124.4
123.2
120.9
120.2
118.6
112.1
111.9
56.0
55.6
52.5
46.5
31.5
29.9
25.0
11.8
f1 (ppm)

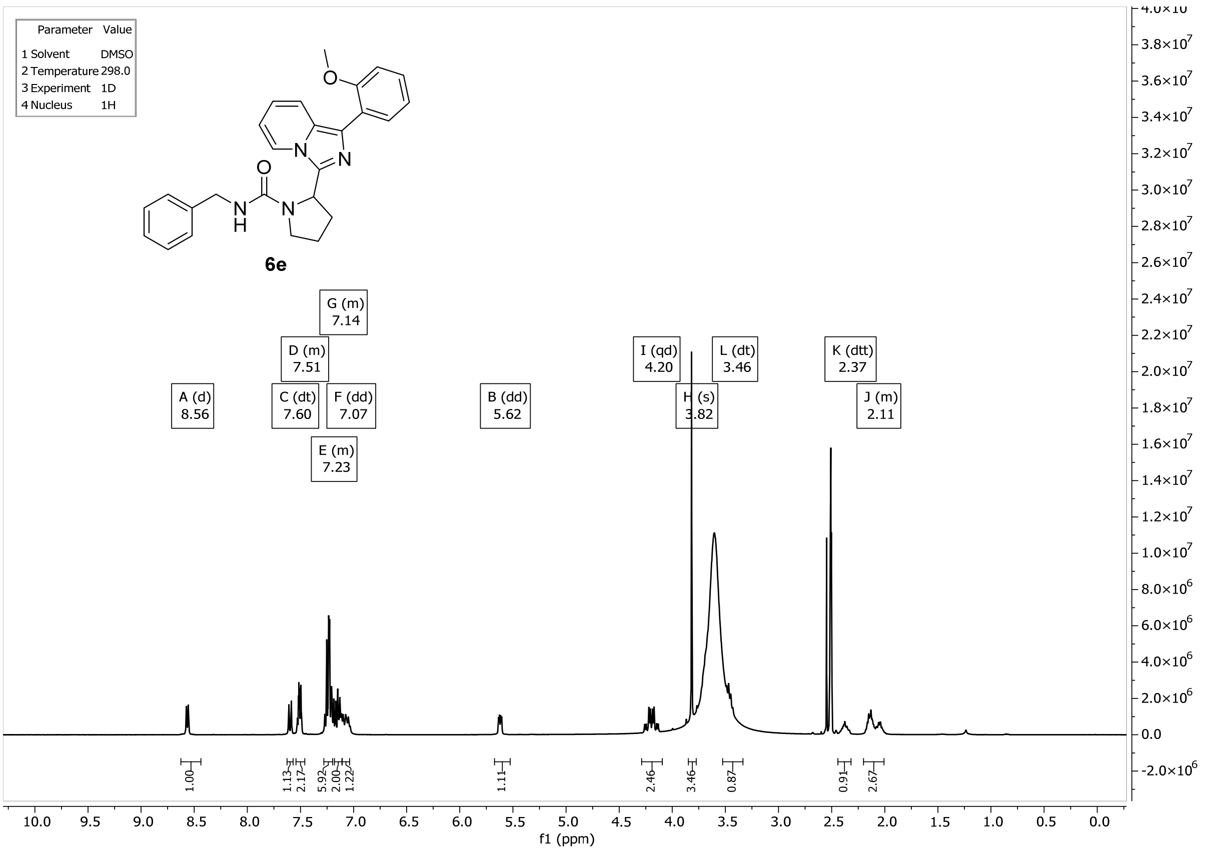
Parameter Value
1 Solvent DMSO
2 Temperature 298.0
3 Experiment 1D
4 Nucleus 1H
O
N
N
O
N
H
N
6e
G (m) 7.14
D (m) 7.51
I (qd) 4.20
L (dt) 3.46
K (dtt) 2.37
A (d) 8.56
C (dt) 7.60
F (dd) 7.07
B (dd) 5.62
H (s) 3.82
J (m) 2.11
E (m) 7.23
1.00
1.13
2.17
5.92
2.00
1.22
1.11
2.46
3.46
0.87
0.91
2.67
10.0 9.5 9.0 8.5 8.0 7.5 7.0 6.5 6.0 5.5 5.0 4.5 4.0 3.5 3.0 2.5 2.0 1.5 1.0 0.5 0.0
f1 (ppm)

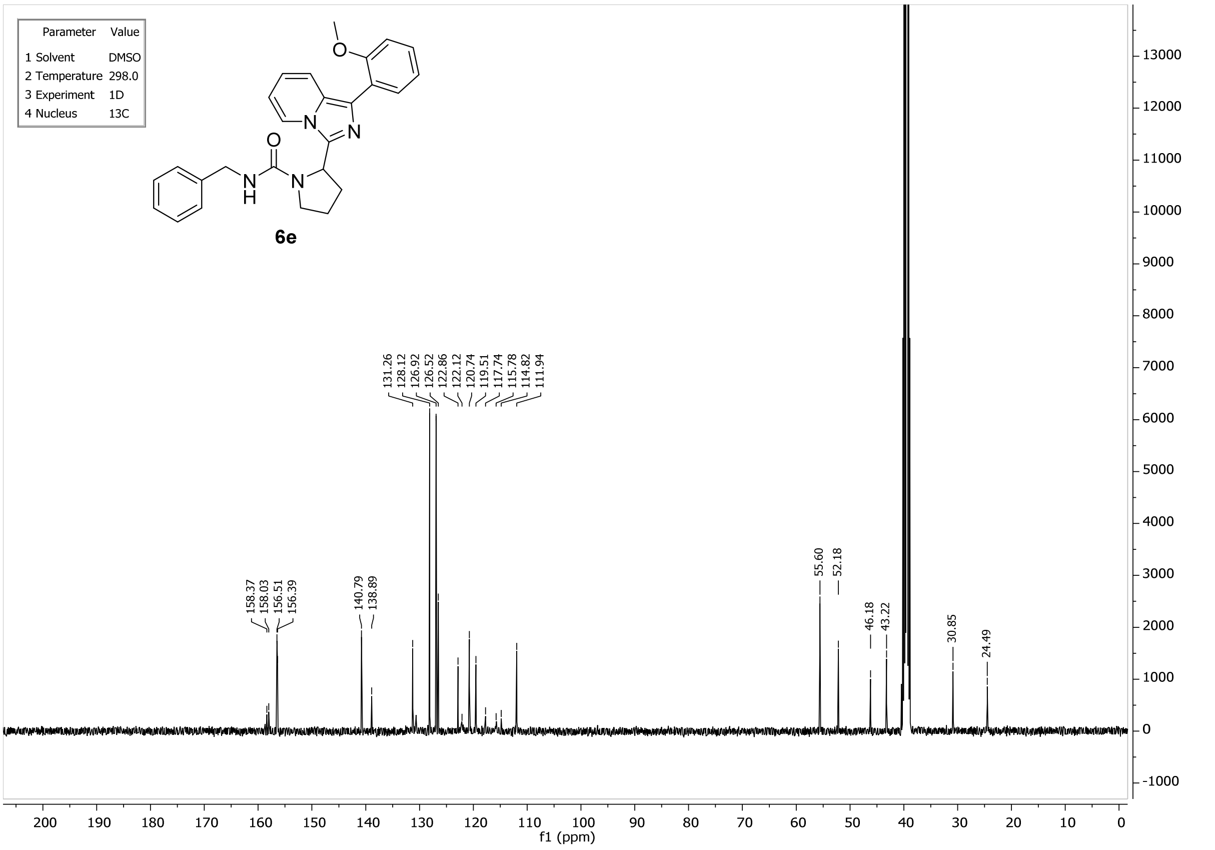
Parameter Value
1 Solvent DMSO
2 Temperature 298.0
3 Experiment 1D
4 Nucleus 13C
O
N
N
O
N
H
N
6e
131.26
128.12
126.92
126.52
122.86
122.12
120.74
119.51
117.74
115.78
114.82
111.94
158.37
158.03
156.51
156.39
140.79
138.89
55.60
52.18
46.18
43.22
30.85
24.49
200 190 180 170 160 150 140 130 120 110 100 90 80 70 60 50 40 30 20 10 0
f1 (ppm)

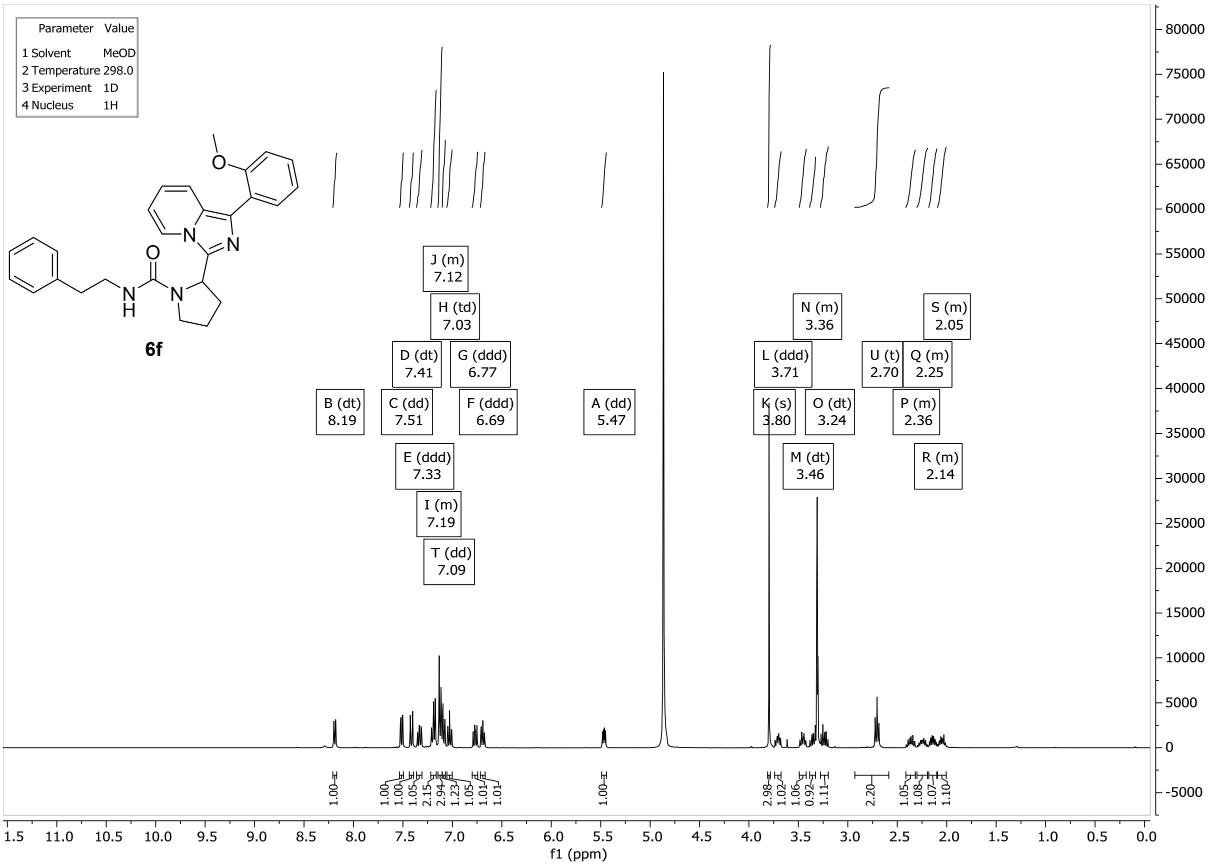

Parameter Value
1 Solvent MeOD
2 Temperature 298.0
3 Experiment 1D
4 Nucleus 1H
6f
J (m) 7.12
H (td) 7.03
D (dt) 7.41
G (ddd) 6.77
B (dt) 8.19
C (dd) 7.51
F (ddd) 6.69
A (dd) 5.47
E (ddd) 7.33
I (m) 7.19
T (dd) 7.09
N (m) 3.36
S (m) 2.05
L (ddd) 3.71
U (t) 2.70
Q (m) 2.25
K (s) 3.80
O (dt) 3.24
P (m) 2.36
M (dt) 3.46
R (m) 2.14
f1 (ppm)

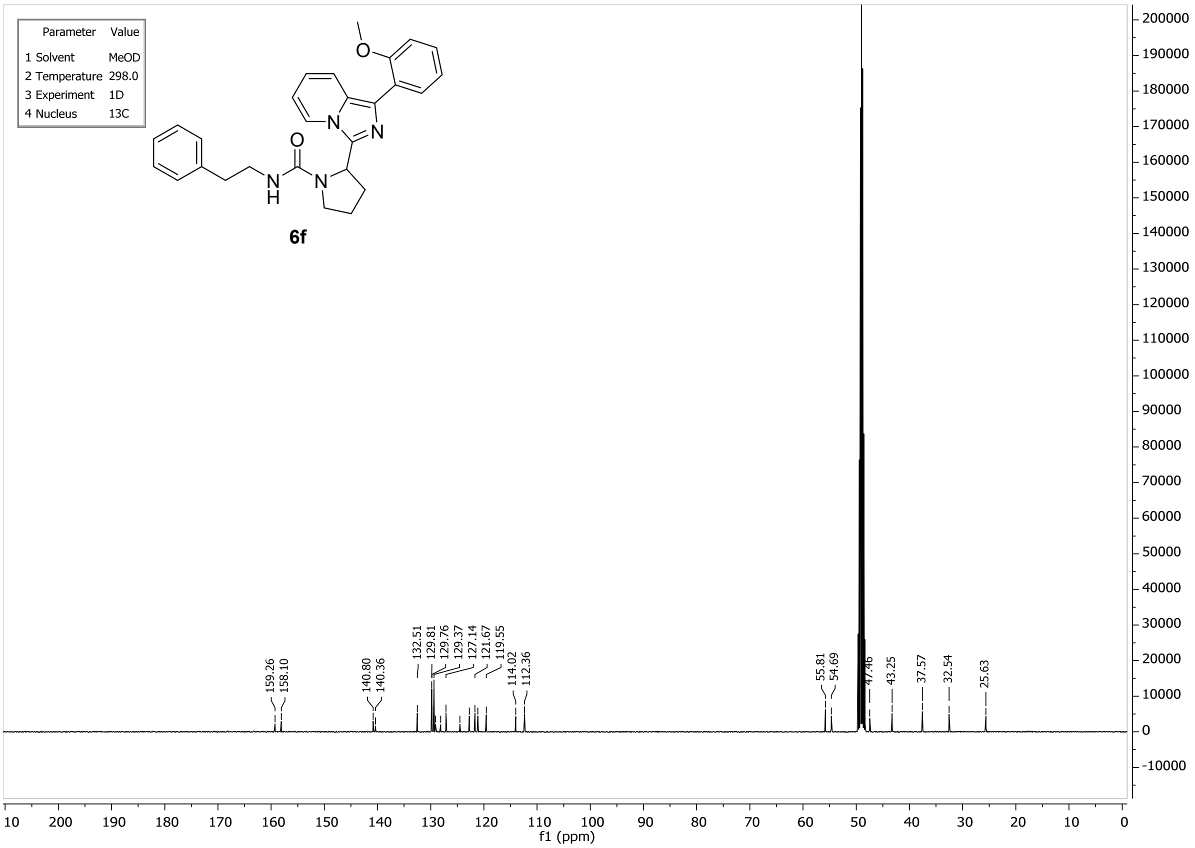

Parameter Value
1 Solvent MeOD
2 Temperature 298.0
3 Experiment 1D
4 Nucleus 13C
6f
159.26
158.10
140.80
140.36
132.51
129.81
129.76
129.37
127.14
121.67
119.55
114.02
112.36
55.81
54.69
47.46
43.25
37.57
32.54
25.63
f1 (ppm)

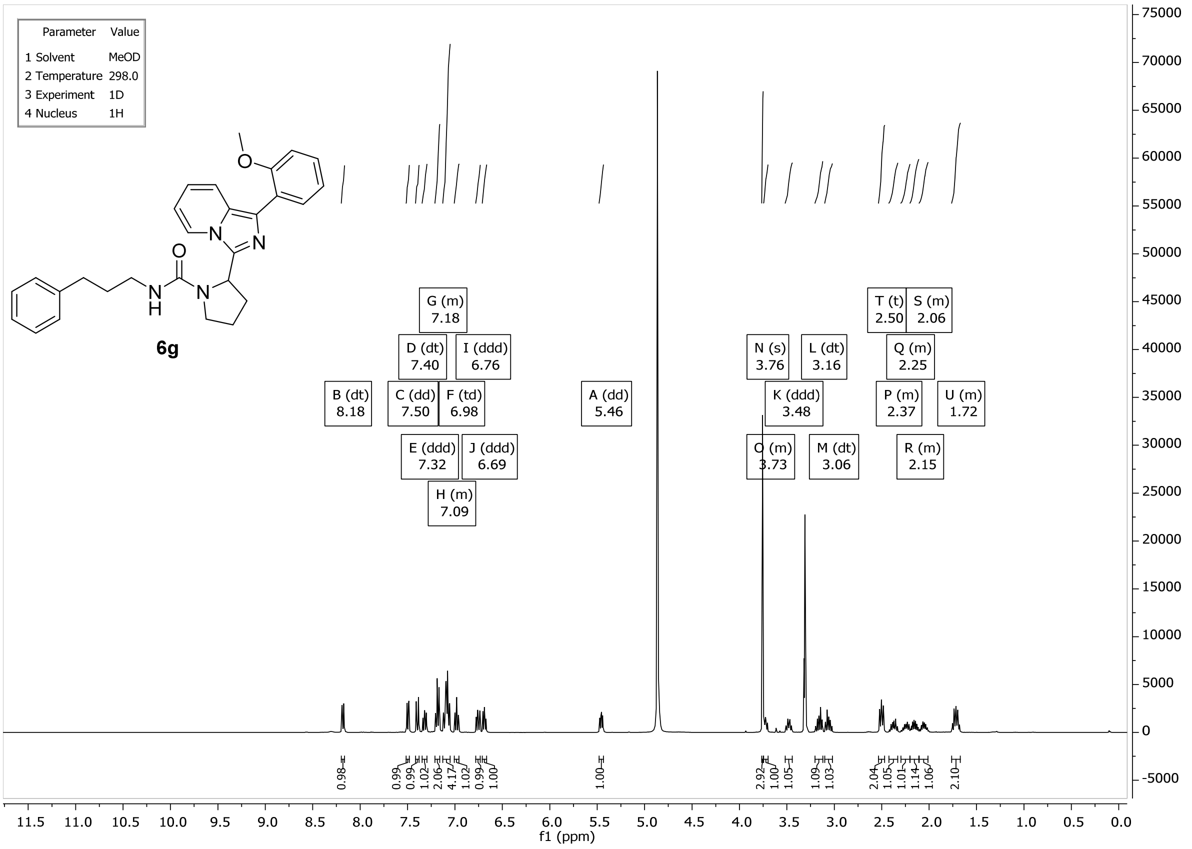
Parameter Value
1 Solvent MeOD
2 Temperature 298.0
3 Experiment 1D
4 Nucleus 1H
6g
G (m) 7.18
D (dt) 7.40
I (ddd) 6.76
B (dt) 8.18
C (dd) 7.50
F (td) 6.98
E (ddd) 7.32
J (ddd) 6.69
H (m) 7.09
A (dd) 5.46
N (s) 3.76
L (dt) 3.16
K (ddd) 3.48
O (m) 3.73
M (dt) 3.06
T (t) 2.50
S (m) 2.06
Q (m) 2.25
P (m) 2.37
U (m) 1.72
R (m) 2.15
0.98
0.99
0.99
1.02
2.06
4.17
1.02
0.99
1.00
1.00
2.92
1.00
1.05
1.09
1.03
2.04
1.05
1.01
1.14
1.06
2.10
f1 (ppm)

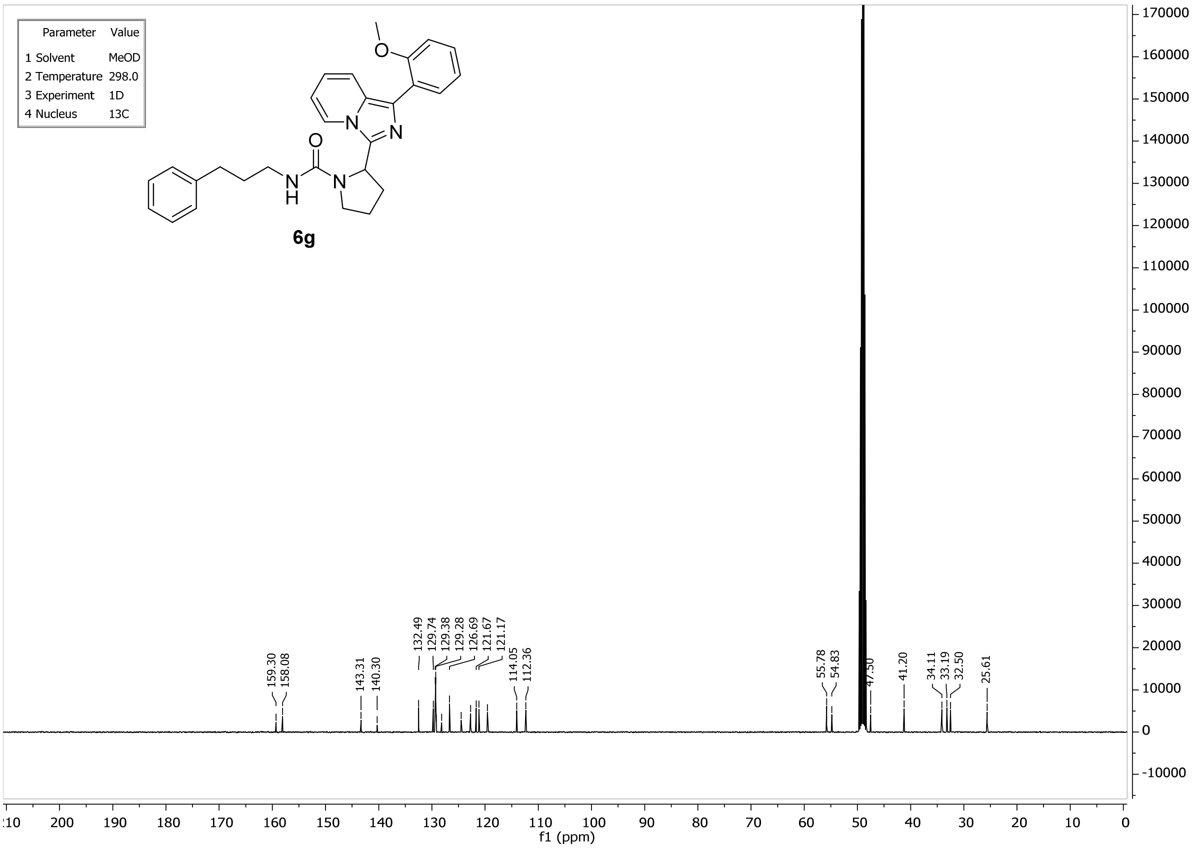
Parameter Value
1 Solvent MeOD
2 Temperature 298.0
3 Experiment 1D
4 Nucleus 13C
6g
159.30
158.08
143.31
140.30
132.49
129.74
129.38
129.28
126.69
121.67
121.17
114.05
112.36
55.78
54.83
47.50
41.20
34.11
33.19
32.50
25.61
f1 (ppm)

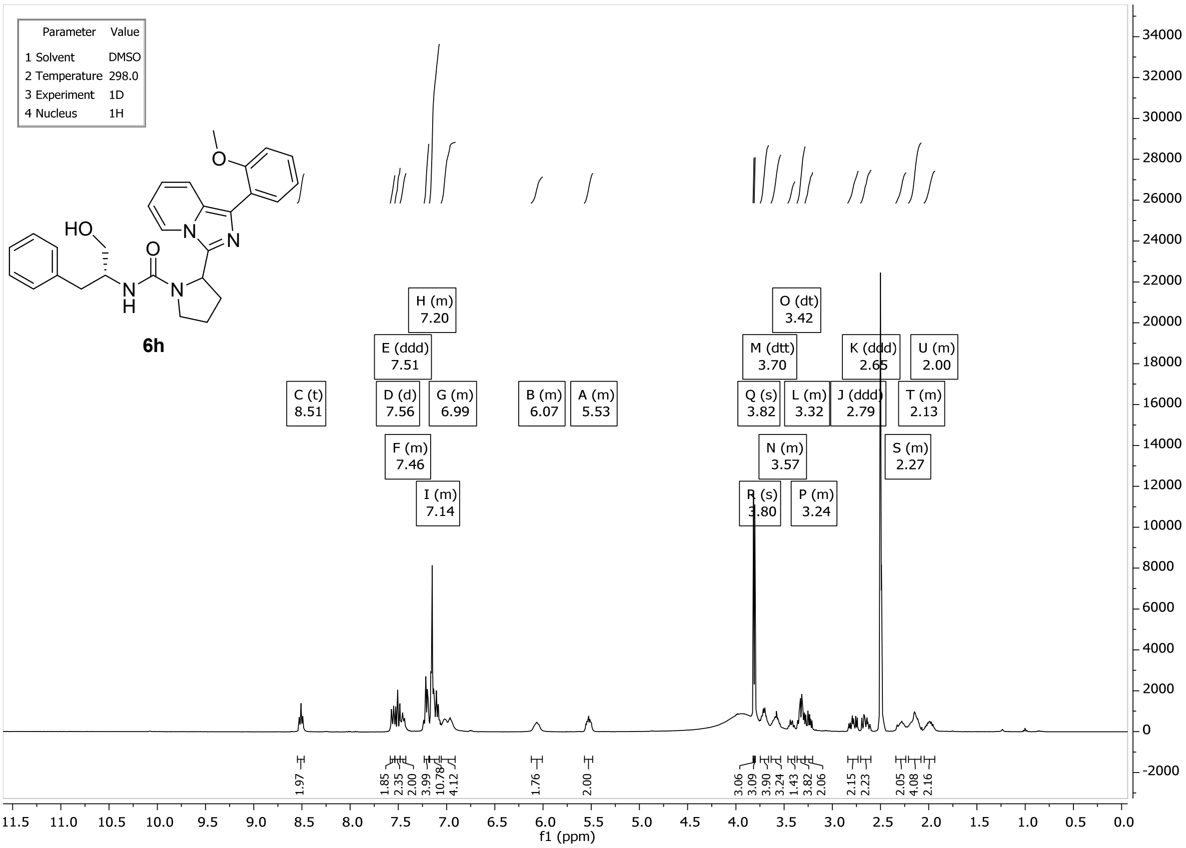
Parameter Value
1 Solvent DMSO
2 Temperature 298.0
3 Experiment 1D
4 Nucleus 1H
HO
O
N
H
N
N
N
O
6h
H (m) 7.20
E (ddd) 7.51
C (t) 8.51
D (d) 7.56
G (m) 6.99
B (m) 6.07
A (m) 5.53
F (m) 7.46
I (m) 7.14
O (dt) 3.42
M (dtt) 3.70
K (ddd) 2.65
U (m) 2.00
Q (s) 3.82
L (m) 3.32
J (ddd) 2.79
T (m) 2.13
N (m) 3.57
S (m) 2.27
R (s) 3.80
P (m) 3.24
f1 (ppm)

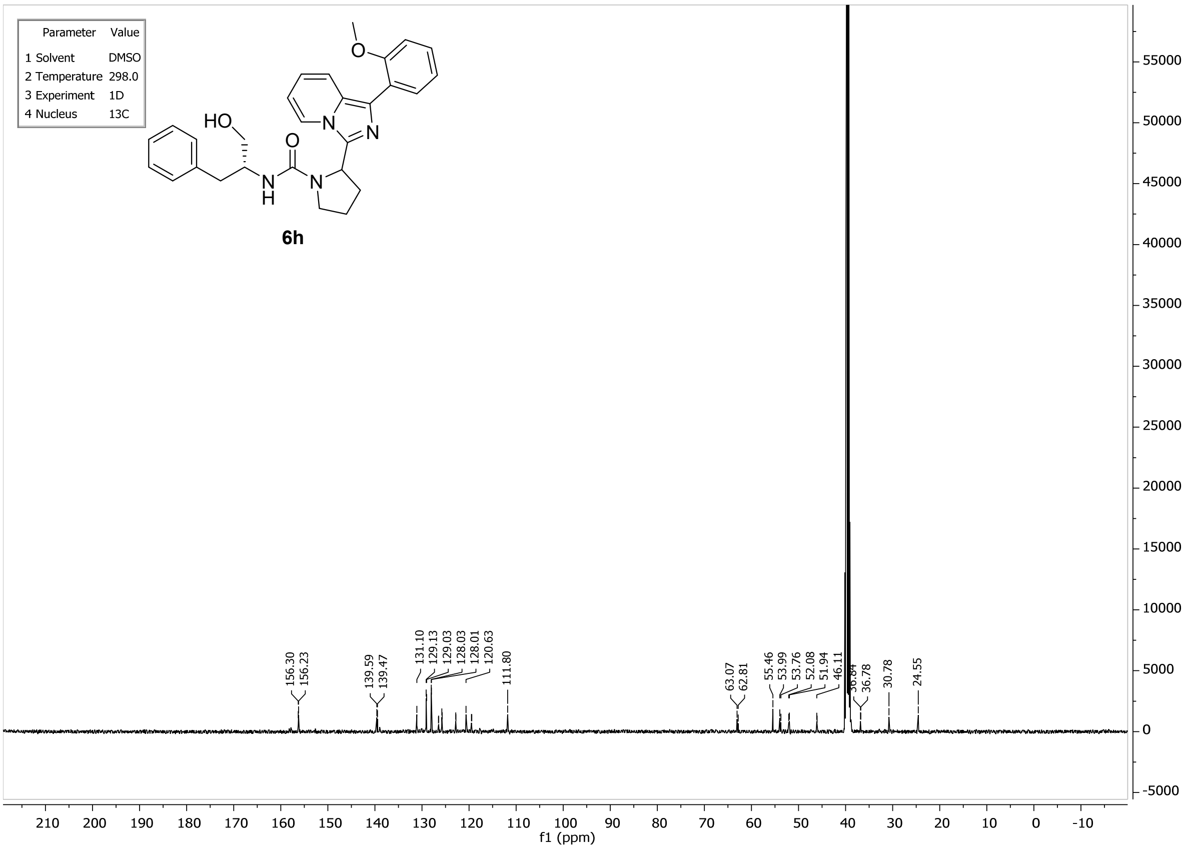
Parameter Value
1 Solvent DMSO
2 Temperature 298.0
3 Experiment 1D
4 Nucleus 13C
HO
O
N
H
N
N
N
O
6h
156.30
156.23
139.59
139.47
131.10
129.13
129.03
128.03
128.01
120.63
111.80
63.07
62.81
55.46
53.99
53.76
52.08
51.94
46.11
36.78
30.78
24.55
f1 (ppm)

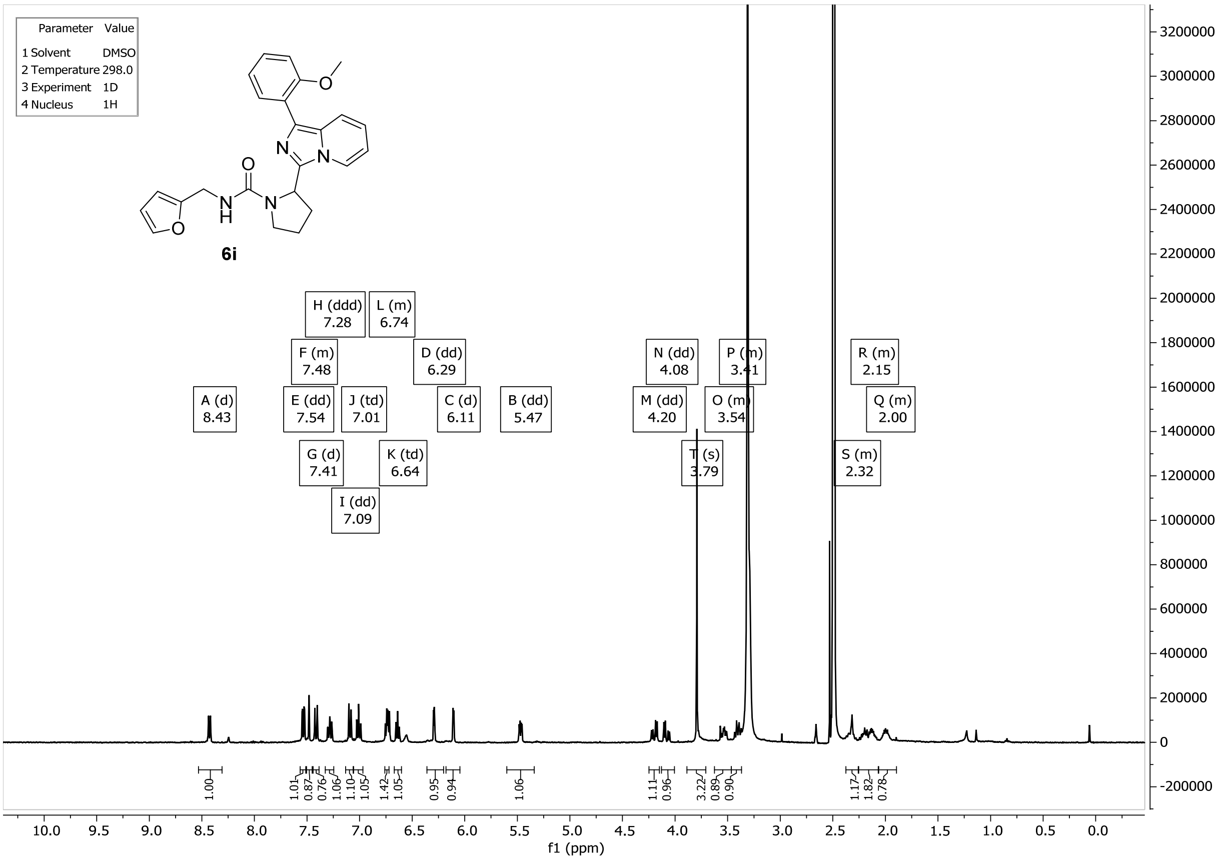

Parameter Value
1 Solvent DMSO
2 Temperature 298.0
3 Experiment 1D
4 Nucleus 1H
6i
A (d) 8.43
B (dd) 5.47
C (d) 6.11
D (dd) 6.29
E (dd) 7.54
F (m) 7.48
G (d) 7.41
H (ddd) 7.28
I (dd) 7.09
J (td) 7.01
K (td) 6.64
L (m) 6.74
M (dd) 4.20
N (dd) 4.08
O (m) 3.54
P (m) 3.41
Q (m) 2.00
R (m) 2.15
S (m) 2.32
T (s) 3.79
1.00 1.01 0.87 0.76 1.06 1.10 1.05 1.42 1.05 0.95 0.94 1.06 1.11 0.96 3.22 0.89 0.90 1.17 1.82 0.78
f1 (ppm)

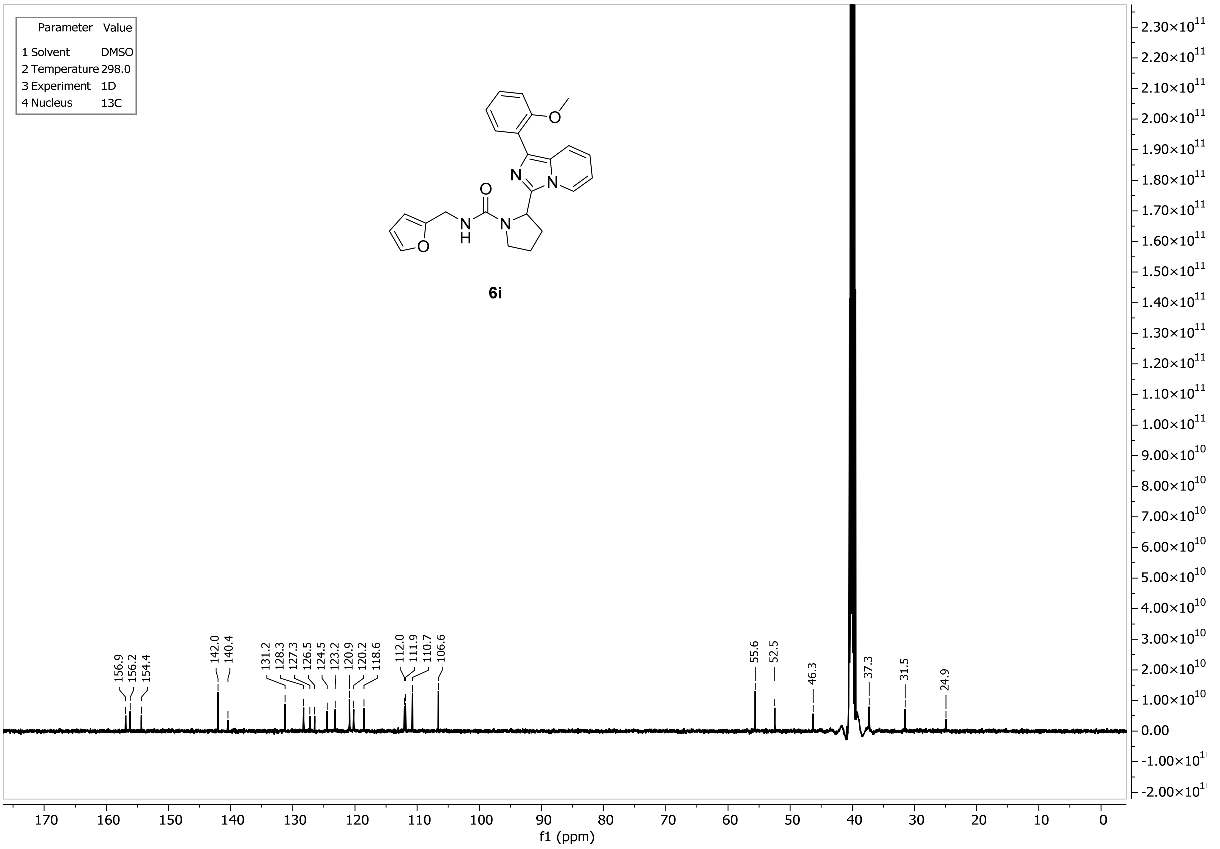

Parameter Value
1 Solvent DMSO
2 Temperature 298.0
3 Experiment 1D
4 Nucleus 13C
6i
156.9 156.2 154.4 142.0 140.4 131.2 128.3 127.3 126.5 124.5 123.2 120.9 120.2 118.6 112.0 111.9 110.7 106.6 55.6 52.5 46.3 37.3 31.5 24.9
f1 (ppm)

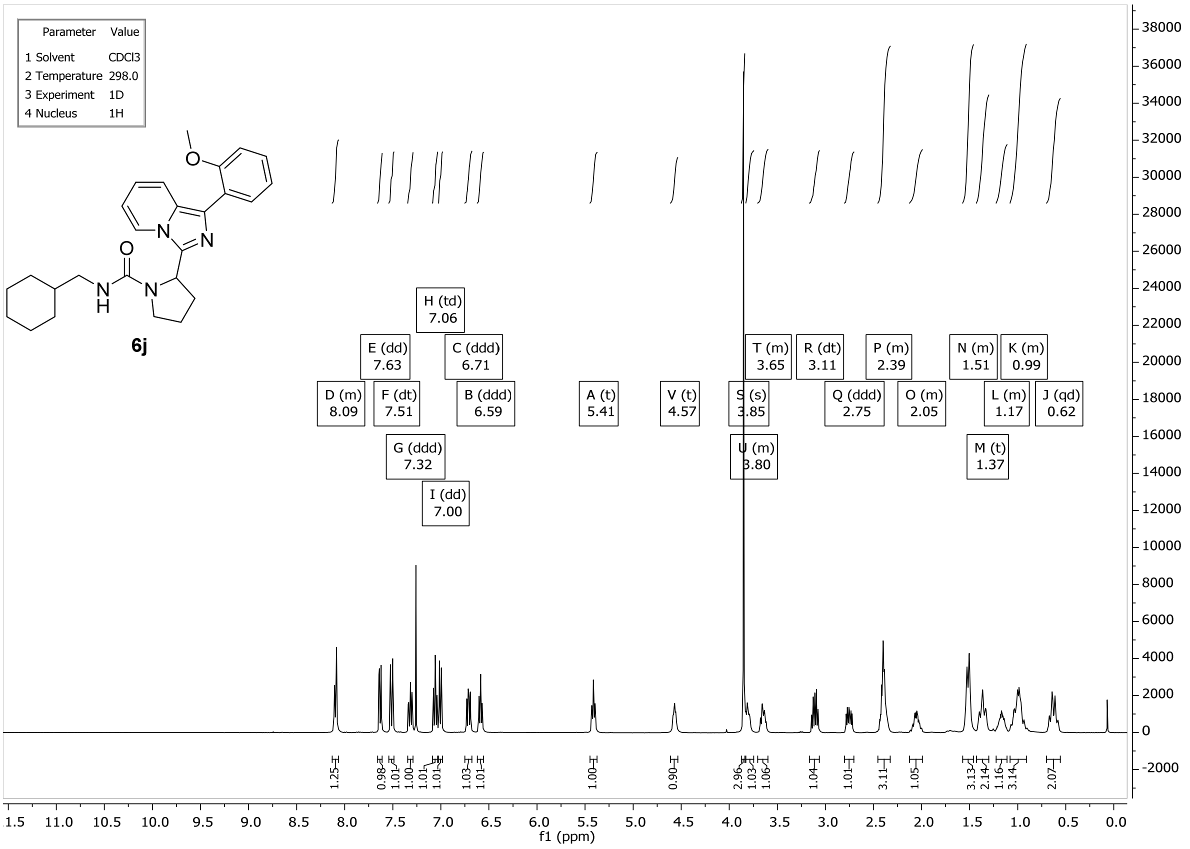
Parameter Value
1 Solvent CDCl3
2 Temperature 298.0
3 Experiment 1D
4 Nucleus 1H
6j
H (td) 7.06
E (dd) 7.63
C (ddd) 6.71
T (m) 3.65
R (dt) 3.11
P (m) 2.39
N (m) 1.51
K (m) 0.99
D (m) 8.09
F (dt) 7.51
B (ddd) 6.59
A (t) 5.41
V (t) 4.57
S (s) 3.85
Q (ddd) 2.75
O (m) 2.05
L (m) 1.17
J (qd) 0.62
G (ddd) 7.32
U (m) 3.80
M (t) 1.37
I (dd) 7.00
f1 (ppm)

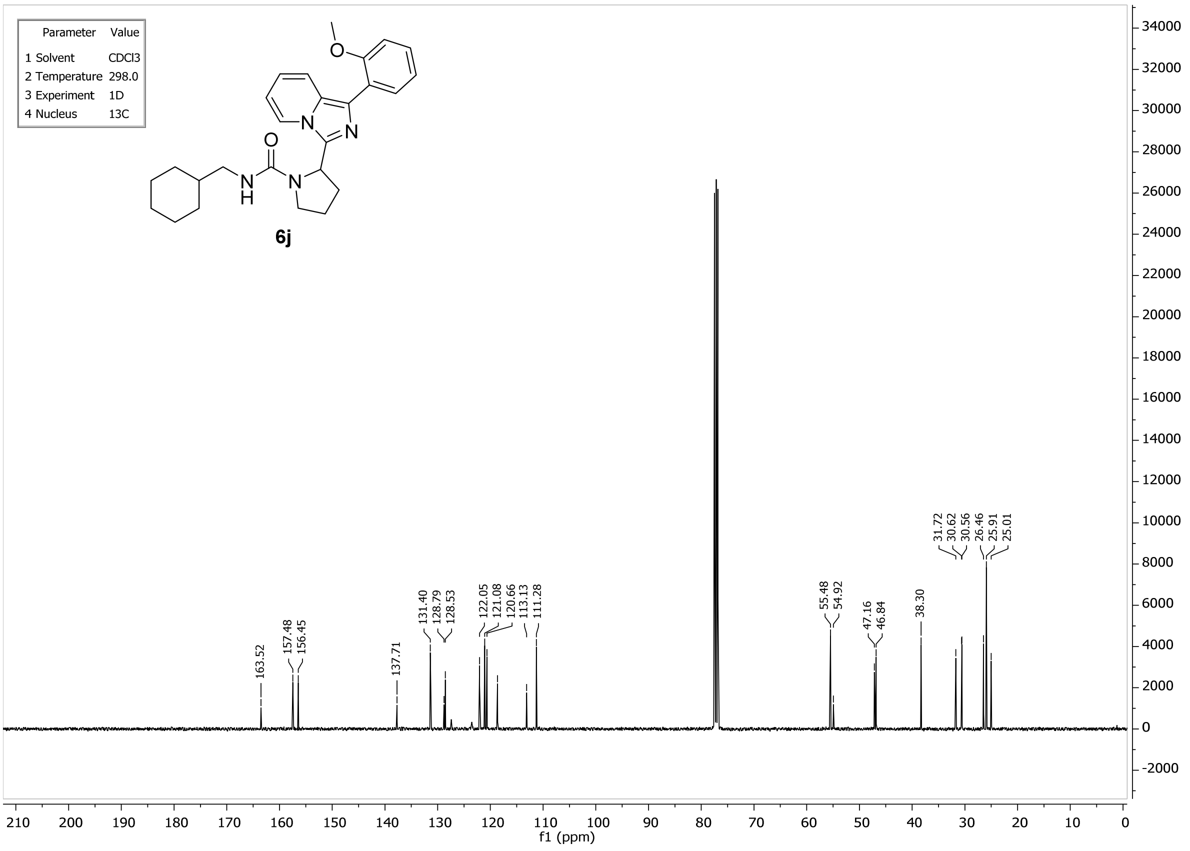
Parameter Value
1 Solvent CDCl3
2 Temperature 298.0
3 Experiment 1D
4 Nucleus 13C
6j
163.52
157.48
156.45
137.71
131.40
128.79
128.53
122.05
121.08
120.66
113.13
111.28
55.48
54.92
47.16
46.84
38.30
31.72
30.62
30.56
26.46
25.91
25.01
f1 (ppm)

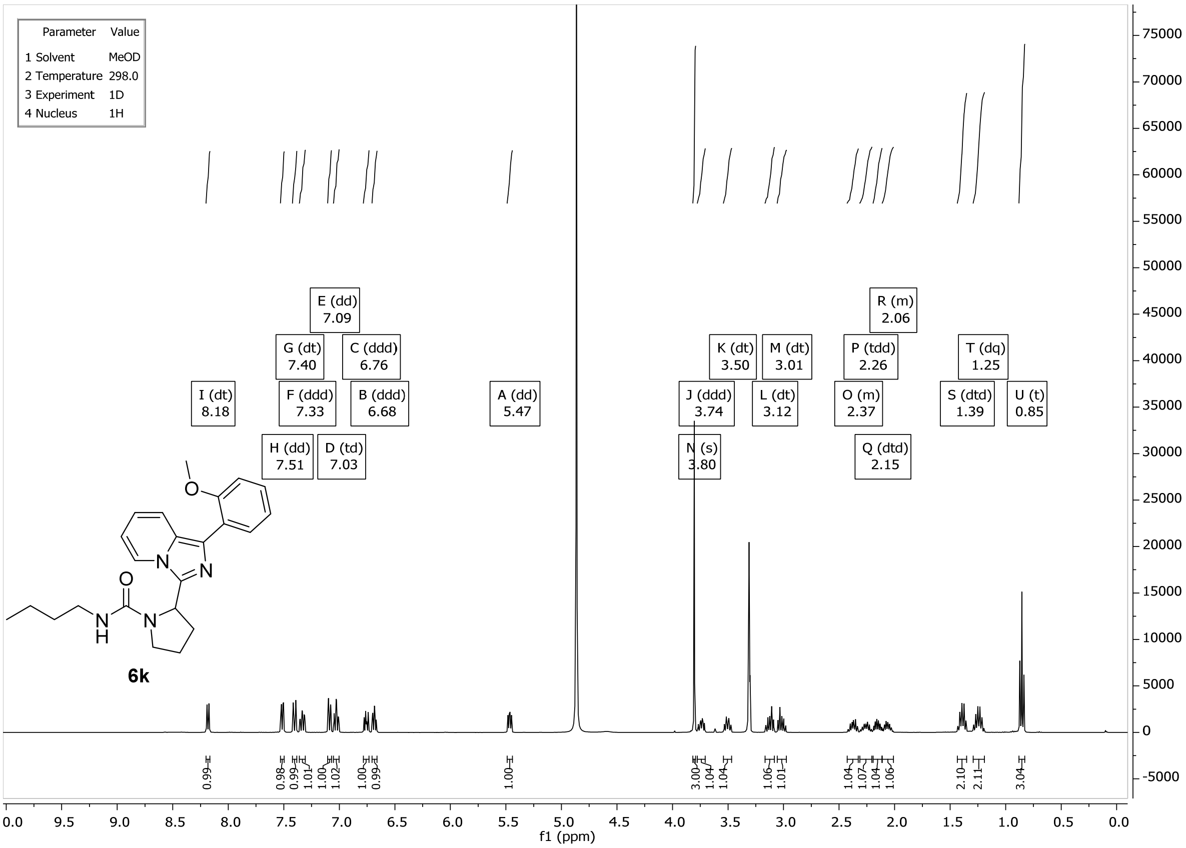

Parameter Value
1 Solvent MeOD
2 Temperature 298.0
3 Experiment 1D
4 Nucleus 1H
E (dd) 7.09
R (m) 2.06
G (dt) 7.40
C (ddd) 6.76
K (dt) 3.50
M (dt) 3.01
P (tdd) 2.26
T (dq) 1.25
I (dt) 8.18
F (ddd) 7.33
B (ddd) 6.68
A (dd) 5.47
J (ddd) 3.74
L (dt) 3.12
O (m) 2.37
S (dtd) 1.39
U (t) 0.85
H (dd) 7.51
D (td) 7.03
N (s) 3.80
Q (dtd) 2.15
6k
f1 (ppm)

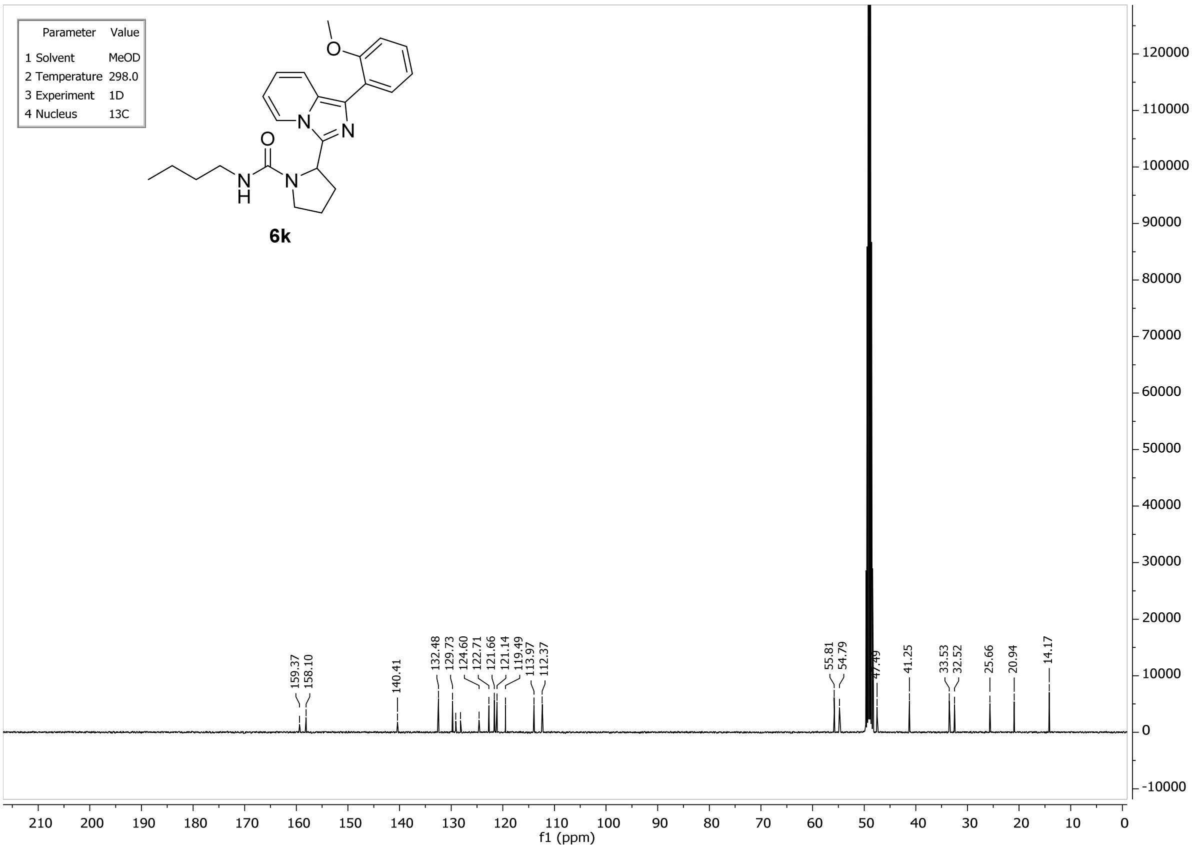

Parameter Value
1 Solvent MeOD
2 Temperature 298.0
3 Experiment 1D
4 Nucleus 13C
6k
159.37
158.10
140.41
132.48
129.73
124.60
122.71
121.66
121.14
119.49
113.97
112.37
55.81
54.79
47.49
41.25
33.53
32.52
25.66
20.94
14.17
f1 (ppm)

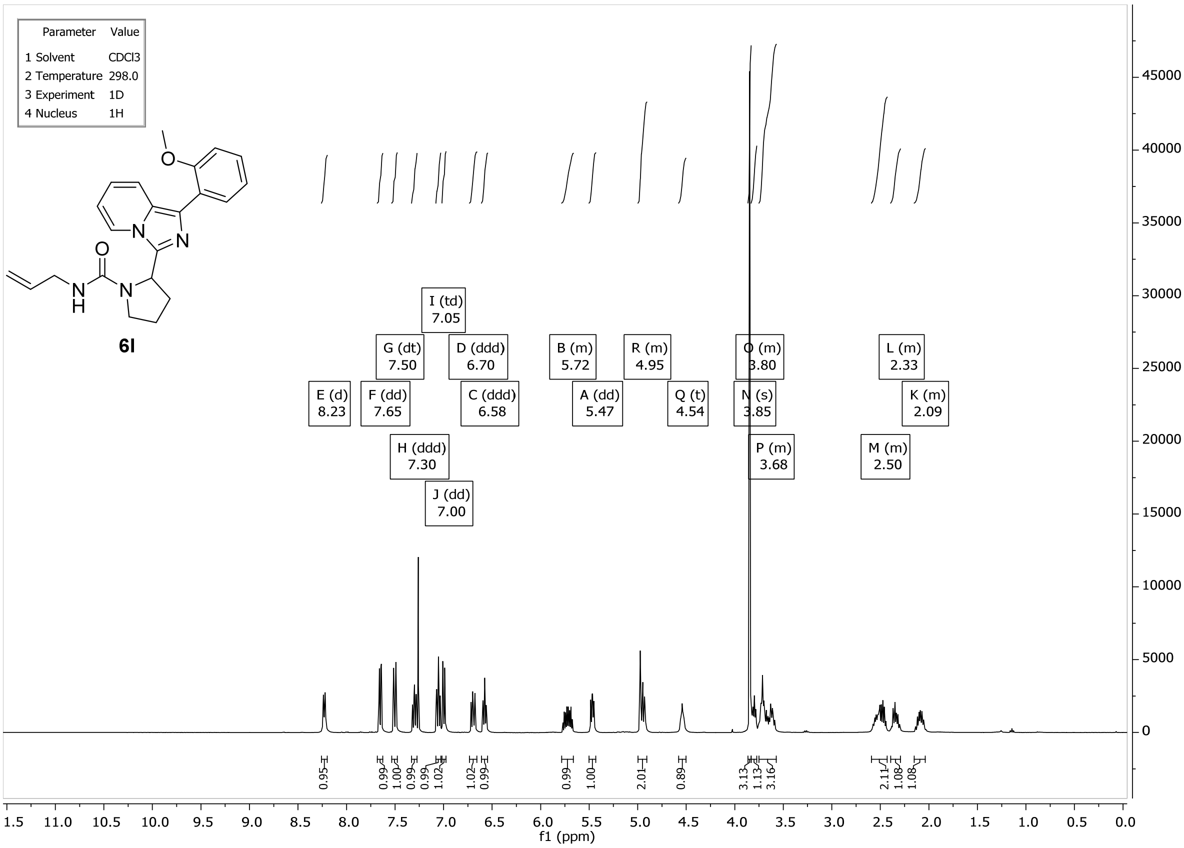
Parameter Value
1 Solvent CDCl3
2 Temperature 298.0
3 Experiment 1D
4 Nucleus 1H
6l
E (d) 8.23
F (dd) 7.65
G (dt) 7.50
H (ddd) 7.30
I (td) 7.05
J (dd) 7.00
D (ddd) 6.70
C (ddd) 6.58
B (m) 5.72
A (dd) 5.47
R (m) 4.95
Q (t) 4.54
N (s) 3.85
O (m) 3.80
P (m) 3.68
M (m) 2.50
L (m) 2.33
K (m) 2.09
0.95
0.99
1.00
0.99
0.99
1.02
1.02
0.99
0.99
1.00
2.01
0.89
3.13
1.13
3.16
2.11
1.08
1.08
f1 (ppm)

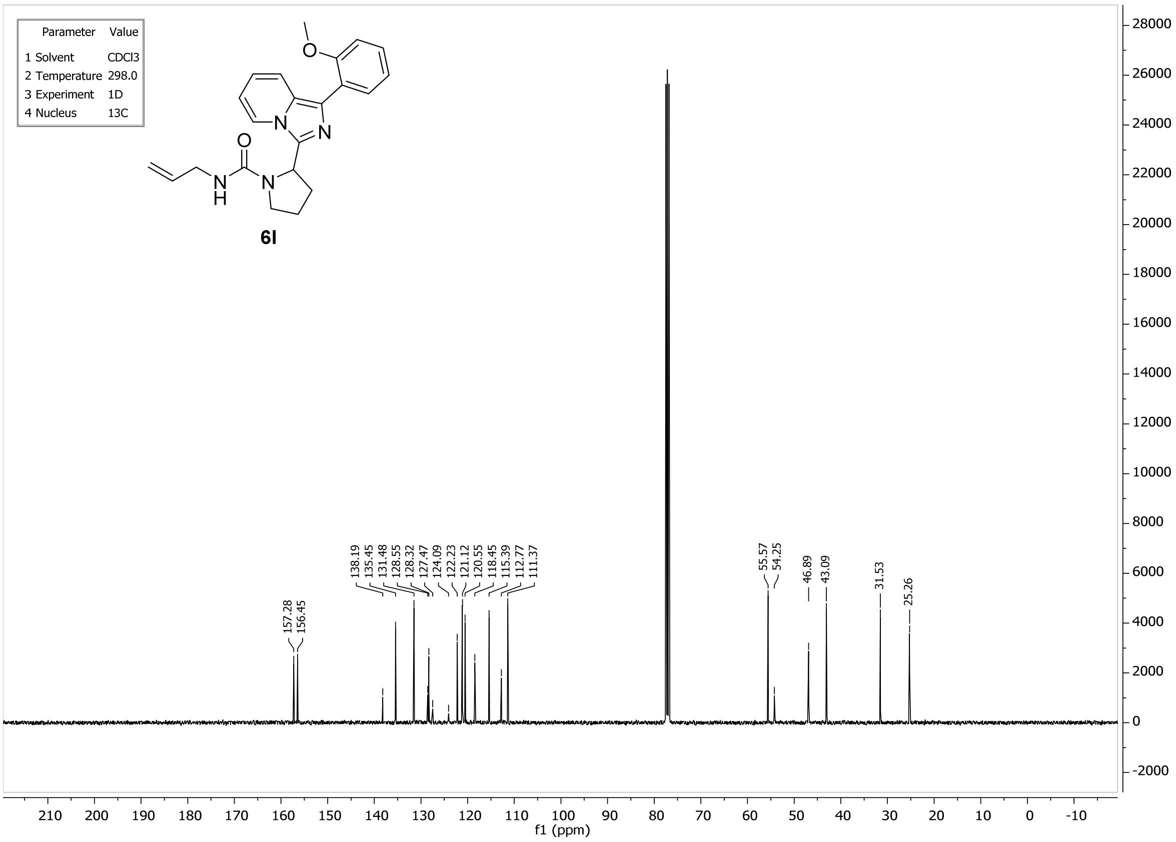
Parameter Value
1 Solvent CDCl3
2 Temperature 298.0
3 Experiment 1D
4 Nucleus 13C
6l
157.28
156.45
138.19
135.45
131.48
128.55
128.32
127.47
124.09
122.23
121.12
120.55
118.45
115.39
112.77
111.37
55.57
54.25
46.89
43.09
31.53
25.26
f1 (ppm)

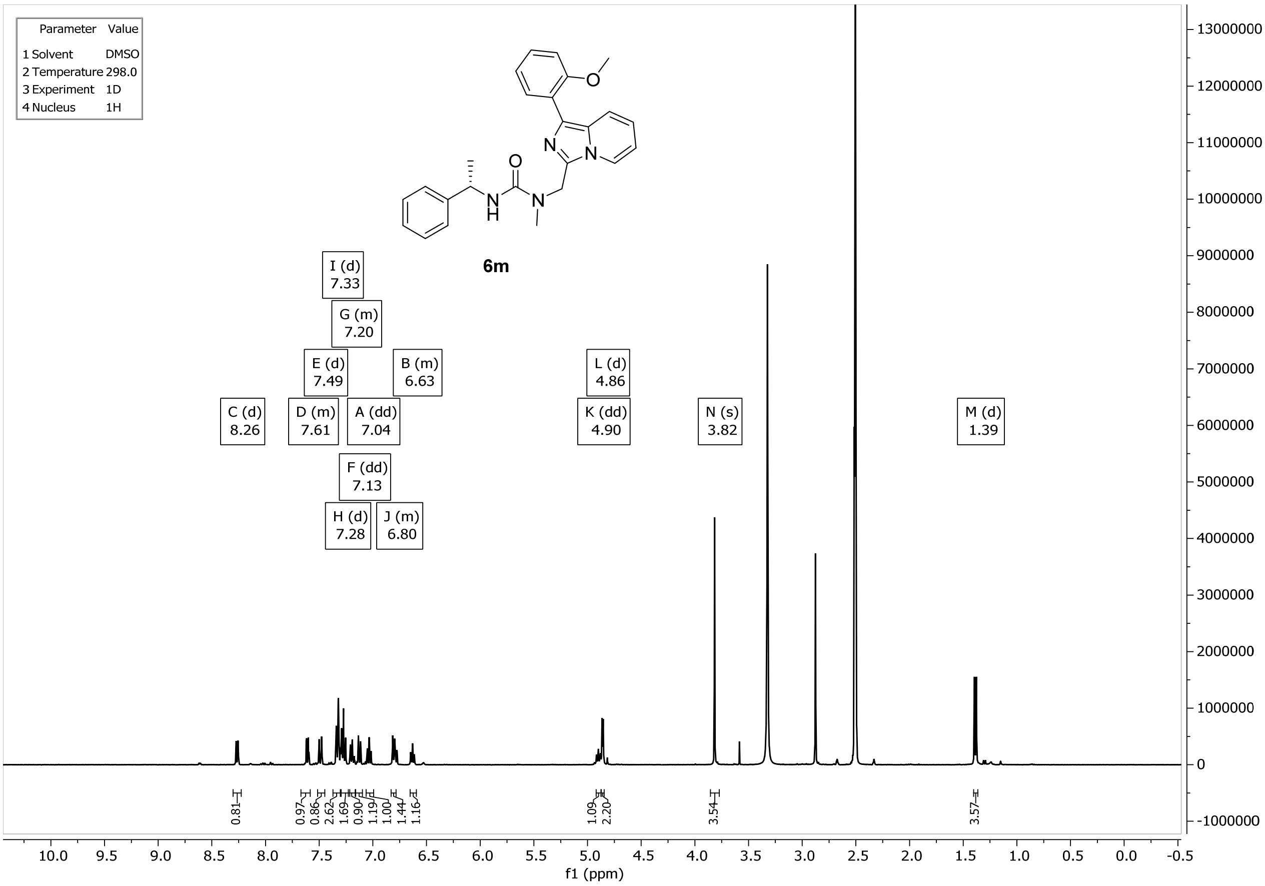
Parameter Value
1 Solvent DMSO
2 Temperature 298.0
3 Experiment 1D
4 Nucleus 1H
6m
I (d) 7.33
G (m) 7.20
E (d) 7.49
B (m) 6.63
L (d) 4.86
C (d) 8.26
D (m) 7.61
A (dd) 7.04
K (dd) 4.90
N (s) 3.82
M (d) 1.39
F (dd) 7.13
H (d) 7.28
J (m) 6.80
0.81
0.97
0.86
2.62
1.69
0.90
1.19
1.00
1.44
1.16
1.09
2.20
3.54
3.57
f1 (ppm)

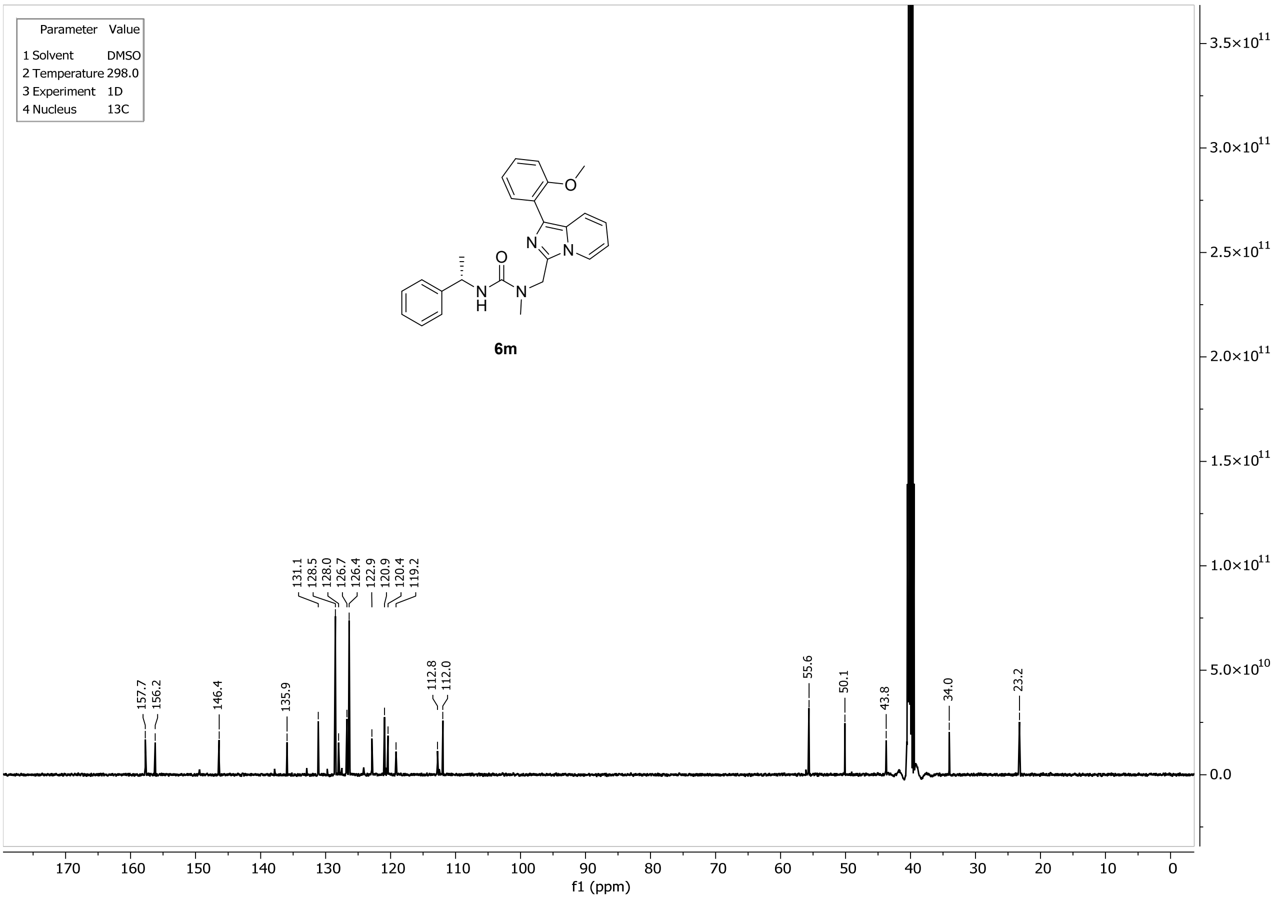
Parameter Value
1 Solvent DMSO
2 Temperature 298.0
3 Experiment 1D
4 Nucleus 13C
6m
157.7
156.2
146.4
135.9
131.1
128.5
128.0
126.7
126.4
122.9
120.9
120.4
119.2
112.8
112.0
55.6
50.1
43.8
34.0
23.2
f1 (ppm)

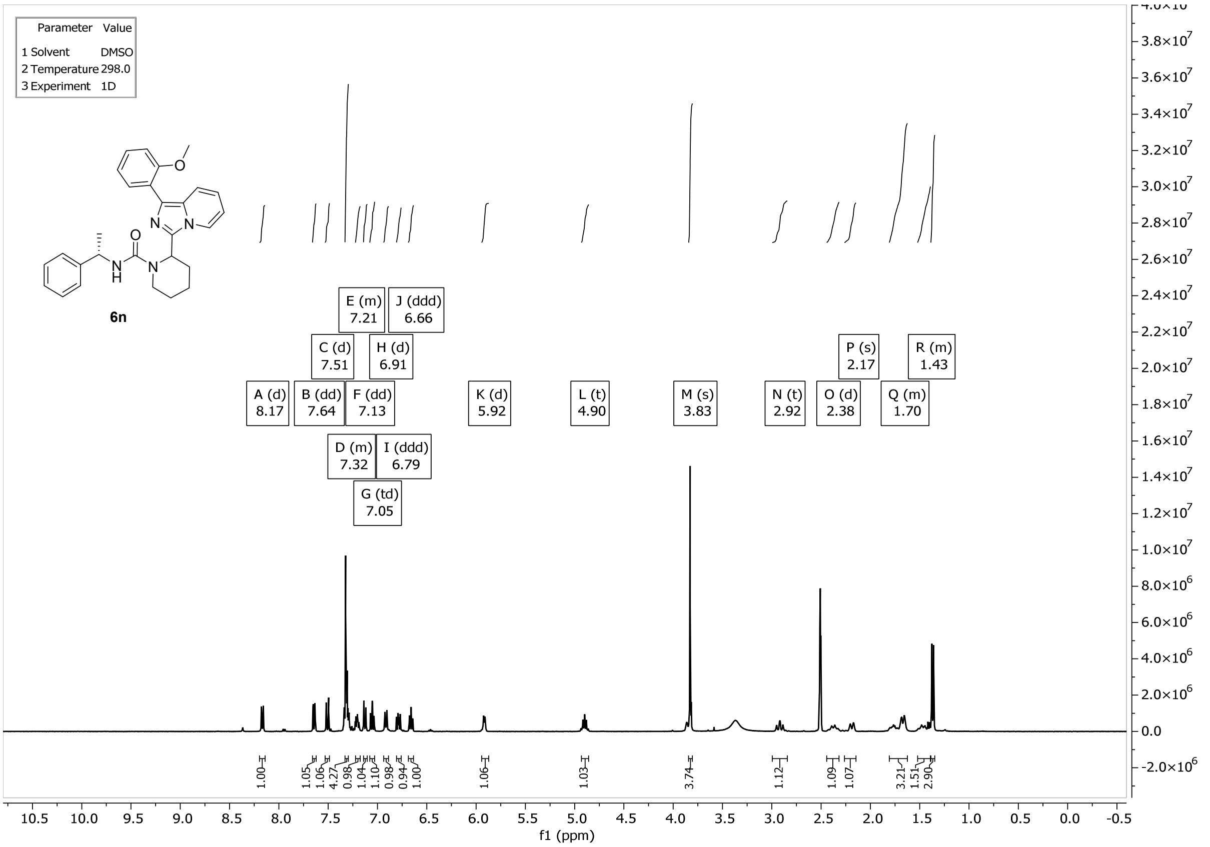
Parameter Value
1 Solvent DMSO
2 Temperature 298.0
3 Experiment 1D
6n
A (d) 8.17
B (dd) 7.64
C (d) 7.51
D (m) 7.32
E (m) 7.21
F (dd) 7.13
G (td) 7.05
H (d) 6.91
I (ddd) 6.79
J (ddd) 6.66
K (d) 5.92
L (t) 4.90
M (s) 3.83
N (t) 2.92
O (d) 2.38
P (s) 2.17
Q (m) 1.70
R (m) 1.43
1.00 1.05 1.06 4.27 0.98 1.04 1.10 0.98 0.94 1.00 1.06 1.03 3.74 1.12 1.09 1.07 3.21 1.51 2.90
f1 (ppm)

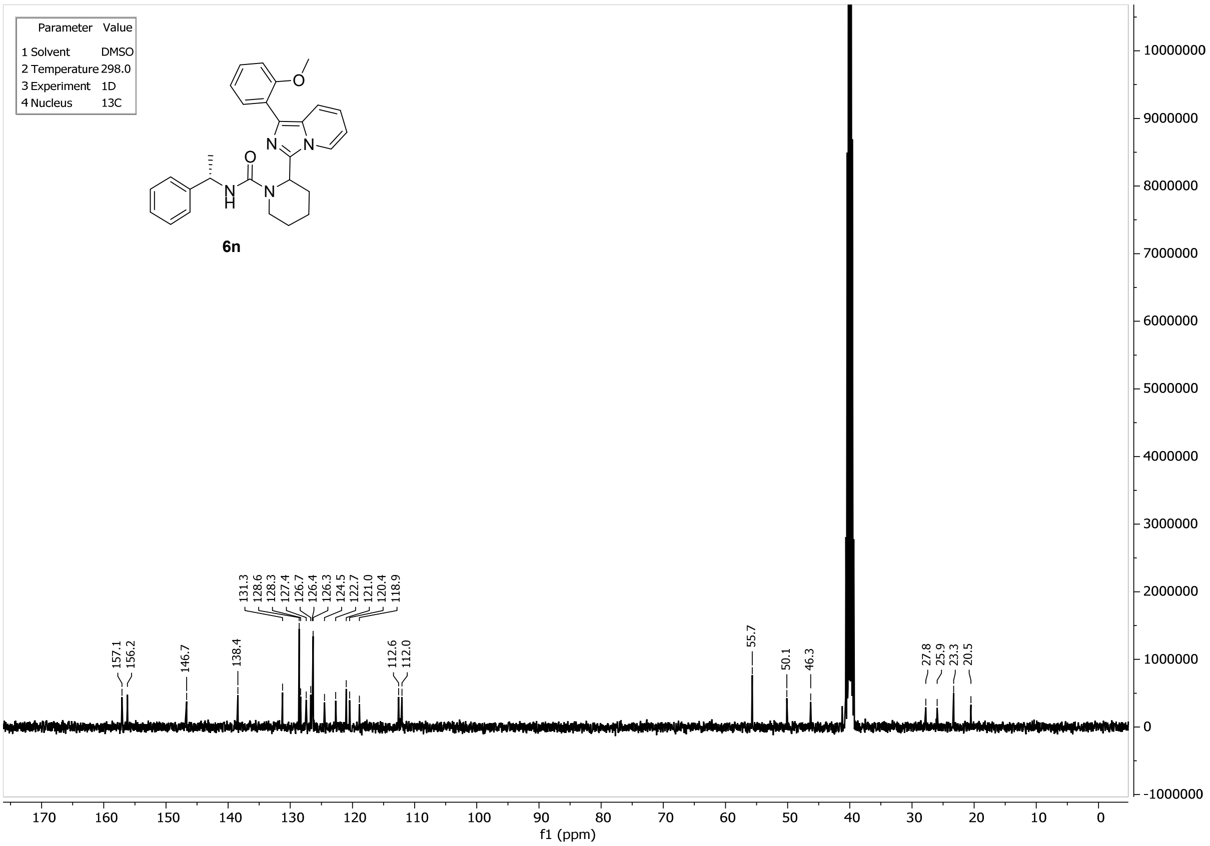
Parameter Value
1 Solvent DMSO
2 Temperature 298.0
3 Experiment 1D
4 Nucleus 13C
6n
157.1 156.2 146.7 138.4 131.3 128.6 128.3 127.4 126.7 126.4 126.3 124.5 122.7 121.0 120.4 118.9 112.6 112.0 55.7 50.1 46.3 27.8 25.9 23.3 20.5
f1 (ppm)

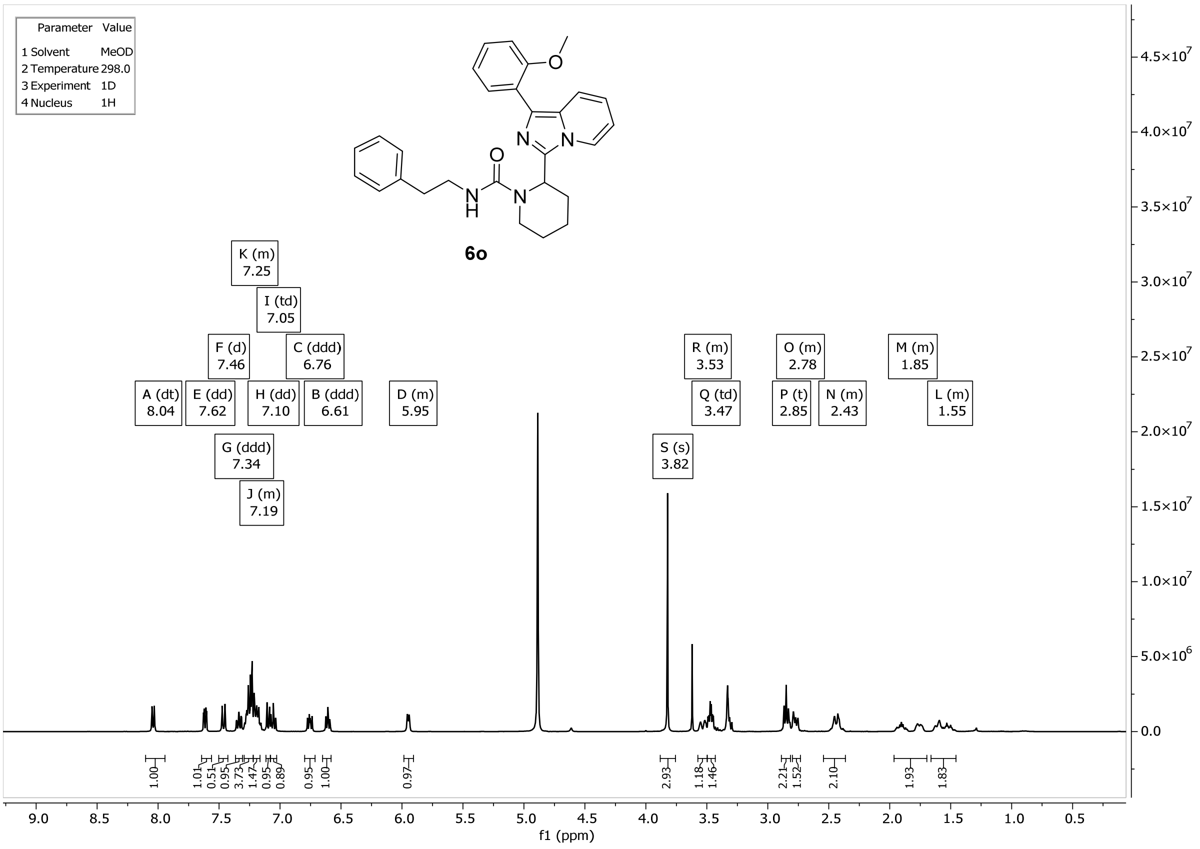
Parameter Value
1 Solvent MeOD
2 Temperature 298.0
3 Experiment 1D
4 Nucleus 1H
6o
A (dt) 8.04
E (dd) 7.62
F (d) 7.46
G (ddd) 7.34
K (m) 7.25
J (m) 7.19
H (dd) 7.10
I (td) 7.05
C (ddd) 6.76
B (ddd) 6.61
D (m) 5.95
S (s) 3.82
R (m) 3.53
Q (td) 3.47
P (t) 2.85
O (m) 2.78
N (m) 2.43
M (m) 1.85
L (m) 1.55
1.00 1.01 0.51 0.95 3.73 1.47 0.95 0.89 0.95 1.00 0.97 2.93 1.18 1.46 2.21 1.52 2.10 1.93 1.83
f1 (ppm)

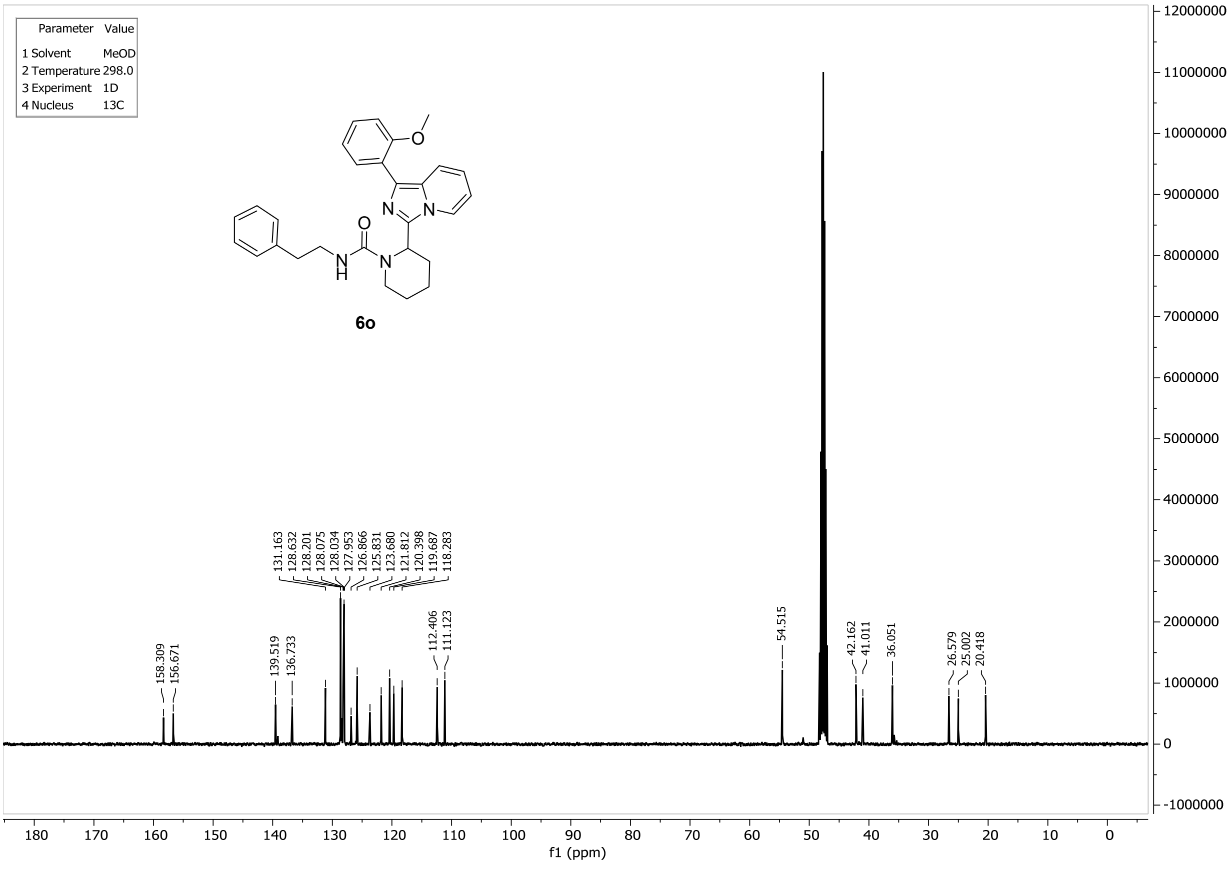
Parameter Value
1 Solvent MeOD
2 Temperature 298.0
3 Experiment 1D
4 Nucleus 13C
6o
158.309 156.671 139.519 136.733
131.163 128.632 128.201 128.075 128.034 127.953 126.866 125.831 123.680 121.812 120.398 119.687 118.283
112.406 111.123
54.515 42.162 41.011 36.051 26.579 25.002 20.418
f1 (ppm)

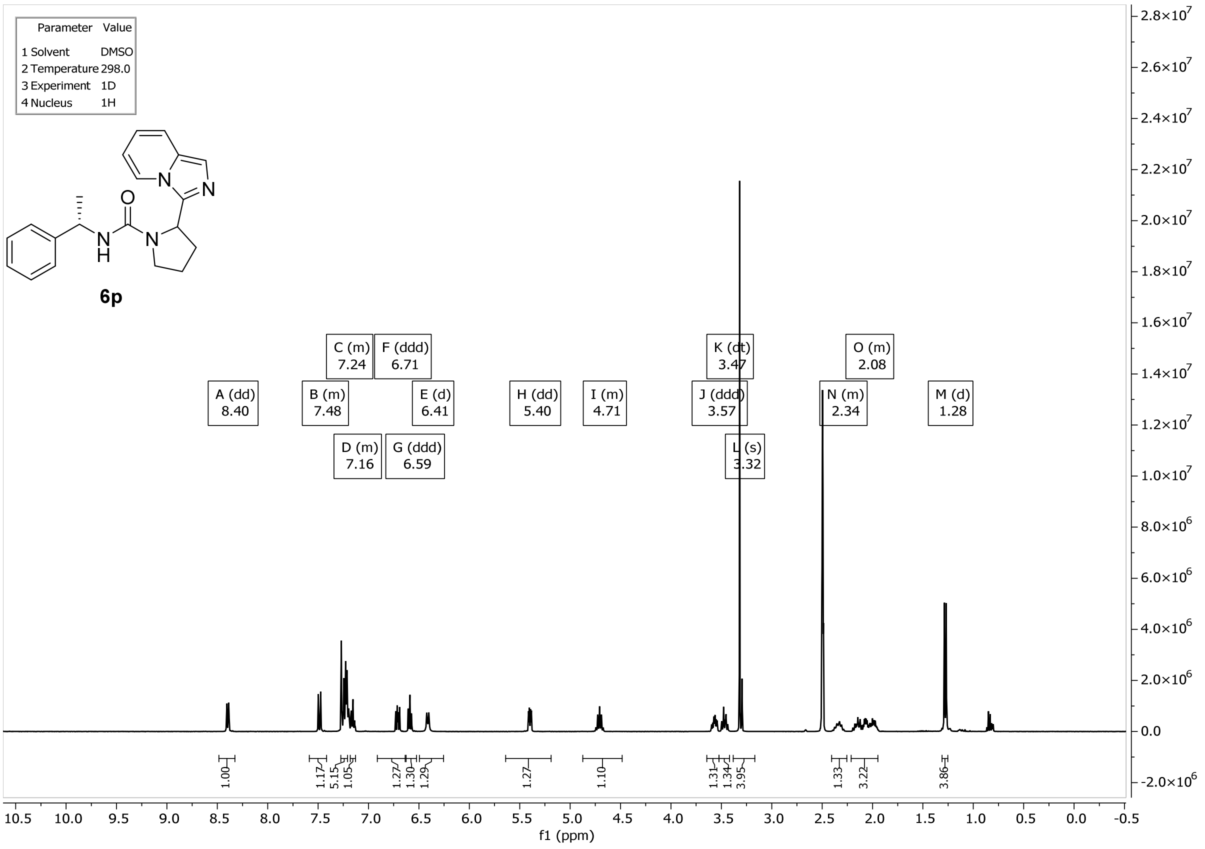
Parameter Value
1 Solvent DMSO
2 Temperature 298.0
3 Experiment 1D
4 Nucleus 1H
6p
A (dd) 8.40
B (m) 7.48
C (m) 7.24
D (m) 7.16
E (d) 6.41
F (ddd) 6.71
G (ddd) 6.59
H (dd) 5.40
I (m) 4.71
J (ddd) 3.57
K (dt) 3.47
L (s) 3.32
M (d) 1.28
N (m) 2.34
O (m) 2.08
1.00
1.17
5.15
1.05
1.27
1.30
1.29
1.27
1.10
1.31
1.34
3.95
1.33
3.22
3.86
f1 (ppm)

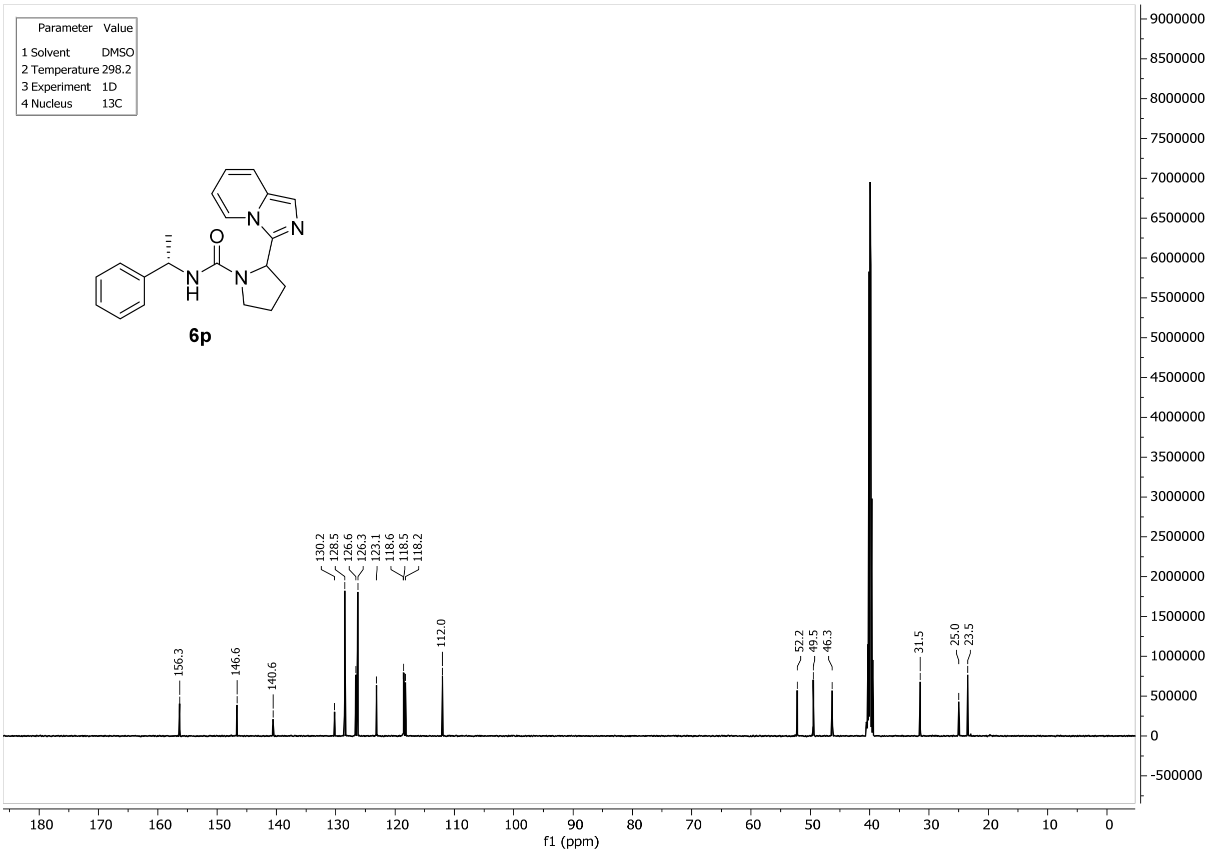
Parameter Value
1 Solvent DMSO
2 Temperature 298.2
3 Experiment 1D
4 Nucleus 13C
6p
156.3
146.6
140.6
130.2
128.5
126.6
126.3
123.1
118.6
118.5
118.2
112.0
52.2
49.5
46.3
31.5
25.0
23.5
f1 (ppm)

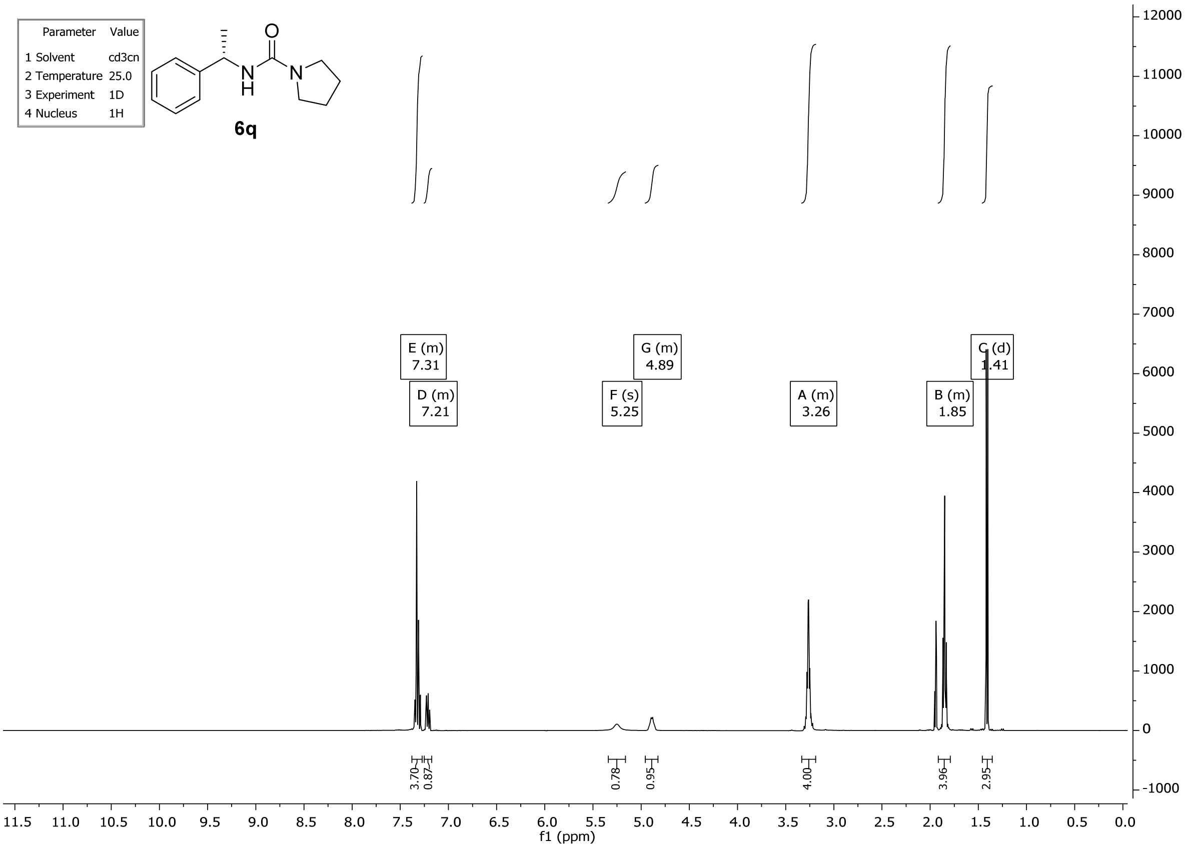
Parameter Value
1 Solvent cd3cn
2 Temperature 25.0
3 Experiment 1D
4 Nucleus 1H
6q
E (m) 7.31
D (m) 7.21
F (s) 5.25
G (m) 4.89
A (m) 3.26
B (m) 1.85
C (d) 1.41
3.70
0.87
0.78
0.95
4.00
3.96
2.95
f1 (ppm)

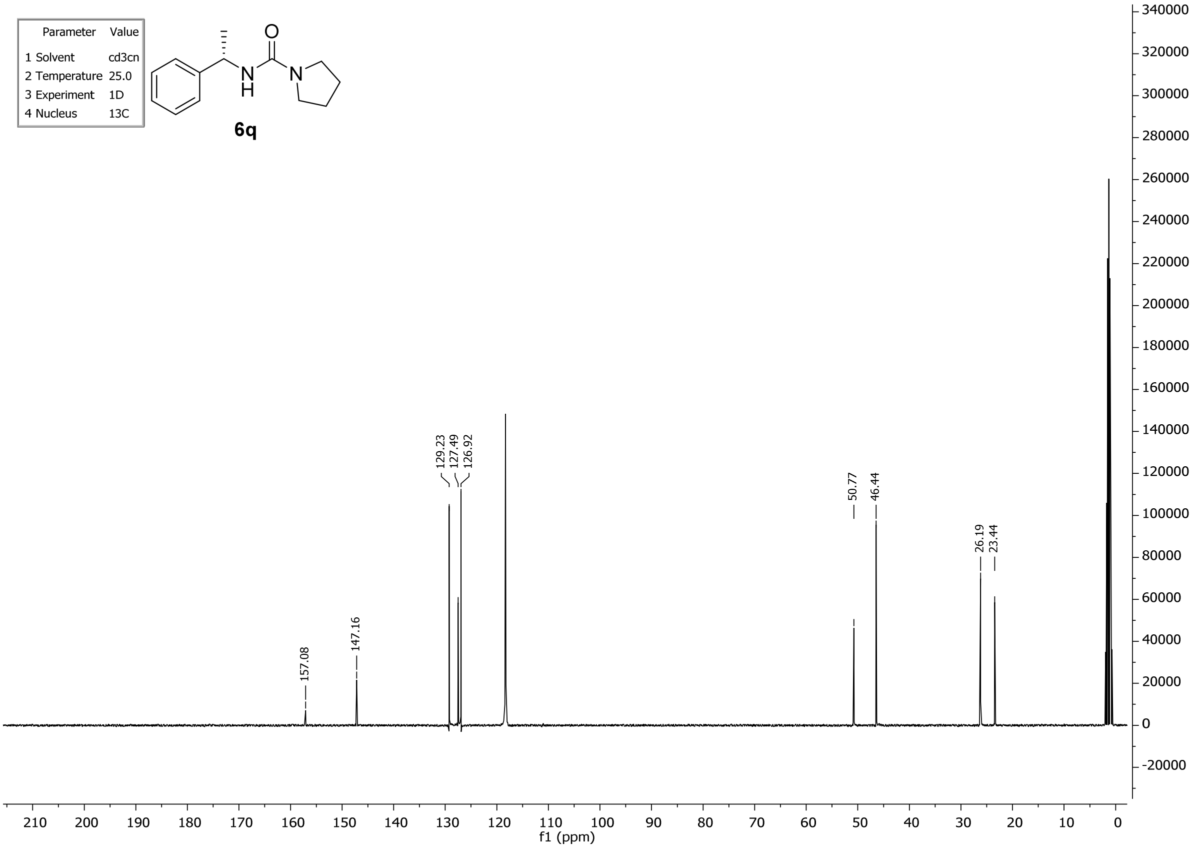
Parameter Value
1 Solvent cd3cn
2 Temperature 25.0
3 Experiment 1D
4 Nucleus 13C
6q
157.08
147.16
129.23
127.49
126.92
50.77
46.44
26.19
23.44
f1 (ppm)

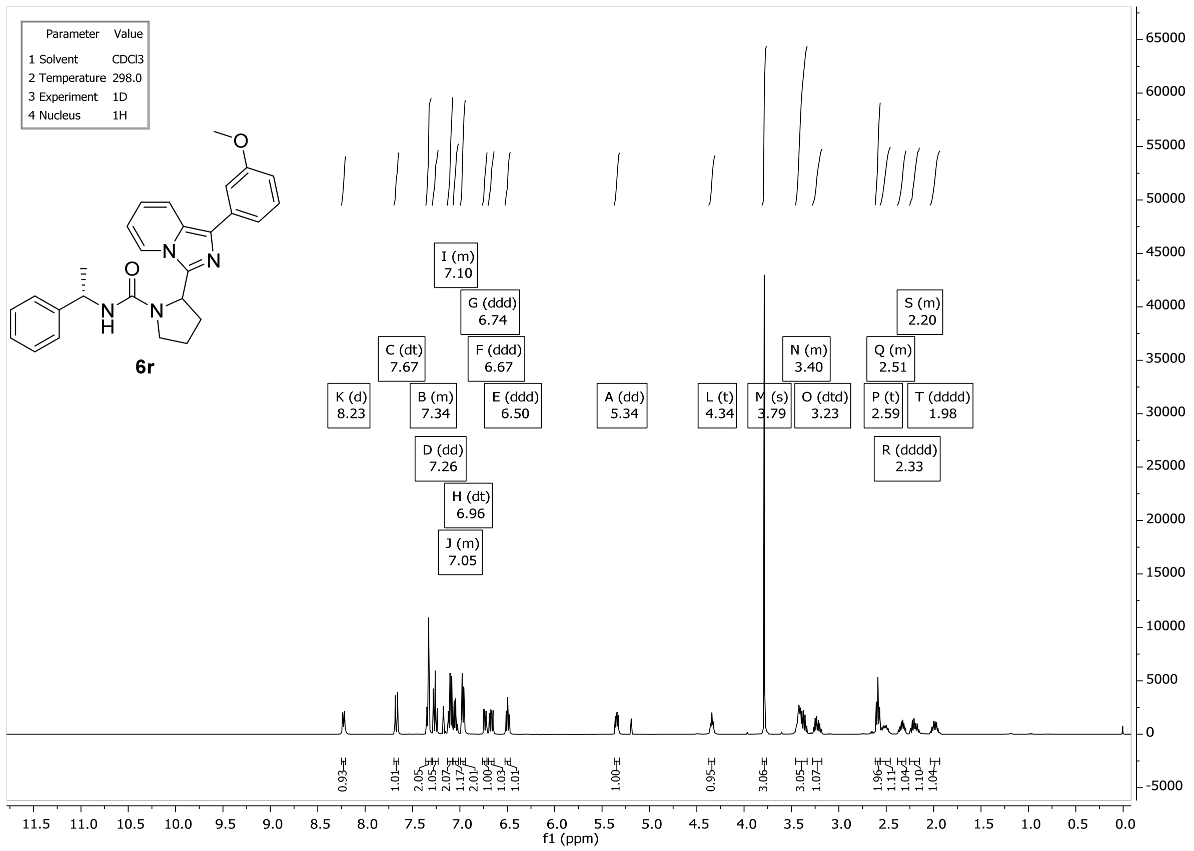
Parameter Value
1 Solvent CDCl3
2 Temperature 298.0
3 Experiment 1D
4 Nucleus 1H
6r
K (d) 8.23
C (dt) 7.67
B (m) 7.34
D (dd) 7.26
I (m) 7.10
J (m) 7.05
H (dt) 6.96
G (ddd) 6.74
F (ddd) 6.67
E (ddd) 6.50
A (dd) 5.34
L (t) 4.34
M (s) 3.79
N (m) 3.40
O (dtd) 3.23
P (t) 2.59
Q (m) 2.51
R (dddd) 2.33
S (m) 2.20
T (dddd) 1.98
f1 (ppm)

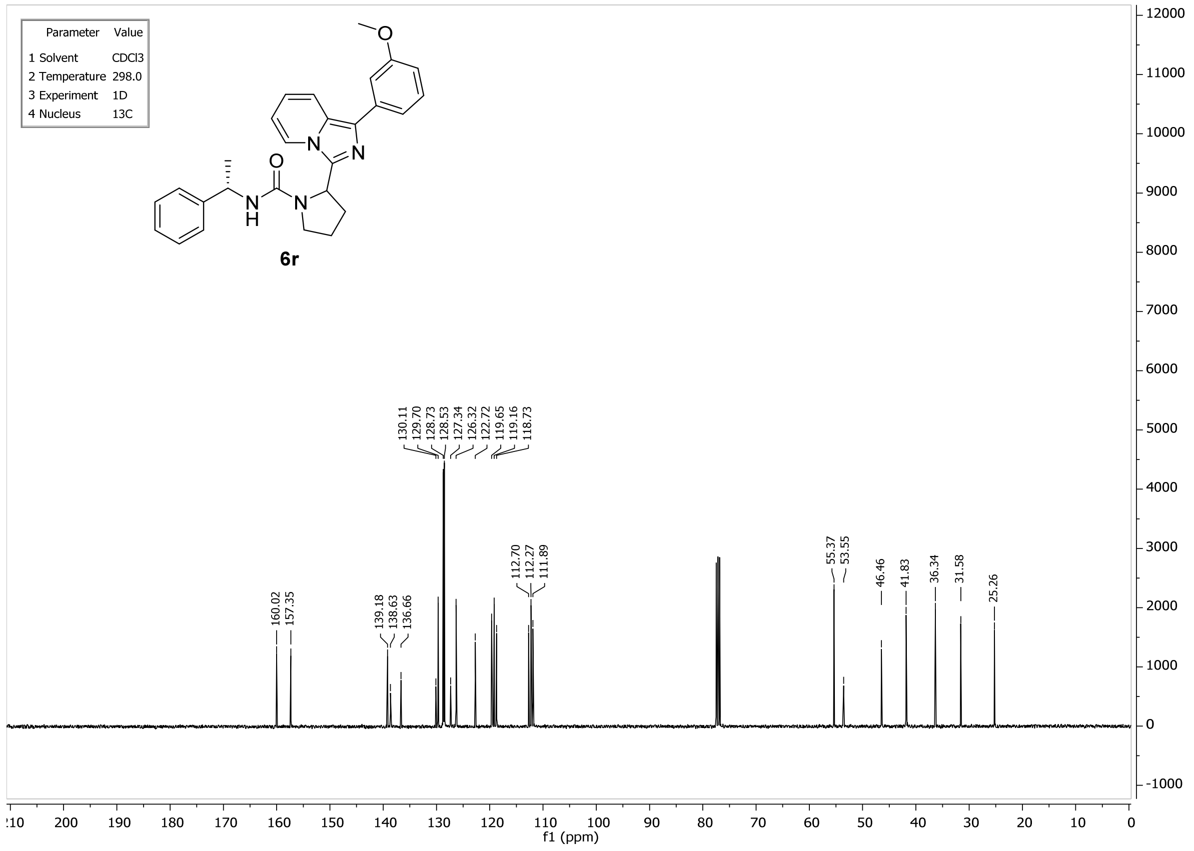
Parameter Value
1 Solvent CDCl3
2 Temperature 298.0
3 Experiment 1D
4 Nucleus 13C
6r
160.02
157.35
139.18
138.63
136.66
130.11
129.70
128.73
128.53
127.34
126.32
122.72
119.65
119.16
118.73
112.70
112.27
111.89
55.37
53.55
46.46
41.83
36.34
31.58
25.26
f1 (ppm)

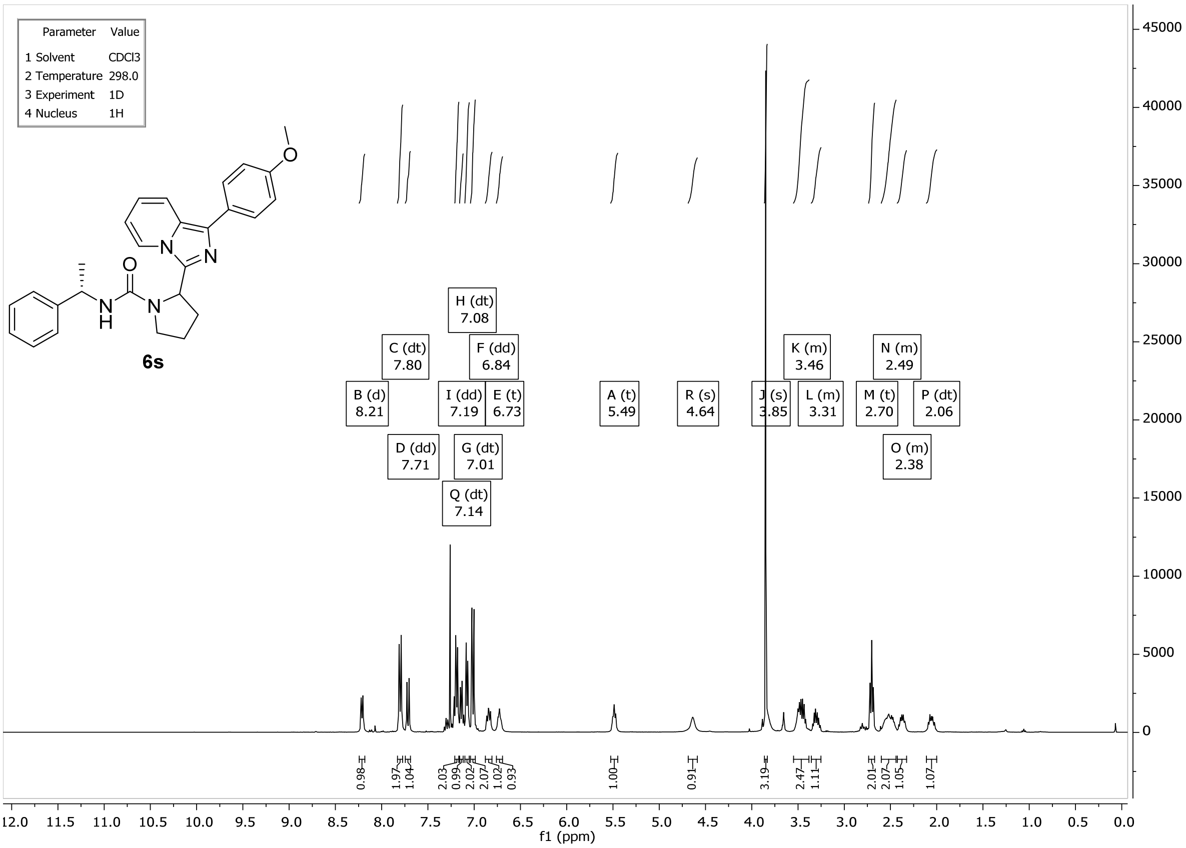

Parameter Value
1 Solvent CDCl3
2 Temperature 298.0
3 Experiment 1D
4 Nucleus 1H
6s
H (dt) 7.08
C (dt) 7.80
F (dd) 6.84
K (m) 3.46
N (m) 2.49
B (d) 8.21
I (dd) 7.19
E (t) 6.73
A (t) 5.49
R (s) 4.64
J (s) 3.85
L (m) 3.31
M (t) 2.70
P (dt) 2.06
D (dd) 7.71
G (dt) 7.01
O (m) 2.38
Q (dt) 7.14
0.98 1.97 1.04 2.03 0.99 2.02 2.07 1.02 0.93 1.00 0.91 3.19 2.47 1.11 2.01 2.07 1.05 1.07
f1 (ppm)

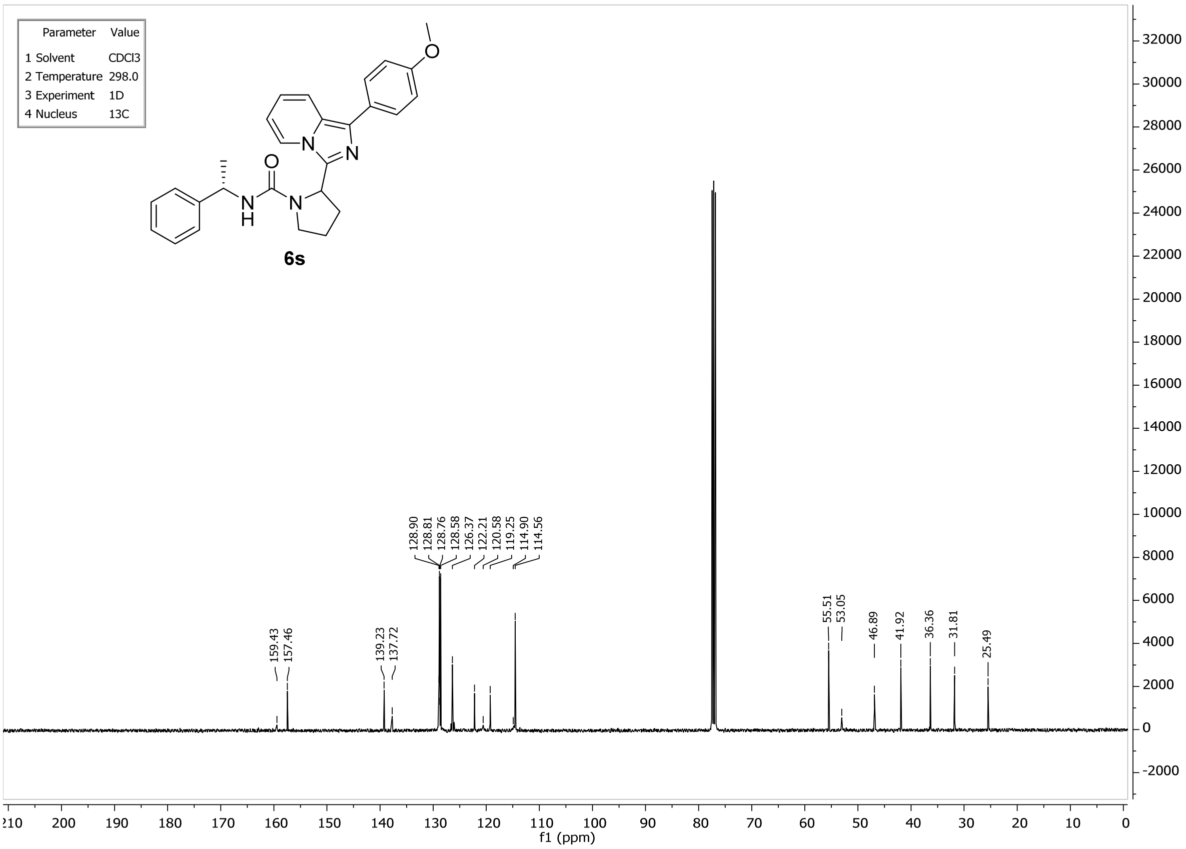

Parameter Value
1 Solvent CDCl3
2 Temperature 298.0
3 Experiment 1D
4 Nucleus 13C
6s
159.43 157.46 139.23 137.72
128.90 128.81 128.76 128.58 126.37 122.21 120.58 119.25 114.90 114.56
55.51 53.05 46.89 41.92 36.36 31.81 25.49
f1 (ppm)

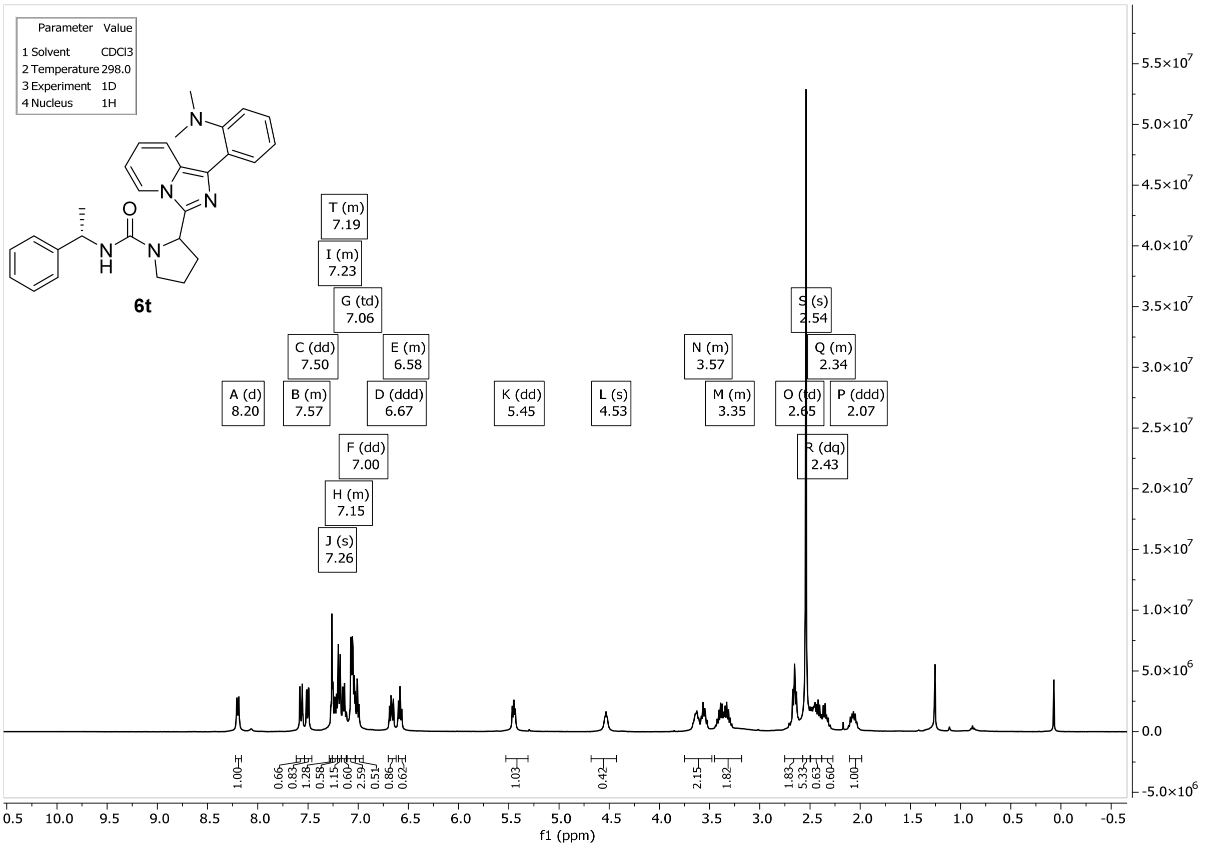
Parameter Value
1 Solvent CDCl3
2 Temperature 298.0
3 Experiment 1D
4 Nucleus 1H
6t
A (d) 8.20
B (m) 7.57
C (dd) 7.50
D (ddd) 6.67
E (m) 6.58
F (dd) 7.00
G (td) 7.06
H (m) 7.15
I (m) 7.23
J (s) 7.26
K (dd) 5.45
L (s) 4.53
M (m) 3.35
N (m) 3.57
O (td) 2.65
P (ddd) 2.07
Q (m) 2.34
R (dq) 2.43
S (s) 2.54
T (m) 7.19
1.00
0.66
0.83
1.28
0.58
1.15
0.60
2.59
0.51
0.86
0.62
1.03
0.42
2.15
1.82
1.83
5.33
0.63
0.60
1.00
f1 (ppm)

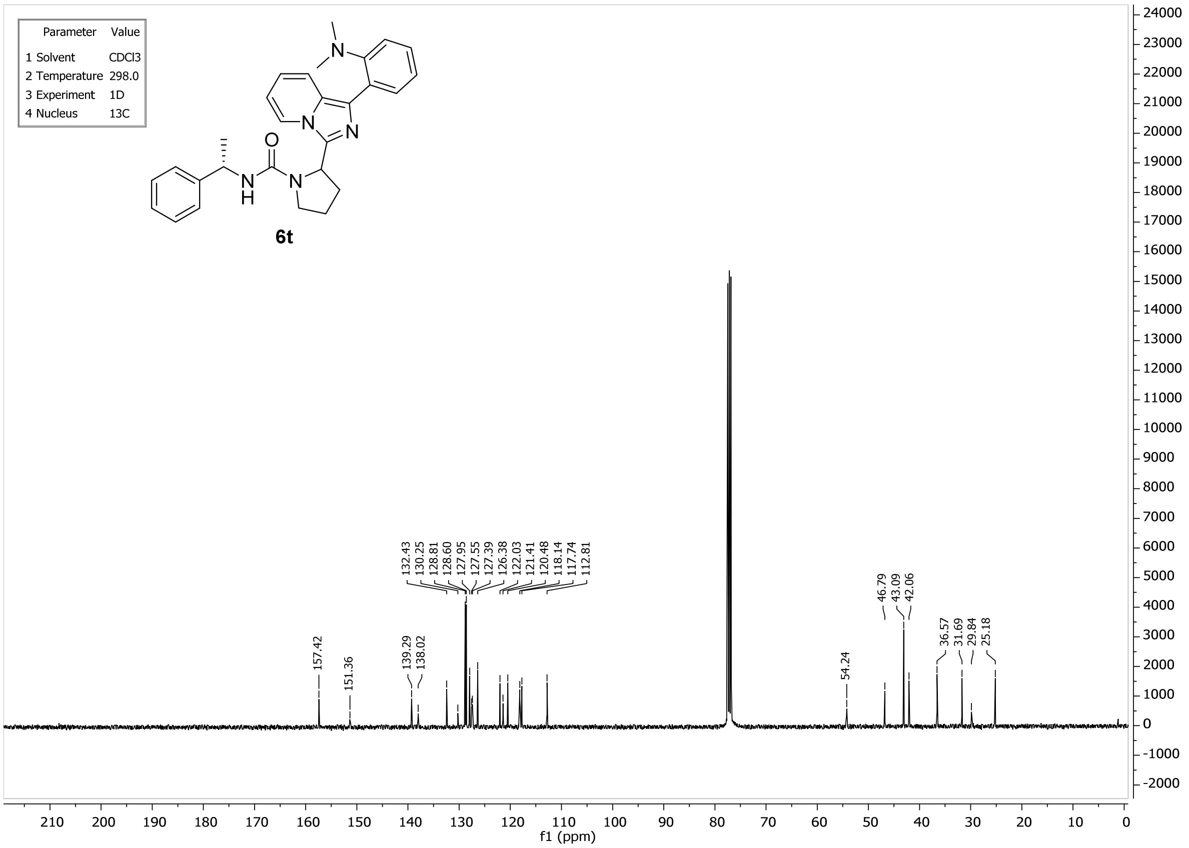
Parameter Value
1 Solvent CDCl3
2 Temperature 298.0
3 Experiment 1D
4 Nucleus 13C
6t
157.42
151.36
139.29
138.02
132.43
130.25
128.81
128.60
127.95
127.55
127.39
126.38
122.03
121.41
120.48
118.14
117.74
112.81
54.24
46.79
43.09
42.06
36.57
31.69
29.84
25.18
f1 (ppm)

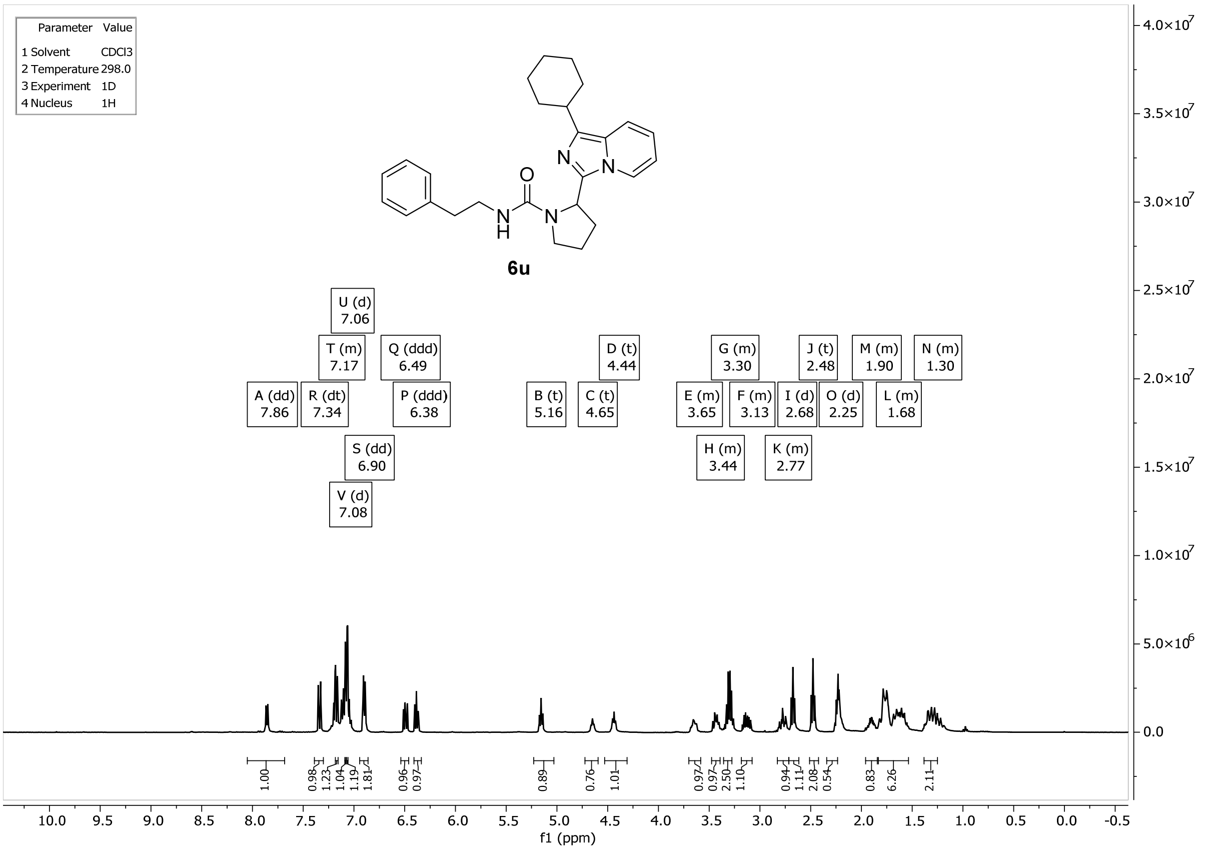
Parameter Value
1 Solvent CDCl3
2 Temperature 298.0
3 Experiment 1D
4 Nucleus 1H
6u
A (dd) 7.86
R (dt) 7.34
T (m) 7.17
U (d) 7.06
V (d) 7.08
S (dd) 6.90
Q (ddd) 6.49
P (ddd) 6.38
B (t) 5.16
C (t) 4.65
D (t) 4.44
E (m) 3.65
H (m) 3.44
G (m) 3.30
F (m) 3.13
I (d) 2.68
K (m) 2.77
J (t) 2.48
O (d) 2.25
M (m) 1.90
L (m) 1.68
N (m) 1.30
f1 (ppm)

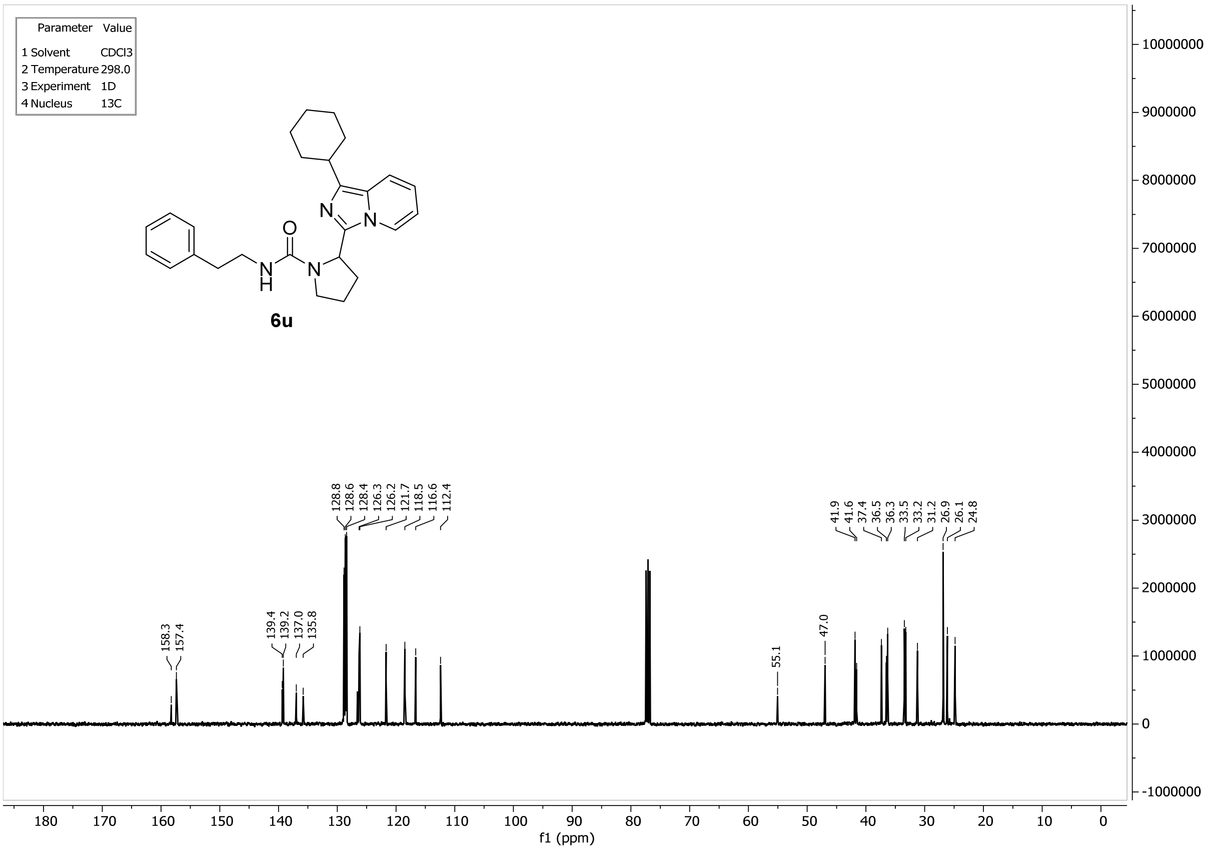
Parameter Value
1 Solvent CDCl3
2 Temperature 298.0
3 Experiment 1D
4 Nucleus 13C
6u
158.3
157.4
139.4
139.2
137.0
135.8
128.8
128.6
128.4
126.3
126.2
121.7
118.5
116.6
112.4
55.1
47.0
41.9
41.6
37.4
36.5
36.3
33.5
33.2
31.2
26.9
26.1
24.8
f1 (ppm)

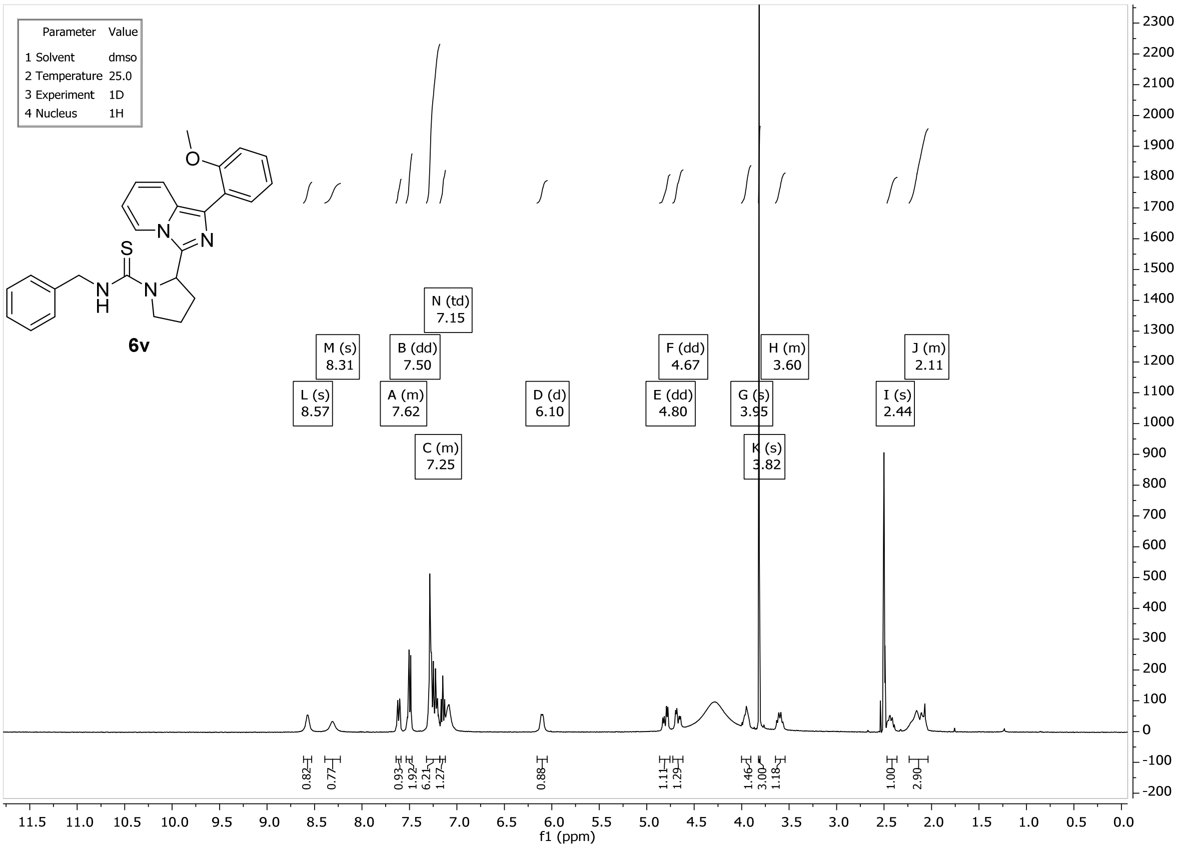

Parameter Value
1 Solvent dmso
2 Temperature 25.0
3 Experiment 1D
4 Nucleus 1H
6v
N (td) 7.15
M (s) 8.31
B (dd) 7.50
F (dd) 4.67
H (m) 3.60
J (m) 2.11
L (s) 8.57
A (m) 7.62
D (d) 6.10
E (dd) 4.80
G (s) 3.95
I (s) 2.44
C (m) 7.25
K (s) 3.82
f1 (ppm)

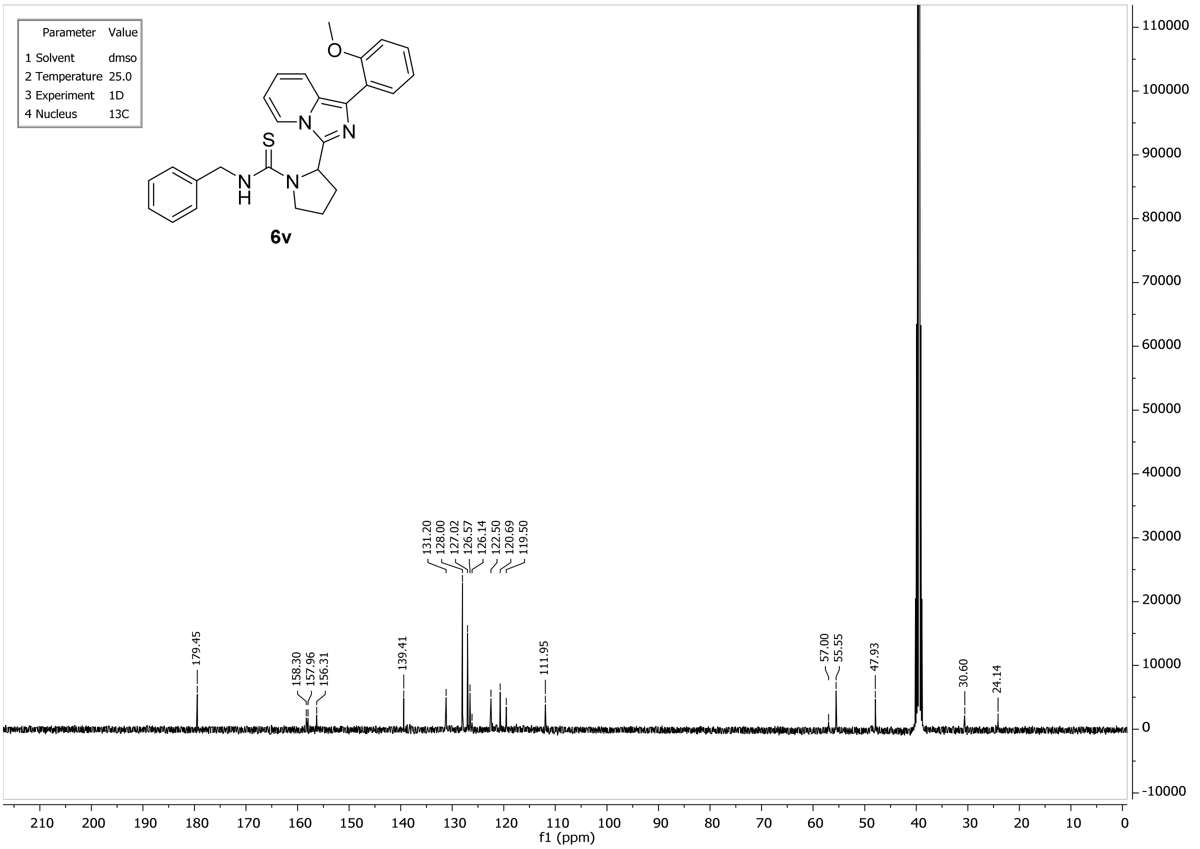

Parameter Value
1 Solvent dmso
2 Temperature 25.0
3 Experiment 1D
4 Nucleus 13C
6v
179.45
158.30
157.96
156.31
139.41
131.20
128.00
127.02
126.57
126.14
122.50
120.69
119.50
111.95
57.00
55.55
47.93
30.60
24.14
f1 (ppm)

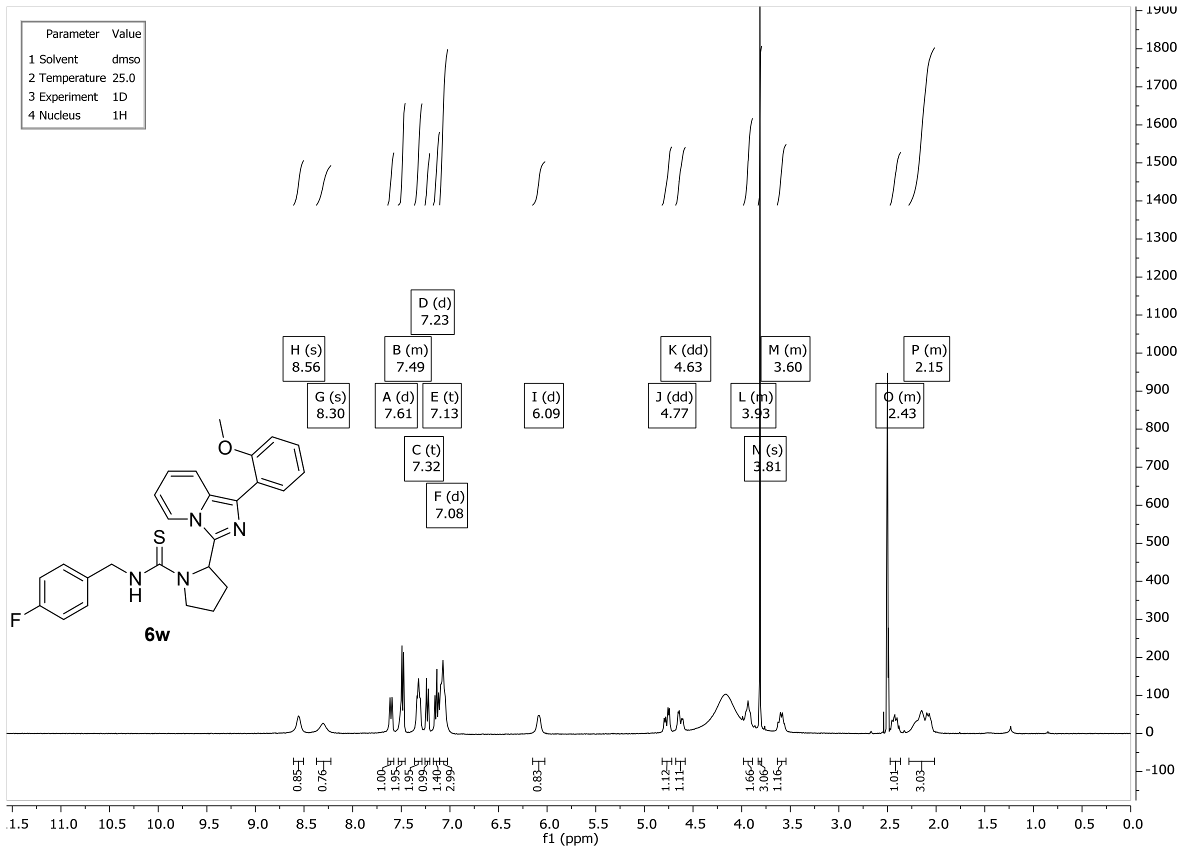
Parameter Value
1 Solvent dmso
2 Temperature 25.0
3 Experiment 1D
4 Nucleus 1H
6w
H (s) 8.56
G (s) 8.30
B (m) 7.49
A (d) 7.61
D (d) 7.23
E (t) 7.13
C (t) 7.32
F (d) 7.08
I (d) 6.09
K (dd) 4.63
J (dd) 4.77
L (m) 3.93
N (s) 3.81
M (m) 3.60
O (m) 2.43
P (m) 2.15
0.85
0.76
1.00
1.95
1.95
0.99
1.40
2.99
0.83
1.12
1.11
1.66
3.06
1.16
1.01
3.03
f1 (ppm)

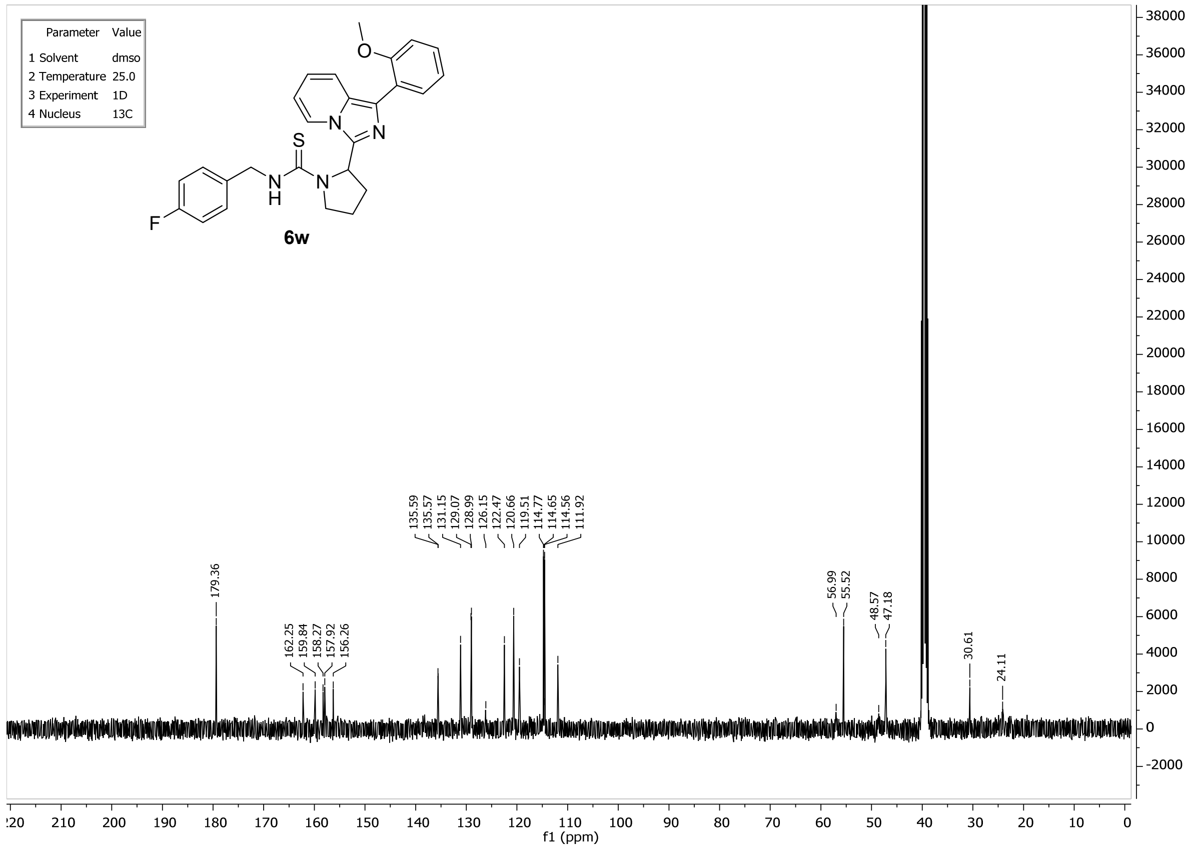
Parameter Value
1 Solvent dmso
2 Temperature 25.0
3 Experiment 1D
4 Nucleus 13C
6w
179.36
162.25
159.84
158.27
157.92
156.26
135.59
135.57
131.15
129.07
128.99
126.15
122.47
120.66
119.51
114.77
114.65
114.56
111.92
56.99
55.52
48.57
47.18
30.61
24.11
f1 (ppm)

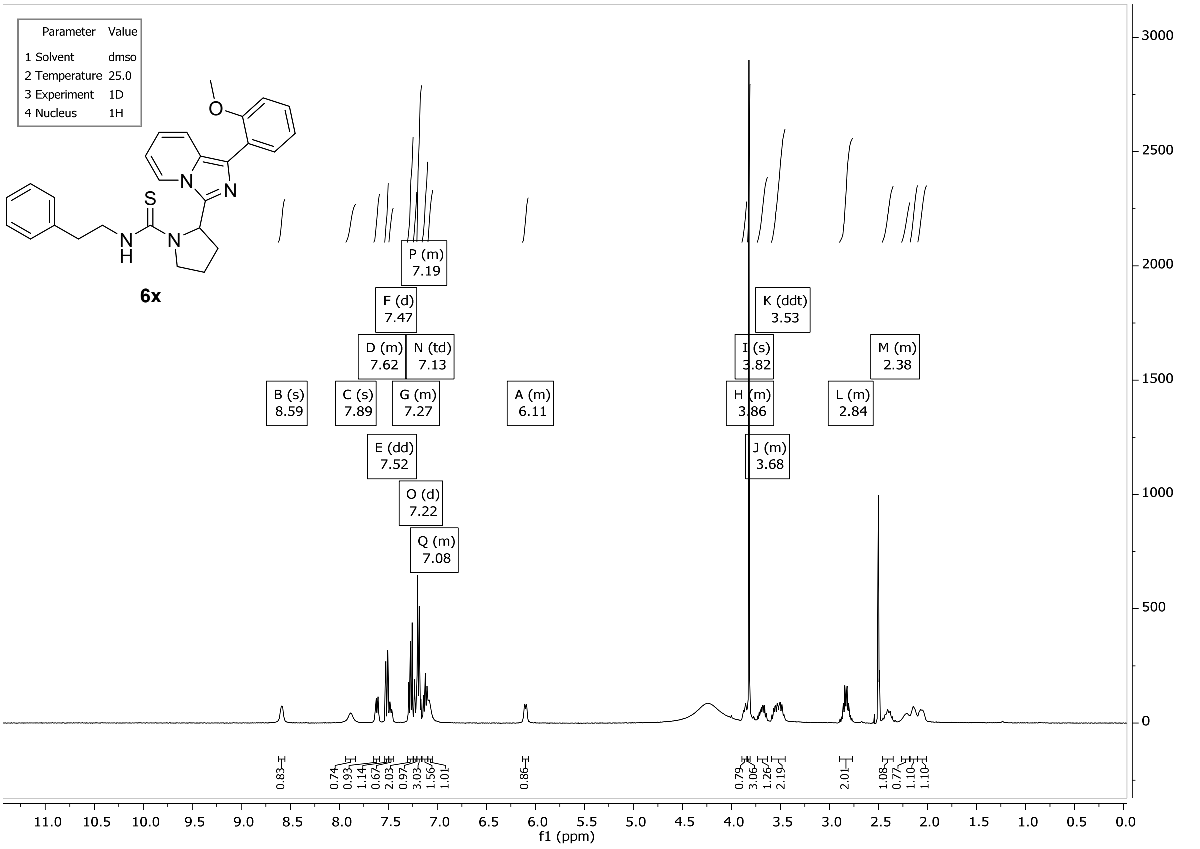
Parameter Value
1 Solvent dmso
2 Temperature 25.0
3 Experiment 1D
4 Nucleus 1H
6x
B (s) 8.59
C (s) 7.89
D (m) 7.62
E (dd) 7.52
F (d) 7.47
G (m) 7.27
O (d) 7.22
P (m) 7.19
N (td) 7.13
Q (m) 7.08
A (m) 6.11
H (m) 3.86
I (s) 3.82
J (m) 3.68
K (ddt) 3.53
L (m) 2.84
M (m) 2.38
0.83
0.74
0.93
1.14
0.67
2.03
0.97
3.03
1.56
1.01
0.86
0.79
3.06
1.26
2.19
2.01
1.08
0.77
1.10
1.10
f1 (ppm)

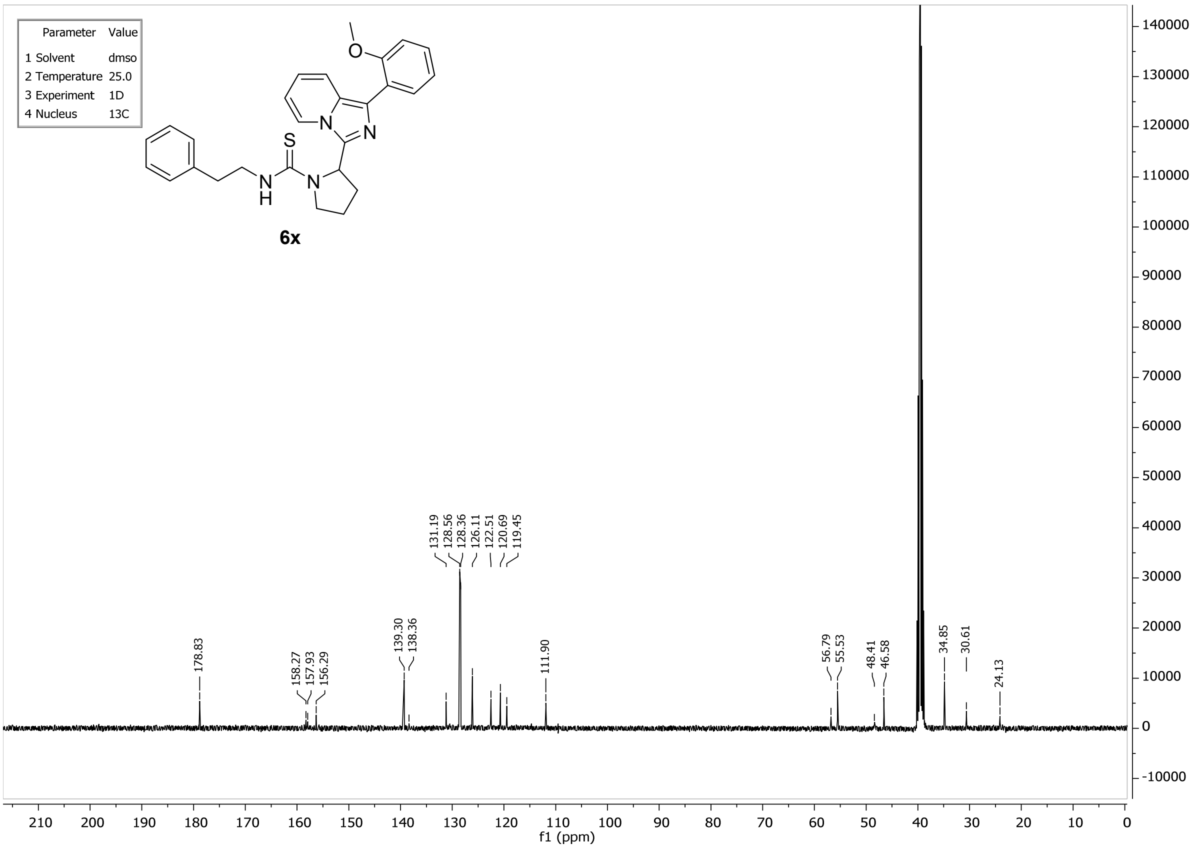
Parameter Value
1 Solvent dmso
2 Temperature 25.0
3 Experiment 1D
4 Nucleus 13C
6x
178.83
158.27
157.93
156.29
139.30
138.36
131.19
128.56
128.36
126.11
122.51
120.69
119.45
111.90
56.79
55.53
48.41
46.58
34.85
30.61
24.13
f1 (ppm)
